# Family-Wide Evaluation of Multiple C2 Domain and Transmembrane Region Protein in *Gossypium hirsutum*

**DOI:** 10.3389/fpls.2021.767667

**Published:** 2021-10-25

**Authors:** Qianqian Hu, Mengting Zeng, Miao Wang, Xiaoyu Huang, Jiayi Li, Changhui Feng, Lijie Xuan, Lu Liu, Gengqing Huang

**Affiliations:** ^1^Hubei Key Laboratory of Genetic Regulation and Integrative Biology, School of Life Sciences, Central China Normal University, Wuhan, China; ^2^Joint Center for Single Cell Biology, School of Agriculture and Biology, Shanghai Jiao Tong University, Shanghai, China; ^3^Institute of Cash Crops, Hubei Academy of Agricultural Sciences, Wuhan, China; ^4^Xinjiang Key Laboratory of Special Species Conservation and Regulatory Biology, College of Life Science, Xinjiang Normal University, Ürümqi, China

**Keywords:** cotton, main stem apex development, GhMCTP, gene expression, protein interaction, KNOX family protein

## Abstract

Multiple C2 domain and transmembrane region proteins (MCTPs) are a group of evolutionarily conserved proteins and show emerging roles in mediating protein trafficking and signaling transduction. Although, several studies showed that MCTPs play important roles during plant growth and development, their biological functions in cotton remain largely unknown. Here, we identify and characterize 33 *GhMCTP* genes from upland cotton (*Gossypium hirsutum*) and reveal the diverse expression patterns of *GhMCTPs* in various tissues. We also find that *GhMCTP7*, *GhMCTP12*, and *GhMCTP17* are highly expressed in the main stem apex, suggesting their possible roles in shoot development. Through analyzing different cotton species, we discover plant heights are closely related to the expression levels of *GhMCTP7*, *GhMCTP12*, and *GhMCTP17*. Furthermore, we silence the expression of *GhMCTP* genes using virus-induced gene silencing (VIGS) system in cotton and find that *GhMCTP7*, *GhMCTP12*, and *GhMCTP17* play an essential role in shoot meristem development. GhMCTPs interact with GhKNAT1 and GhKNAT2 and regulate meristem development through integrating multiple signal pathways. Taken together, our results demonstrate functional redundancy of GhMCTPs in cotton shoot meristem development and provide a valuable resource to further study various functions of GhMCTPs in plant growth and development.

## Introduction

The development of multicellular organisms is regulated by various signaling pathways. These signaling transduction events are mediated by extensive intercellular and intracellular membrane trafficking processes, which control the signal perception and the trafficking of signal molecules from one compartment to another. Thus, membrane trafficking regulates the processing, modification, and secretion of signal molecules, or conversely, their translocation to the nucleus (Shilo and Schejter, 2011; Van Norman et al., 2011).

C2 domain is one of the most prevalent eukaryotic lipid-binding domains and could serve as a docking module that targets proteins to a specific intracellular membrane (Nalefski and Falke, 1996; Cho and Stahelin, 2006). A large number of C2 domain-containing proteins have been identified, and most of them are involved in membrane trafficking and signal transduction (Corbalan-Garcia and Gómez-Fernández, 2014). Multiple C2 domain and transmembrane region proteins (MCTPs) are evolutionarily conserved in eukaryotic organisms, containing 3–4 C2 domains at the N-terminus and transmembrane regions at the C-terminus (Shin et al., 2005; Lek et al., 2012). MCTPs mediate the trafficking of key regulators and are essential for signaling transduction in diverse species, thus regulating various developmental processes (Liu et al., 2013; Genç et al., 2017). MCTPs also function as unique membrane tethers controlling endoplasmic reticulum (ER)-plasma membrane (PM) contact specifically at plasmodesmata and regulate cell-to-cell communication (Brault et al., 2019).

The function of MCTP was first identified in *Caenorhabditis elegans* in a high-throughput RNAi screening. Genetic mutants of *MCTP* were embryonic lethal (Maeda et al., 2001). In addition, different alleles of *mctp* mutants in *Drosophila* were isolated and showed to regulate various developmental processes, including larval development and neurotransmission, suggesting multiple roles of MCTPs in different developmental stages (Tunstall et al., 2012; Genç et al., 2017). However, the molecular functions of MCTPs in regulating these processes were still largely unknown.

The invertebrate organisms *C. elegans* and *Drosophila melanogaster* contain a single *MCTP* gene. In all plant lineages, the number of MCTP repertoire significantly increases, and each of MCTPs exhibits distinct or overlapping patterns of gene expression and subcellular protein localization (Liu et al., 2018a; Hao et al., 2020; Zhu et al., 2020), suggesting more diverse and specific functions of MCTPs in regulating multiple cellular and developmental processes. Several members of MCTP proteins have been identified in plants to mediate the intercellular and intracellular trafficking of various macromolecules. QUIRKY (QKY) and FT-INTERACTING PROTEIN 1 (FTIP1) belong to the *MCTP* family, mediate the trafficking of florigen protein FLOWERING LOCUS T (FT) from companion cells to sieve elements, thus regulating flowering time in *Arabidopsis* (Liu et al., 2012, 2019). QKY also interacts with and stabilizes a leucine-rich repeat receptor-like kinase SCRAMBLED (SCM), and is required for proper cell-type patterning and organogenesis (Trehin et al., 2013; Vaddepalli et al., 2014; Song et al., 2019; Mergner et al., 2020). In addition, two other MCTP proteins, FTIP3 and FTIP4, interact with key meristem regulator SHOOTMERISTEMLESS (STM) and control its subcellular localization and intercellular trafficking in the shoot apex, thus determining the fate of shoot apical meristem (Liu et al., 2018b).

Multiple C2 domain and transmembrane region proteins also regulate multiple developmental processes in other plant species. OsFTIP1, the closet ortholog of FTIP1 in rice, mediates the flowering transition by affecting the trafficking of RICE FLOWERING LOCUS T1 (RFT1) from companion cells to sieve elements (Song et al., 2017). OsFTIP1 also determines the nuclear localization of rice MOTHER OF FT AND TFL1 (OsMFT1) and promotes drought tolerance (Chen et al., 2021). Another MCTP protein in rice OsFTIP7 facilitates nuclear translocation of a homeodomain transcription factor, *Oryza sativa* homeobox 1 (OSH1), to determine the auxin-mediated anther dehiscence in rice (Song et al., 2018). ZmCpd33, the closet homolog of QKY in maize, promotes sucrose export from companion cells into sieve elements (Tran et al., 2019). DOFTIP1, the orchid orthologs of FTIP1, plays an important role in promoting flowering in the orchid *Dendrobium Chao Praya Smile* (Wang et al., 2017). These results demonstrate that MCTPs are involved in diverse protein trafficking events and regulate plant development.

Upland cotton (*Gossypium hirsutum*) is an important economic crop in the world, and it is the primary fiber crop and an important oil crop (John and Crow, 1992). The architecture of cotton plants is determined primarily by their plant heights, shoot branching patterns, and flowering patterns, all of which directly affect cotton planting strategies, yield, planting area, mechanized harvesting suitability, and cotton planting costs (Reinhardt and Kuhlemeier, 2002; Su et al., 2018). In *G. hirsutum*, *GhMCTPs* have been genome-widely identified and the gene expression patterns have been analyzed (Hao et al., 2020); however, the biological functions of GhMCTPs in *G. hirsutum* are still largely unknown.

In this study, we systematically investigated MCTPs in cotton and analyzed their gene expressions in various developing cotton tissues. We further characterized the function of *GhMCTP7*, *GhMCTP12*, and *GhMCTP17* in shoot development. GhMCTP7, GhMCTP12, and GhMCTP17 show distinct or overlapping subcellular localization patterns in *Nicotiana benthamiana* leaf epidermal cells. They interact with GhKNAT1 and GhKNAT2 and regulate shoot apical meristem development through integrating multiple signaling pathways. Our results demonstrate the functional redundancy of GhMCTPs in shoot development and reveal a gene regulatory framework that determines the meristem fate, providing a valuable resource for cotton architecture improvement.

## Materials and Methods

### Plant Materials

The seeds of upland cotton (*G. hirsutum “coker 312”*) were surface sterilized with 70% (v/v) ethanol for 1min, and then with 10% hydrogen peroxide for 2h, followed by washing with sterile water several times. The sterilized seeds were germinated on one-half strength Murashige and Skoog (MS) medium (12-h-light/12-h-dark cycle, 28°C), and seedlings were transplanted to the soil for further growth. The roots, stems, main stem apex (MSA, apex length is about 5mm), young leaves of three-leaf stage cotton plants, and 10DPA (days post-anthesis) cotton fiber after flowering were harvested for RNA extraction.

The shoot apexes (about 1cm) of eight upland cotton cultivars (*G. hirsutun* Okra, Emian JD1718, Lumian 1, Jimian 958, Emian JB2150, Emian SJA146, Emian JC1751, Zaosong2) were harvested for RNA extraction when these plants were flowering. The height of plants was calculated for each cultivar (*n*=50) when cotton bolls were open.

### Sequence Analysis

A BLASTP search was performed using the protein sequences of 16 *Arabidopsis* MCTPs as query sequences on the website of COTTONGEN^1^ by chosen the *G. hirsutum* (AD1) ZJUv2.1 proteins (totally 72,761 protein sequences). The proteins with high sequence similarity (E-value=0) were selected as putative GhMCTPs. All GhMCTP sequences were then manually searched against MotifScan,^2^ InterProScan,^3^ and SMART,^4^ to confirm the sequence containing the C2 domain and PRT_C domain. Finally, a total of 33 GhMCTP members were identified. The chromosomal location, amino acid length, protein molecular mass, and isoelectric point of the 33 GhMCTPs were analyzed using COTTONGEN^5^ and ExPASy ProtParam.^6^ DNA and protein sequences were analyzed using DNASTAR software (DNAStar, MD, United States).

### Phylogenetic Analysis

The protein sequences of 33 GhMCTPs were used as queries to identify GhMCTP homologs in *Gossypium raimondii* and *Gossypium arboretum* from COTTONGEN,^7^ and different plant species from Phytozome v12.^8^ A phylogenetic tree of deduced GhMCTP amino acid sequences was constructed using the neighbor-joining algorithm with default parameters, with 1,000 bootstrap replicates in MEGA_X_10.2.4 (Kumar et al., 2016).^9^

### Gene Structure and Chromosomal Mapping

The Gene Structure Display Server Program^10^ was used to draw the exon-intron structure of *GhMCTP* genes based on the full-length genome sequence and the corresponding coding sequences. Domain analysis of all MCTP proteins from various plant species was performed by InterProScan. The chromosomal location information of all MCTP genes was derived from the annotation information downloaded on the COTTONGEN (see footnote 5; Supplementary Table 1).^11^ Based on the location information of *GhMCTPs*, we manually drew the chromosome map using Photoshop software.

### Heat-Map Analysis of Gene Expression

The reads per kb per million reads (RPKM) values denoting the expression levels of *GhMCTP* genes were obtained from a comprehensive profile of the TM-1 transcriptome data (Trapnell et al., 2012; Zhang et al., 2015),^12^ and the expression data of main stem apex were generated in this study. A heat-map analysis was performed using *TBtools* (Chen et al., 2020).^13^

### Expression Analysis

Total RNA was extracted from cotton roots, stems, leaves, ovules, and the 10days fiber after flowering using the RNAprep Pure Plant kit (TIANGEN, Beijing, China) and reverse transcribed using Moloney Murine Leukemia Virus Reverse Transcriptase (Promega, Madison, Wisconsin, United States) according to the manufacturer’s instructions. Quantitative real-time PCR was performed using MJ Research DNA Engine Option 2 detection system with the fluorescent intercalating dye SYBR-Green (Toyobo). The relative expression levels were normalized to a cotton polyubiquitin gene (*GhUBI1*, GenBank accession no. EU604080).

A two-step PCR procedure was performed in all experiments using the previously described method (Huang et al., 2013). The expressions of the putative target genes were determined using the comparative cycle threshold method. To achieve optimal amplification, PCR conditions for each set of primers were optimized for annealing temperature and Mg^2+^ concentration. Data presented in the quantitative real-time PCR (qRT-PCR) analysis are the mean and SD of three biological replicates of plant materials and three technical replicates in each biological sample using gene-specific primers. Primers used for qRT-PCR are designed to target *GhMCTP-A* and *GhMCTP-D* simultaneously and are listed in Supplementary Table 2.

### Yeast Two-Hybrid Assay

The N-terminal fragments of *GhMCTP7*, *GhMCTP12*, and *GhMCTP17* devoid of the sequences encoding the transmembrane regions were amplified and cloned into pGADT7 (Prey vector, Clontech). The coding sequences of *GhKNAT1* and *GhKNAT2* were amplified and cloned into pGBKT7 (Bait vector, Clontech). The prey and bait vectors were transformed into AH109 and Y187 cells, respectively. After mating, all transformed cells were grown on a Synthetic Defined-Ade/-His/-Trp/-Leu medium for interaction tests. Primers used are listed in Supplementary Table 2.

### Luciferase Complementation Imaging Assay

The coding sequences of *GhMCTP7*, *GhMCTP12*, and *GhMCTP17* were amplified and cloned into the N-terminal Luciferase fusion vector JW771-N-terminal luciferase fragment (nLUC). The coding sequences of *GhKNAT1* and *GhKNAT2* were amplified and cloned into the C-terminal Luciferase fusion vector JW772-C-terminal luciferase fragment (cLUC). The resulting plasmids were transformed into *Agrobacterium* cells, which were infiltrated into *N. benthamiana* leaves. The luciferase complementation imaging (LCI) assay was performed as described previously (Chen et al., 2008). The LUC luminescence signal was visualized using a cryogenically cooled CCD camera (Night Shade LB985, Berthold Technologies) and indiGO software. Primers used are listed in Supplementary Table 2.

### *Agrobacterium tumefaciens*-Mediated VIGS

Overnight *Agrobacterium* cultures with the desired tobacco rattle virus (TRV) vectors, including *TRV2:GhMCTP7*, *TRV2:GhMCTP12*, *TRV2:GhMCTP17*, and *TRV2:GhKNAT1/2*, were infiltrated into two fully expanded cotyledons of 10-day-old cotton plants grown at 22–24°C as described previously (Gao et al., 2013). At least 15 plants were inoculated for each construct. The *TRV2:GhCLA1* construct was included as a visual marker for virus-induced gene silencing (VIGS) efficiency. To simultaneously silence the expression of *GhMCTP7*, *GhMCTP12*, and *GhMCTP17*, *Agrobacterium* cultures containing *TRV2:GhMCTP7*, *TRV2:GhMCTP12*, and *TRV2:GhMCTP17* were mixed and infiltrated. Primers used are listed in Supplementary Table 2.

## Results

### Identification of MCTP Genes in Cotton

**Figure 1:**
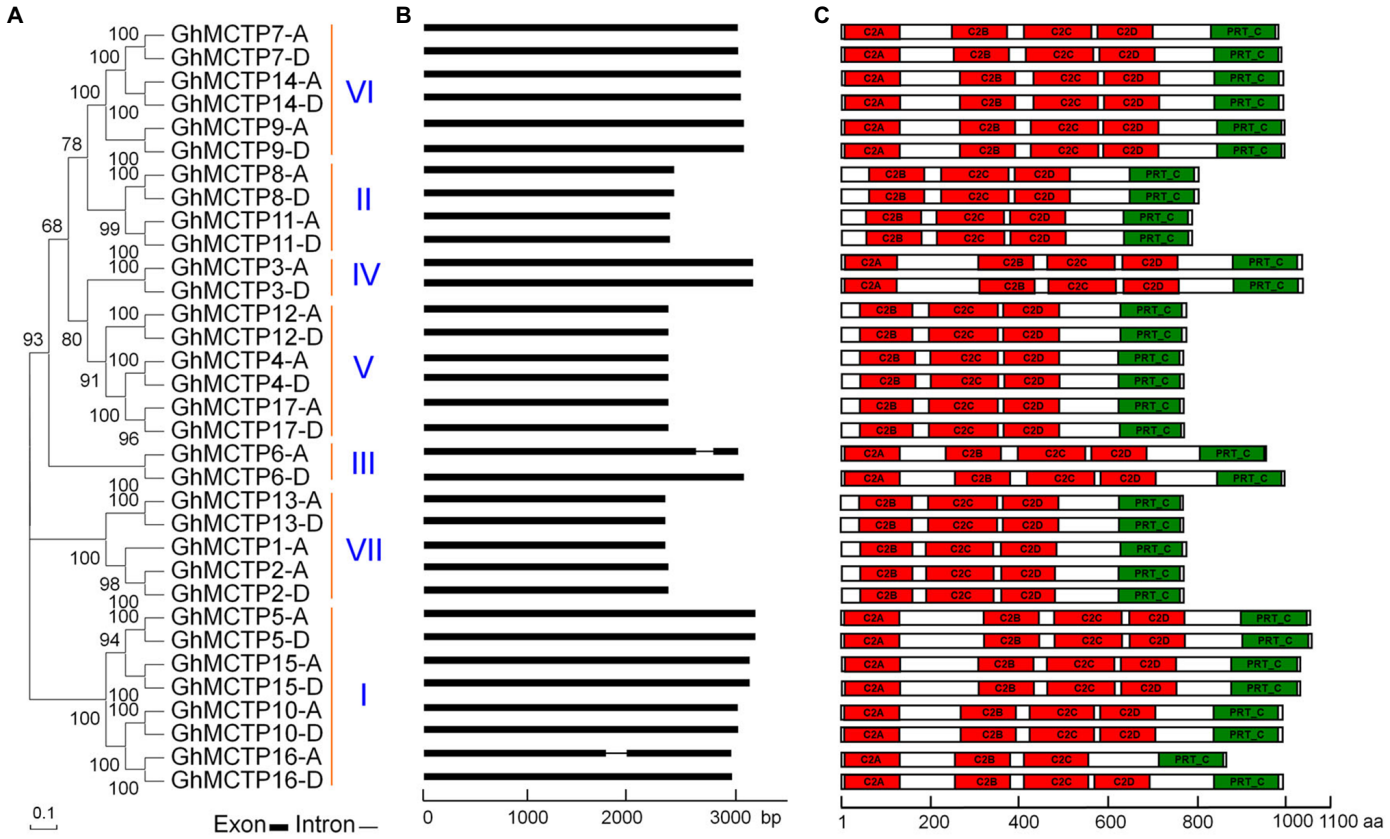
Characterization of multiple C2 domain and transmembrane region proteins (*MCTP*) family proteins in upland cotton (*Gossypium hirsutum*). **(A)** Thirty-three GhMCTP proteins are classified into seven groups based on phylogenetic analysis of MCTP proteins in *G. hirsutum*. The phylogenetic tree was generated with MEGA X using the neighbor-joining algorithm. Numbers on the major branches indicate bootstrap values (>50%) in 1,000 replicates. **(B)** Schematic diagrams showing the gene structures of *GhMCTP* genes. The coding regions are indicated by black boxes. The intron is represented by a black line. **(C)** Protein motif analysis of GhMCTPs. The prediction of protein motifs is based on InterProScan. C2 domain and Phosphoribosyltransferase C-terminal are labeled as red and green boxes, respectively. bp, base pair; aa, amino acids.

As MCTPs were first identified in *Arabidopsis* in plants (Liu et al., 2018a), we used 16 MCTP protein sequences in *Arabidopsis* as queries to search against the protein database of *G. hirsutum* to identify putative GhMCTPs in cotton using the BLASTP program in COTTONGEN [*G. hirsutum* (AD1) ZJUv2.1 proteins (72761)]. The identified MCTP members were further used as queries to search for other possible MCTP proteins in *G. hirsutum*. All these putative GhMCTP proteins were subjected for domain analysis to confirm that all these candidates contain 3–4 N-terminal C2 domains and phosphoribosyltransferase C-terminal region (PRT_C). Finally, we identified a total of 33 GhMCTP proteins in *G. hirsutum* (Figure 1).

We classified these 33 GhMCTPs into seven clades through phylogenetic analysis based on multiple sequence alignment (Figure 1A). In addition, we aligned all these GhMCTP protein sequences and identified 16 pairs of MCTP protein with high sequence similarity. The members from each pair are originated from the cotton A subgenome (At, where ‘t’ stands from tetraploid) and D subgenome (Dt), respectively. The GhMCTPs were therefore classified as GhMCTP-A and GhMCTP-D. The members in these two subgroups were further designated according to their locations on the chromosome (Supplementary Figure 1; Supplementary Table 1), according to the naming principle in other cotton genome studies (Paterson et al., 2012; Huang et al., 2020).

We analyzed the gene structures of all *GhMCTPs* and found most of *GhMCTP* genes do not contain introns (Figure 1B). Similar to MCTPs in *Arabidopsis*, most GhMCTP proteins contain 1–4 C-terminal transmembrane regions except for GhMCTP3 (Supplementary Figure 2), suggesting the functional conservation and divergence among MCTPs. We also searched the MCTP proteins in the diploid cotton *Gossypium arboreum* and *Gossypium raimondii* (Paterson et al., 2012; Huang et al., 2020), and identified 17 and 18 MCTP members from *G. arboreum* genome (A-genome) and *G. raimondii* genome (D-genome), respectively (Supplementary Table 1).

### Phylogenetic Analysis of MCTP Homologs in Plants

**Figure 2:**
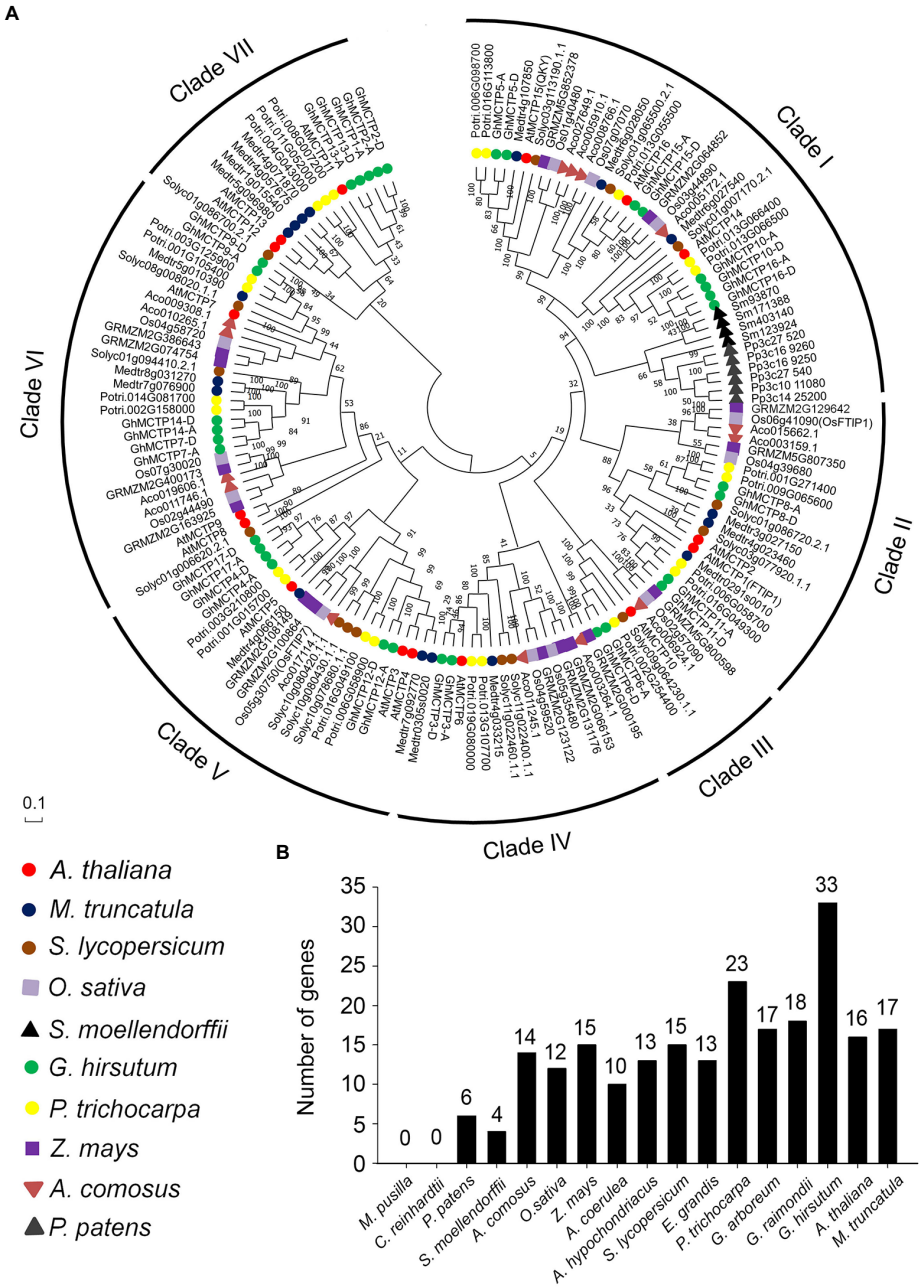
Polygenetic relationships of MCTP homologs in different plant species. **(A)** Proteins from 10 different species (*Arabidopsis thaliana*, *Medicago truncatula*, *Solanum lycopersicum*, *Oryza sativa*, *Selaginella moellendorffii*, *G. hirsutum*, *Populus trichocarpa*, *Zea mays*, *Ananas comosus*, and *Physcomitrella patens*) are indicated by different icons and are classified into seven groups. All available gene names are also indicated. The level of statistical support was conducted by neighbor-joining method, and numbers on the major branches indicate bootstrap values. **(B)** Numbers of *MCTP* genes in different species.

To identify MCTP homologs in plant genomes, we performed BLASTP or TBLASTN search in protein and genome databases in Phytozome 12 using MCTP1 as a query sequence. We obtained MCTP homologs in different land plants and algae including dicotyledons (*G. hirsutum*, *G. arboreum*, *G. raimondii*, *Arabidopsis*, *Aquilegia coerulea*, *Amaranthus hypochondriacus*, *olanum lycopersicum*, *Eucalyptus grandis*, *Populus trichocarpa*, and *Medicago truncatula*), monocotyledons (*Ananas comosus*, *Oryza sativa*, and *Zea mays*), moss (*Physcomitrella patens*), fern (*Selaginella moellendorffii*), and algaes (*Chlamydomonas reinhardtii* and *Micromonas pusilla*; Supplementary Table 3). To understand the evolutionary relationships among MCTPs in plants, MCTP homologs in different species were analyzed in detail using neighbor-joining and maximum likelihood methods, and the unrooted phylogenetic tree was constructed (Figure 2A).

The MCTP homologs could be classified into seven clades, namely clade I to clade VII. Each clade contains MCTPs from eudicot plants (Figure 2A). The clade I was the largest branch containing 41 members, which are from different plant species including eudicots, monocots, fern, and moss. Clade III is the smallest one with only eight members. Furthermore, clade VII is specific for eudicots, and clade I is the only clade containing MCTPs from moss (*P. patens*) and fern (*S. moellendorffii*; Figure 2A). It is noteworthy that MCTPs exist in many plant lineages (Figure 2B), suggesting the fundamental roles of MCTPs in plant development. The number of MCTPs is greatly expanded in dicotyledons and monocotyledons (Figure 2B), indicating that the family members of MCTPs among species increase substantially after several rounds of whole-genome duplication, and may evolve to generate functional specialized MCTPs to respond to changing environmental stimuli.

### Expression Analysis of *GhMCTPs* in Upland Cotton

**Figure 3:**
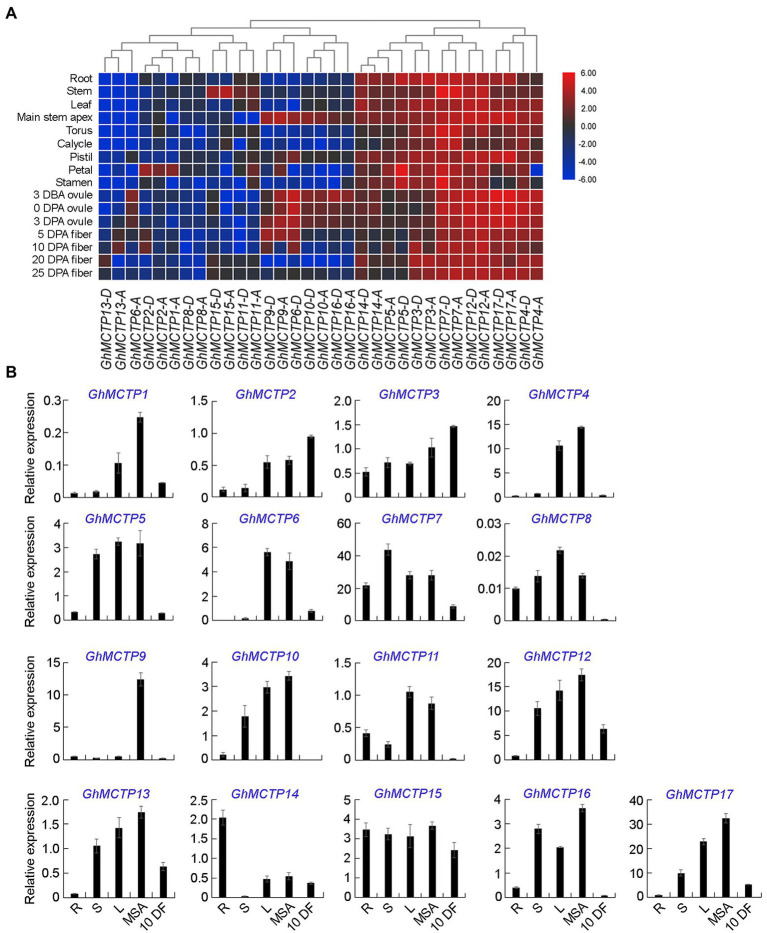
Expression patterns of *GhMCTPs* in upland cotton. **(A)** Heat map analysis of *GhMCTP* gene expressions in different organs of upland cotton. The relative fold changes in gene expression for all *GhMCTP* genes were compared. The color from blue to red indicates low to high expression. DBA, days before-anthesis; DPA, days post-anthesis. **(B)** Quantitative real-time PCR (qRT-PCR) analysis of 17 *GhMCTPs* in various tissues of upland cotton. Results were normalized against the expression level of *GhUBI1*. R, root; S, stem; L, leaf; MSA, main stem apex; and 10 DF, fibers at 10-day post-anthesis. Error bars indicate SD.

To identify the potential roles of GhMCTPs in cotton development, the expression patterns of all *GhMCTPs* were investigated in various cotton tissues, including root, stem, leaf, main stem apex, torus, calycle, pistil petal, and stamen, through analyzing previously published transcriptome datasets (You et al., 2017). We also analyzed the expression of all *MCTPs* in ovules and fibers at several developmental stages (Figure 3A). Based on their expression profiles, *GhMCTPs* were generally divided into two groups. One group of *GhMCTPs* was highly expressed in almost all tissues, including *GhMCTP3-A/D*, *GhMCTP4-A/D*, *GhMCTP5-A/D*, *GhMCTP7-A/D*, *GhMCTP12-A/D*, *GhMCTP14-A/D*, and *GhMCTP17-A/D*. Members of the other groups were tissue-preferred genes (Figure 3A). For example, *GhMCTP1-A* and *GhMCTP2-A/D* were preferentially expressed in petals. *GhMCTP6-D*, *GhMCTP9-A/D*, *GhMCTP10-A/D*, and *GhMCTP16-A/D* were highly expressed in the main stem apex and ovules at early stages (Figure 3A). This tissue/organ-preferred expression pattern indicates that these MCTPs may function in specific developmental stages.

We further isolated different cotton tissues, including root, stem, leaf, main stem apex, and fibers at 10-day post-anthesis, and carried out qRT-PCR to investigate expression profiles of all *GhMCTPs* (Figure 3B). The results revealed that *GhMCTP* genes were expressed in all organs and tissues detected. Furthermore, we found that *GhMCTPs* from different subfamilies exhibited different expression patterns (Figure 3), suggesting that *GhMCTPs* might be involved in different cotton developmental processes.

### GhMCTP7, GhMCTP12, and GhMCTP17 Function Additively to Regulate Cotton Shoot Meristem Development

The majority of *GhMCTP* genes were highly detected in the main stem apex (Figures 3A,B), suggesting their possible roles in meristem development. In *Arabidopsis*, two MCTP proteins, FT INTERACTING PROTEIN 3 (FTIP3) and FTIP4 have been reported to play an essential role in mediating shoot meristem development, thus determining the overall plant architecture (Liu et al., 2018b). GhMCTP12-A and GhMCTP12-D were the closest homologs of FTIP3 and FTIP4 in *G. hirsutum* (Figure 2A). In cotton, GhMCTP7-A/D and GhMCTP17-A/D showed sequence similarity with GhMCTP12-A/D (Figure 2A), and *GhMCTP7*, *GhMCTP12*, and *GhMCTP17* were all highly expressed in the main stem apex (Figure 3). These results suggest that they are potential candidates to regulate meristem development.

**Figure 4:**
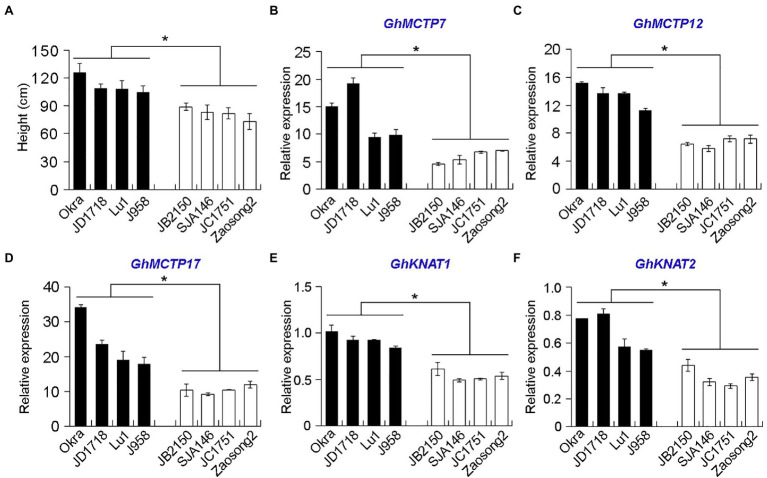
The expression levels of *GhMCTP7/12/17* and *GhKNAT1/2* are correlated with the average plant height (cm) of cotton varieties. **(A)** Comparison of plant height of eight allotetraploid cotton cultivars (*G. hirsutun*). The heights of plants are calculated when cotton bolls were opening. The cotton varieties are divided into two groups according to their plant heights. The varieties over 100cm in height or under 100cm are grouped and are shown in black bars or white bars, respectively. **(B-F)** Quantitative real-time PCR analysis of *GhMCTP7* **(B)**, *GhMCTP12* **(C)**, *GhMCTP17* **(D)**, *GhKNAT1* **(E)**, and *GhKNAT2* **(F)** in main stem apex of different cotton species. The X-axis represents different upland cotton species, while the Y-axis represents gene relative expressions. Results were normalized against the expression level of *GhUBI1*. Error bars indicate SD of three biological replicates (^*^p<0.05).

The effect of different cotton cultivars on plant height was highly significant. We chose eight allotetraploid cotton cultivars and divided them into two groups based on their plant heights (Figure 4A). To understand the roles of GhMCTPs in shoot development, we examined the expressions of *GhMCTP7*, *GhMCTP12*, and *GhMCTP17* in the shoot apex of selected allotetraploid cotton cultivars and found that the expression levels of *GhMCTP7*, *GhMCTP12*, and *GhMCTP17* were correlated with their plant heights (Figures 4B–D).

**Figure 5:**
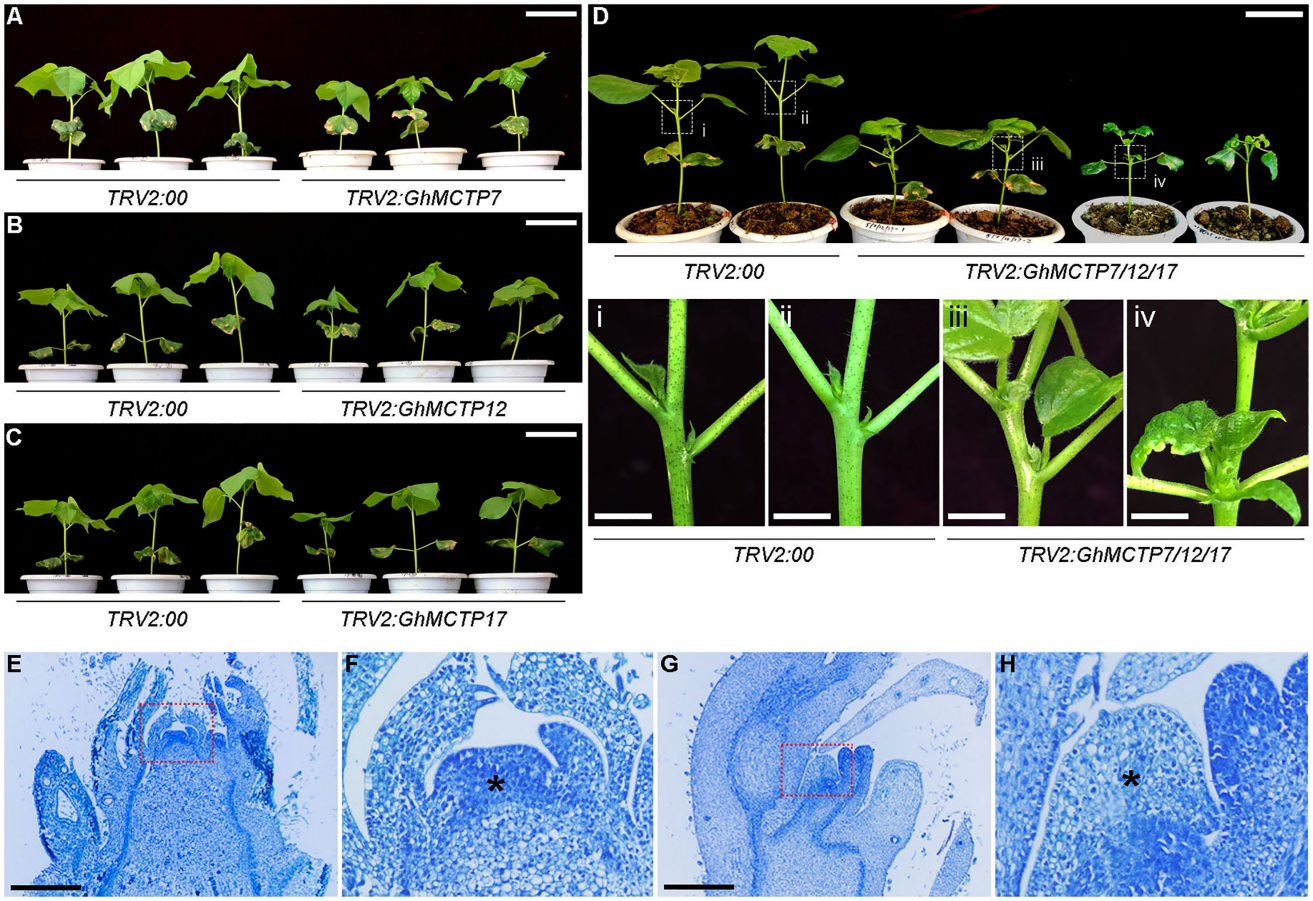
Phenotypic analyses of *TRV2:GhMCTPs* plants. **(A–C)** Plant height comparison of 3–4 leaf stage *TRV2:GhMCTP7* **(A)**, *TRV2:GhMCTP12* **(B)**, and *TRV2:GhMCTP17* **(C)** plants with *TRV2:00* plants (negative control group). Scale bars=5cm. **(D)** Plant height comparison of four leaf stage *TRV2:GhMCTP7/12/17* with *TRV2:00* plants (upper panel). The lower panels show the magnified view of boxes indicated in the upper panel. Scale bars=5cm (top) and 1cm (bottom). **(E–H)** Median longitudinal section of main inflorescence shoot apices of *TRV2:00* **(E)** and *TRV2:GhMCTP7/12/17* **(G)** plants. Scale bars=1μm. **(F,H)** The magnified views of boxes are indicated in **(G)** and **(H)**, respectively. Asterisks indicate main inflorescence meristems.

Virus-induced gene silencing is a powerful reverse genetic technology for quick functional characterizations of plant genes, which serves as an alternative to mutant collection or creating stable transgenic lines (Gao et al., 2013; Lange et al., 2013; McGarry et al., 2016). To further understand the biological functions of *GhMCTPs* in shoot development, we silenced the gene expression of *GhMCTP7*, *GhMCTP12*, and *GhMCTP17* using TRV-based VIGS technique. Real-time quantitative PCR assays showed that the expressions of *GhMCTPs* were downregulated in cotton main stem apex of *TRV2:GhMCTP* plants compared to negative control plants treated with *TRV2:00* (Supplementary Figure 3); however, most of the VIGS-mediated gene silencing lines exhibited mild dwarf phenotype compared to those of negative control plants (Figures 5A–C). We reasoned that GhMCTP7, GhMCTP12, and GhMCTP17 might play a redundant role to regulate meristem development. Thus, we conducted a VIGS assay to simultaneously silence the expression of *GhMCTP7*, *GhMCTP12*, and *GhMCTP17* (Supplementary Figure 3B). The resulting plants exhibited a dwarf phenotype, with down-curly leaves and occasionally shoot branching (Figure 5D; Supplementary Figure 4). Furthermore, the longitudinal sections showed that *TRV2:GhMCTP7/12/17* plants had a narrow dome-shaped meristem, and the organs generated at the flanking of the meristems were also malformed and disordered positioned (Figures 5E–H). These results substantiate that *GhMCTP7*, *GhMCTP12*, and *GhMCTP17* are essential for shoot meristem development.

### Subcellular Localization of GhMCTP7, GhMCTP12, and GhMCTP17

**Figure 6:**
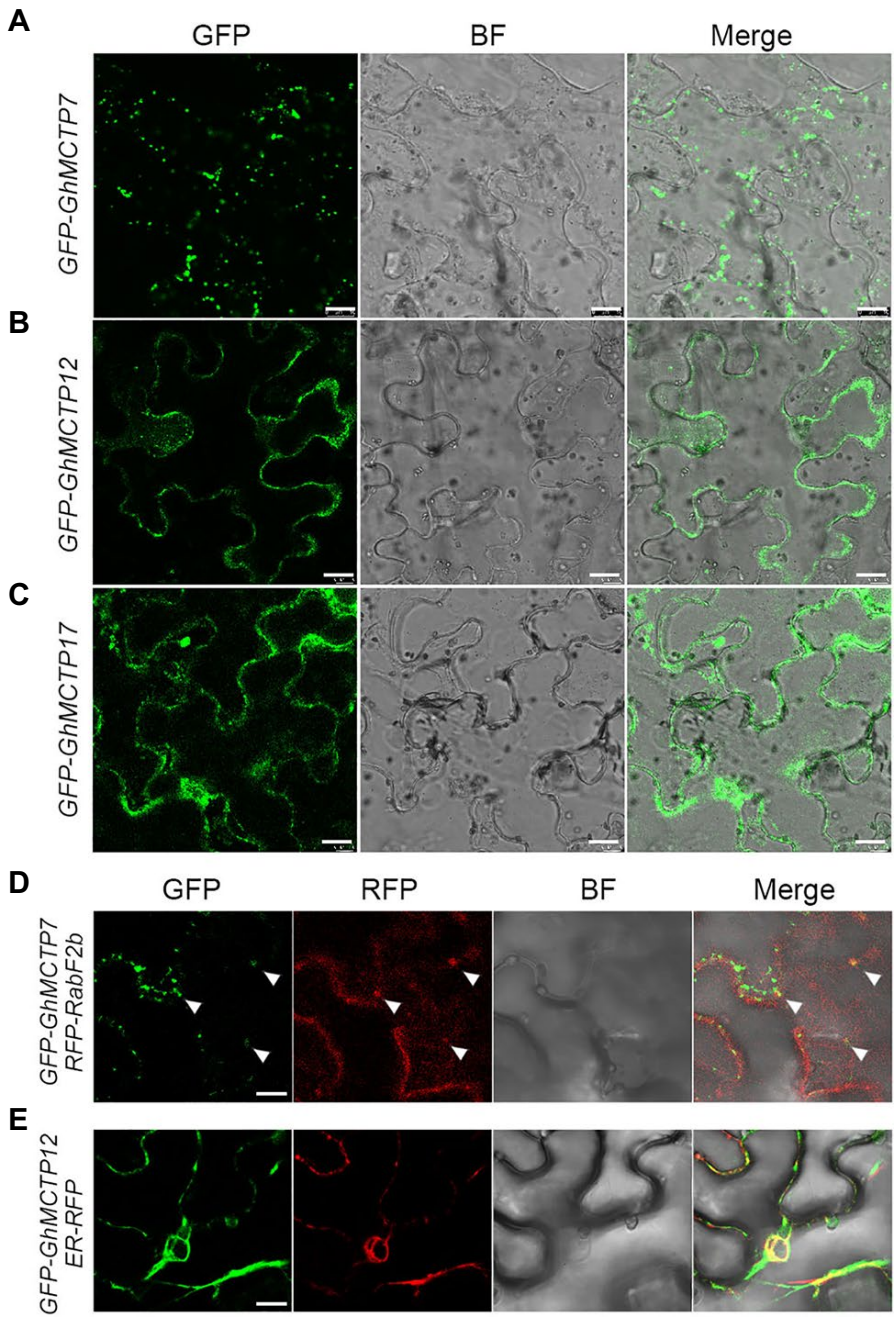
Subcellular localization of green fluorescent protein (GFP)-GhMCTP7/12/17 in *Nicotiana benthamiana* leaf epidermal cells. **(A–C)** Representative GFP fluorescence images of GFP-GhMCTP7 **(A)**, GFP-GhMCTP12 **(B)**, and GFP-GhMCTP17 **(C)**. Scale bars=50μm. **(D)** Colocalization of GFP-GhMCTP7 with an endosome marker (RFP-RabF2b). **(E)** Co-expression of GFP-GhMCTP12 with an ER marker (ER-RFP). Scale bars=10μm.

To determine the subcellular localization of GhMCTP7, GhMCTP12, and GhMCTP17, we transiently expressed their full-length open reading frames fused with the green fluorescent protein (GFP)-MCTPs reporter in *N. benthamiana* leaf epidermal cells and observed two different types of subcellular localization patterns (Figure 6; Supplementary Figure 5). GhMCTP7 was localized to puncta-like structures in the cytosol within cells (Figure 6A; Supplementary Figure 5). We then coexpressed *35S:GFP-GhMCTP7* with the fluorescence-tagged endosome marker *35S:RFP-RabF2b* (Jaillais et al., 2006), and observed GhMCTP7 was partially localized in endosomal compartments (Figure 6D). GhMCTP12 and GhMCTP17 were localized in whole cells and substantially colocalized with an ER marker, RFP-HDEL (Figures 6B,C,E; Nelson et al., 2007). These results suggest that GhMCTP7, GhMCTP12, and GhMCTP17 function coordinately to regulate intercellular signaling and control cotton development.

### GhMCTP7/12/17 Interact With GhKNAT1/2 to Regulate Shoot Development

FTIP3 and FTIP4, two MCTP proteins in *Arabidopsis*, are required for shoot apical meristem development through mediating subcellular localization and intercellular trafficking of STM (Liu et al., 2018b). GhMCTP7, GhMCTP12, and GhMCTP17 shared high sequence similarities with FTIP3 and FTIP4 in *Arabidopsis* (Figure 2A). Our finding on the tissue expression patterns of *GhMCTP7*, *GhMCTP12*, and *GhMCTP17* in the shoot apex and their roles in cotton meristem development prompted us to investigate whether GhMCTP7, GhMCTP12, and GhMCTP17 interact with KNOTTED1 (KN1)-like homeobox (KNOX) family proteins in cotton, like their counterparts in *Arabidopsis* (Liu et al., 2018b).

**Figure 7:**
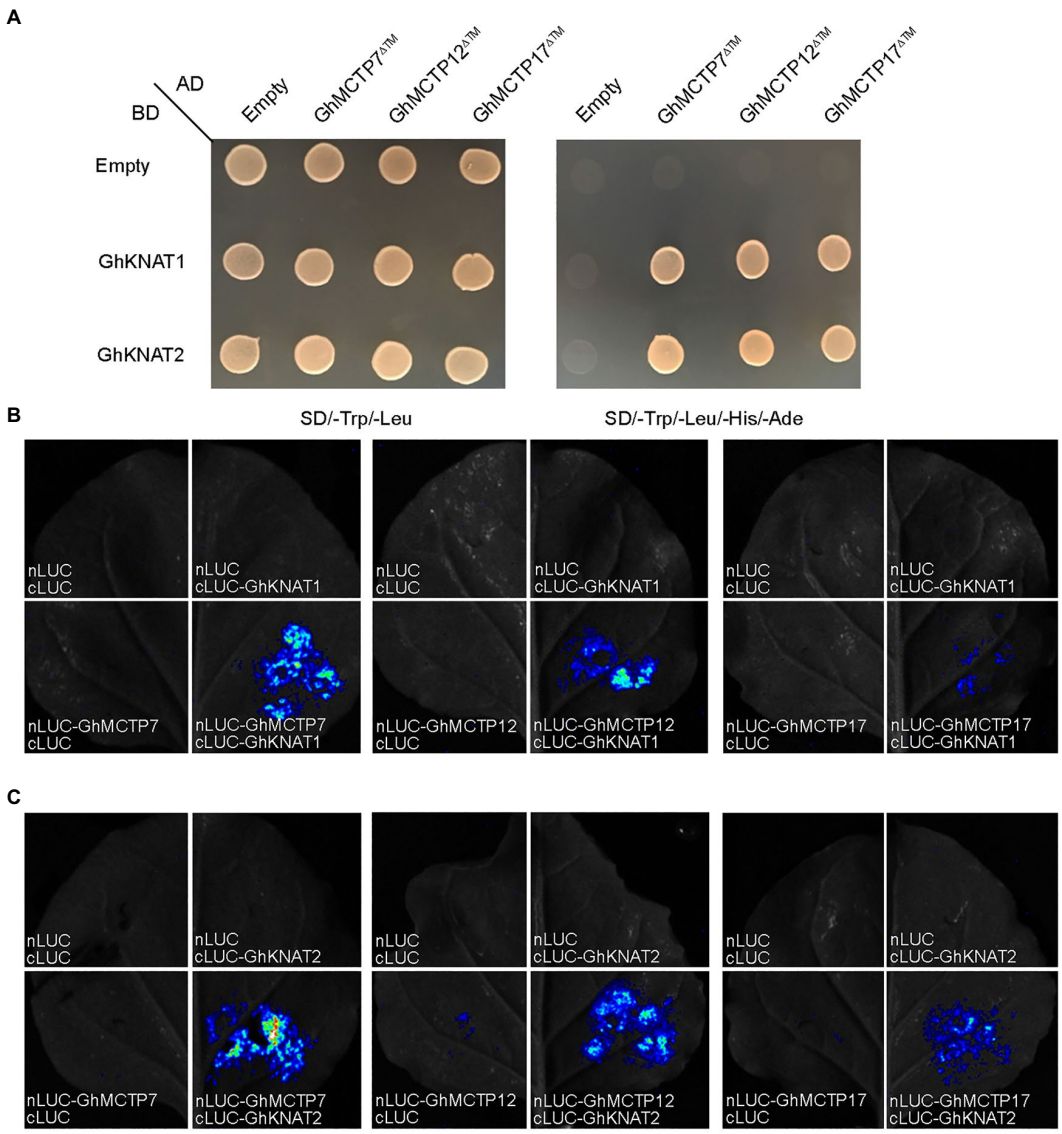
GhMCTP7/12/17 interact with GhKNAT1/2. **(A)** Yeast two-hybrid assay showing the interaction between GhMCTP7/12/17^∆^™ and GhKNAT1/2. Transformed yeast cells were grown on SD -Ade/-His/-Leu/-Trp medium. **(B,C)** Luciferase complementation imaging (LCI) assays showing that GhMCTP7/12/17 interact with GhKNAT1 **(B)** and GhKNAT2 **(C)** in *N. benthamiana* leaves. Fluorescence signal intensities represent their protein interaction intensities.

First, we examined the expression profiles of all *GhKNAT* genes in different tissue of upland cotton. The results showed that *GhKNAT1* and *GhKNAT2* were highly expressed in the main stem apex (Supplementary Figure 6). We then investigated whether GhMCTP7/12/17 interact with GhKNAT1/2. We conducted a detailed analysis of protein interaction between GhMCTP7/12/17 and GhKNAT1/2. Yeast two-hybrid assays revealed that the GhMCTP7/12/17^∆^™, a truncated GhMCTP7/12/17 devoid of the transmembrane region, interacted with GhKNAT1/2 (Figure 7A). To test the interaction between GhMCTP7/12/17 and GhKNAT1/2 in planta, we performed luciferase complementation imaging (LCI) assays. We coexpressed nLUC-GhMCTP7/12/17 and cLUC-GhKNAT1/2 and detected fluorescence signals in *N. benthamiana* leaves (Figures 7B,C). These results demonstrate the protein interactions between GhMCTP7/12/17 and GhKNAT1/2 in plants.

**Figure 8:**
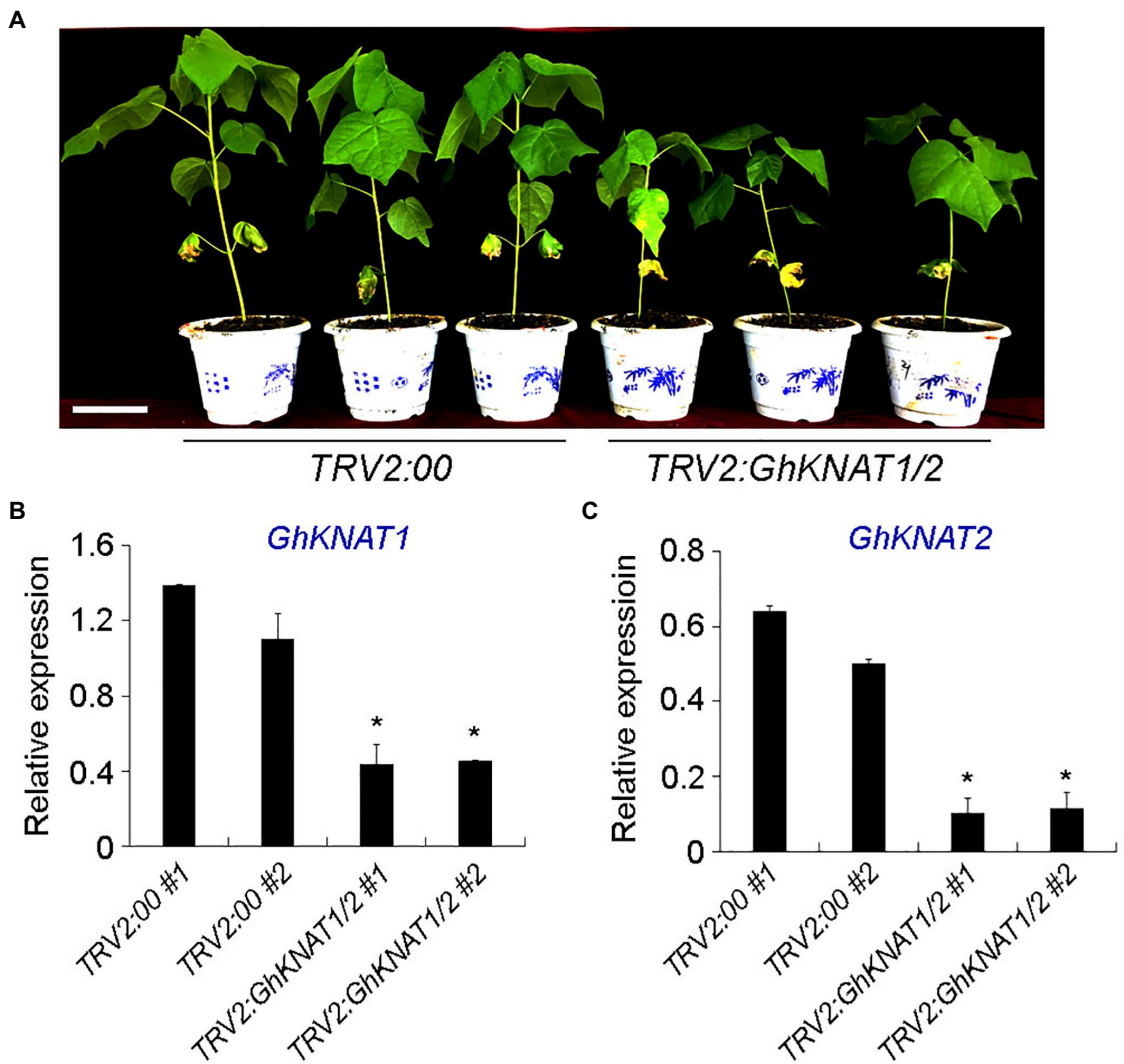
Phenotypic analyses of *TRV2:GhKNAT1/2* plants. **(A)** Plant height comparison of 5–6 leaf stage *TRV2:GhKNAT1/2* plants with *TRV2:00* plants (negative control group). **(B,C)** Expression analysis of *GhKNAT1* and *GhKNAT2* in main stem apex of *TRV2:00* **(B)** and *TRV2:GhKNAT1/2* **(C)** plants, respectively. Results were normalized against the expression level of *GhUBI1*. Error bars indicate SD of three biological replicates (^*^p<0.05).

Comparing the expression levels of *GhKNAT1* and *GhKNAT2* in the shoot apex of selected allotetraploid cotton cultivars, we also found that the expression levels of *GhKNAT1* and *GhKNAT2* were higher in the taller cotton variants (Figures 4E,F), suggesting that GhKNAT1 and GhKNAT2 are the potential regulators for meristem development. Then, we further investigated the effects of GhKNAT1 and GhKNAT2 on meristem development. We silenced the expression of *GhKNAT1* and *GhKNAT2* using the VIGS in soil-grown upland cotton. Most of these *TRV2:GhKNAT1/2* plants exhibited a dwarf phenotype (Figure 8A). Examination of some selected plants revealed that the expressions of *GhKNAT1* and *GhKNAT2* were downregulated in these *TRV2:GhKNAT1/2* plants (Figure 8B), suggesting that GhKNAT1 and GhKNAT2 regulate meristem development.

### GhMCTP7/12/17 Are Involved in the Regulation of Multiple Signal Pathways

KNOTTED1-like homeobox transcription factors promote meristem function through directly targeting various transcription factors and genes participating in hormone pathways (Bolduc et al., 2012). Given that GhMCTP7/12/17 may regulate meristem development through mediating the function of GhKNAT1/2, we further conducted a detailed expression analysis to investigate the involvement of GhMCTP7/12/17 in regulating key regulators in meristem development and hormone signaling pathway.

**Figure 9:**
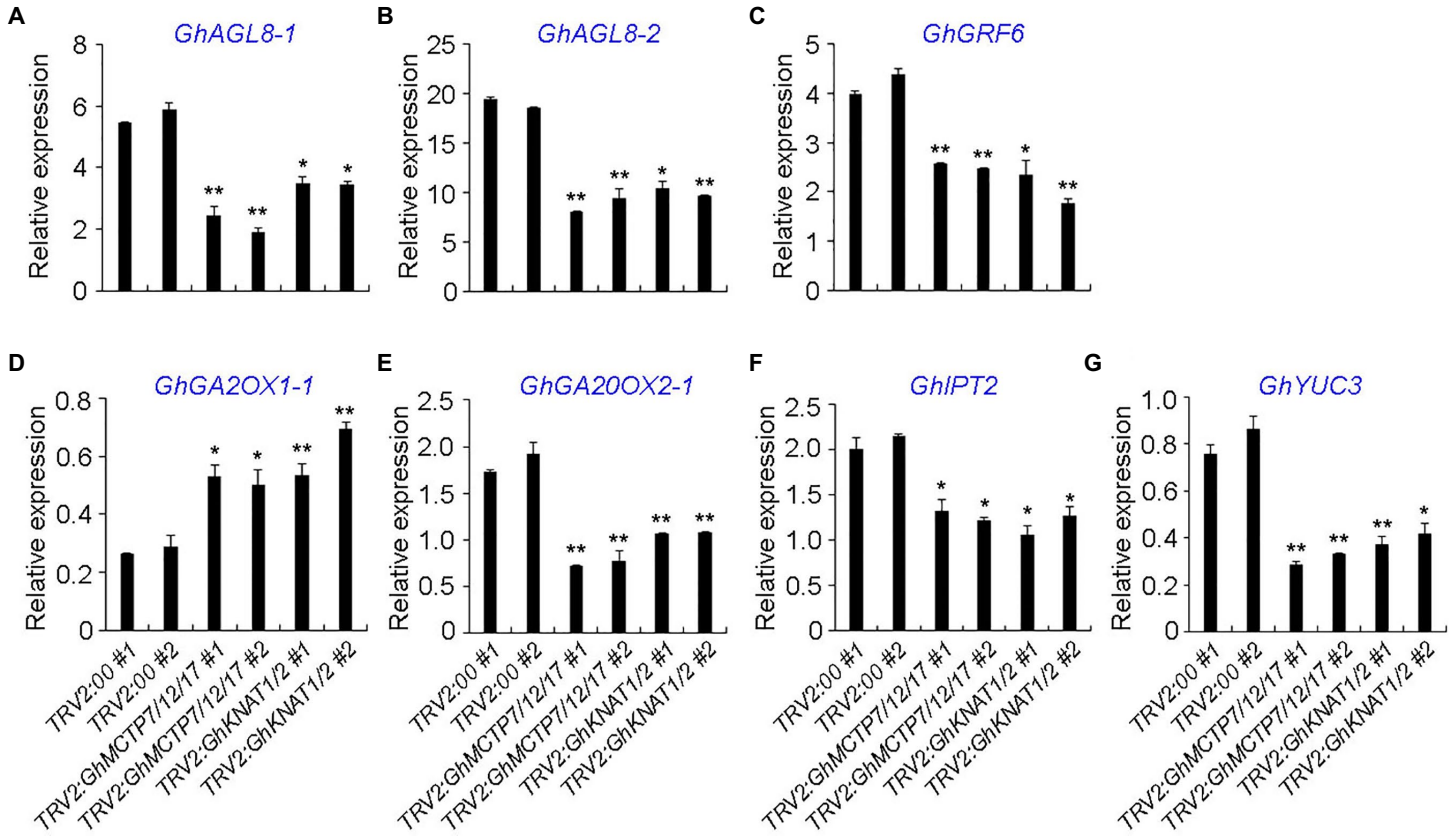
Quantitative analysis of multiple shoot meristem regulators in main stem apex of *TRV2:00*, *TRV2:GhMCTP7/12/17*, and *TRV2:GhKNAT1/2* plants. The expression levels of *GhALG8-1* **(A)**, *GhAGL8-2* **(B)**, *GhGRF6* **(C)**, *GhGA2OX1-1* **(D)**, *GhGA20OX2-1* **(E)**, *GhIPT2* **(F)**, and *GhYUC3* **(G)** in *TRV2:GhMCTP7/12/17* and *TRV2:GhKNAT1/2* plants. Results were normalized against the expression level of *GhUBI1*. Error bars indicate SD of three biological replicates (^*^p<0.05; ^**^p<0.01).

To further characterize the functions of GhMCTP7/12/17 and GhKNAT1/2, the expressions of selected genes, which counterparts in *Arabidopsis* are key regulators in shoot meristem development and genes in various hormone signaling pathways, were analyzed. First, key regulators with high expression in the main stem apex were selected based on the public expression data (Supplementary Figures 7–10). We then carried out qRT-PCR to examine the expressions of the selected genes in the main stem apex of *TRV2:00*, *TRV2:GhMCTP7/12/17*, and *TRV2:GhKNAT1/2* plants (Figure 9). *Gh AGAMOUS-LIKE 8-1/-2* (*GhAGL8-1/-2*), the homologs of *APETALA1* (*AP1*) in cotton, regulate plant height and early maturity of cotton (Su et al., 2018). GENERAL REGULATORY FACTORs (GRFs) play important roles in flowering regulation and meristem development (Sang et al., 2021). We found that the expressions of *GhAGL8-1/2* and *GhGRF6* were downregulated in both *TRV2:GhMCTP7/12/17* and *TRV2:GhKNAT1/2*, suggesting the delayed determination of floral meristem identity (Figures 9A,B).

KNOTTED1-like homeobox protein promotes meristem development through repressing gibberellin (GA) biosynthesis and activating cytokinin (CK) pathway (Jasinski et al., 2005). KNOX protein also regulates the expressions of auxin-related genes (Bolduc et al., 2012), suggesting that KNOX protein integrates multiple phytohormone signaling pathways to regulate meristem development. We studied the expression of cytokinin biosynthesis and signaling genes, auxin biosynthesis and signaling genes, GA biosynthesis and catabolism genes in both *TRV2:GhMCTP7/12/17* and *TRV2:GhKNAT1/2*. We observed that the expression of GA catabolic enzyme *GhGA2OX1-1* was elevated in both *TRV2:GhMCTP7/12/17* and *TRV2:GhKNAT1/2*, whereas GA biosynthesis gene *GhGA20OX2-1*, CK biosynthesis gene *Gh ISOPENTENYLTRANSFERASE 1* (*GhIPT1*), and auxin biosynthesis gene *Gh YUCCA3* (*GhYUC3*) were consistently downregulated in *TRV2:GhMCTP7/12/17* and *TRV2:GhKNAT1/2* (Figures 9E–G). These results suggest that MCTP7/12/17 regulate meristem development partially through GhKNAT1/2-mediated regulatory pathway.

We also observed that some other regulators in meristem development and phytohormone signaling pathway in main stem apex of *TRV2:00*, *TRV2:GhMCTP7/12/17*, and *TRV2:GhKNAT1/2* plants (Supplementary Figure 11). Stem cell regulators *Gh AINTEGUMENTA-1/-2/-3* (*GhANT-1/-2/-3*), *Gh GROWTH-REGULATING FACTOR 1* (*GhGRF1*), and *Gh GRF1-INTERACTING FACTOR 3* (*GhGIF3*) were downregulated in *TRV2:GhMCTP7/12/17* plants (Mudunkothge and Krizek, 2012; Kim and Tsukaya, 2015); however, their expressions in *TRV2:GhKNAT1/2* plants were not changed (Supplementary Figures 11A–E). In addition, we observed that, although the expressions of *Gh CLAVATA3/ESR-RELATED 27* (*GhCLE27*) and *Gh KNOTTED1-LIKE HOMEOBOX GENE 4* (*GhKNAT4*) were consistently downregulated in both *TRV2:GhMCTP7/12/17* and *TRV2:GhKNAT1/2* plants, the downregulation of *GhKNAT4* and *GhCLE27* was more obvious in *TRV2:GhMCTP7/12/17* plants (Fletcher, 2020; Supplementary Figures 11F,G). Auxin efflux regulator *Gh PIN-FORMED 3/-2* (*GhPIN3/-2*) and *Gh PHOTOSYSTEM I LIGHT HARVESTING COMPLEX GENE2* (*Gh LHCA2*) has been reported to regulate cotton height (Su et al., 2018; Ma et al., 2019). We observed that the expressions of *GhPIN3/-2* and *GhLHCA2* were downregulated in *TRV2:GhMCTP7/12/17* plants, but minor altered in *TRV2:GhKNAT1/2* plants (Supplementary Figures 11H–J). Furthermore, the expression of *GhARR5D*, which functions in the cytokinin signaling pathway, was increased in *TRV2:GhMCTP7/12/17* plants but not in *TRV2:GhKNAT1/2* plants (Supplementary Figure 11L). These results demonstrate that *GhMCTP7*, *GhMCTP12*, and *GhMCTP17* also regulate meristem development independent of *GhKNAT1* and *GhKNAT2*, possible through other *KNOX* family members.

## Discussion

The development of multicellular organisms relies on the coordination of a variety of specialized cell types through intercellular communication. *MCTP* family proteins in *Arabidopsis* and its orthologs in several plant species have been shown to play an important role in protein intercellular movement and are essential for plant development (Liu et al., 2013, 2019; Hao et al., 2020; Zhu et al., 2020). MCTP proteins have been identified in the cotton genome (Hao et al., 2020); however, their biological functions are still largely unknown. In this study, we identified 33 *GhMCTP* genes from the upland cotton genome and analyzed their evolutionary relationships. Through examining the expression patterns of all *GhMCTPs* in different tissues of upland cotton, we found that *GhMCTP7*, *GhMCTP12*, and *GhMCTP17* are highly expressed in the main stem apex and play a key role in shoot development. GhMCTP7/12/17 interacted with GhKNAT1/2 and modulated the expression of multiple shoot meristem regulators in a *GhKNAT1/2*-dependent and independent manner. Our findings suggest that GhMCTP proteins are evolutionarily conserved in upland cotton and play conserved roles in meristem development.

We have systematically characterized 33 *GhMCTP* genes in *G. hirsutum*, which are grouped into seven clades based on the phylogenetic analysis (Figure 1A). The key feature of MCTPs is the presence of multiple C2 domain at the N-terminus and PRT_C domain at the C-terminus (Figure 1C). All MCTPs in *Arabidopsis* contain the C-terminal transmembrane region, which anchors the MCTP in the intracellular membrane. In upland cotton, GhMCTP3 does not contain the C-terminal transmembrane region, and the transmembrane regions of some GhMCTPs are located in the N-terminal C2 domain region (Supplementary Figure 2), suggesting the functional divergence among MCTPs in different species. Comparing with the limited number of MCTPs in animals, a large number of MCTPs are identified in cotton and other plant lineages (Figure 2A), implying that plants have evolved functional specialized MCTPs to regulate distinct biological processes. We also noticed that all GhMCTPs exist in cotton A subgenome and D subgenome, demonstrating their fundamental roles in cotton development. Chromosome distribution of *GhMCTP* shows that *GhMCTP* genes are dispersed across the chromosome but not in a cluster pattern (Supplementary Figure 1), indicating that the *GhMCTP* gene family does not simply arise from chromosome region duplication but also are involved in the extensive reshuffling and divergent evolution. It is also noteworthy that *MCTP* family proteins exist in most plant lineages (Figure 2B), suggesting the fundamental roles of *MCTPs* in plant development. *GhMCTPs* exhibit distinct or overlapping expression patterns in various tissues at different developmental stages (Figure 3), demonstrating *MCTPs* are functionally specialized during plant evolution.

In *Arabidopsis*, different *MCTPs* showed distinct patterns in various tissues, and no *MCTPs* exhibited the identical expression pattern at both vegetative and reproductive tissues (Liu et al., 2018a), suggesting that *MCTPs* might be differentially regulated and play different roles in various tissues. Additionally, some *MCTPs* share similar expression patterns in several tissues (Liu et al., 2018a), indicating that *MCTPs* might function redundantly during plant development. To understand the biological function of *GhMCTPs* in cotton, we examined the expression patterns of all *GhMCTP* in various tissues and revealed their distinct or overlapping expressions in various tissues (Figure 3). *GhMCTP7*, *GhMCTP12*, and *GhMCTP17* are highly expressed in the shoot apex, and their expression levels are correlated with the plant heights in different allotetraploid cotton cultivars. Furthermore, plants with the downregulation of *GhMCTP7*, *GhMCTP12*, and *GhMCTP17* exhibit dwarf phenotype, demonstrating that they might function redundantly to regulate meristem development.

Multiple C2 domain and transmembrane region proteins have been shown to regulate multiple developmental processes by mediating the trafficking of various macromolecules. In *Arabidopsis*, FTIP3 and FTIP4 interact with and regulate STM intercellular and intracellular trafficking, thus affecting the protein distribution within the meristem (Liu et al., 2018b). Regulation of STM trafficking at subcellular and tissue levels causes early termination of shoot apices and continuously generation secondary shoots, resulting in dwarf and bushy phenotypes. The position of leaves and branches, timing of the flowering, and relative position of reproductive structures are traits that affect cotton productivity. Genetic engineering the cotton architecture is crucial for cotton domestication and will benefit crop production. GhMCTP7, GhMCTP12, and GhMCTP17 show high sequence similarity with FTIP3 and FTIP4, hinting that *GhMCTP7*, *GhMCTP12*, and *GhMCTP17* might regulate meristem development through *KNOX* family proteins. Considering the protein interactions between GhMCTP7/12/17 and GhKNAT1/2 (Figures 7, 8), we reasoned that GhMCTP7, GhMCTP12, and GhMCTP17 might regulate the function of GhKNAT1 and GhKNAT2 to modulate meristem development. Consistently, gene silencing of *GhKNAT1* and *GhKNAT2* in *TRV2:GhKNAT1/2* results in a dwarf phenotype (Figure 7), similar to *TRV2:GhMCTP7/12/17*.

KNOTTED1-like homeobox-like transcription factors promote meristem function through regulating various transcription factors and manipulating phytohormone pathways (Jasinski et al., 2005; Bolduc et al., 2012). We examined some of the putative downstream targets of *GhKNAT1* and *GhKNAT2* in the main apex of *TRV2:00*, *TRV2:GhMCTP7/12/17*, and *TRV2:GhKNAT1/2* plants (Figure 9). The expression levels of several *GhKNAT1/2* putative targets are consistently upregulated or downregulated in both *TRV2:GhMCTP7/12/17* and *TRV2:GhKNAT1/2* plants (Figure 9). We also observed that genes with altered expression in *TRV2:GhMCTP7/12/17* are not similarly affected in *TRV2:GhKNAT1/2*, suggesting that *GhMCTP7*, *GhMCTP12*, and *GhMCTP17* also regulate meristem development independent of *GhKNAT1* and *GhKNAT2* (Supplementary Figure 11). Taken together, these results demonstrate that *GhMCTP7*, *GhMCTP12*, and *GhMCTP17* function redundantly to regulate meristem development partially through *GhKNAT1* and *GhKNAT2*.

Plant development requires cell-cell communication and the coordination of various specialized cell types. Non-cell-autonomous signals are one of the regulatory mechanisms to integrate various signals and coordinate plant development. Through the combination of different approaches, an increasing number of mobile signals, such as transcription factors and peptides, have been discovered to regulate plant growth. Severe mutations of key regulators in plants always lead to embryonic lethality or obvious growth defect, which precludes evaluation of later phenotypes and prevents its application for crop breeding (Paaby and Rockman, 2013). Genetic engineering the *cis*-elements of key regulators will bypass its lethality effect and expose multiple pleiotropic roles of this gene (Hendelman et al., 2021). Regulation of protein trafficking will be another alternative approach to make dysfunction of key regulators. MCTP-mediated regulation of proteins at sub-cellular and tissue levels provides a chance to modulate the protein function at the desired degree, which could generate a phenotype that different from the severe mutants and provide important implications for biotechnological application for crop breeding.

## Conclusion

In conclusion, our systematic analysis of *GhMCTPs* in upland cotton illustrates their diverse expression patterns. We find that *GhMCTP7*, *GhMCTP12*, and *GhMCTP17* are highly expressed in the main stem apex, and they function redundantly to regulate the development of the main stem apex and affect cotton architecture. We also show that the expression levels of *GhMCTP7*, *GhMCTP12*, and *GhMCTP17* in the shoot apex of eight selected allotetraploid cotton cultivars are correlated with their plant heights. In addition, VIGS plants silenced for *GhMCTP7*, *GhMCTP12*, and *GhMCTP17* show a dwarf phenotype. Taken together, this study provides important clues for studying the function of *GhMCTPs*, deepens our understanding of cotton apex regulation, and establishes a resource for cotton breeding.

## Data Availability Statement

The original contributions presented in the study are included in the article/**Supplementary Material**, further inquiries can be directed to the corresponding authors.

## Author Contributions

GH, QH, and MZ conceived and designed the experiment and performed most of the experiments. MW, XH, and JL performed some of the experiments and assisted in data analysis. CF analyzed some data. QH, LX, LL, and GH analyzed the data and wrote the manuscript. All authors contributed to the article and approved the submitted version.

## Funding

This work was supported by the National Natural Science Foundation of China (grant no. 31271317), Outstanding Youth Science Fund of Xinjiang Uygur Autonomous Region (grant no. 2021D01E17), Fundamental Research Funds for the Central Universities (grant nos. CCNU16A02047 and CCNU19TS064), and the Shanghai Pujiang Program (20PJ1405200).

## Conflict of Interest

The authors declare that the research was conducted in the absence of any commercial or financial relationships that could be construed as a potential conflict of interest.

## Publisher’s Note

All claims expressed in this article are solely those of the authors and do not necessarily represent those of their affiliated organizations, or those of the publisher, the editors and the reviewers. Any product that may be evaluated in this article, or claim that may be made by its manufacturer, is not guaranteed or endorsed by the publisher.

## Footnotes

^1^ https://www.cottongen.org/blast/protein/protein

^2^ http://myhits.isb-sib.ch/cgi-bin/motif_scan

^3^ http://www.ebi.ac.uk/Tools/pfa/iprscan

^4^ http://smart.emblheidelberg.de/

^5^ https://www.cottongen.org/find/genes

^6^ http://us.expasy.org/tools/protparam.html

^7^ https://www.cottongen.org/

^8^ https://phytozome.jgi.doe.gov/pz/portal.html

^9^ https://www.megasoftware.net

^10^ http://gsds.cbi.pku.edu.cn/

^11^ https://www.cottongen.org/species/Gossypium_hirsutum/ZJU-AD1_v2.1

^12^ http://structuralbiology.cau.edu.cn/gossypium

^13^ https://github.com/CJ-Chen/TBtools
