# Supplementary material for: Family-Wide Evaluation of Multiple C2 Domain and Transmembrane Region Protein in Gossypium hirsutum
Source: Front Plant Sci. 2021 Oct 25;12:767667. doi: 10.3389/fpls.2021.767667 (PMC8573151; doi:10.3389/fpls.2021.767667)
Supplement: Supplementary file 1 [file Data_Sheet_1.docx]

**Family-wide Evaluation of Multiple C2 Domain and Transmembrane Region Protein in *Gossypium hirsutum***

Qianqian Hu^1＃^, Mengting Zeng^1＃^, Miao Wang^1^, Xiaoyu Huang^1^, Jiayi Li^3^, Changhui Feng^2^, Lijie Xuan^3^, Lu Liu^3^*, Gengqing Huang^1,4^*

^＃^These authors contributed equally to this manuscript

^1^ Hubei Key Laboratory of Genetic Regulation and Integrative Biology, School of Life Sciences, Central China Normal University, Wuhan, Hubei, China

^2^ Institute of Cash Crops, Hubei Academy of Agricultural Sciences, Wuhan, Hubei, China

^3^ Joint Center for Single Cell Biology, School of Agriculture & Biology, Shanghai Jiao Tong University, Shanghai, China

^4^ Xinjiang Key Laboratory of Special Species Conservation and Regulatory Biology, College of Life Science, Xinjiang Normal University, Urumuqi, Xinjiang Autonomous Region, China

***Corresponding author:**

Gengqing Huang

Tel: +86-13476151985

Email: gqhuang@mail.ccnu.edu.cn

Lu Liu

Tel: +86-021-34208699

Email: lu.liu@sjtu.edu.cn


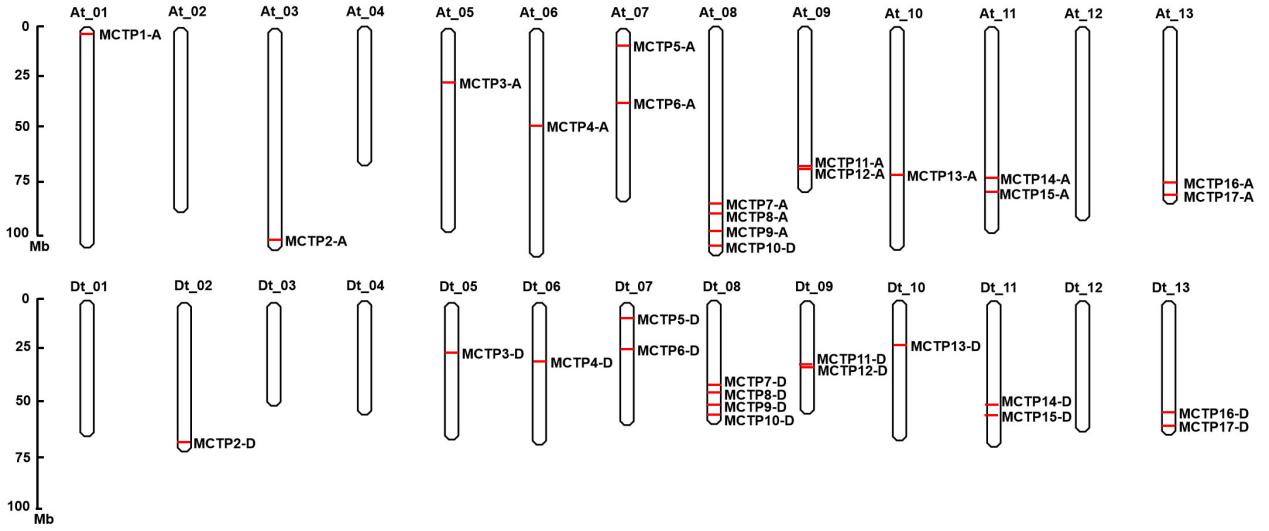


**Supplementary Figure 1 |** Chromosome distribution of *GhMCTP* genes. *GhMCTP-A* and *GhMCTP-D* are derived from the A-subgenome and D-subgenome progenitor in the tetraploid cotton.


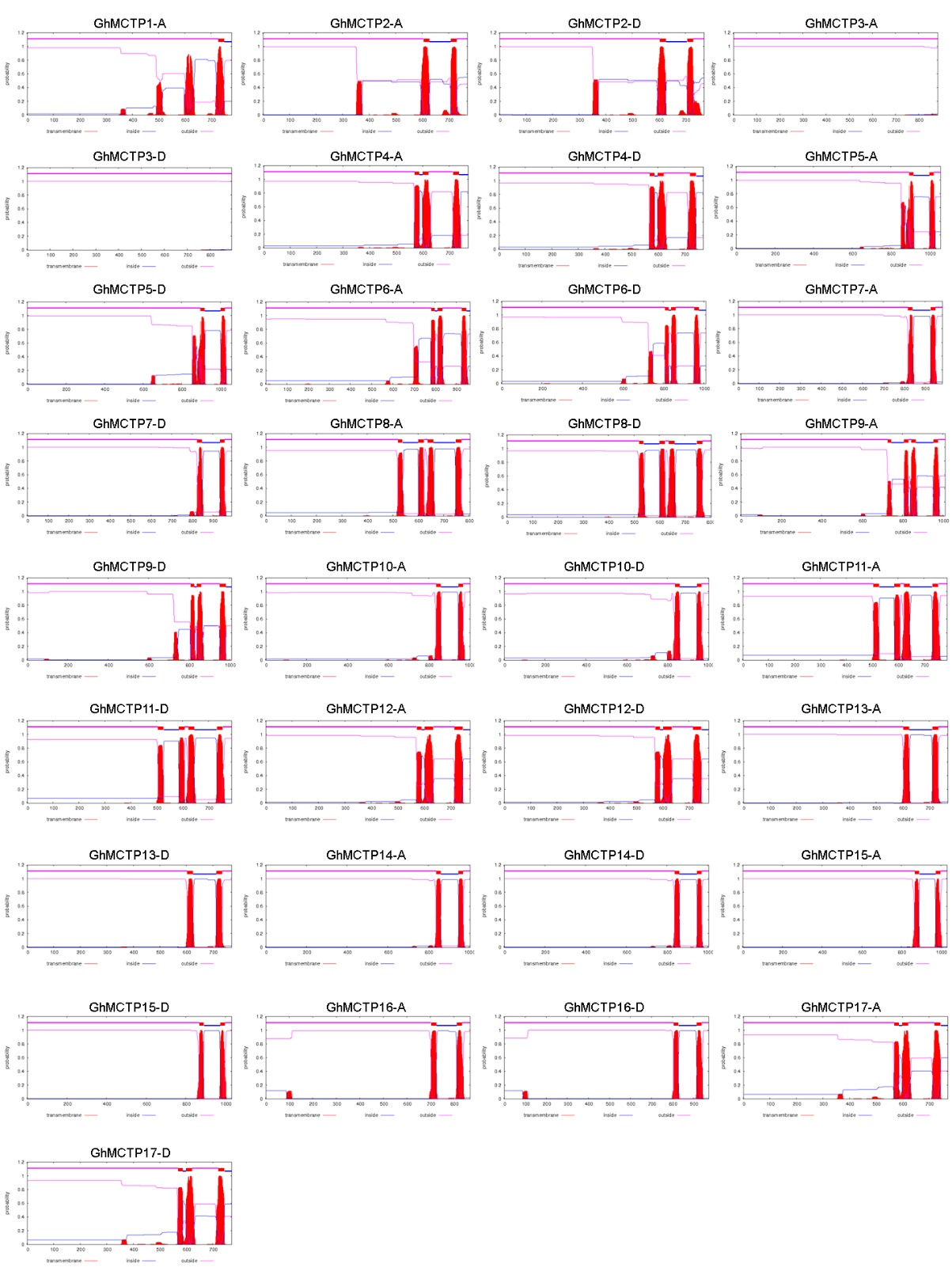


**Supplementary Figure 2 |** Topology prediction of transmembrane helices of 33 GhMCTPs in cotton. Topology prediction was performed using TMHMM program (http://www.cbs.dtu.dk/services/TMHMM/)*.* Red columns indicate the transmembrane regions of MCTP proteins.


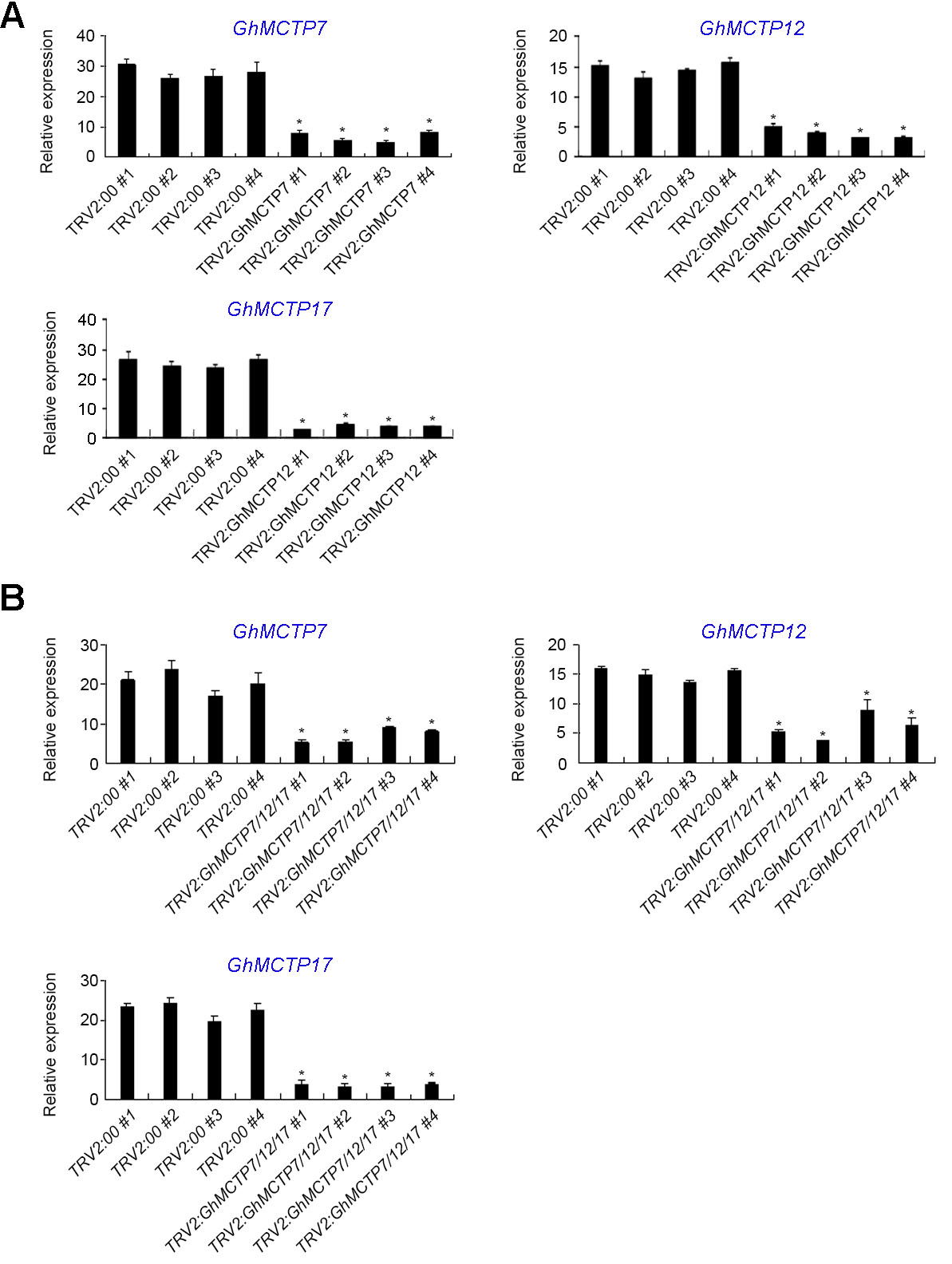


**Supplementary Figure 3 |** Expression analysis of *GhMCTP7*, *GhMCTP12*, and *GhMCTP17*. **(A)** Quantitative analysis of *GhMCTP7*, *GhMCTP12*, and *GhMCTP17* expression in main stem apex of representative *TRV2:00*, *TRV2:GhMCTP7*, *TRV2:GhMCTP12*, and *TRV2:GhMCTP17* plants. **(B)** Quantitative analysis of *GhMCTP7*, *GhMCTP12*, and *GhMCTP17* expression in main stem apex of representative *GhMCTP7/12/17* plants. Results were normalized against the expression level of *GhUBI1*. Error bars represent SD of three biological replicates (*P < 0.01).


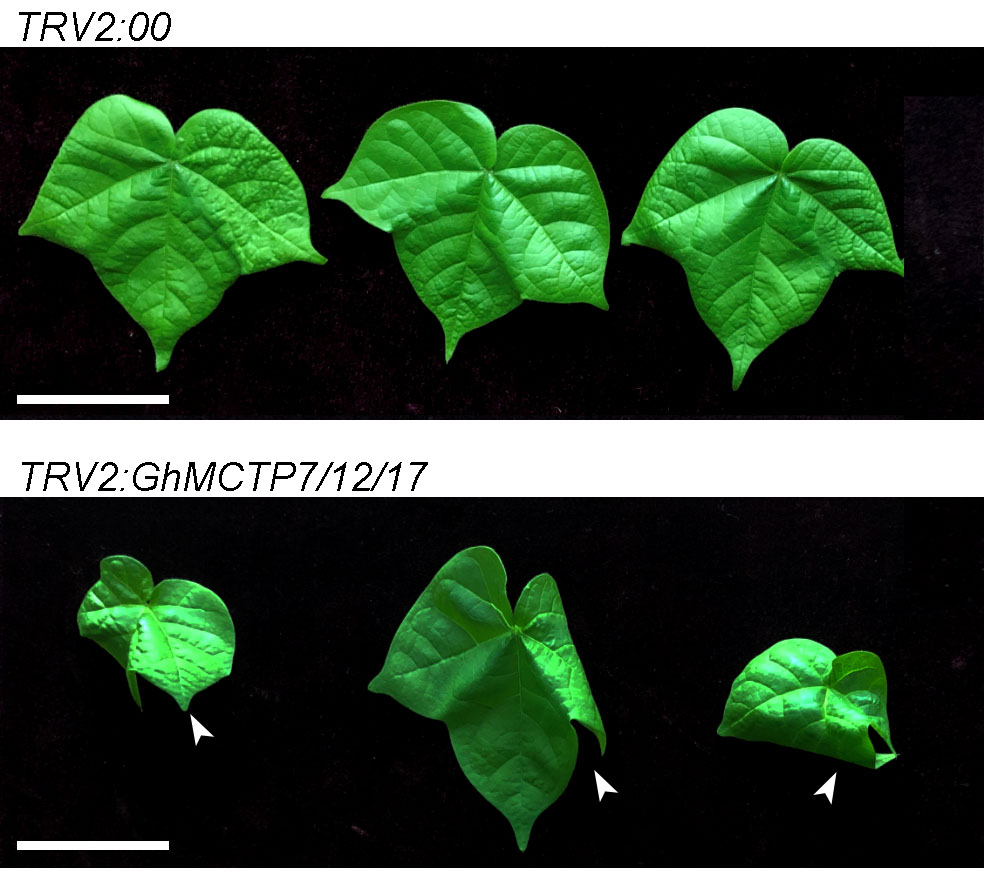


**Supplementary Figure 4 |** *GhMCTP7*, *GhMCTP12*, and *GhMCTP17* regulate leaf development. *TRV2:GhMCTP7/12/17* plants have smaller and curling leaf (white arrowhead indicated) compared with *TRV2:00* plants. Scale bars = 3 cm.


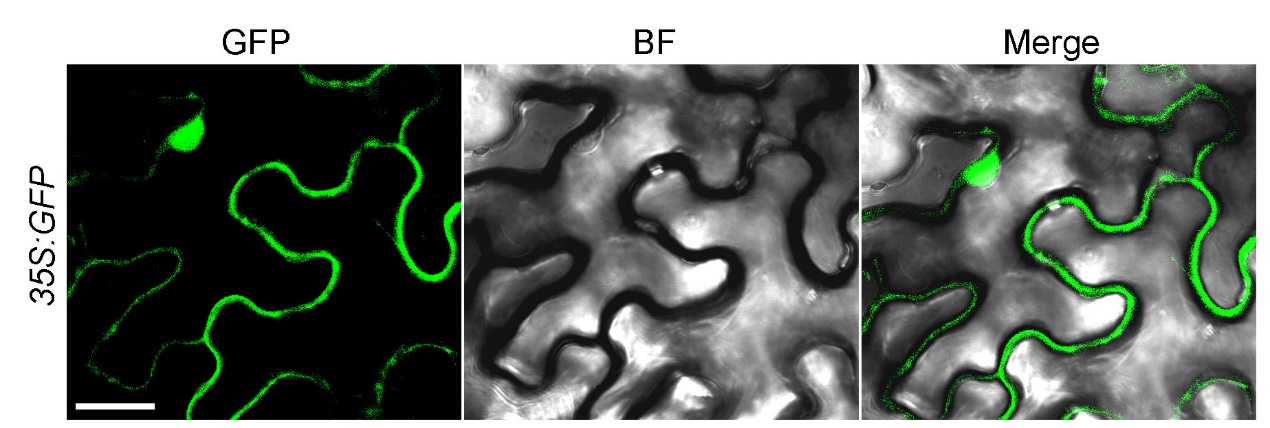


**Supplementary Figure 5 |** Subcellular localization of free GFP in *N. benthamiana* leaf epidermal cells. Scale bars = 20 µm.


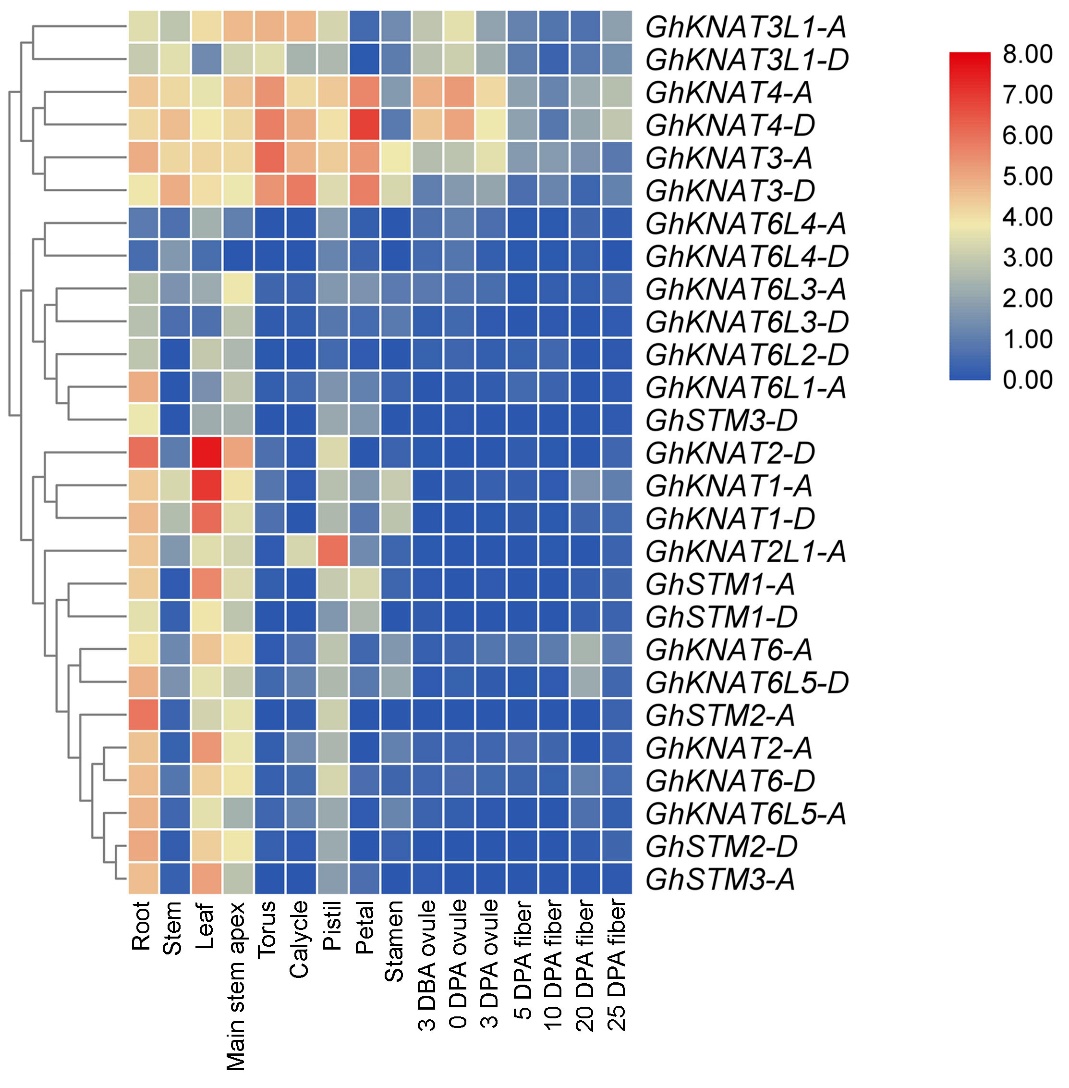


**Supplementary Figure 6 |** Heat map analysis of *GhKNAT* genes in different organs in upland cotton. The relative fold change in the gene expression of KNOX family genes was compared. The color from blue to red indicates low to high expression. DBA, days before-anthesis; DPA, days post-anthesis.


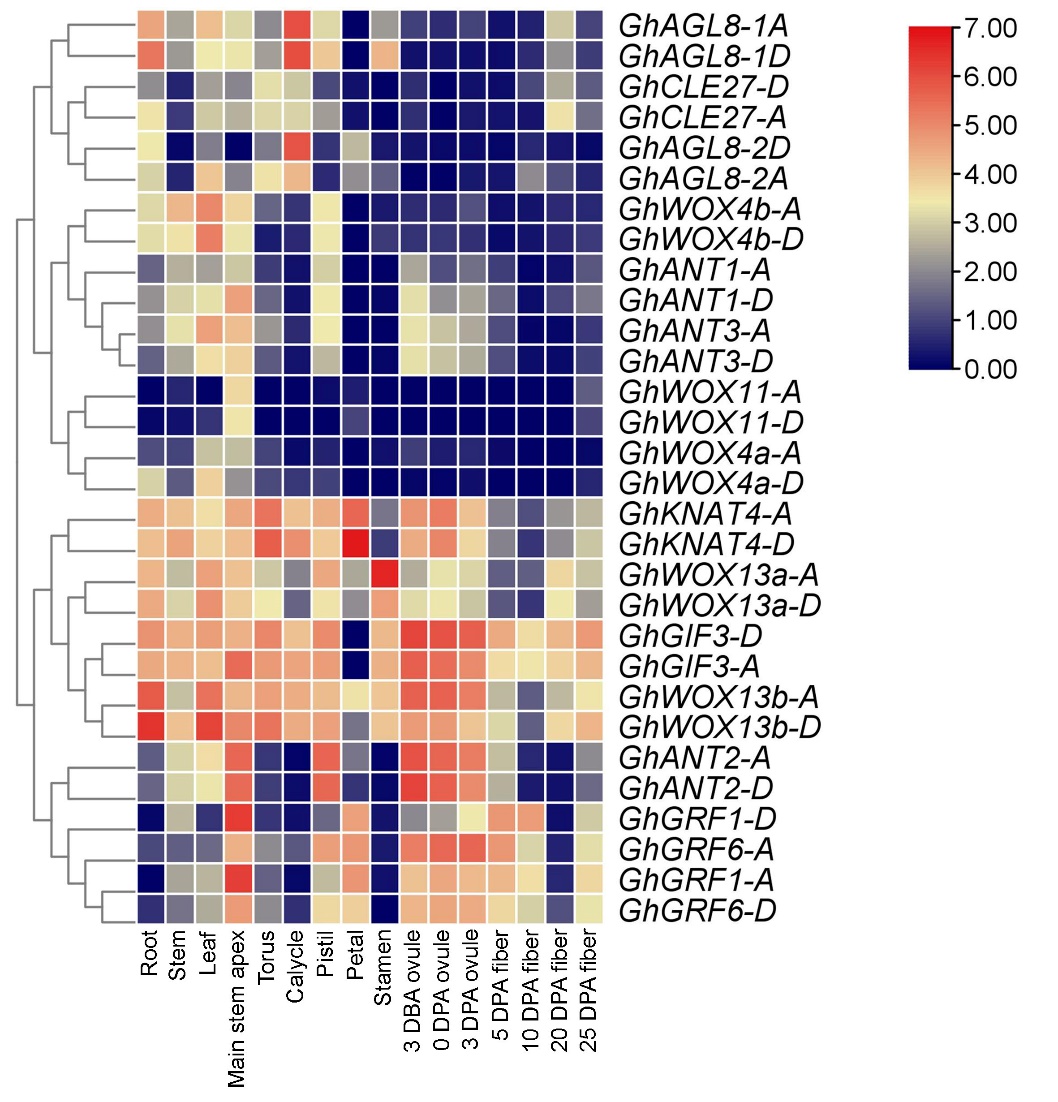


**Supplementary Figure 7 |** Heat map of some putative shoot apical meristem regulators in different organs in upland cotton. The relative fold change in the gene expression was compared. The color from blue to red indicates low to high expression. DBA, days before-anthesis; DPA, days post-anthesis.


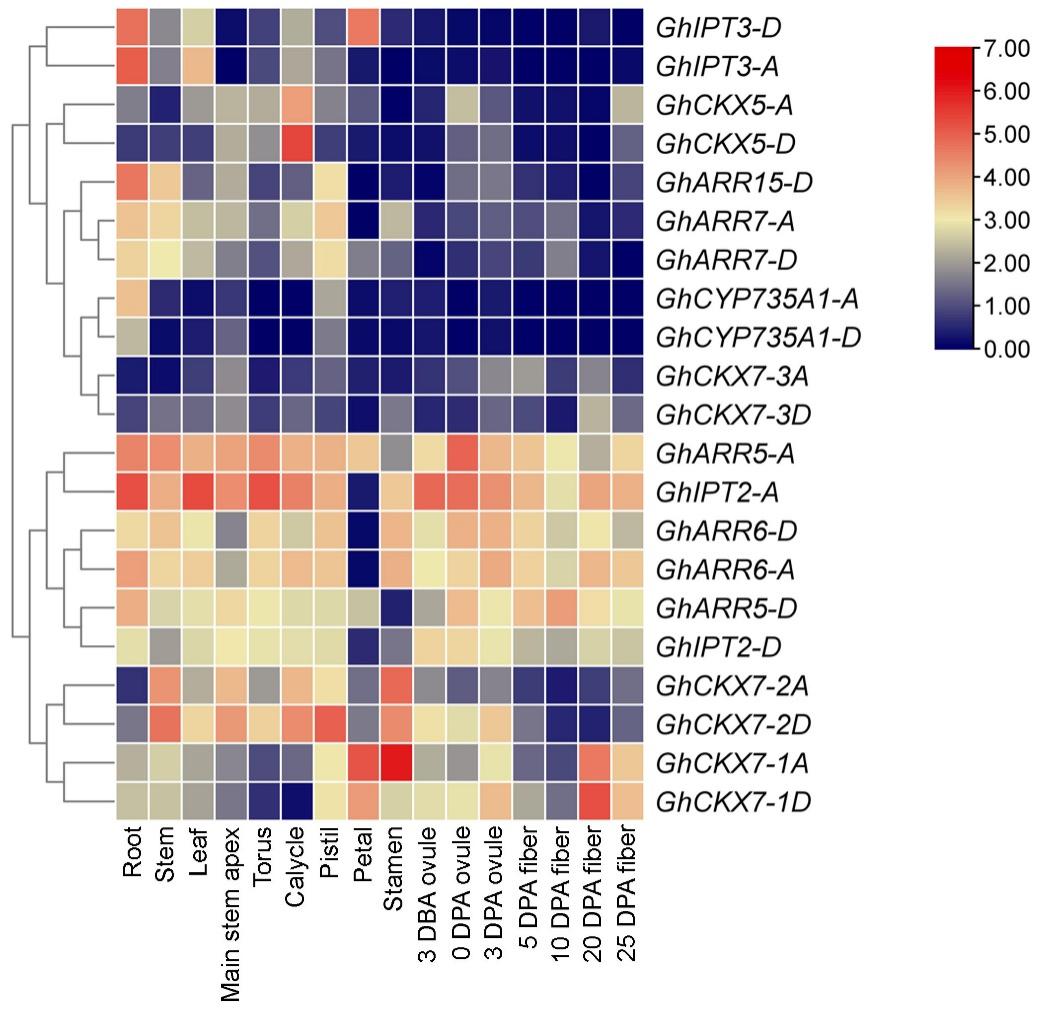


**Supplementary Figure 8 |** Heat map of cytokinin biosynthesis and signaling regulators in different organs in upland cotton. The relative fold change in the gene expression was compared. The color from blue to red indicates low to high expression. DBA, days before-anthesis; DPA, days post-anthesis.


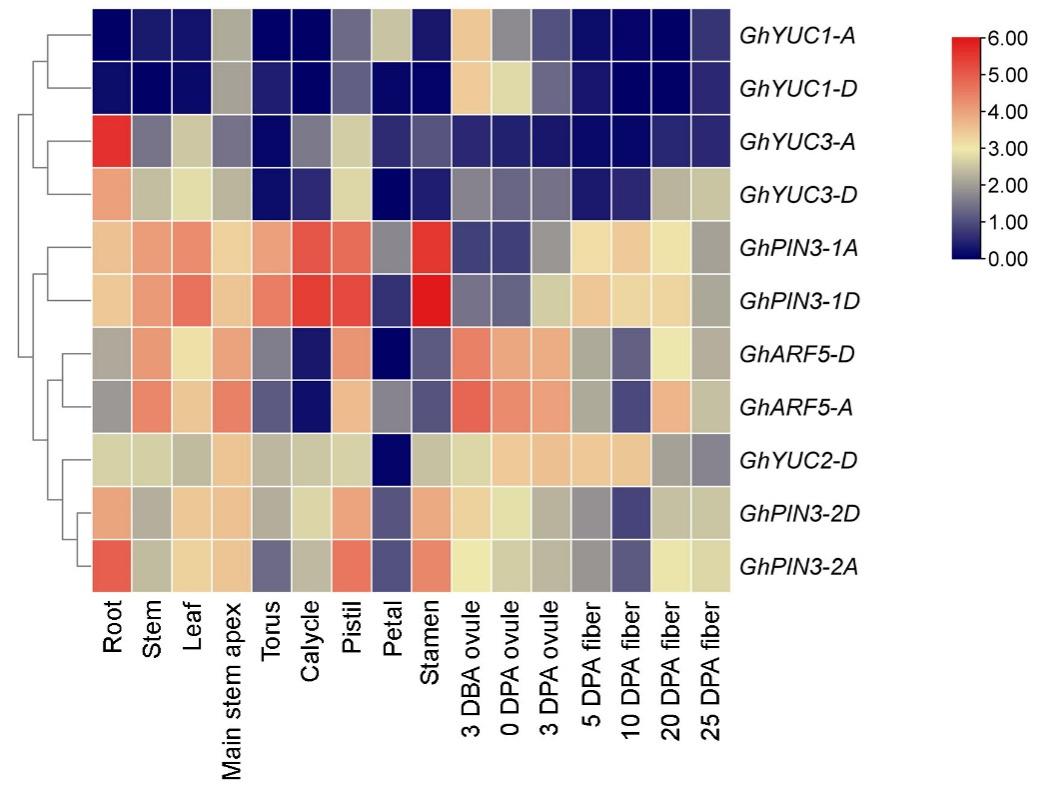


**Supplementary Figure 9 |** Heat map of auxin transport, biosynthesis, and signaling regulators in different organs in upland cotton. The relative fold change in the gene expression was compared. The color from blue to red indicates low to high expression. DBA, days before-anthesis; DPA, days post-anthesis.


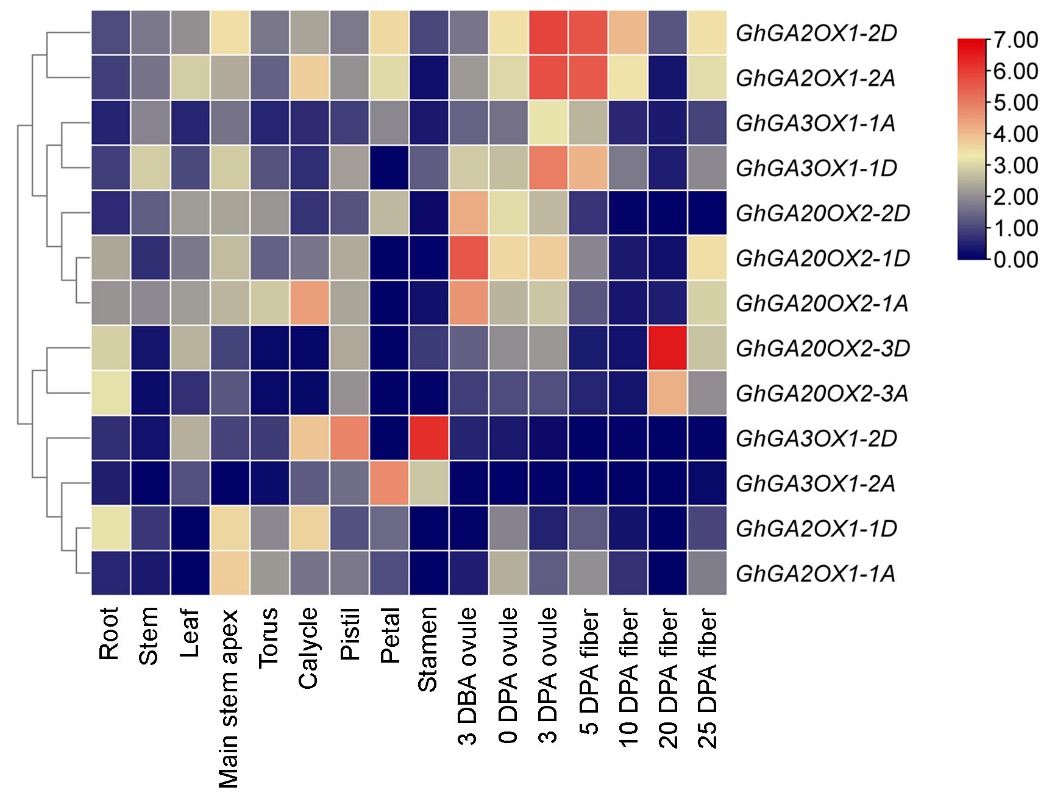


**Supplementary Figure 10 |** Heat map of Gibberellin (GA) biosynthesis and catabolism regulators in different organs in upland cotton. The relative fold change in the gene expression was compared. The color from blue to red indicates low to high expression. DBA, days before-anthesis; DPA, days post-anthesis.


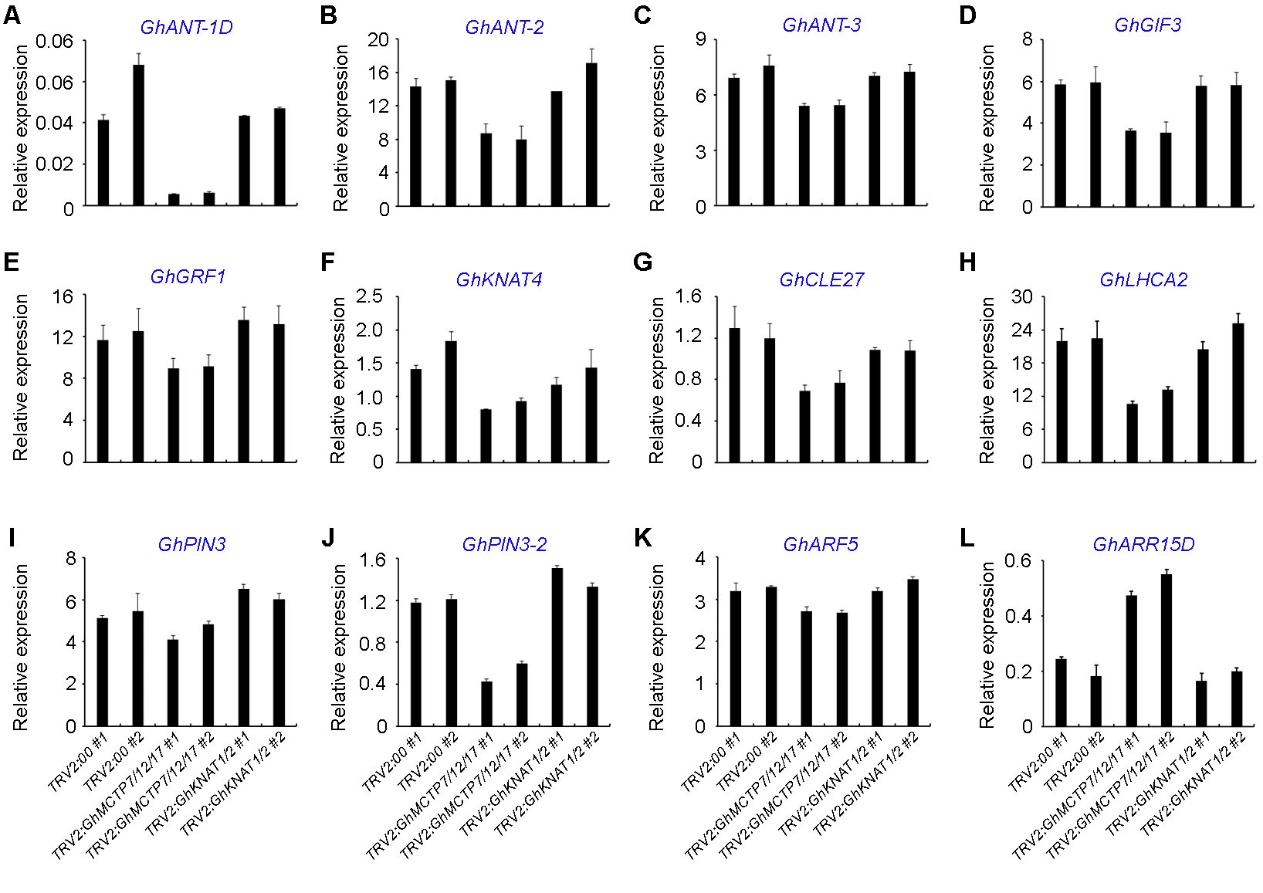


**Supplementary Figure 11 |** Quantitative analysis of several shoot meristem regulators in main stem apex of *TRV2:00*, *TRV2:GhMCTP7/12/17*, and *TRV2:GhKNAT1/2* plants. Quantitative analysis of the expression levels of *GhANT-1D* **(A)**, *GhANT-2* **(B)**, *GhANT-3* **(C)**, *GhGIF3* **(D)**, *GhGRF1* **(E)**, *GhKNAT4* **(F)**, *GhCLE27* **(G)**, *GhLHCA2* **(H)**, *GhPIN3* **(I)**, *GhPIN3-2* **(J)**, *GhARF5* **(K)**, and *GhARR15D* **(L)** in *TRV2:00*, *TRV2:GhMCTP7/12/17* and *TRV2:GhKNAT1/2* plants. Results were normalized against the expression level of *GhUBI1*. Error bars indicate SD of three biological replicates.

**Supplementary Table 1 |** *MCTP* gene family in cotton (*Gossypium hirsutum L. acc.* TM-1, *G. arboreum*, and *G. raimondii*) ^a^

| **Gene name ^b^** | **Gene symbol** | **Chromosome and Location** | **Length**  **(a.a.)** | **MW**  **(KD)** | **pI** | **Orthologs and ID in *G. arboreum* (Length a.a.)** | **Orthologs and ID in *G. raimondii* (Length a.a.)** | **Homologs in *Arabidopsis*** | **E-value for BlastP** |
| --- | --- | --- | --- | --- | --- | --- | --- | --- | --- |
| GhMCTP1-A | GH_A01G0096 | A01:759545 to 757209 | 778 | 89.2 | 7.22 | GaMCTP1 Gar01G00960 (778) | GrMCTP1a Gorai.002G010600.1 (776) | AT3G61720 (AtMCTP12) / AT5G03435 (AtMCTP13) | 0.0 |
|  |  |  |  |  |  |  | GrMCTP1b Gorai.002G116500.1 (768) | AT3G61720 (AtMCTP12) / AT5G03435 (AtMCTP13) | 0.0 |
| GhMCTP2-A | GH_A03G2424 | A03:111400941 to 111403256 | 771 | 86.8 | 7.68 | GaMCTP2 Gar03G00370 (771) | GrMCTP2 Gorai.005G266200.1 (771) | AT3G61720 (AtMCTP12) / AT5G03435 (AtMCTP13) | 0.0 |
| GhMCTP2-D | GH_D02G2586 | D02:69561109 to 69563421 | 770 | 86.8 | 8.11 |  |  |  | 0.0 |
| GhMCTP3-A | GH_A05G2753 | A05:30354179 to 30357259 | 1044 | 120.1 | 8.72 | GaMCTP3 Gar05G29440 (1046aa) | GrMCTP3 Gorai.009G288400.1 (1046) | AT1G22610 (AtMCTP6) | 0.0 |
| GhMCTP3-D | GH_D05G2770 | D05:26242555 to 26245695 | 1046 | 120.3 | 8.80 |  |  |  | 0.0 |
| GhMCTP4-A | GH_A06G1275 | A06:70018717 to 70016400 | 772 | 88.1 | 9.05 | GaMCTP4 Gar06G14310 (775) | GrMCTP4 Gorai.010G128400.1 (772) | AT5G12970 (AtMCTP5) | 0.0 |
| GhMCTP4-D | GH_D06G1235 | D06: 28819923.. 28822241 | 772 | 88.1 | 9.05 |  |  |  | 0.0 |
| GhMCTP5-A | GH_A07G0726 | A07:8566280 to 8569453 | 1057 | 118.9 | 7.02 | GaMCTP5 Gar07G07780 (1063) | GrMCTP5 Gorai.001G073200.1 (1059) | AT1G74720 (AtMCTP15 / QKY) | 0.0 |
| GhMCTP5-D | GH_D07G0717 | D07:7557848 to 7561027 | 1059 | 119.0 | 6.92 |  |  |  | 0.0 |
| GhMCTP6-A | GH_A07G1673 | A07:40252190 to 40249156 | 966 | 108.4 | 9.51 | GaMCTP6 Gar07G19450 (1012) | GrMCTP6 Gorai.001G183000.1(1012) | AT1G04150 (AtMCTP10) | 0.0 |
| GhMCTP6-D | GH_D07G1672 | D07:27948190 to 27945155 | 1011 | 113.7 | 9.28 |  |  |  | 0.0 |
| GhMCTP7-A | GH_A08G1568 | A08:102899407 to 102902364 | 985 | 111.7 | 9.17 | GaMCTP7 Gar08G17690 (985) | GrMCTP7 Gorai.004G157900.1 (991) | AT3G61300 (AtMCTP8) / AT4G00700 (AtMCTP9) | 0.0 |
| GhMCTP7-D | GH_D08G1587 | D08:51331809 to 51334784 | 991 | 112.4 | 9.21 |  |  |  | 0.0 |
| GhMCTP8-A | GH_A08G1725 | A08:107192850 to 107190433 | 805 | 92.5 | 9.01 | GaMCTP8 Gar08G19560 (801) | GrMCTP8 Gorai.004G174500.1 (805) | AT5G48060 (AtMCTP2) | 0.0 |
| GhMCTP8-D | GH_D08G1741 | D08:54365516 to 54363099 | 805 | 92.7 | 9.08 |  |  |  | 0.0 |
| GhMCTP9-A | GH_A08G2079 | A08:116463125 to 116460090 | 1011 | 115.0 | 9.06 | GaMCTP9 Gar08G23700 (1011) | GrMCTP9 Gorai.004G210900.1 (1011) | AT4G11610 (AtMCTP7) | 0.0 |
| GhMCTP9-D | GH_D08G2089 | D08:60994015 to 60990983 | 1010 | 115.0 | 9.11 |  |  |  | 0.0 |
| GhMCTP10-A | GH_A08G2479 | A08:121564816 to 121561805 | 1003 | 112.8 | 6.94 | GaMCTP10 Gar08G28110(1001) | GrMCTP10 Gorai.004G252500.1 (1003) | AT3G03680 (AtMCTP14) | 0.0 |
| GhMCTP10-D | GH_D08G2487 | D08:65691104 to 65688093 | 1003 | 112.7 | 7.08 |  |  |  | 0.0 |
| GhMCTP11-A | GH_A09G1426 | A09:69891042 to 69893411 | 789 | 90.9 | 9.13 | GaMCTP11 Gar09G16260 (789) | GrMCTP11 Gorai.006G140900.1 (789) | AT5G06850 (AtMCTP1/AtFTIP1) | 0.0 |
| GhMCTP11-D | GH_D09G1373 | D09:40335945 to 40338314 | 789 | 91.1 | 9.12 |  |  |  | 0.0 |
| GhMCTP12-A | GH_A09G1428 | A09:69961239 to 69958912 | 775 | 88.9 | 9.10 | GaMCTP12 Gar09G16300(775) | GrMCTP12 Gorai.006G141200.1 (775) | AT3G57880 (AtMCTP3) / AT1G51570 (AtMCTP4) | 0.0 |
| GhMCTP12-D | GH_D09G1375 | D09:40388189 to 40385862 | 775 | 88.9 | 9.10 |  |  |  | 0.0 |
| GhMCTP13-A | GH_A10G1491 | A10:77149258 to 77151570 | 770 | 88.0 | 8.11 | GaMCTP13 Gar10G15220 (770) | GrMCTP13 Gorai.011G142300.1 (771) | AT4G20080 (AtMCTP11) | 0.0 |
| GhMCTP13-D | Gh_D10G1266 | D10:22449216 to 22446901 | 771 | 88.1 | 8.85 |  |  |  | 0.0 |
| GhMCTP14-A | GH_A11G0906 | A11:8024248 to 8027265 | 1031 | 115.2 | 7.14 | GaMCTP14 Gar11G28440(1031) | GrMCTP14 Gorai.007G259900.1 (1031) | AT5G17980 (AtMCTP16) | 0.0 |
| GhMCTP14-D | GH_D11G0941 | D11:7683857 to 7686874 | 1031 | 115.4 | 6.80 |  |  |  | 0.0 |
| GhMCTP15-A | GH_A11G2576 | A11:93937265 to 93940360 | 1005 | 114.9 | 9.07 | GaMCTP15 Gar11G09630 (1005) | GrMCTP15 Gorai.007G098200.1 (1005) | AT3G61300 (AtMCTP8) / AT4G00700 (AtMCTP9) | 0.0 |
| GhMCTP15-D | GH_D11G2630 | D11:52216105 to 52219200 | 1005 | 114.9 | 9.03 |  |  |  | 0.0 |
| GhMCTP16-A | GH_A13G1887 | A13:100933209 to 100936126 | 869 | 97.7 | 5.58 | GaMCTP16 Gar13G21930 (972) | GrMCTP16 Gorai.013G196200.1 (972) | AT3G03680 (AtMCTP14 ) | 0.0 |
| GhMCTP16-D | GH_D13G1840 | D13:55729929 to 55732847 | 972 | 109.3 | 6.11 |  |  |  | 0.0 |
| GhMCTP17-A | GH_A13G2434 | A13:108688936 to 108691251 | 771 | 88.2 | 9.08 | GaMCTP17 Gar13G28530 (771) | GrMCTP17 Gorai.013G250100.1 (771) | AT5G12970 (AtMCTP5) | 0.0 |
| GhMCTP17-D | GH_D13G2425 | D13:62538928 to 62541243 | 771 | 88.2 | 9.18 |  |  |  | 0.0 |

^a^ Genes information in G. raimondii from Paterson et al. 2012, *G. arboreum* from Huang et al. 2020 and *G. hirsutum* from Hu et al. 2019.

^b^ A and D were derived from the A-genome and D-genome progenitor in the tetraploid cotton.

Reference:

Reference:

Hu, Y., Chen, J., Fang, L., Zhang, Z., Ma, W., Niu, Y., et al. (2019). *Gossypium barbadense* and *Gossypium hirsutum* genomes provide insights into the origin and evolution of allotetraploid cotton. Nat Genet. 51, 739-748. doi: 10.1038/s41588-019-0371-5.

Huang, G., Wu, Z., Percy, R.G., Bai, M., Li, Y., Frelichowski, J.E., Hu, J., Wang, K., Yu, J.Z., Zhu, Y. (2020). Genome sequence of *Gossypium herbaceum* and genome updates of *Gossypium arboreum* and *Gossypium hirsutum* provide insights into cotton A-genome evolution. Nat Genet. 52, 516-524. doi: 10.1038/s41588-020-0607-4.

**Supplementary Table 2 |** List of primers of this study used for different experiments.

| Gene name | Primers (5’-3’) | Purpose |
| --- | --- | --- |
| GhMCTP1 RT–F | CGACTCTTGCAACGAACAAGAT | qRT-PCR |
| GhMCTP1 RT–R | TGTACTCCACAACTTCTTGCCT | qRT-PCR |
| GhMCTP2 RT–F | GAGGTATGATCGATTGCGAAGC | qRT-PCR |
| GhMCTP2 RT–R | GTGGGCAATCTTCCGAACACGT | qRT-PCR |
| GhMCTP3 RT–F | ACATGGTGGCTCAATACGGTAG | qRT-PCR |
| GhMCTP3 RT–R | ACCACTTGCAAATAGCAGTTAC | qRT-PCR |
| GhMCTP4 RT–F | TCAGACTGTGATTGGTGATCTG | qRT-PCR |
| GhMCTP4 RT–R | AACATGCTGTCCGATCTCGCAG | qRT-PCR |
| GhMCTP5 RT–F | CAAGATACCAGCAGGCATGGAT | qRT-PCR |
| GhMCTP5 RT–R | TCTTGGGTCCCTCCAACTCACT | qRT-PCR |
| GhMCTP6 RT–F | GTATGATCGACTAAGGAGTGTG | qRT-PCR |
| GhMCTP6 RT–R | CAAGGCAACTTGCTTCGGAATC | qRT-PCR |
| GhMCTP7 RT–F | CGAATTCAGACTGTTGTTGGTG | qRT-PCR |
| GhMCTP7 RT–R | GATCGGTGCCGAAGGTGTCTTG | qRT-PCR |
| GhMCTP8 RT–F | CATGTTCCTAGTGGGACTATG | qRT-PCR |
| GhMCTP8 RT–R | CAGCAGCGACTCAAATCTCTC | qRT-PCR |
| GhMCTP9 RT–F | GGTTTCACAAGCTGAGGCAGT | qRT-PCR |
| GhMCTP9 RT–R | CACTATGGCTGCTATAAGGCAG | qRT-PCR |
| GhMCTP10 RT–F | CACAGTACGGTTCCGATACGAC | qRT-PCR |
| GhMCTP10 RT–R | GTGTCGGATGTAGTAGAACCCT | qRT-PCR |
| GhMCTP11 RT–F | GACGAACTCGATGAAGAGTTTG | qRT-PCR |
| GhMCTP11 RT–R | TCACATACAGTGCCACTGCTG | qRT-PCR |
| GhMCTP12 RT–F | ACACAGGGTGAGAGGCTGCAAT | qRT-PCR |
| GhMCTP12 RT–R | CATAGCATGGAATCAGTTCTTG | qRT-PCR |
| GhMCTP13 RT–F | GAGGTATGATCGGCTGAGAAGC | qRT-PCR |
| GhMCTP13 RT–R | GCATTGAAGGAATGCTGATTCG | qRT-PCR |
| GhMCTP14 RT–F | GGGTATGGAAGTATCGTTATCG | qRT-PCR |
| GhMCTP14 RT–R | GGCTGCTGCAAGACAGAATATC | qRT-PCR |
| GhMCTP15 RT–F | GCACGAGGTATGATAAGTTACG | qRT-PCR |
| GhMCTP15 RT–R | AAGTTCAGTGCCGGTGACGGCA | qRT-PCR |
| GhMCTP16 RT–F | AGTCCCGATGAGCTGGATGAAG | qRT-PCR |
| GhMCTP16 RT–R | ATCCTGCGCCTAACACTAACAC | qRT-PCR |
| GhMCTP17 RT–F | TCCTTTATCCCGAGCTGATACT | qRT-PCR |
| GhMCTP17 RT–R | TTGCGAGGTCACCGATCACAGT | qRT-PCR |
| GhUBI1 RT-F | GGGATGCAAATCTTCGTGAAAAC | qRT-PCR |
| GhUBI1 RT-R | CTGAATCTTCGCTTTCACGTTATC | qRT-PCR |
| GhKNAT1 RT-F | TGGGAGTTACACTACAAATGG | qRT-PCR |
| GhKNAT1 RT-R | TCATGGGCCCAAACGGTAAGG | qRT-PCR |
| GhKNAT2 RT-F | TGGCCGTACCCGTCGGAGACT | qRT-PCR |
| GhKNAT2 RT-R | TCATGGGCCCAAACGGTAAGG | qRT-PCR |
| GhKNAT3 RT-F | GAACTCAAACAGGGTTACAAAG | qRT-PCR |
| GhKNAT3 RT-R | CTTCCTTTGATTGATGAACCA | qRT-PCR |
| GhKNAT4 RT-F | CAATTACAACAACATGTTCGTG | qRT-PCR |
| GhKNAT4 RT-R | CTCTCTGTTGGGATCAAAGGTC | qRT-PCR |
| GhKNAT6 RT-F | ACTACCAAGAGAAGCACGACA | qRT-PCR |
| GhKNAT6 RT-R | TCAGTCCTCTGAAAAGAATTG | qRT-PCR |
| GhANT-1D RT-F | CTACTCACTTCTCCAATGCTTC | qRT-PCR |
| GhANT-1D RT-R | ACTGCCTGGACAGATGCTTAG | qRT-PCR |
| GhANT-2 RT-F | GAATCGTGTAAGCTAGGGACT | qRT-PCR |
| GhANT-2 RT-R | GTGAGCCATTGAAATGGCAGT | qRT-PCR |
| GhANT-3 RT-F | GTTGACTAGTTTGAGCAGCTC | qRT-PCR |
| GhANT-3 RT-R | CTAAGCATCTGTCCAGGCAGT | qRT-PCR |
| GhAGL8-1 RT-F | TCATCAGATTCTTGCATGGAG | qRT-PCR |
| GhAGL8-1 RT-R | GTGCTCCAAGTTCTGAAGCT | qRT-PCR |
| GhAGL8-2 RT-F | GCGACTGAGTCTTGCATGGA | qRT-PCR |
| GhAGL8-2 RT-R | TTGCTCCAAATTTTGAAGCTC | qRT-PCR |
| GhYUC1 RT-F | CACATGGTAGTTAGAAACACA | qRT-PCR |
| GhYUC1 RT-R | GACGTTCTTGAGCTCGATAG | qRT-PCR |
| GhYUC2D RT-F | GTTCGATGTGGACGTCGAAG | qRT-PCR |
| GhYUC2D RT-R | CCTTGGAGCTGCTTCAGTAC | qRT-PCR |
| GhYUC3 RT-F | GGAGATGTTCTTCATTCAAC | qRT-PCR |
| GhYUC3 RT-R | GGTATATACTTCAACAGATTC | qRT-PCR |
| GhARR5 RT-F | AGCATCTGAGTCTCAATTGCA | qRT-PCR |
| GhARR5 RT-R | AAGATTCACCTTCAAACCATTG | qRT-PCR |
| GhARR6 RT-F | AGATTACACGACCACAGAGTTA | qRT-PCR |
| GhARR6 RT-R | GAATCCAGTGTTGAAGGAGAC | qRT-PCR |
| GhARR7 RT-F | CAACAGATGCTTGGAAGATGG | qRT-PCR |
| GhARR7 RT-R | TCAGACAACTTCCAATTCATTG | qRT-PCR |
| GhARR15D RT-F | CTGGAACCAAGGCATTGGAG | qRT-PCR |
| GhARR15D RT-R | TCCGAAGACATGATGACAACT | qRT-PCR |
| GhGRF1 RT-F | GTACTAACAGCAGCACAGGTG | qRT-PCR |
| GhGRF1 RT-R | CTGAACCAAGGAGGTTATTGC | qRT-PCR |
| GhGRF6 RT-F | GAGTCTCTTCATTTCCTCAGT | qRT-PCR |
| GhGRF6 RT-R | AATGAGGTCTGGTTGGATCTG | qRT-PCR |
| GhGIF3 RT-F | TCAACAAGCAATGCAAGGTC | qRT-PCR |
| GhGIF3 RT-R | AGTTGTGCTGCCCTGCCCA | qRT-PCR |
| GhARF5 RT-F | CATGCTGCTGCCAATAGAAG | qRT-PCR |
| GhARF5 RT-R | TCCAAGTCGCTAATACCAACT | qRT-PCR |
| GhLHCA2 RT-F | ACTCTCTTCATCGTTGAGCT | qRT-PCR |
| GhLHCA2 RT-R | CAGCTCCTTGATCTTCTCAG | qRT-PCR |
| GhCLE27 RT-F | CTTCTATGGTGGTGATGTTG | qRT-PCR |
| GhCLE27 RT-R | CTCTTCGTTTGCTATCTTCA | qRT-PCR |
| GhPIN3-1 RT-F | GCTTAATCTTGATCATGGTCT | qRT-PCR |
| GhPIN3-1 RT-R | CACAAGCGATGATCTTGGGT | qRT-PCR |
| GhPIN3-2 RT-F | CTTGCAGGTCCAGCTGTGAT | qRT-PCR |
| GhPIN3-2 RT-R | AGACAAGCGTTATGGGCAAG | qRT-PCR |
| GhIPT2 RT-F | GCGACAGGCCATAGGTGTTC | qRT-PCR |
| GhIPT2 RT-R | GTCAATGGCTTCTTCTAACA | qRT-PCR |
| GhIPT3 RT-F | CTGCTGCAAGAAGCAATACA | qRT-PCR |
| GhIPT3 RT-R | CACTATCTCCGTACTAGGAC | qRT-PCR |
| GhWOX4-1 RT-F | ACGGGAGAGACAAAAGCAGAAG | qRT-PCR |
| GhWOX4-1 RT-R | GTGCAATGGGAAGAGCTCCAGA | qRT-PCR |
| GhWOX4-2D RT-F | TCGCGAGCGACAAAAGCAGAAG | qRT-PCR |
| GhWOX4-2D RT-R | CGGGAATAGCTCTAGAGTTCT | qRT-PCR |
| GhWOX11 RT-F | CATCAGTTTTATGCCCTTCAGA | qRT-PCR |
| GhWOX11 RT-R | GCTTTCACCGTGGAGCAAACTC | qRT-PCR |
| GhWOX13a RT-F | GTCGAGTCACCGAATGAAAAGA | qRT-PCR |
| GhWOX13a RT-R | TGCCGTAGTCACCTGCCTGGTC | qRT-PCR |
| GhWOX13b RT-F | GTGTATAACTGGTTTCAAAACAG | qRT-PCR |
| GhWOX13b RT-R | TCTCTGGGTTCTGAAAGCACAC | qRT-PCR |
| GhMCTP7 SalI-F | ACGCGTCGACCTATGAGTAGTCTCAAGTTGGGGGT | yeast two hybrid |
| GhMCTP7 PstI-4C-R | CTTCTGCAGTTACACGGCCTGATGTCGCAGAAT | yeast two hybrid |
| GhMCTP12 BamHI-3C-F | CGCGGATCCGTATGATGCCCCGTCCTCCACC | yeast two hybrid |
| GhMCTP12 SalI-3C-R | ACGCGTCGACTCAAGGATGTGAGTACATATGCAT | yeast two hybrid |
| GhMCTP17 NdeI-F | CTTCATATGCAGAAGCCACCACAGTCTATAG | yeast two hybrid |
| GhMCTP17 XmaI-3C-R | CCCCCCGGGATGCAACATGTTAATCAAACTC | yeast two hybrid |
| GhKNAT1 NdeI-F | CTTCATATGGAAGATTATAATCAGCTA | yeast two hybrid |
| GhKNAT1 BamHI-R | CGCGGATCCTCATGGGCCCAAACGGTAAG | yeast two hybrid |
| GhKNAT2 NdeI-F | CTTCATATGGAGGAATATAATCAGAT | yeast two hybrid |
| GhKNAT2 BamHI-R | CGCGGATCCTCATGGGCCCAAACGGTAAG | yeast two hybrid |
| GhMCTP7 EcoRI-F | CCGGAATTCAGGATACCATGTTCTTGATG | VIGS |
| GhMCTP7 BamHI-R | CGCGGATCCCCATCGATGTCGATGAAAATC | VIGS |
| GhMCTP12 EcoRI-F | CCGGAATTCATGATGCCCCGTCCTCCACCTG | VIGS |
| GhMCTP12 BamHI-R | CGCGGATCCTCCCTGCCTATCCTCCAACCT | VIGS |
| GhMCTP17 EcoRI-F | CCGGAATTCGAAGGGGGATAAAGCTAAAGG | VIGS |
| GhMCTP17 BamHI-R | CGCGGATCCCACCGGTCTATGGTCCAACCT | VIGS |
| GhKNAT1/2 VIGS EcoRI-F | CCGGAATTCGCGGAGGATCGAAGCTCAACT | VIGS |
| GhKNAT1/2 VIGS BamHI-R | CGCGGATCCGTAAGGACCATCACCCATGTA | VIGS |
| GhMCTP7D-771/2 KpnI-F | CGGGGTACCATGAGTAGTCTCAAGTTGGGGGT | LCI assay |
| GhMCTP7D-771/2 SalI-R | ACGCGTCGACCAGCATACTATCTGTCCTTGCAG | LCI assay |
| GhMCTP12A-771/2 KpnI-F | CGGGGTACCATGATGCCCCGTCCTCCACCTG | LCI assay |
| GhMCTP12A-771/2 SalI-R | ACGCGTCGACTCATAGCATGGAATCAGTTCT | LCI assay |
| GhMCTP17A-771 SacI-F | CgagctcATGCAGAAGCCACCACAGTCTAT | LCI assay |
| GhMCTP17A-771 SalI-R | ACGCGTCGACTCATAACATGCTGTCTGATC | LCI assay |
| GhKNAT1-771/2 KpnI-F | CGGGGTACCATGGAAGAATATAATCAGCT | LCI assay |
| GhKNAT1-771/2 SalI-R | ACGCGTCGACTCATGGGCCCAAACGGTAAG | LCI assay |
| GhKNAT2-771/2 KpnI-F | CGGGGTACCATGGAGGAATATAATCAGAT | LCI assay |
| GhKNAT2-771/2 SalI-R | ACGCGTCGACTCATGGTCCCAAACAGTAAG | LCI assay |
| GhMCTP7 XbaI-GFP-F | GCTCTAGAATGAGTAGTCTCAAGTTGGGGGT | GFP localization |
| GhMCTP7 PstI-GFP-R | CTTCTGCAGTTACAGCATACTATCTGTCCTTG | GFP localization |
| GhMCTP12 BamHI-GFP-F | CGCGGATCCATGATGCCCCGTCCTCCACCTG | GFP localization |
| GhMCTP12 SalI-GFP-R | ACGCGTCGACTCATAGCATGGAATCAGTTCTTG | GFP localization |
| GhMCTP17 XbaI-GFP-F | GCTCTAGAATGCAGAAGCCACCACAGTCTAT | GFP localization |
| GhMCTP17 PstI-GFP-R | AACTGCAGTCATAACATGCTGTCTGATCTTGC | GFP localization |

**Supplementary Table 3 |** MCTP sequences in different plant species.

>GRMZM5G852378(CPD33)

MAAGGGGGPPPPGPPPMVRRLAVEVVDARDLVPKDGLGTSSAFAVVDFDGQRKRTRTVPRDLSPQWHERLEFAVHDPANMHAEALDVSLYHDRRFNPSGGGGGSGKNHFLGRVRIYGSQFSRRGEEGIVYFPLEKRSLLSWIRGEVGLKIYYYDEPAMMPPPPEDKPPEQQADNAPPPEVPPEAPRELPEMPAPTEAAVEVQQPAAQPPIIIVEEAPMHPPMMMPPMHGPHGPHGPMMPPMHGPHGPMMPPPMHGPHGPMMPPPPEAPQPEPEPKPEPEFGDQYPPEVRKTRMASSTERVRVVRHPSGGLGPDYYAPSPRVIPGRFVSTGESVEPVQSSSYDLVEPMRYLFVRVVRVRGIRACEGPYVKVQAGPHSLRSRPGRDVSGTGNPEWNQVFAISNAKPEPTLEISVWDGGAPSPAEAFLGGVCFDLSDVPVRDQPDGPLAPQWYRLEGGEPGMVTGDIMVAVWIGTQADEAFPEAWNTDAPYAAYTRSKVYQSPKLWYLRASIIEAQDLRVPAPPPGLPFDVRVKIQLGFQSARTRRSVASSSGSAFAWSEDLMFVASEPLDDNLIVLVEDRSMIKEPALLGHATIPVTSVEQRLDERQIVAPRWFNLEGGTSGIGMPHGYDGGPPAFYSGRLHLRLCLEGGYHVLDEAAHVCSDYRPTAKQLWKPPVGVLELGIIGACGLLPMKTKGGAKGSTDAYCVAKYGKKWVRTRTITDSLNPRWNEQYTWQVYDPCTVLTVAVFDNWRMFAGAGDERQDYRIGKVRVRVSTLESNRAYTASYPLLVLLRSGLKKMGEVQLAVRFSSPAQLPDTWATYTTPLLPRMHYLRPIGVAQQEALRGAAVRTVATWLARSEPPLGPEVVKYMLDANAHTWSVRRAKANWFRIMGVLAWAVGLARWLDGVQRWRNPSTTVLVHALYLVLVWYPELVVPTASLYVFMIGVWYYRFRPRAPVGMDARLSQADTVDGDELEEEFDPVPPPEVLRLRYERLRTLAGRVQRVMGDVAAQGERLQALVSWRDPRASRIFVGVCLAVAVALYAMPPKMVAVASGFYYLRHPMFRDPMPPPAVNFFRRLPSLSDRLL

>GRMZM2G000195

MKLVVEVVGAHDLPARRGRVTPFVQVAFGGQRHATGVRPGEANPTWNETVVFVVDAIVGRLSDRSIDVGVYHRRASGGKSCLGRVRLFGAAVAPSAEEAVLLRCPLDKPRFFAPARGEVALRLYLAPYGPPATLAAAGNAYSSTYATTFNDTASMAGGPETVVGGADTQSSPAPVTKKKEPVQEPAVHVFNSIPTQSSTGSLIFPPPPPPSMPPPTGAAKATKKAAPGTAGDAKAAEYLMVDKLEFLYVNVVRARGLSGTDLTLGTDPYVEVRVGNYSAVTRHLVRNHEPEWNQVFAFSKDQLQADNVELIVKDKNLIVWDSIVGKADLSIAEVPSLALPNRPLAPQWYRLKGAKGQWTGGEVNVAAWKGSQSDEAFAGALHAGAHDLALPAVAATQTKSYYAPRLCYLRCHVIAAQDLVHPESSRRSRMSVLARVQLGAQRLSTRASPSARWDENFFLVAAWPFDEPLEIAVMDIASPERHELLGEVTFPRGSIKVQQFDKTKFMPPAPLWYDLNLPRSSDGGGDGEGDARDRGRRHDFSRKIQLRVYYDAAYHVLDEAMSYASDFQPSAKSLRSQAIGVLELAVLRATGLRSTKRPNGGRVAVNAYCVAKYGHKWIRTRTLLDTASPSWQEQFTFDVFDPCTVLTVALFDNSQLSDEASRRGDTDAPLGKVRIRVSTLASGRTYEQPYSLFVVHPTGLLRCGELHLAVRFTHTAWLNMISLYLRPMLPNQHFAKPIPTHLVPRLRRHAADAIASRLARAEPPLLPGVVHYVLRDPSTYPRPDVSQDYAYSMRRSLAACARLRDVLAPLAAFGRWFRGVRDWDNPVTTVLVLIVFFVLVWMPSLIISTFFLYLFSLGVWNFWRRPARPAQMEHYSDGVPQAMFEEEFDAGFPSGTTPEALHERYWRLRGTATSIQVFIGDVASKGERVHALLAWRDGRATVIALVVVAALTVVTYAVPFRALVSVTGVYVMRHPLLRRKEPSALMSFFRRLPSDAEVML

>GRMZM2G064852

MVAEGARRRVVVEVCNARNLMPKDGQGTACAYAVVDFDGQRRRTATRPRDLNPQWGERLEFLVHDPGAMASETLELNLYNDKKAITAAGSGRRGGTFLGKVKVAGASFAKAGDETLVYYPLEKRSVFSQIKGEIGLKIWFVDDPPPPPAPAAAEEKEAAAAAAGEGKDVKDKAPDAAAAPAAEEKKPETAPSEEKKPAEAKAEEKKPESAEKKDDKKKSPEKGKKDGDKPKEEGKAEKDKKDAAAPPPSPSKLAPPRsPSKKDLAIAGVAGDLEIRPQSAAEKSMAASGASASYDLVDRVPYLFVRLLKAKRHGGGDGQPLYAQLSIGTHAVRTRAATAAGEWDLVFAFHKDSLTDTSLEVTVLEEAKKPAKEGDPVPPEANLGFVSFDLQEVPKRSPPDSALAPQWYTLEGHGSEDGAAVCDVMLSVWVGTQVDEAFQEAWQSDSGGYLVHTRSKAYLSPKLWYLRLSVIQAQDLRLPSPPDAKAKQCGPIFPELYVKAQLGAQVFKTGRVQLGSAAAGTANPSWNEDLLFVAAEPFDPFLTVVVEDVFSGQAVGQSRVPLSTVHRRSDDRVEPPSRWLNLCGGEARPYAGRVHVRVCLEGGYHVLDEAANVASDVRAASKQLSKPPVGMLEVGVRGAANLVPMKIAKDGASGSTDAYVVLKYGPKWARTRTILDQFNPRWNEQYAWDVFDPCTVLTIAVFDNVRYKAAAADDPGKLPRDSRIGKLRIRLSTLDTNRVYANTFALTAVHPVGVRKMGELELAIRFTCPSWLTLMQAYGSPLLPRMHYVKPLGAAQQDVLRHTAMRTVSGRLARSEPPLGPEVVQYLLDTDTQSWSMRRSKANWFRVVGCLSHVATAVRWAHRVRTWAHPPTTVLVHLLLVAVVLCPEMILPTVCLYLFLVLLWRYRARARQPAGMDPRLSHVDSVSPDELDEEFDGLPSGRPADVVRMRYDRLRAVAARAQTLLGDVAAQGERVEALLSWRDPRATAVFAVVCLLAALVLYAVPFKVLLLGMGFYYLRHPRFRGDMPSAGFNFFRRLPSLSDRVF

>GRMZM2G066153

MKGATPPRPFMVPGPGGPMPPPQQQFGLVETRPPLAAVLRPRFNIPGLHPSAAAAAAASGAGKIASTYDLVEPMRFLYVHVVKARDLPAVSATGSIDPFVEVKLGNFKGTTPVRAASHSPSWQQVFAFSAAHLQSHLLEVALKAKDLAGDDLVGRVAFDLSEVPVRVPPDSPLAPQWYRLETKRGEKLPHGEIMLSVWLGTQADEAFPDAWHSDAHAAAGPAAVASTRAKVYFSPKLVYLRVAAIAAQDLVPHDASRPMTACVKLQLAGQVRRTRPGAPPGTPNPIWNEEFMFVASEPFDEPLLVTVEDRVAPGRDEILGRIVLPLKAAMPRHDHFGKPVEPRWYSLMRHSDDPDKKEVKFASKIQIRMSLDFGYHVLDESTYYSSDLQPSSKPARKPSIGMLELGVLGARNLIPMKPKDGRTTDAYCVAKYGPKWVRTRTILDTLNPQWNEQYTWEVFDPCTVITVVVFDNGQIGSKNGGGPDQRIGKVRIRLSTLETDRVYTHFYPLLVLHPSGLKKTGELHLAVRFTCTAWVNMMALYGRPLLPKMHYTHPIAVMQLDYLRHQAMQIVAARLSRAEPPLRREVVEYMLDVDSHMFSLRRSKANFHRITSLFFGFVAMLKWYHSIRSWCNPITTMLVHMLFLILICYPELILPTIFLYMFMIGLWNYRYRPRHPSHMDTKLSHAELTHPDELDEEFDTFPSSRPAEIVRMRYDRLRSVGGRVQAVVGDLATQGERAHALLSWRDPRATAIFIFLSLVIAVVLYVTPFQVLMVIAMLYLLRHPRFRSRMPSVPFNFYRRLPAKSDMLL

>GRMZM2G074754

MEERGFPNDATKEGNDAYKLATYKLGVEVVSAHDLMRKEGQGSASACVELTFDGQRFRTVVKEKDLNPVWNERFYFNISDPSNLRALALEAYVYSVNKTIESSRSFLGKVRIAGTSFVPFPDAVVMHYPLEKRGMFSRVKGEMGMKVYITNDPAIKASNPLPAMDPVSNNPPPAPSTAEQIAADIIGTNLHKSQEHRSEAKTLHTIAKEVHHNHGHLPASFGEQPSKYSVDQMKPGSQPPRIVRmYsAASQQPmDYALKETSPFLGGGQVVGGRVIHGEKNASTYDLVERTQYLFVRVVKARDLPDMDVTGSLDPYVEVRVGNYRGITKHFEKQKNPEWNAVFAFSRDRMQASVLEVVVKDKDLIKDDFVGFVRFDLNDVPIRVPPDSPLAPEWYRLVGKSGDRSMGELMLAVWVGTQADEAFPDAWHSDAATLEDPSTVTHMKSKVYHAPRLWYLRVNIIEAQDVAILDKTRCPDVFVRAQVGHQLGRTKPVQARNFNPFWNEDIMFVAAEPFEDHLVLTLEDRVGPNKDEMLGRVIIPLAMVDRRADDRIVHGKWFSLEKPVLVDVDQLKRDKFSTRLHIRLCLDGGYHVLDESTNYSSDLRPTAKQLWKPSIGLLELGVLGAQGIVPMKTRDGKGSSDTYCVAKYGSKWVRTRTIMNNPHPRFNEQYTWEVYDPATVLTVGVFDNGQLGEKTSSGKDGKIGKVRIRLSTLESGRVYTHSYPLLVLHPSGVKKMGELHLAIRFSSTSLVNMLYLYSRPLLPKMHYVRPIPVLQVDMLRHQAVQIVAARLSRMEPPLRKEVVEYMTDFDSHLWSMRKSKANFFRLVTVFSGLFAASRWFIGICSWKNPITTVLVHILFIMLVCFPELILPTVFLYMFLIGIWNFRYRPRYPPHMNTKISHAEAVHPDELDEEFDTFPTSRNPEIVRVRYDRLRSVAGRIQIVVGDIATQGERVQALLSWRDPRATSVFVLFCLIAAIVLYVTPLQVLAALGGFYVMRHPRFRHRLPSVPVNFFRRLPARTDSML

>GRMZM2G100864

MMQRPLLRPEEYSLKETSPHLGGAAAGDKLTTTYDLVEQMQYLYVRVVKAKELPNMDITGSCDPYVEVKLGNYKGQTQHFEKKNNPEWNQVFAFSKERIQSSVVEIVVKDKDLVKDDFIGRVIFDLNEVPKRVPPDsPLAPQWYRLEDRNGHKVKGELMLAVWMGTQADEAFPEAWHSDAASVPGDGLASIRSKVYLTPKLWYLRVNVIEAQDLIPNDRARFPEVYVKAMLGNQVLRTRAPSRTLNPMWNEDLMFVAAEPFEEHLILSVEDRVAPGKDEVIGRTMISLHHVPRRLDHRLLTSQWYNLEKHVIIDGEQKKETKFSSRIHLRICLEGGYHVLDESTHYSSDLRPTAKPLWKPSIGMLELGILTAQGLLPMKTKDGRGTTDAYCVAKYGQKWVRTRTIIDSFTPKWNEQYTWEVYDPCTVVTIGVFDNCHLNGGEKVNGARDTRIGRVRIRLSTLETDRVYTHSYPLIVLTPGGVKKMGEVQLAVRFTCSSLLNMMHLYTQPLLPKMHYVHPLSVMQVDNLRRQATNIVSTRLGRAEPPLRKEIVEYMLDVDSHMWSMRKSKANFFRIMSVLSPLVAVTKWFDQICRWRNPLTTILIHVLFMILVLYPELILPTVFLYLFLIGVWYYRWRLRQPPHMDTRLSHAETAHPDELDEEFDTFPTSRPPDVVRMRYDRLRSVAGRIQTVVGDLATQGERLQSLLSWRDPRATALFVVFCFVAAIVLYVTPFRVVVFLAGLYMLRHPRFRHKMPSVPLNFFRRLPARTDSML

>GRMZM2G108149

MMQRPPLRPEEYSLKETTPHLGGAAAGDKLTTTYDLVEQMQYLYVRVVKAKELPNKDITGSCDPYVEVKLGNYKGQTGHFEKKNNPEWNQVFAFAKERIQSSVVEILVKDKDLVKDDFIGRVIFDLNEVPKRVPPDsPLAPQWYRLEDRNGHKVKGELMLAVWMGTQADEAFPEAWHSDAASVPGDGLASIRSKVYLTPKLWYLRVNVIEAQDLIPNDKTRFPEVYVKAMLGNQVQRTRALASRTLNPLWNEDLMFVAAEPFEEHLVLSVEDRVAPGKDEVIGRTIIALQHVPRRLDHRLLTSQWYNLEKHVIIDGEQKKETKFSSRIHLRICLEGGYHVLDESTHYSSDLRPTAKPLWKPSIGILELGILTAQGLLPMKTKDGRGTTDAYCVAKYGQKWVRTRTIIDSFTPKWNEQYTWEVYDPCTVVTIGVFDNCHLNGGEKANGARDTRIGKVRIRLSTLETDRVYTHSYPLIVLTPGGVKKMGEVQLAVRFTCSSLLNMMHLYSQPLLPKMHYVHPLSVIQVDNLRRQATSIVSTRLGRAEPPLRKEIVEYMLDVDSHMWSMRKSKANFFRITGVLSPLFAVARWFDQICHWKNPLTTVLIHVLFMILVLYPELILPTIFLYLFLIGVWYYRWRPRQPPHMDTRLSHAETAHPDELDEEFDTFPTSRPPDLVRMRYDKLRSVAGRIQTVVGDLATQGERLQSLLSWRDPRATALFVVFCFVAAIVLYVTPFRVVVFLAGLYVLRHPRFRHKMPSVPLNFFRRLPARTDSML

>GRMZM2G123122

MKLVVEISDAADLAPKDGTASCNPYVEVDFDDQRQRTATKPADRSPYWNQTLVFDVRDPARFPSLPIDVSVFHDRRLQDHNALRPQTFLGRVRINGASVAPSPQEAVLQRYPLEKRSFFSRVSGDIAIRIYLVGDDVNNESVQVPAPPNHHHHPQQQQQQQESVAAAAATGDPERMVRSAFAPQQPSAVEQPAAAGNGGDEHEARPPRIFRSVPAAEPRRATLHAVAAPPPPPGQTVVMPKPAASAPPGPAFGLVETKPPLPAKMGPRAAAAAAAKIASTYDMVEPMTYLYVSVVKARDLPNMDVTGALDPYVEVKLGNFKGVTKHLDKNPNPVWRQTFAFSREHLQSNLLEVAIKDKDMIKDDFVGRVLFDMTDIPQRVPPDsPLAPQWYRLADRSGEKLRHGEIMLAVWIGTQADEAFPEAWHSDAHSLPFEGLSNTRSKVYYSPKLAYLKVVAIAAQDVVPAGSEKGRPLAPVIAKIQLGWQVRRTRPGQPQGSANPVWNEEFMFVAADPFDEPLVVTVEERVAAGRDEPVGRVIIPVQLPSYVPRNDVAKSVEAKWFNLSRALTADEAAAGVTAAKALSEKTTFSSKIHLRLSLETAYHVLDESTHYSSDLQPSAKKLRKSPIGILELGILSARNLVPMKAKEGRLTDPYCVAKYGSKWVRTRTLLNTLAPQWNEQYTWEVFDPCTIVTVAVFDNGYVLGGGEGSKDQRIGKVRVRLSTLEIDRVYTHFYPLMTLTPGGLKKTGELHLAVRFTCTAWANMLGMYGKPLLPKMHYSHPISVLQLDYLRFQAMQMVAARLGRAEPPLRREVVEYMLDVDSHMFSLRRSKANFYRITSLFSGAVAVAKWMEGICKWKNPLTTVLVHVLFLILVCYPELILPTVFLYLFMIGMWNYRRRPRKPPHMDTVLSHAESGLVHPDELDEEFDTFPTSKPGDVVRMRYDRLRSVAGRVQTVVGDLATQGERAQALLSWRDPRATAIFIMLSLVVAVVLYVTPFQVVAVVLGLYLLRHPRFRSKQPSVPFNFYKRLPAKSDMLL

>GRMZM2G129642

MAQGHGGDPHHEDFQLKDTNPLLGEQWPKGAAGPVRPAGGGIAGWLGVDKPSSTYDLVEQMFFLYVRVVKAKDLPPNPITGAPMDPYVEVRLGNYKGKTRHFDRRANPEWDQVFAFSKSRVQSNVLEVFLKDREMLGRDDYVGKVTFDLAEVPTRVPPDsPLAPQWYRLEERRGEGGKVRGELMLAVWIGTQADEAFPEAWHSDAAAVRGEGVASVRSKAYVSPKLWYLRVNVIEAQDVQPQERGRAPEVFVKAQVGNQILKTSVAAPTPTLSPRWNEDLVFVVAEPFEEQLVLTVEDRVSPRKDDLLGRAVLPLTLFDKRLDHRPFVQSRWFDLEKFGVGAAIEGETRRELRFASRVHVRACLEGAYHVMDESTMYISDTRPTARQLWKPPVGVLEIGILGAAGLQPMKTRDGRGTTDAYCVAKYGQKWVRTRTMIGSFAPTWNEQYTWEVFDPCTVITIGVFDNCHLGGGSNGGAGQPARDARIGKIRIRLSTLETDRVYTHAYPLIALQRSGVKKMGELRLAVRFTCLSLMNMVHLYTQPLLPRMHYLHPFTVTQLDALRYQAMGIVAARLGRAEPPLHREVVEYMLDVESHMWSMRRSKANFFRAVSLFSGVAGAARWFGDVCRWRNVATTALVHVLLLILVWYPELILPTVFLYMFLIGLWNYRRRPRHPPHMDTKMSWAEAAHPDELDEEFDTFPTSRPQDVVYMRYDRLRSVAGRIQTVAGDMATQGERLQSLLGWRDPRATCLFVVFCLLAAVVLYVTPFRIVALVAGLYVLRHPRFRSRLPSVPSNFFRRLPSRADSML

>GRMZM2G131176

MKGAMPPRQFMVPGPGGPMLPPQQQFGLVETRPPLAAVLRPRFNIPGLHPSAAAAAAASGAGKIASTYDLVEPMRFLYVHVVKARDLPAVSATGAIDPFVEGGQPQPVLAAVFAFSATHLQSHLLEVALKAKDLAGDDLVGRVAFDLAEVPVRVPPDsPLAPQWYRLETKRGEKLPHGEIMLSVWLGTQADEAFPDAWHSDAHAAAGPAAVASTRAKVYFSPKLVYLRVAAIAAQDLIPHDTSRPMSACVKLQLAGQLRRTRPGAPPGTPNPIWNEEFMFVASEPFDEPLVVTVEDRVAPGRDEMLGRIFLPLAAAMPRHDHFGKPVEPRWYSLMRPSDDPDKKEVKFASKIQIRMSLDFGYHVLDESTYYSSDLQPSSKPARKPSIGMLELGVLGARNLVPMKPKDGRTTDAYCVAKYGPKWVRTRTILDTLNPQWNEQYTWEVFDPCTVITVVVFDNGQIGSKNGGGPDQRIGKVRIRLSTLETDRVYTHFYPLLVLNPSGLKKTGELHLAVRFTCTAWVNMMALYGRPLLPKMHYTQPIAVMQLDYLRHQAMQIVAARLSRAEPPLRREVVEYMLDVDSHMFSLRRSKANFHRITSLFFGFLAMLKWYDGIRSWWNSITTVLVHMLFLILICYPELILPTIFLYMFMIGLWNYRFRPRHPSHMDTKLSHAELTHPDELDEEFDTFPSSRPAEIVRMRYDRLRSIGGRVQTVVGDLATQGERAHALLSWRDPRATAIFVFLSLVVAVVLYVTPFQVLMVIGMLYLLRHPRFRSRMPSVPFNFYRRLPARSDMLL

>GRMZM2G163925

MAGVLPRFGPFGPLPPSDEFGIRETRPRLAGRRAGGYDLVERMEYLYVRILKARDLKWTGSFDPLAEVKLGSYSCATRHIEKTTSPEWNDVFAFSRERIQASFLDVVVKGKGFAKDDFVGRLRFDLADAPLRVPPDSALAPQWYHVFDKKAERGGEVMMAVWFGTQADECFPLAVHADAAFAVDAKLAAHIRCKQYTVPRLWYVRVNVIEARDIAFADKARVGEVFVRSRIAAQVHKTRTCVARLPTCGWNEDHMFVAAEPFEDHLILSVEDRVKVDKEEVIGHVHIPFKEFERRWDARPIRPRWFNLVRPEGAAKIDKFSAKICVRLCLEGGYRVLTEPVHYLSDVRPAARELWHHRPPIGLIELGIHNAFGLSSVRTRDGRGSCDAYCVAKYGVKWFRTQTVIDSLAPRFHQQCFWDVHDHCTVLTVAVFHNCQIGDKGGLVTGDPVKDILLGKVRIRLSTLETGRIYTHAYPLVSLHGGGIKKMGELQLAVRFSSTSTLGLLQTYAQPHLPPMHYHSPLSIVHQETLRREAVSLIAHRLGRMDPPLRRECIEHLCEAHSHRWSMRRSKAHFFRLMAALAPLFTGLRWFVDVCHWKNPSTTVAVHIIYAMLVCCPNLIMPTFFMYKFLIGLWNYRRRPRHPWHVDTKVSHAEMAHLDELDEEFDDFPTARRPEVIRMRYDRLRSLGARIQEMVGDVAAHAERARCAMTWRDPRATAMYLLACLFLAVTTLLAPFQAVALLTGFYVMRHPTLRQRLPDVPANFFRRLPCKVDCLL

>GRMZM2G386643

MATYKLGVEVASAHDLMPKDGQGSASACVELTFDGQRFRTAVKEKDLNPVWNERFYFNVSDPSNLPELALEAYVYNVNKTLESSRSFLGKVRIAGTSFVPFPDAVVMHYPLEKRGMFSRVKGELGMKVYITNDPAIKASNPLPAMDPVSNNPLPAPSPAEQIAADITGTNLHTSQEHRSEAKTLHTIAKEVHHHHNHGHLPATFGEQPSKYSIDQMKPQSQPPRIVRmYsAASQQPmDYALKETSPFLGGGQVVGGRVIRGEKNASTYDLVERMQYLFVRVVKARDLPDMDVTGGLDPYVEVRVGNYRGITKHFEKQKNPEWNAVFAFSRDRMQASVLEVVVKDKDLIKDDFVGFVRFDLNDVPIRVPPDSPLAPEWYRLVSKSGDKSMGELMLAVWVGTQADEAFPDAWHSDAATLEDPSAVTHMKSKVYHAPRLWYLRVNIIEAQDVAILDKTRYPDVFVRAQVGHQLGRTKPVQARNFNPFWNEDIMFVAAEPFEDHLVLTLEDRVGPNKDEMLGRVIIPLAMIDRRADDRIVHGKWFNLEKPVLVDVDQLKKEKFSTRLHLRLCLDGGYHVLDESTNYSSDLRPTAKQLWKPSIGLLELGVLGAQGIVPMKTRDGKGSSDTYCVAKYGSKWVRTRTIMNNPNPRFNEQYTWEVYDPATVLTVGVFDNGQLGEKTGEKTSSGKDGKIGKVRIRLSTLETGRVYTHSYPLLVLHSSGVKKMGELHLAIRFSSTSLVNMLYLYSRPLLPKMHYVRPIPVLQVDMLRHQAVQIVAARLSRMEPPLRKEVVEYMTDFDSHLWSMRKSKANFFRLMTVFSGLFAVSKWFSGVCSWRNPITTVLVHILFIMLVCFPELILPTVFLYMFLIGIWNFRYRPRYPPHMNTKISHAEAVHPDELDEEFDTFPTSRNPEVVRMRYDRLRSVAGRIQTVVGDIATQGERVQALLSWRDPRATAVFVLFCLVAAIVFYVTPLQVIAALGGFYVMRHPRFRHRLPSVPVNFFRRLPARTDSML

>GRMZM2G400173

MSNLKLGVEVTSAHDLLPKEQDTANPFVEVDFDGQKFRTAVKDRDLNPVWNEQFYFNISDPSRLPELHLEAYVYHADRASNSKSCLGKVRISGTSFVSQPDAMPLHYPLEKRTILSRARGELGLRVFLTDDPSVRVSAAPAQQEFDMLSTPTTAQEQQAAANSISNPFQETRANPVRQFQHLPREQQRPAQPQPYYAEGSYGDQQQQQRSFSAVANKAAAPQPQVQVSRMYAPGPQQPVDFQLKETSPTLGGGRVIGGRVYPGQKAGAYDLVEKMQYLFVRVVKARDLPNMDITGSLDPYVEVHLGNYKMKTKHFEKNQRPEWDEVFAFPKEVMQSTMLQVVVKDKDVLRDDYVGRVSIDLNEVPLRVPPDSPLAPEWYRLMGKDGVRDRGELMLAVWYGTQADECFPSAIHAGSTPVDSHLHSYIRGKVYPAPRMWYVRVNVIEGQDIYPMENRIPDVLVKVRLGHQLLRTRQVRSPTRNFMWNEELMFVAAEPFEDDLLISVVDRVAQDKDEVIGEAIIPLARLPRRADHKPVLPAWFDLRRPGIIDVNQLKEDKFYAKVSLRVCLEGGYHVLDESTQYCSDLRPTMKQLWKPPIGMLEVGILSANGLNPTKTRNSRGSCDAYCVAKYGSKWVRTRTIVDSLSPRFNEQYTWEVFDHGTVLTIGLFDNCHISGDDNKDGSSGHMDKPIGKVRIRLSTLDTARVYTHSYPLLFLSPSGVKKMGELHLAIRFTVTSLINVLFTYSRPLLPKMHYAQPLSIVQQEMLRHQAVLLVAQRLGRMEPPVRREVVEFMSDARSHLWSMRRSKANFFRLMQVFSGFIAAGKWFADVCQWKNPVTTVLVHVLFIMLVLYPDLILPTIFLYMFLIGLWNYRFRPRFPPHMNTRISYADVALPDELDEEFDTFPTSKSPDLIRMRYDRLRHVAGRIQTVVGDIATQGERLQSLLSWRDPRATAMFLIFCLITAIILYVTPFQVVALCLGFFGMRHPRFRHKVPSAPANFFRRLPAKTDSLL

>GRMZM5G800598

MAKAEKLVVEVVAAHNLMPKDGQGSSSPYVEVEFEHQKRRTRARPKELNPVWNERLVFPVSDPDDLPYRAIDVGVYNDRGAAVGGGGAPHGRNFLGKVRVPSAGVPAPGEEAVPQLFTLEKRSLFSHIRGEITLKIYRVNSGDVVVKSKQEKPAKAVVVGPEVVAAPTVTGPKKQPHSHPHPPPPQQQHQRHPLAAVQPPPEPPMDVMPQPPVPMAMKPVAMHADPYPVPPMFSGPADFSLKETRPRLGSGVVADKASATYDLVEQVEYLYVRVVRARGVPMATEAVAEVKLGNYRGVTPAVPSHNWDQVFAFSRETIQSSFVEVFVRARGSDDHVGRVWFDLSEVPRRAPPDSTLAPQWYSMEDRKGQRGGAEVMLAVWFGTQADESFAEAWHSKAAGVHGNGALGSIRSKVYVAPKLWYLRVSVIEGQDLFPMDKGPLAIGRFPELFVRAQVGSQIMRTRPAPVVSTRGPASPFWNEDLMFVVAEPFEEFLVLSVEDRVSPGRDELLGRLVVPVSAIERRWDWKPVVSRWFGLDCGTGGGGNVAGNSVHRFGSRRVHLRLSLDGGYHVLDEATAYSSDLQPTAKQLWKPHVGVLELGVLGATGLMPMKSRDGGRGATTDAYCVAKYGQKWIRTRTIVDSLCPRWNEQYTWDVFDPCTVITVGVFDNCHVDGASGSAARDSCIGKVRIRLSTLETDRVYTHAYPLLMLHPTGVKKMGELHLAVRFACGNAGNMFHAYAHPLLPKMHYAEPLLVRQVETLRCQATNVVAARLGRAEPPLGKEVVEYMLDHRSSLWSMRRSKANFFRLINVLSGPVAIGRWFELVRSWQRPVHSCLAVFTFLVFLATPELVLPTAFLAMAFAGLWRYRGRPRHPPHMEMRLSHADGATADELDEEFDTFPSTRGDVVRFRYDRLRSVAGRVQTVVGDIATQGERMQAVLSWRDPRATLLFAVACVAAAVIAYCVPTKVMVGMWGLYAMRPPRFRSRMPSPLMNFFRRLPSRADILL

>GRMZM5G807350

MYSFVPPCMPFLAGDGEADRLTNRRARSSAAGAAILPASPSMAYAYPYVFRAQAPPVSVEDHKAKDAAPAPQVKEQWPAGGSRSASPRGAGTGWPDGLGSGSGESQRLASAYDLVETMHYLYVRVVKVRGLPASAVTGGCRPYVEVRVDNYRGATRHCEGKESPEWNLVFAFSRDRVQATVLEVFVRDRDALGRDDCVGRVAFDIAEAPVRVPPDsPLAPQWYRLEGSAGGRMVANGEVMLAVWVGTQADEAFPDAWHATAASVLGGDGGAAVHNTRSKVYVTPKLWYLRVGVLEAQDVVPPGACATPDKGRHAEVFAKVQVGGTVLRTRPCTTRGPTNLAWNEELVFAVAEPFEDPAVLIIEARVHPGKDEIVGRALLPLTIFEKRLDCRPVQSQWFSLEHFGRPAPAVFAGRVHLRACLEGAYHVMEEPTMYASDTRPTARQLWRPPIGVLEVGVLGAQGLTPMKTVDGRGMTDAYCVAKYGQKWVRTRTVVDSCSPRWNEQYTWEVYDPCTVLTLAVFDNCHLGSASAGNGALRDQRIGKVRIRLSTLEMDKTRTSAHPLVVLHPSGLRKNGELCLAVRLTCLTLGSVVRMYGQPLLPKAHYVQPLTVVQLDSLRRQAMSIVAARLSRAEPPLRREVVEYMLDADSLVWSIRRSKANFFRVTALLSGAASTVRWLADVCRWKNPATTVLVHVLFVTLMCFPELILPTMFLYMSTAGLWNYRRRPRRPPSMDAGLSCAEATHPDELDEELDTFPTSRPNAVVRLRYDRLRSVAGRIQTVVGDVATQGERIRSLLTWRDPRATALFTAFCLVAAAVLYVTPVRVVSLVVGLYVLRHPRFRGRMPSAAGNFFKRLPSQADTML

>Medtr0291s0010

MSSSKPAPKPNTDDYKLKDTKPELGEKWPHGGQRGGTGWLYSERATSTYDLVEQMFYLYVRVVKAKELPPNPVTGNVDPYVEVKVGNYKGKTRHFEKKTNPEWKQVFAFSKEKIQSSVVEVFVRDKEMVARDDYIGKVEFDMHEVPTRVPPDSPLAPQWYRLGNLKGETRTRGEVMLAVWMGTQADEAFPEAWHSDSASVKGEGVYNIRSKVYVNPKLWYLRVNVIEAQDVQPHDKSQPPQVFVKAQVGQQVLKTKLCPTKTPNPMWNEDLVFVAAEPFEENLVLTLENKASPGKDEVVAKLTLPLNKFETRMDHRPVHSRWYNVERFGFGVLEGDKGNELKFSSRIHLRVCLEGAYHVLDESTMYISDTRTTARQLWKQPIGILEVGILSAQGLSPMKTSNGKSSTDAYCVAKYGMKWVRTRTITESFNPKWNEQYTWEVHDPCTVITFGVFDNCHLGGGNSQQSGAKTNDAKIGKVRIRLSTLEMDRIYTNSYPLLVLKPSGLKKMGELQLAIRFTCLSLAHIIYLYGHPLLPKMHYLHPFTVNQLDSLRYQAMNIVAVRLGRAEPPLRKEVVEYMLDVDSHIWSLRRSKANFFRIVSLFSGVISMSKWLGEVQKWKNPVTTILVHVLFFILICYPELILPTIFLYMFLIGIWNFRKRPRNPPHMDTKISWAEAAHPDELDEEFDTFPTSKAQDVIRMRYDRLRSVAGRIQTVVGDIATQGERLQALLSWRDPRATFLFVIFCLVTAVALYVTPFKIVISVAGIFWLRHPKFRSKLPSVPSNFFKRLPSGADSML

>Medtr0305s0020

MQRPPPEDFLLKETKPHLGGGKISGDKLTSTYDLVEQMQYLYVRVVKAKDLPSKDVTGSCDPYAEVKLGNYKGTTRHFEKKTNPEWNQVFAFSKDRLQASVLEVTVKDKDVVKDDFIGRVWFDLNEVPKRVPPDSPLAPQWYRLEDRKGDKVKGELMLAVWMGTQADEAFPEAWHSDAATVSGTDALANIRSKVYLSPKLWYLRVNVIEAQDLQPTDKGRYPEVFVKAILGNQALRTRISQSRSINPLWNEDLMFVAAEPFEEPLILSVEDRVAPNKEEVLGRCAIPLQFMDRRLDHKPVNTRWFNLEKHIIVEGEKKKEIKFASRIHMRVCLEGGYHVLDESTHYSSDLRPTAKQLWKSGIGVLEVGILSAQGLMPMKNKDGRGTTDAYCVAKYGQKWIRTRTIIDSFMPRWNEQYTWEVFDPCTVITIGVFDNCHLHGPDKAGGAKDSRIGKVRIRLSTLETDRVYTHSYPLLVLHPTGVKKMGEIQLAVRFTCSSLLNMMHMYSLPLLPKMHYIHPLTVSQLDSLRHQATQIVSMRLSRAEPPLRKEVVEYMLDVGSHMWSMRRSKANFFRIMGVLSGLIAAGKWFDQICNWKNPITTVLIHILFIILVMYPELILPTVFLYLFLIGIWHYRWRPRHPPNMDTRLSHADSAHPDELDEEFDTFPTSRPSDIVRMRYDRLRSIAGRIQTVVGDLATQGERLQSLLSWRDPRATALFVIFCLLAATILYVTPFQVVALLTGIYVLRHPRFRHKLPSVPLNFFRRLPARTDCML

>Medtr1g015540

MANNENAPKETSVNNNAAFEADKLTRRYDLVEEMEFLFVRVVKVIDFPNIHNLYVEVVLGNAKATTFFLETSNSSLNQVFAFDNGKNSSSNVDVFLKDRTSGMFIGHVKFAVGDIPKRVPPESSLAPQRYTLEDQAGTNLARGAIMLSMWFGTQADEYFPQAWCSDTTEITDDSVCYTRSKVYMSPSLRYVKVTVIQAHHLLLQFPPESSELFVQVGLGKSFCLRTSFSKEKSAKPFWNEDLMFVTQEPFDEELVLSVEQVRLADHVNVSLGTYTTNLNNSNDVDIRFDDVPADDRWVDLNRPGIIENAREVKFASKIHLRISLNGGYHVSDEPLEYSSDFRPSSRDHWPPSIGVLELGILKATNLMPMKIGGRTDAYCVAKYGPKWVRTRTSVDSREPRWNEQYVWEVYEPFTVITIGVFDNNQLDPESRARGARDTIMAKIRIRLSTLENGKVYAHSYPLIGLHPSGVTKMGEIHLAVKFTWTSQSTFTFPFESIFNKCALYGRPLFPAVHYFLPLSPTQFDTLRNQAFRIISVSLSEAEPALREEVVSYMLDMRSDMWSMRKGIANYNRIMSLISYFFAFWKWLEDIRQWKNPIEAVLFHIFCLCVLLYPEPMIPLVSFYLFKIGLDNYNFKKHEHPCHIDATLSGADTTNYDDLEEELVFFPTQIGGEHLRRRYDRLRVIGRNGQKRVDELATILEKLQSLISWRDPRATFIFLVFCVVCLPVTYFVPLKVIIFPCIFIYLRHPRFRSNTPWHAENIFRRLPSKQAFIL

>Medtr3g027150

MMSIKRRPRGNNPMTMHAINPQVQQGHPNSHHEEDYNVRDTSPQLGERWPNGGNYNGRGWMSGGERSTSTYDLVEQMFYLYVRVVKAKNLTLNSLTSTCDPYVEVRLGNYKGRTKHLDKRSNPEWNQVYAFSKDQIQSSILEVIVKDKETVGRDDYIGRVAFDLNEVPTRVPPDSPLAPQWYRLEDRRGEGRVRGDIMLAVWNGTQADEAFSDAWHSDAATVYGEGVFNIRSKVYVSPKLWYLRVNVIEAQDVISSDRNRVPEVFIKAQMGSQVLRTKVCPTRSTTQIWNEDLVFVAAEPFEEQLTITVEDRVHGSKDEVLGKIMLPLTLFEKRLDHRPVHSRWFNLEKYGFGMMEGDRRNEVKFSSRIHMRICLEGGYHVLDESTLYASDHRPTARQLWKQPIGMLEVGILGAQKLLPMKMNNSRGSTDAYCVAKYGQKWIRTRTILDTFSPKWNEQYTWEVYDPCTVITLGVFDNCHLGGGGEKAPSGGSNAARDSRIGKVRIRLSTLEANRIYTNSYPLLVLHQNGVKKMGELQLAIRFTTLSIANMVYIYGQPLLPKMHYLSPFTVNQVENLRYQAMNIVAMRLGRAEPPLRKEAVEYMLDVDSHMWSMRRSKANFFRMMSLFSSAITMGKWFNQVCNWKNPVTSVLVHILFLILILYPELILPTIFLYMFLIGLWNYRFRPRNPPHMDTKLSWAEGANPDELDEEFDTFPSSKPHDVVRMRYDRLRSVAGRIQTVVGDIATQGERFHSLLSWRDTRATSLFIVFSLCSAVILYATPPRVVALVTGLYFLRHPKFRSKMPSVPSNFFKRLPAQTDSML

>Medtr4g023460

MKLIVEVINAHDLMPKDGEGSASTFVEVDFENQLSRTRTVPKNLNPTWNQKLVFNLDTTKPYHHKTIEVSVYNDRRQPNPGRNFLGRVRIPCSNIVKEGDEVYQILPLENKWFFSSVKGEIGLKVYIASESKYKDFSPISSSKLAKLSPSTPKQEPESTATNLVPLHGTPSTTETLEADPNEEGSALDASKETTEVEKVHFVAASNYSIEESQSSSIDIDQEPKIEIEEPVEQISSQKLDKHQVHQQPRISIKKRPQDNLFTMHSVDPQLQSSRAENYNHSNDGNMQPRISIKRRPRPQGIPPSTTHSVNPQVHPRYDERYNLKGPNQQPRILVETPRHVSSPPRHGVDPQVNTSNDENYSVEETTNPQIGEKWPSDGAYDGRKWTSSGERLTSTHDLVEQMFYLYVRVVKAKDLPPGTITSSCDPYVEVKLGNYRGRTKHLEKKLNPEWNQVFAFSKDRIQSSVLEVFVKDKEMVGRDDYLGRVIFDLNEIPTRVPPDSPLAPQWYRLQHLRGEGMVRGDIMLAVWMGTQADEAFSDAWHSDAATVYGEGVFNIRSKVYVSPKLWYLRVNVIEAQDVIPSDRNRLPEVSVKAHLGCQVLKTKICSTRTTSPLWNEDLVFVAAEPFEEQLTITVEDHVQPSKDEVLGRISLPLNLFEKRLDHRPVHSRWFSLEKFGFGALEGDRRNEQKFSSRIHLRVCLEGGYHVLDESTLYISDQRPTARQLWKQPIGILEMGILGAKGLLPMKMKDGHGSTDAYCVAKYGQKWIRTRTLLDTFSPKWNEQYTWEVYDPCTVITLGVFDNCHLGEKAPSGSSIKDSRIGKVRIRLSTLEANKIYTNSYPLLVLHQHGVKKMGELQLTVRFTALSLANMFHIYGQPLLPKMHYLQPFTVNQIDNLRYQAMNIVAMRLGRAEPPLRKEIVEYMLDVDSNIWSMRRSKANFFRVMSLFSGLITIGRWFNDVCHWKNHITSILVHILFLILVWYPELILPTCFLYMFLIGLWNYRFRPRQPPHMDTKLSWAESVHPDELDEEFDTFPTSRSHDAVRMRYDRLRTVAGRIQTIVGDIATQGERFMSLLSWRDPRGTTLFVLFSLCAAVIFYATPFRVVVLVTGLYNLRHPKFRNKLPSVPSNFFKRLPARTDSLL

>Medtr4g033215

MNRLVVEVHDASNLMPKDGKGSANPYVQINFDEQQVKTQTKYQDQNPYFNEKFMFNINTSRDLAHKTVEVGVYNHNDKKPNSKKNFLGKVRISGDSIPISESESSIKRYPLEHSKGDIALKMFAFHDPFANTPPTPNSHPPPQHSQTKTSFESFEPDPDEEIPLQEINTNINMEDEENMFSDSEKKKKNKKKKEKEVRTFHSIGTEKEKPSHSHGHGHGHAPAPAPAPASAFPSVNHGANFASFATPRVETQTRVDYAKSGPPNVMLMQIPKQNPEYALVETAPPLAARLRYKGGNKVSTTYDLVEQMHFLYVNVVKAKELPVMDITGSLDPYVEVKLGNYKGVTKHLDKNQHPVWKQIFAFSKERLQSNLLEVTVKDKDLISKDDFVGRIMFDLTEVPVRVPPDSPLAPQWYRLEDKKGMKINHGEIMLAVWMGTQADESFPEAWHSDAHNVSHSNLSNTRSKVYFTPKLYYLRVEVIEAQDLVPHDKGRVPQASVRVQLGSQMRFTRVSQMRGVNPIWNEELMFVAAEPFEDIIIVTVEDKFGPNNVEILGREIMSVRNVPQRLETGKLPDSRWFNLHRPSAVGEEETEKKKEKFSSKIHLRICLEAGYHVLDESTHFSSDLQPSSKHLRRKNIGYLELGILSARNLLPMKGKDGRTTDAYCVAKYGNKWVRTRTLLDTLSPRWNEQYTWEVHDPCTVITVSVFDNHHLNGSSDHKDQRIGKVRIRLSTLETDRVYTHYYPLLVLQPNGLKKNGELHLAVRFTCTAWVNMVAQYGRPLLPKMHYVQPIPVRHIDWLRYQAMQIVAARLARAEPPLRRESVEYMLDVDYHMWSLRRSKANFHRIMSLLSGFTAVCKWLNDICTWRNPITTCLVHVLFLILVCYPELILPTIFLYLFVIGIWNYRFRPRNPPHMDARLSQAEACHPDELDEEFDTFPTTRPADIVRMRYDRLRSVGGRVQTVVGDLATQGERAQALLSWRDSRATAIFIIFSLIWAVFIYITPFQVIAIIVGLFMLRHPRFRSKMPSVPVNFFKRLPSKSDTMI

>Medtr4g057675

MANQNHNQNQRKQKEDFDLKETTPNINAGRVISGDRLPITFDLVEQMKFLFARVVRANDLPETGKSDTCNPFVEVKLGSFVGTTRVFEKISNPEWNQVFAFSKERIQEQVLEIVVKEKDPVADYPYVIGRVAFTISDIPMRVPPDSPLAPQWYKLEGQNMVKLDQGELMVSVWMGTQADESFPDAWHSDATTTSVENITYTRSKVYISPRLWYLRVNVIQAQDLLLKGNSEIFIQGVLGNLALRSRPMKINPNPVWNEDLMFVSAEPFDESLLLSVEQGQGNSNKHENLGSCVIHLKDVEKRIDATPTASVWYNLQKPKELEGKEEVKFSTRLHLRISLDGGYHVLDEATHYSSDLRPSSKYLNKPSIGVLELGILNAVGLSPMKKDRTDAYCVAKYGSKWVRTRTIVDILSPRWNEQYTWEVYDPCTVITIVVFDNGHLHGGGKNNVGGKNGDGGVDKRIGKVRIRLSTLESDRIYTHSYPLINLHTQGAKKMGEIQLAVRFSCPSLLNVLQTYAQPLLPKMHYICPLSMFQIDSLRNQAAAITILRFRRAEPPLSKEVVEFMLDMRSNVWSMRRGRAQFYRIASLLSGFVSIVKLIEEIHSWKNSVTTIGGYSIFCFFNYKPGAILPLTFTFLLLNGIWQYRIRPRYPSHMDIKLSHADTATTEELEEEFDPFPTKFSGGNLHKRYDRLRGISGRVLVVMGDLATQGERVHSLISWRDPRAMALFLIFCLIAAILTYFILFRYILFISVTYVLRPPRLRFDMPAFPQNFLRRMPAKSDGML

>Medtr4g066150

MQKPPNSHEFALKETTPNIGAGAVTRDKLSCTYDLVEQMQYLYVRVVKAKELPTKDVTGSLDPYVEVKLGNYKGITKHFEKKSNPQWNQVFAFSKDRIQASVLEVIVKDKDVIADDFVGRVWFDLNDIPKRIPPDSPLAPQWYRLEDRKGEKVKGELMLAVWMGTQADEAFPDSWHSDAAMVGSEAVANIRSKVYLSPKLWYVRVNVIEAQDLIPSDKSRYPEVFVKVALGNQFSRTRISQLKTINPIWNEDLMFVAAEPFEEPLVLTVEDRVGQNKDETLGKCMIPLQMVQRRLDHKPVNTRWHNLEKHLVVEGEKKDTKFASRIHLRLCLDGGYHVLDESTHHSSDLRPTAKQLWKSSIGILEVGIISAHGLMPMKTRDGRGTTDAYCVAKFGQKWIRTRTIVDSFSPKWNEQYTWEVFDPSTVITIGVFDNNHLHGGDKSKDSRIGKVRIRLSTLETDRVYTHSYPLLVLNPSGVKKTGEVQLAVRFTNSSYFNMLYMYSMPLLPKMHYIHPLSVIQLDHLRHQATQIVSMRLSRAEPPLRKEVVEYMLDVDSHMWSMRRSKANFFRIMKVLSSLIAFGKWFDQICNWKNPITSILIHILFIILVLYPELILPTIFLYMFMIGIWNFRWRPRHPPHMDTRLSHADAAFPNELDEEFDTFPTSRSSDIVRMRYDRLRSIGGRVQSVVGDLATQGERFQSLLSWRDPRATTLFVTFCFVAAMILYVTPFQVVLLITGFYVLRHPRFRQKLPSVPLNFFRRLPARSDSML

>Medtr4g071870

MANQNHNQNQRKPKEDFDLKETTPNINAGRVISGDRLPITFDLVEQMKFLFARVVRAKDLPETGKSDTCNPFVEVKLGSFVGTTRVFEKISNPEWNQVFAFSKERIQEQVLEIVVKEKDPVADHPDVIGRVAFTISDIPMRVPPDSPLAPQWYKLEGQNMVKLDQGELMVSVWMGTQADESFPDAWHSDATTTSVENITYTRSKVYISPRLWYLRVNVIQAQDLLLKGNNEIFIQGVLGNLSLRSRPMKINPNPVWNEDLMFVAAEPFDESLLLSVEQGQGNSSKHENLGSCVIHLKDVERRIDATPTASVWYNLQKPKELEGKEEVKFSTRLHLRISLDGGYHVLDEATHYSSDLRPSSKYLNKPSIGVLELGILNAVGLSPMKKDRTDAYCVAKYGSKWVRTRTIVDSLSPRWNEQYTWEVYDPCTVITIVVFDNGHLHGGGKNNVGGKNGDGGVDKRIGKVRIRLSTLESDRIYTHSYPLINLHTQGAKKMGEIQLAVRFSCPSLLNVLQTYAQPLLPKMHYICPLSMFQIDSLRNQAAAITILRFRRAEPPLSKEVVEFMLDMRANVWSMRRGRAQFYRITSLLRGFVSIVKLIEEIHSWKNSVTTIGGYSIFCFFNYKPGAILPLTFTFLLLNGIWQYRISGGNLQKRYDRLRGISGRVLVVMGDLATQGERVQSLISWRDPRAKALFLIFCLIAAILTYFIPFRYILFISVTYVLRPPRLRFDMPAFPQNFLRRMPAKSDGML

>Medtr4g107850

MNMATTPFQQGPPQTVRRLAVEVVDARNLLPKDGQGSSSPYVVADFDGQRKRTTTRFKELNPVWNELLEFIVSDPDNMEFEELEVEVYNDKKFGNGSGRKNHFLGRVKLYGTQFFGRGEEALVYYTLEKKSVFSWIRGEIGLKIYYYDELLQQDEQQQQQQDQPSQPPPEEERHGGGAEQERNNHSHRHPMMVEEGRVFQVEQMEHCVPLPDGPPSPRVVVMEESPSPVVRVQQDPPLPEMYAQPEPEMQYHHHHPEVRKMQTMRNDRVKIMKRPNGNGNGDYAPKDISGKKPNGESERIHPYDLVEPMQYLFVRIVKVRGLNPPTESPFVKVRTSSHYVRSKPASFRPNEPNDSPEWNQVFALGYSKTDATGATLEISVWDSPTEQFLGGVCFDLSDVPIRDSPDSPLAPQWYRLEGGAAEQNAVRVSGDIQLSVWIGTQSDDAFPEAWSSDAPYVAHTRSKVYQSPKLWYLRVTVMEAQDLNLTPNLPPLTAPEIRVKVQLGFQSQRTRRGSMNHHSMSFHWHEDLLFVAGEPLEDSMVLLVEDRTTKEAALLGHVVIPLTSIEQRIDDRHVPAKWFPLEGGSYCGRVHLRLCLEGGYHVLDEAAHVCSDFRPTAKSLWKPPVGILELGILGARGLLPMKSKGPGKGSTDSYCVAKYGKKWVRTRTVTDSFDPRWNEQYTWQVYDPCTVLTVGVFDNWRMFADVAEEKPDCRIGKIRIRVSTLESNKIYTSSYPLLVLTRNGLKKMGEIELAVRFACHGFFPDTCAVYQQPLLPKMHYIRPLGVAQQEALRGAATKMVAQWLARSEPPMGHEVVRYMLDADSHAWSMRKSKANWFRIVAVLAWAVGLAKWLDDIRRWKNPVTTVLLHILYLVLVWYPDLIVPTGFLYVVLIGIWYYRFRPKIPAGMDTRLSQAEAVDPDELDEEFDTMPSSKPPDLVRVRYDRLRMLAARVQTVLGDFATQGERVQALVSWRDPRATKLFIGVCLVIAVILYSVPPKMVAVALGFYYLRHPMFRNPMPPASLNFFRRLPSLSDRLM

>Medtr5g010390

MINLKLGVDVVGAHNLLPKDGEGSSNAFVELYFDGQKFRTTIKEKDLNPVWNESFYFNISDPSNLHYLTLEAYVHCHSKATNSSSFLGKVSLTGTSFVPQADAVVLHYPLEKRGIFSRVRGELGLKIYITDNPTIKSSIPNPSVESMPTNNHAEVHGPTGSMRNGLSRDKVESSRHTFHHLPNTNHQRHQHQQHSTGYADTHYVPKYEADEMKADQPQPMKLVHMHSVTSLQPVDFALKETSPFLGGGRVVGGRVVHKDKTASTYDLVERMYFLYVRVVKARELPSMDLTGSLDPFVEVRIGNYRGITKHYDKNQNPEWHQVFAFSKERMQASVLEVVIKDKDLIKDDFVGIVRFDINEIPLRVPPDSPLAPEWYRLDDKKGEKVKGELMLAVWIGTQADEAFSEAWHSDAASPVDSTPATTTVIRSKVYHAPRLWYVRVNVVEAQDLIPTEKNRFPDAYVKVQIGNQVLKTKTVPARTLNPQWNEDLLFVAAEPFEDHVILSVEDRVGPGKDEIIGRVIIPLNAVERRADDRIIHSRWFNLEKPVAVDVDQLKREKFASRIQLRLCLDGGYHVLDESTHYSSDLRPTAKQLWRPPIGVLELGVLNAIGLHPMKTRDGRGTSDTYCVAKYGHKWVRTRTLVDNLSPKYNEQYTWEVFDPATVLTVGVFDNSQISGEKGHNKDLKIGKVRIRISTLETGRIYTHSYPLLVLHPTGVKKMGELHLAIRFSCTSFANMLYLYSKPLLPKMHYVRPFAVMQLDMLRHQAVNIVAARLGRAEPPLRKEVVEYMSDVDSHLWSMRRSKANFFRLMTVFSGVFAVGKWLGDICMWLNPITTVLVHVLFLMLVCFPELILPTLFLYLFLIGVWNFRYRPRYPPHMNTRISQADVVHPDEMDEEFDTFPTSKNPDLVRMRYDRLRSVAGRIQTVVGDLASQGERIHALLSWRDPRATSLFITFCLLAALVLYVTPFQMVAGLAGFYFMRHPRFRHRLPSAPINFFRRLPARTDSML

>Medtr5g096980

MALAKVDFSLKAISPITDNLGITSQTDLVEINLFLFVKIVRARNLFAHNGHNNLDPYVEVTAGRFLGRTFCLQGNTNPEWDQVFALENDQIEKEGIKTVEIFVKDNVARYDPYLGMISLEIFHIPKRFPTDSALAPKWFVLEDECKRRYRGELMMCCWIGNQADEAFHEASHLQLGHVLISARHTLNTCSRVYIMPRVWCLRLNLLQVEGLILEIDDPSESSDIFITATFGNGTRTLASKSVKSNNGNPIWNEKDILFAVAEPLDEILFLTVEQGTLARCKRLGTCVFPVKKAQTPLQNPDRLVTMDVIQNERFFVGKLSMRVTLDGGYHMFDDDPRYSTDVNPTDNGVWRPNIGVFEMGILNATGLPEMKPQGRTDAYCVAKYGSKWVRSRTVVNSLSPKWNEQYSWKVYDPSTFFIISVFDNSQLHEEYIAAGANDTRIGKVRISLSEMEINTVYNYSYPLVQLQPSGLKKMGEIQLSFKFTSPSKANLYKKYTMPMLFPQHFEDPLSQAQLYGLRQQTIELVRSNMSKAEPPLRNEVVDYMLDSREIVWSMRRCKADFERINVFLNCLVGIYTYFDDVRKWKDLVSPIIAHLLLVVLFFLPQSLLPAIFLALIVHMLQEFQIKPKTLSHADLHLSHVHTASEDELQEEFDPMPSKFEDIILMHRYDRLRVSAGRVVTQMGEFAATMERLQSLLSFQDSTATMLVMISCLIIGIVALAVPFRYLVFVWFLYFLRHPMFRSPFPPFYENWIRRMPSKLDSMI

>Medtr6g027540

MTTTEVVSVRKLIVEVINAKNLMPKDGQGTASAYAIVDFDGQRKRTKTKSRDLNPQWDEKLEFLVLDQESMTSETMEINLYNDKKTTGKRSTFLGKVKISGSTFVKSGEEVIVYYPLEKRSVFSQIKGELGLKISYVDEILPVTDSAGDDKKEEKVEEKSPVTESAGDGEKKEENPPVTDSAGDEKKKEENTKEEEKPKEETPAEKTPAPGNPPENPPATPPAPEVVNPPVAETKDVKIKEKQYEIVQKRADVINVSDHELRSLSRDRSRSVAYDLVDRMPFLYVRVVKAKRCESKSESVKLFSKLVIGTHSVRTKSENEGKDWDQVFAFDKEGLNSTSLEVSVWSESESESENKEKQITEISLGTVSFDLQEVPKRVPPDSPLAPQWYTLESENSPGNDVMIAVWIGTQADEAFQESWQSDSGGLIPETRAKVYLSPKLWYLRLTVIQTQDLQLGLAGSGSEHKVRSPELFVKAQLGAQVFKTGRTGLVSSGNPTWNEDLVFVASEPFEPFLVITLEDVSNSRSIGKTKIHVASMERRLDDRTDVKSRWFNLCGSEENLSYTGRIHIRACLEGGYHVIDEAAHVTSDVRASAKQLMKPPIGLLEVGIRGATNLLPVKTKDGTRGTTDAYVVAKYGPKWVRTRTIVDRFNPRWNEQYTWDVYDPCTVLTIGVFDNGRFQKDARDVRMGKIRVRLSTLDTNRVYVNSYSLIVLLPGGARRMGEIEIAVRFSCSSWLSLMQAYTSPILPRMHYVKPFGPGQQDVLRQTAMKIVTARLARSEPALGSEVVQFMLDSDTHVWSMRKSKANWFRLVGFLSRATTVFYWLDGIRTWVNPATTVLVHALLIAIVFCPYLILPTVFMYAFLILILRFRNRMRVPKNMDPRMSYVDMVSLDELDEEFDGFPTMRSVEVVRIRYDRVRALAGRAQSLIGDVAAQGERLEALFSWRDPRATAMFAVFCLVMSLVFYAVPFKGFVLLAGFYYLRHPRFRGDMPTVFVNFFRRLPSFSDQIM

>Medtr6g028050

MATVRKLIVEVIDAQNLAPKDGHGTSSPYIVIDFHGQRRKTRTLVRDLNPVWNETLSFNVGERNEILGDVLELDVYHEMKHSPTRRENSLGQVRLSSTQFVKKGEEALIYYELKKKSLFNMVQGKVGLKIYYVDEEIPPPPPPVPVPENPPAPSSEPPSGKVEESPPPPSELEKVEPPPSDQPSGAPPPPSEAGPQPKAEGEQEPKVEPEPVPEQIPVQEEVVDPMDANPPEAFEMAAASISRSNSEVRFSGINGPHPQPIRRSASTASFTSEASMDSMLIERSTFDLVEKMHYLFIRVVKARYLPTNGNPIVKISVSGHDVNSKPARKTTTFEWNQTFAFARDTHDSSPILEITVWDPQTIEENRSLLGGVCFDVNEIPVRDPPDSSLAPQWYRMEGGGAQHGDLMIATWIGTQADESFADAWKSDTTNHVNSKAKVYQSPKLWYLRVTILEAQDITPLTPTLKESWFHFQIRAQIGFQVLKTKTTVTKNGIVSWNEDLLFVAAEPLTVSDFIVFSLENRQHKAPVTMGVVKIPLTAVERRVDDRNVGSRWFTFDDPNDEKRSGYKGRLHLRLCFDGGYHVMDEAAHVTSDYRPTARQLWKPPVGTIELGIIGCKNLIPMKTVNGKSSTDGYCVAKYGNKWVRTRTVSDNLEPKWNEQYTWKVFDPSTVLTIGVFDSFSVFESDNSKTEMTNESTRPDFRIGKVRIRISTLQTGRVYKNTYPLLVLTHGGLKKMGEIEIAIRFVRTVQRLDFLHVYSQPMLPLMHHIKPLGVVHQEVLRNTAVKMVAGHLSRSEPPLRKEVVFYMLDADSHNFSIRKVRANWCRIINVVAGLIEIVRWIEDTRGWKNPTATILVHALLVMLVWFPDLIIPTLAFYVFAVGAWNYRFRARDPLPHFDPKISLADVVDREELDEEFDIVPSTRSYEAVRARYDKLRTLGARVQTVLGDLATQGERVQALVTWRDPCATGIFVFLCLVVAMILYLVPSKMVAMACGFYYLRHPIFRDRLPSPGLNFFRRLPSLSDRIM

>Medtr7g076900

MSSLKLCVEVVGAHDLVAKDGEGSSTTFVELEFDDQKFRTTTKDKDLSPYWNEIFYFNITDPSKLSNLNLEACINHYNKTNGSKIPLGKVKLTGTSFVPHSDAVVLHYPLEKKGIFSRTKGELGLKVFITNNPSLRASNPLPAMQEPFVNNGFMNTDQNLAQDQIPVPASFTNQILNNVLKKKNESRHTFHNLPKSNDGKEKKSNVTVGMHEMKSGPSAPKVVKAFAGTAASAMDYVIKETNPSLGGGKVVGGRILRGSNNSPSSTYDLVEPMDYLFIRVVKARDLPRMDLTGSLDPYVIVKVGNFKGTTNHFEKNNSPEWNLVFAFAKENQQATTLEVVIKDKDTIHDDFVGTVRFDLYDVPKRVPPDSPLAPQWYRIVNKKGEMMNTGEIMLAVWHGTQADEAFPDAWHSDSMSPNESFSANYAQIRSKVYTSPRLWYLRVKVIEAHDLVSHDNKSRAPDAFVKVQHGNQIFKTKPVQSRINNPRWDQGTLFVAAEPFEEPLIITVEDKDETIGNIVIPLSTIEKRVDDRKVRSRWYPLAKSMSSAMEAEERKIKEKNKDKDKFASRIHIDVFLDGGYHVLDESTYYSSDLRPTSRQLWKKAIGVLELGILNADVQPTKTRDGRGAADVYCVAKYGHKWVRTRTIVGSLSPKFHEQYYWEVYDPSTVLTLGVFNNGQLNDSNDSNDSKIGKVRIRLSTLETGRIYTHNYPLLSLQGSGLKKMGEVHLAIRFSCTSMMNMINLYFKPHLPKMHYTKPLNIFEQEKLKFQAMIIVQARLGRTEPPLRKEVVGYMSDTDSHLWSMRKSKANINRLKEVFSGLISVGSWLIEISTWKNSVTTVLVHILYMMLVCFPQLILPTMFLYMFIIGLWKWRFRPRNPPHMNTSLSCTDVTTPDELDEEFDTFPTKKSQDIVRWRYDRLRSLAGRVQSVVGDIATQGERLHALLNWRDPRATYIFMAFSFVAAIVLYLIPTQLVFLSAGFYLMRHPKLRGKLPSAPVNFFRRLPALTDSML

>Medtr7g092770

MQRPPPEDFLLKETKPHLGGKVSGDKLTSTYDLVEQMQYLYVRVVKAKELPSKDVTGSCDPYVEVKLGNYKGTTRHFEKKTNPEWNQVFAFSKDRIQASVLEVFVKDKDFVKDDFIGRVWFDLNEIPKRVPPDSPLAPQWYRLEDRKSDKVKGELMLAVWMGTQADEAFPEAWHSDAATVSGTDALANIRSKVYLSPKLWYLRVNVIEAQDLQPSDKGRFPEVYVKAILGNQTLRTRISQSRSINPMWNEDLMFVAAEPFEEPLILSVEDRVAPNKEELLGKCVIPLQMMDRRLDHKPVNTRWFNIEKHVVIMEGDKKKEIKFASRIHMRVCLEGGYHVLDESTHYSSDLRPTAKQLWKSSIGVLEVGILNASGLMPMKSNNGRGTTDAYCVAKYGQKWVRTRTIIDSFAPRWNEQYTWEVFDPCTVITIGVFDNCHLHHGGDKPGGQRDSKIGKVRIRLSTLETDRVYTHSYPLLVLHPTGVKKMGEIQLAVRFTCSSLLNMMHMYSNPLLPKMHYIHPLTVSQLDSLRHQATQIVSMRLSRAEPPLRKEVVEYMLDVGSHMWSMRRSKANFFRIMGVLSGLIAVGKWFDQICNWKNPITTVLIHILFIILVMYPELILPTIFLYLFLIGVWYYRWRPRHPPHMDTRLSHADSAHPDELDEEFDTFPTTRPSDIVRMRYDRLRSIAGRIQTVVGDLATQGERLQSLLSWRDPRATALFVLFCLIAAIVLYVTPFQVVALLSGIYVLRHPRFRHKLPSVPLNFFRRLPARTDCML

>Medtr8g031270

MSNLKLGVEVVGAHDLMPKDGEGSASAFVELHFDDQKFRTTTKEKDLNPVWNEKFYFTIADPSKLPSLALDACVYHYNSKNNNPKIFLGKVRLTETSFVPLSDAVVLHYPLEKKITFSRVKGELGLKVFVTEDPSVRSTNVFPDQKPSMDSDQHSNKDQPPVSLTDSFLNMFSRKKNVPKHSFHSIPGSNQEEHKSSPPVAAKMDVDHVKHGMKSGPPQKIMHAYADSLSPFDYALKETSPSLGGGQVIGGRVIRGNKPSSTYDLVEPMRYLFVRVTRARDLPSKTGSLNPYVQVKAGNFKGTTKHLEKNQEPEWNEVFAFSRDNLQSTTLEVEVKDKGTILDETVGTVRFVLHDVPTRVPPDSPLAPEWYQIEKSGKKKKGELMLAVWFGTQADEAFPDAWHSDTLFPGGNSSVSHHQMRSKVYHSPRLWYVRVRVIEAQDLILSEKSQMSDAYVKVQTGNQILKTKPVQSRTKNMRWDQELMFVAAEPFDEPLILSIENRIGPNKDETIGAVVIPLTKVEKRADDRIIRTRWYNLEQSMSSAMDGEQGKMNDVFSSRIHLSVCLDGGYHVFDESTYHSSDLRPTSRQLWKKPIGVLELGILNVDGLHPMKARDGRGTSDAYCVAKYGRKWVRTRTLSNTLDPKYNEQYTWEVFDPATVLTVGVFDNGQVNGPDNKDLLIGKVRVRISTLETGRVYTNSYPLLMLHPSGVKKMGELHLAIRFSCYSMVDLMQLYFKPHLPKMHYKRPLNVMEQEMLRQQAVNVVASRLSRAEPPLRKEVVEYMSDTHSHLWSMRRSKANFYRLMTVFSGFLSVGRWLGEVSTWNHPMTTVLVHILFVMLVCFPELIMPTMFLYVFVIGMWNWRFRPRCPPHMNTRLSYTDGVTPDELDEEFDTFPSTKNPDVVRWRYDRLRSVAGRVQSVVGDLATQGERVQALVSWRDPRASSMFMAFCFVSAIVLYITPFQMPILMGGFYFMRHPMFRSKVPAAPVNFYRRLPALTDSML

>Potri.001G015700

MQKLPQSVDFALKETSPNIGAGSVTGNKLSCTYDLVEQMQYLYVRVVKARDLPPKDVTGSCDPYVEVKLGNYKGVTKHFEKKSNPEWNQVFAFSKDRIQASVLEVFVKDKDVVLDDLIGWMMFDLNEVPKRVPPDSPLAPQWYRLEDRKGGKIKSGELMLAVWMGTQADEAFPDAWHSDAASVGPDGVNNIRSKVYLSPKLWYVRVNVIEAQDLVPSDKSRFPEVFVKGTLGNQALRTRTSHIKTINPMWDDDLIFVAPEPFEEPLILTVEDRLGPNKDEVLGKCVIPLQLVQRRLDHKPVNTRWFNLEKHVVLDGELKKETKFSSRIHVRICLDGGYHVLDESTHYSSDLRPTAKQLWRPSIGILELGVLSAVGLMPMKMKDGRGTTDAYCVAKYGQKWVRTRTIVDSFTPRWNEQYTWEVFDPCTVITVGVFDNGHLHGGGGGKDSRIGKVRIRLSTLETDRVYTHSYPLLVLHPAGVKKTGEVQLAVRFTCSSLVNMLHMYSHPLLPKMHYIQPLSVMQLDSLRHQAMQIVSMRLSRAEPPLRKEVVEYMLDVDLHKWSMRRSKANFFRIMGVLSGLIAVGKWFDQICNWKNPLTTILIHLLFIILVLYPELILPTVFLYLFVIGLWNFRWRPRHPPHMDTRLSHADAAHPDELDEEFDTFPTSRPSDIVRMRYDRLRSIAGRVQTVVGDLATQGERFQSLLSWRDPRATTLFVTFCLIAAIVLYVTPFQVVGLLIGIYVLRHPRFRHKLPSVPLNFFRRLPARSDSML

>Potri.001G105400

MMSNIKLGVEVVSAHNLLPKDEHGSSSAFVELDFDGQRFRTTIKEKDLHPVWNESFYFNVSDPSNLHYLTLDAHVYCNIRATNSRSFLGKVCLTGNSFVLHSDAVVLHYPLEKRGIFSRVRGELGLKVYITDDASIKSSTPLPAVESLPTKDPGLTHTEAPVVHPMTNSVPHKRVERHTFHHLPNPNHQQNQHQNHSSAPAISHHVPKYVADEMKAAETQPPKLVRMYSASSSQPVDYALKETSPFLGGGRVVGGRVIHGDKTASTYDLVERMYFLYVRVVKARDLPAMDVTGSLDPFVEVRIGNYRGITKHFEKKQNPEWNQVFAFSRERMQASVLEVVIKDKDLVKDDFVGVIRFDINEVPLRVPPDSPLAPEWYRLEDKKGEKIKGELMLAVWIGTQADEAFPDAWHSDAATPVDSTPASSTVIRSKVYHAPRLWYVRVNVVEAQDLVPSEKNRFPEVYVKVQIGNQVLKTKTYQARTFSALWNEDLLFVAAEPFEDHLVLSVEDRVGPGKDEIIGRVIIPLSSVEKRADDRIIHSCWFNLEKPVAVDVDQLKKDKFSSRIHLRVCLDGGYHVLDESTHYSSDLRPTAKQLWRPPIGMLELGILNAVGLHPMKTRDGRGTSDTYCVAKYGHKWVRTRTLIDNLSPKYNEQYTWEVFDPATVLTVGVFDNNQLGEKGSSGKDLKIGKVRIRISTLETGRVYTHSYPLLVLHPTGVKKMGELHLAIRFTCISFANMLYQYSRPLLPKMHYIRPFTVMQLDMLRHQAVNIVALRLGRAEPPLRKEVVEYMSDVDAHLWSMRRSKANFFRLMTIFSGLFAAGKWFGDICMWKNPITTVLVHVLYLMLACFPELILPTVFLYMFLIGIWNYRYRPRYPPHMNTKISQAEVVHPDELDEEFDTFPTSRSPELVRMRYDRLRSVSGRIQTVVGDIATQGERFQALLSWRDPRATAIFVIFCLVAALVLFVTPFQVIAALAGFYMMRHPRFRYRTPSVPINFFRRLPSRTDSML

>Potri.001G271400

MKLVVEIVDAHDLMPKDGKGSASPFVEVDFQNQLSKTKTIPKNLNPVWNQKLLFDLDETKNRHHQSIEVSVYNERRPIPGRNFLGRTRIPCSNVVKKGDEVYQTFQLEKKWFFSTVKGEIGLKIYTSLESKAPPLPSPSQPPPSNIPPETSASSSSLPTITHIAENTGRDCRTLAALPRAEILHTSEAITEQPGKKISAISETSGGFPAKEPKNSNKEPTKIRADTTQHVHKHQVLQKTSQSVEKLPNGAPYTMHAANPSAHSSDLDDFNLKDTDPQLGERWPSGGAYGGRGWMNGERYASTYDLVEQVSYLYVRIVKAKDLPSSSITASCDPYVEVKLGNYKGRTRHFEKKMNPEWNQVFAFSKDRIQSSVLEVFVKDKEMVGRDDYLGRVVFDLNEVPTRVPPDSPLAPQWYRLEDRRGEGKVRGEIMLAVWMGTQADEAFPDAWHSDAASVYGEGVLNIRSKVYVSPKLWYLRVNVIEAQDVVPSDRSRLPEVFVKVQVGNQVLRTKIHPTRTANPLWNEDLVFVVAEPFEEQLFLTVEDRLTPLKDDVLGKISVPLNIFEKRLDHRPVHSRWFNLEKYGFGVLEADRRKELKFSSRIHLRVCLEGGYHVMDESTMYISDQRPTARQLWKQPVGILEVGILGAQGLLPMKMKDGRGSTDAYCVAKYGQKWVRTRTIVDTFNPKWNEQYTWEVYDPCTVITLGVFDNCHLGGGEKPTAANAARDLRIGKVRIRLSTLEAYRTYTHSYPLLVLHPLGVKKMGELQLAVRFTTLSLANMIYVYGHPLLPKMHYLHPFTVNQVDNLRYQAMNIVAVRLGRAEPPLRKEVVEYMLDVDSHTWSMRRSKANFFRIMSLVSGLFSMSHWFGDICQWRNPITSVLVHILFLILIWYPELILPTLFLYMFLIGIWNYRFRPRHPPHMDTKLSWAEAVHPDELDEEFDTFPTSKSHDIVRMRYDRLRGVAGRIQTVVGDIATQGERFQSLLSWRDPRATSLFIVFCLCAAVVLYVTPFRVVALVAGLYYLRHPRFRSKLPSVPSNFFKRLPARTDSLL

>Potri.002G158000

MSNLKLGVEVVGAHDLMAKDGQGSASAFVELHFDQQKFRTTIKDKDLSPVWNENFYFNISDPSSLSNLTLEAHVYHHKREKNSKSSLGKVRLTGTSFVPYSDAIVLHYPLEKQGILSRVKGELGLKVFVTNDPSIRSSNPLPAMESSLFSDSRATQAQAPEQQTPNVAQKVFSDGKSESRHTFHHLPNPSQSQKQQHAPPAATQPSVDYGIREMKSEPQAPRVVRMFPGLSAQPVDYTPKETSPFLGGGQIVGGRVIRGDRPASTYDLVEQMKYLFVRVVKARDLPTMDVTGSLDPYVEVKVGNYKGTTKHFEKKQNPEWNEVFAFARDRMQSSVLEVVVKDKDLIKDDFVGIVRFDLHEVPTRVPPDSPLASEWYRLEDKKGEKSKAELMLAVWYGTQADEAFPDAWHSDAISPDSSSIISTLIRSKVYHSPRLWYVRVNVIEAQDLVASDKSRFPDAYVKVQIGNQVLKTKMVQSRTLSPVWNEDLLFVAAEPFDDHLILSVEDRTGPNKDESIGKVVIPLNTVEKRADDRMIRSRWFGLEKSVSASMDEHQSKKDKFSSRLHLRVVLDGGYHVLDESTHYSSDLRPTAKQLWRPSIGVLELGILNADGLHPMKTREGKGTSDTYCVVKYGQKWVRTRTIINSLSPKYNEQYTWEVYDPATVLIVGVFDNNHLGGSNGNKDTKIGKVRIRLSTLETGRVYTHSYPLLVLHPSGVKKMGEIHLAIRFSYTSFPNMMFQYSRPLLPKMHYVRPLTVMQQDMLRFQAVNLVAARLGRAEPPLRKEVVEYMSDADSHLWSMRRSKANFFRLMSVFSGLLSVGKWFGEVCMWKNPITTVLVQVLFVMLVCFPELILTTVFLYMFLIGVWNYHSRPRYPPHMSTRISYADAVSPDELDEEFDTFPSRVSPEVVRFRYDRLRSVAGRIQTVVGDMATQGERVQALLSWRDPRATTIFLIFCLVVAIVLYATPFQVLALLGGFYFMRHPRFRHRVPSAPVNFFRRLPARTDSML

>Potri.002G254400

MAMTSKEKLVVEVVAAHNLMPKDGEGSSSPFVEVEFENQRLRTQVKYKDLNPIWNQKLVFHIKDVADLSYRAIEVNVFNERRSSNSRNFLGKVRVSGSSVAKQGEEVVQLHTLDKRSLFSHIRGEISLKLYVSTREEVKEVGGFGNGEVVSSTPGSSNSSKKNKKTQQQNPLILQQPQQLSKEVINNNKQAQEQGQNNINAKSVETNPGGIKPVVTTTALGPGSLVSSSGGGIVGPAGGAGLGGISVHSNGSSEFSLKETSPHLGGGRLNKDKTSSTYDLVELMQYLYVRVVKAKYNMLFGGGEVVAEVKLGNYRGVTKKVIGSSNVEWDQVFAFSKDCIQSSMVEVFVKQGNKDDYLGRVWFDLNEVPRRVPPDSQLAPQWYRMEDKKGDKSKGGELMVSIWFGTQADEAFAEAWHSKAANVHFEGHCSIKSKVYLSPKLWYLRVAVIEAQDIVPGEKGLGMMRFPELFVKVQVGNQILRTKIAGPNPNRSMINPYWSEELMFVVAEPFEDFLFLSVEDRVGPGREEAVGRVMLPVAAIERRHDDKQVVSRWFNLDNQFGSAVESKLVTRFGSKIHLRLSLDGGYHVLDESTMYSSDVRPTAKQLWKPHIGVLEMGILGATGLMPTKLKEGKRESIDAYCVAKYGQKWVRTRTVVDSFSPKWNEQYTWEVFDPCTVITVGVFDNCRTDKNVFNNTGARDSRIGKVRVRLSTLESDRVYTHSYPLLVLHTTGVKKMGELHLAVRFSCANMANMLHMYTLPLLPQMHYVHPLSVNQLDAMRYQAMNVVASRLSRAEPPLGREVVEYMLDHDSHMWSMRRSKANFARLISVLSVFVAMARWVESMRNWHKPVYSTLFVLAFLLWVAMPELIIPSLLLYMAFVGLWRYRTRPRHPPHMDTKLSHVVSVYSDELDEEFDSFPTSRSAETVRMRYDRLRSVAGRIQTVVGDMASQGERFQALLGWRDPRATFLFVVMCLFAAVGFYAVAIRVVVALWGLYVMRPPKFRNKLPPRALSFFRRLPTKADSLL

>Potri.003G125900

MMSNLKLGVEVVSAHNLLPKDEHGSSSAFVELCFDGQRFRTTIKEKDPNPVWSECFYFNIPDPSNLHYLTLDAHVYNNIRATNSRYFLGKVCLTGNSFVPYSDAVVLHYPLEKRGIFSRVRGELGLKVYITDDASIKSSTPLPAVESLPTKDPGLTHAVAPMVDPMTNTVSHKRVERHTFHHLPNPNHQQQQHQNHSSAPSITHHVPKYVADEMKAAETQPPKLVRMHSASSSQPVDHALKETSPFLGGGRVVGGRVIRGDKTASTYDLVERMYFLYVRVVKARDLPAMDVTGSLDPFVEVRVGNYGGITKHFEKKQNPEWNQVFAFSRERMQASVLEVVIKDKDLVKDDFVGVIRFDINEVPSRVPPDSPLAPEWYRLEDKKGEKIKGELMLAVWIGTQADETFPDAWHSDAATPVDNTPATSTVTRSKVYHAPRLWYVRVNVVEAQDLVPSEKTRFPEVYAKVQMGNQVLKTKTCQARTFSALWNEDLLFVAAEPFEDHLVLSVEDRVGPGKDEIIGRVIIPLRSVEKRADDRIIHSRWFNLEKPVAVDVDQFKKDKFSSRIHLRACLDGGYHVLDESTHYSSDLCPTAKQLWRPPIGILELGILNAVGLHPLKTRDGRGTADTYCVAKYGHKWVRTRTLIDNPSPKYNEQYTWEVFDPATVLTVGVFDNSQLGGKGSNGKDLKIGKVRIRISTLETGRVYTHSYPLLVLHPTGVKKMGELHLAIRFTCISFANMLYQYSRPLLPKMHYIRPFNVMQLDMLRHQAVNIVALRLGRAEPPLRKEVVEYMSDVDSHLWSMRRSKANFLRLMTVFSGLFTAGKWFEDICMWKNPITTVLVHVLYLMLACFPELILPTVFLYMFLIGIWNYRYRPRYPPHMNTKISQAEAVHPDELDEEFDTFPTSRSPELVGMRYDRLRSVAGRIQTVIGDIATQGERFQALLSWRDPRATAIFVIFCLVAALVLFVTPFQVIAALAGFYMMRHPRFRYRTPSVPINFFRRLPARTDSML

>Potri.003G210800

MQKPPQSVDFALKETSPNIGAGSVTGDKLSCTYDLVEQMQYLYVRVVKAKDLPPKDITGSCDPYVEVKLGNYKGVTKHFEKKSNPEWNQVFAFSKDRIQASVLEVFVKDKDVVLDDLIGRMMFDLIDVPKRVPPDSPLAPQWYRLEDRKGDKIKAGELMLAVWMGTQADEAFPDAWHSDAASVGPDGVNKIRSKVYISPKLWYVRVNVIEAQDLVPGDKSRFPEVFVRGTLGNQALRTRTSQTKTVNPMWNEDLIFVVAEPFEEPLILTAEDRLGPNKDEVLGKCVIPLQLVQRRLDHKPVNTRWFNLEKHVIVDGEQKKETKFASRIHLRICLDGGYHVLDESTHYSSDLRPTAKQLWRSSIGILELGVLSAVGLMPMKKKDDRGTTDAYCVAKYGQKWIRTRTIVDSFAPRWNEQYTWEVFDPCTVITIGVFDNGHIHSGGGGKDSRIGKVRIRLSTLETDRVYTHSYPLLAIQSSGVRKTGEVQLAVRFTCSSLVNMLHMYSHPLLPKMHYVHPLSVMQLDSLRHQAMHIVSMRLSRSEPPLRKEVVEYMLDVDSHMWSMRRSKANFFRIMAVLSGLIAVGKWFDQICNWKNSLTTILIHILFIILVLYPELILPTIFLYLFLIGLWNYRWRPRHPPHMDTRLSHADAAHPDELDEEFDSFPTSRPSDIVRMRYDRLRSIAGRVQTVVGDLATQGERFQSLISWRDPRATTLFVTFCLIAAIVLYVTPFQVLALLIGLYVLRHPRFRHKLPSVPLNFFRRLPARSDSML

>Potri.004G043000

MPPKEKPRVDYTLKATSPDIGGRKATGSDKLTLVEQRQFIYVRIVKANGLPMNNISGTCNPFVELKIGNYKGITRCFEQTSNPEWNEVYAFTRDQILGGRLEILVRDKESAINEITGHLSFDLGHIPTRFPPDSPLAPQWYKLEDRNGVKIVGELMLAVWIGNQADDAFPVAWHSDAAAVSGKSVTKTRSNVYLSPVLWYLRIQVIAAQDLAPADRNRKPEAYVKAVLGNLVLRTKVSKDTNLNPTWNEEVMFVAAEPFDDPLVLSVEDKMGADKDVCLGRSVIPLHQVEKRLLPQPIGDQWITLQKHVAEGEKKTEVKFAGRLHLRIFLDGVYHVFDEPTYYCSDLRATSPKLWPEKIGVLELGILKAEGLLPTKSKDGRGTTDAYCVAKYGQKWVRTRTIVDSFAPKWNEQYHWDVYDPYTVVTIGVFHNYHLQEGDKNGGKRDPRLGKVRIRLSTLETGRIYTHSYPLLVLQPNGLKKMGELHLAVKFSCNNWIDLFHTYSQPLLPMMHYLKPLSVYQLDSLRHQATYTLSLRLGRADPPLSREVVEYMLDTGVNRWSLRRGKANCERVMACLSGIVFIWGQFDQIRHWKNSAVTILIYSLFVAMVMSPKLILPAFFLAFFVLGVWRFPKRPRHPPHMDTKLSHAETAQHDELDEEFDTFPTSKQGEALKTRYDRLRGIAGRLMIMIGDLATQLERIHALVSWRDPRATAMFLIFCLIACILVHKVQFRYLVLVTWTYAMRPPRLRVGIPSIPQSFLRRLPAKTDSML

>Potri.006G058700

MNPLAAPDHKDDFKLKDTKPQLGERWPHGGPRGGGGWISSERATSTYDLVEQMFYLYVRVVKAKDLPTNPVTGSCDPYIEVKVGNYKGETQHFEKKTNPEWKQVFAFSKERIQSSVVEVILRDRERVKRDDHVGKVVFDMHEVPTRVPPDSPLAPQWYRLEALHGDNKVKGEVMLAVWMGTQADEAFPEAWHSDAASVHREGVLNIRSKVYVSPKLWYLRVNVIEAQDVEPLDRSQLPQVFVKAQVGNQILKTKLCPTRTTNPMWNEDLIFVAAEPFEEQLILTVENKASPAKDEVVGRVDLPLQIFERRLDYRPVHSKWFNLERFGFGALEGDKGHELKFSVRLHLRVCLEGAYHVLDESTMYISDQRPTAWQLWKQPIGILEVGVLSAQGLLPMKTKEGRGTTDAYCVAKYGLKWVRTRTIIENFNPKWNEQYTWEVYDPSTVITFGVFDNCHLGGGEKPATGGGARIDSRIGKVRIRLSTLETDRIYTNSYPLLVLQPSGLKKMGELQLAVRFTCLSLANMIYLYGHPMLPKMHYLHPFTVNQLDSLRYQAMNIVAVRLGRAEPPLRKEIVEYMLDVDSHMWSMRRSKANFFRIVSLFSGVISISKWLGEVCKWKNPVTTVLVHVLFFILVCYPELILPTIFLYMFLIGIWNYRLRPRHPPHMDTKLSWAEAVHPDELDEEFDTFPTSKQQDVARMRYDRLRSVAGRIQTVMGDMATQGERFQALLSWRDPRATSLFVIFCLIAAVVLYVTPFKIITLVTGLFWLRHPRFRSKQPSVPSNFFRRLPSRADSML

>Potri.006G058900

MMQKPPQDDFLLKETNPHLGGGKITGDKLTSTYDLVEQMQYLYVRVVKAKDLPAKDVTGSCDPYVEVKLGNYKGTTRHFEKKTNPEWNQVFAFSKERIQASMLEVTVKDKDLVKDDFIGRVLFDMNEIPKRVPPDSPLAPQWYRLEDRKGDKFKGELMLAVWMGTQADEAFPEAWHSDAATVSGTDSLANIRSKVYLSPKLWYLRVNVIEAQDLVPSDQGRYPEVYVKAILGNQVLRTRVSPSRSINPMWNEDLMFVASEPFEEPLILSVEDRIAPNKDEVLGRCAIPMHHVDRRLDHNPVNTRWFNLEKHVIVEGEKKKEIKFASRIHMRICLEGGYHVLDESTHYSSDLRPTAKQLWKHSIGVLELGILNAQGLMPMKPKDGRGTTDAYCVAKYGQKWVRTRTIIDSFTPKWNEQYTWEVFDPCTVITIGVFDNCHLHGGDKPGGSRDSRIGKVRIRLSTLETDRVYTHSYPLLVLHRNGVKKMGEIHLAVRFTCSSLLNMMHMYSHPLLPKMHYIHPLTVSQLDSLRHQATVIVSVRLSRSEPPLRKEIVEYMLDVGSHMWSMRRSKANFFRIMNVFGGLIALGKWFDQICNWKNPITTVLIHILFIILVLYPELILPTIFLYLFLIGVWHYRRRSRHPPHMDTRLSHAESAHPDELDEEFDTFPTSQSADIVRMRYDRLRSIAGRIQTVVGDLATQGERLQSLLSWRDPRATALFVLFCLIAAIVLYITPFQVVAVLIGLYVLRHPRFRHKLPSVPLNFFRRLPARTDSML

>Potri.006G098700

MTEPPKTVRKVLVEVVDARDLLPKDGQGSSSAYVIADFDGQRKRTTTKYRDLNPVWKETFEFTVSDPSNMEFEELEIEVFNDKKFCNGSGRKNHFLGRVKVYGSQFSKRGDEGIVYFPLEKKSVFSWIRGEIGLRICYYDELLEEDQQQPPPPPEKDAPPPQQQDPQKSPAVTMVEEVRVFQVAEHAEFNYHDYHHHQNDHHQQHQNGTHSPPVAIEESPPPVVHVRMMQTTRESSGNNRVKIMRRPNGDFTPKVISGRFKSEPTERILPYDLVEPMQYLFIRIVKARGLSQNESPFIKLRTSTHFVRSKPASYRPGDSPGSFEWHQVFALGHNNKTDVQSSDAGIIEISVWDSQSEQFLGGVCLDLSDVPVRDPPDSPLAPQWYRLESGAAADQNSCRVSGDIQLSVWIGTQADDAFPEAWSSDAPYVAHTRSKVYQSPKLWYLRVTVIEAQDLRIASNLPPLTAPEIRVKAQLGFQSAKTRRGSMSNHSTSFQWIEDLIFVAGEPLEESLILLVEDRTNKEALLLGHIIIPVSSIEQRIDERHVASKWFALEGGGDTGGGGGGVNGGSYRGRIHLRLCLEGGYHVLDEAAHVCSDFRPTAKQLWKPAIGVLELGILGARGLLPMKTKGGGKGSTDAYCVAKFGKKWVRTRTITDSFDPRWNEQYTWQVYDPCTVLTIGVFDNWHMFGDMSDDKPDCRIGKIRIRVSTLESNKVYTNAYPLLVLLRTGLKKMGEIELAVRFACPSLLPDTCAAYGQPLLPKMHYLRPLGVAQQEALRGAATRMVSLWLARSEPPLGPEVVRYMLDADSHTWSMRKSKANWFRIVAVLAWAVGLAKWLDDIRRWRNSVTTVLVHALYLVLVWYPDLVVPTGFLYVILIGVWYYRFRPKIPAGMDIRLSQAETVDPDELDEEFDTIPSMKPPEIIRARYDRLRVLAARVQTVLGDFATQGERVQALVSWRDPRATKLFIGVCLAITLILYVVPPKMVAVALGFYYLRHPMFRDPMPPASLNFFRRLPSLSDRLM

>Potri.008G007200

MPPKHDFSLREIKPNIDGGKTLTPNMLTLVEPLYFVYVKVVRASHLPLNQATYVEVKSGNYKATTKYIQGTLAPIWNQVFAFNKDRLQAKTIEISVRGKVSVTNEIIGSIEVGIGDIPTRLQGDSSLAPQWYGLEDKNGVSGRSGNLMLAIWVGNQVDDAFSLAWHLDAASVSVDKVSNARPQVYYSPRLWYLKIKVNGAQDLVVSDPNRKPEVYVKATLGNKMIKEIIWWIIWGSVLSLCIKLLRDCCLLFQGPMEKFSSKLRVTIYLDGVYHVFDEPALFSTDLKASSPKLTPGKVGDLELGILKAEGLVPMKSKNGLKTTDAYCVAKYGPKWTRTSTVVSSLEPKWMKQYQWDVLDPCTVIAIGVFDNNNLQAGDGWATDRLIGKVIRIRLSTLEFGRIYKYAYPLVALMPDGVKKMGELHFTLRFIYTKGSGDKIYQYTQPMLPKPAYTDPMSVYQIDSLRNQAVRHIAMRLARAEPPLRREVVESMLSGRGPVWSIRRGKANFQRVMECLKFLKTALIWLDDLRQWKNSRTTIVMFAAFSVFVYYSEIIIPSFFAFLFLKALHNYFKRPRDILCLDTNLSQVESVNTLDWQEELDTFPSSAPFEDLRLRYDRLRAIGYRIEETVGDLATQLERFHAIFSWRDRRATLIFTLFCLVAWIMFYLVPFRLLFFLFGTYLMRSPRFRVTLPPIPQNVFRRLPSRDDCLL

>Potri.009G065600

MKLVVEVVDAHNLMPKDCEGSASPFVEIDFQNQQSRTKTIPKNLNPVWNQKLLFDLDETKNRHHQSIEVSVYNQRRPIPGRNFLEKKWFSSTVVKGEIALKIYTSPESETKVPPLPSPPINTPQPLETSASCSPPPTITHIAENTDLDFKTLAALPKAGTLHTSKAITEQPEKKISAIAETNGGVPEKEPKKSNKEPVKIRADTTQHAHQHQILQQPSLSVEKLPNGTPCTMHPANPTAHSSDPDDFDLKDTNPKLGERWPSGGAYGGRGWMNGERYASTFDLVEQMSYLYVRVVKAKDLPPSSITASCDPYVEVKLGNYKGRTRHFEKKMNPEWNQVFAFSKERMQSLVLEVFVKDKEMVGRDDYLGRVVFDLNEVPTRVPPDSPLAPQWYRLEDRRGEGKVRGDIMLAVWMGTQADEAFPEAWHSDAASVYGEGALNIRSKVYVSPKLWYLRVNVIEAQDVVSNDRGRFPEVFVKVQVGNQVLRTKIHPTKTANPLWNEDLVFVVAEPFEEQVFLTIEDRVTPLKDDVLGKISLPLNIFEKRLDHRPVHSRWFNLEKFGFGVLEADRRRELQFSSRIHLRVCLEGGYHVLDESTMYISDQRPTARQLWKQPVGILEVGILGAQGLLPMKMKDGRGSTDAYCVAKYGQKWVRTRTILDTFNPKWNEQYTWEVYDPCTVITLGVFDNCNLRGGEKPNAANAARDSRIGKVRIRLSTLEAYRIYTHSYPLLVLHPHGLKKMGELQLAVRFTTLSLANMIYVYGHPLLPKMHYLHPFTVNQVDSLRYQAMNIVAVRLGRAEPPLRKEVVEYMLDVDSHMWSMRRSKANFFRIMSLISGLFTMNNWFVDICQWRNPITSVLVHILFLILIWYPELILPTLFLYMFLIGLWNYRFRPRHPSHMDTKLSWAEAVHPDELDEEFDTFPTSKSHDIVRMRYDRLRGVAGRIQTVVGDIATQGERFQSLLSWRDPRATSLFIVFCLCAAVVLYATPFRAVALVAGLYYLRHPRFRSKLPSVPSNFFKRLTAQTDSLL

>Potri.011G052000

MPPKEKPKKDYTLKVTSPDIGGRTVIGSDKLTLVEQRQFLYVRIVRANGLAVNNMTGTCDPFVELKIGNYKGITRCFEQTSNPEWNEVYAFTRDRLQGGRLEILVRDKESAINEIIGCLSFDLGDTPTRFPPNSPLAPQWYKLEDRNGVKVAGELMLSAWIGNQADDAFSVAWHSDAAAVSGKSVTNIRSNVYLSPVLWYLRVQVIAAQDLAPSDKNRKPEAYIKAVLGNLVLRTTVSKDKNPNPTWNEEVMFVAAEPFDDHLILSVEDKMGANKEVCLGRSVIPLHQVEKRLMPQAIGAQWINLEKYVAEGEEKTEVKFASRLHLRIFLDGLYHVFDEPTYYSSDLRATSPKLWPEKIGVLELGILKAEGLLPTKSRDGRGTTDAYCVAKYGRKWVRTSTIVDSYAPKWNEQYCWDVYDPYTVVTIGVFDNCHLQAGDKNDGTGDPRLGKVRIRLSTLETGRIYTHSYPLLVLQPNGLKKMGELHLAVKFSCNNWINLFHTYSQPLLPMMHYLQPLSVYQLDSLRHQATYILSLRLGRADPPLRREVLEYMLDTGVNRWSLRRANANCERVMTCLSGIVVLWRQFDQIRHWKINSAITVLIYSLFVAMVMCPKLILTAFFLAPFVLGVWCFPKRPRHPPHMDTKLSHAETAQPDVLDEEFDSFPSSKQGEALKTRYDRLRGISGRWMIIIGDLATQLERIHALVSWRDSRATAMFLAFCLIACFLVHKVQFKYLVLVIGTYAMRPPRLRAGIPSIPQNFLRRLPAKTDSML

>Potri.013G055500

MATKQKLIVEVVDARNLLPKDGHGSSSPYVVIDFYGQRKRTKSAIRDLNPTWNETLEFNVGKPSNVFGDMLELDVYHDKNYGPTRRINHLGRIRLSSSQFVRKGEEALIYYPLEKKYLFSWTQGEIGLRIYYQDEVTPPPPPPPQPAAAREEEAKADTNQESSPPQPTAEAAAPAESEATQPAEAQKSDVETGATTESNKEQPAEEAKSNEEPPAQAEAAAAPPPSDNAPAPIQPAQEDGDGIVLEPTFRKWGPAPPEIVAASTGSFPEIKVSGINAPQPIIRPVAPTSNYTLEPQESISIERSAFDLVEKMHYLFVRVVKARYLPTSGNPVVRIEVSNSRVQSKPARKTLCFEWDQTFAFGRDAPDSSSIVEISVWDPHDPKSSEMAAAANFLGGICFDVTEIPLRDPPDSPLAPQWYRLEGGGAYRSDLMLATWVGTQADDSFPDAWKTDTAGNINSRAKVYLSPKLWYLRATVLEAQDIFPLMPLKETAVQVKAQLGFQVQKTKTSVSRNGTPSWNEDLLFVAAEPCSDQLIFTLENRQPKGPVTIGMVRIALSATERRVDDRKVASRWFSLEDPRSEKAGYRGRVQLRLCFDGGYHVMDEAAHMSSDYRPTARQLWKQPVGTFELGIIGCKNLSPMKTVDGKGCTDAYCVAKYGPKWVRTRTVCDSLDPKWNEQYTWKVYDPCTVLTIGVFDSSGVYEIDGDKTATRPDFRMGKVRVRLSTLETGKVYRNRYPLILLTNNGVKKMGEIEVAVKFVRATPTLDFLHVYTQPLLPLMHHLKPLGVVQQELLRNSAVKIIATHLSRSEPSLRREVVSYMLDVDTHAFSMRKIRANWIRIINVIASVIDIVRWIDDTRVWKNPTSTVLVHALLIMLVWFPDLIVPTLAFYVFVIGAWNYRFRSRAPLPHFDPKLSLADSADRDELDEEFDPLPSSRPPEMVRTRYDKMRMLGARVQTVLGDFATQGERLQALVTWRDPRATGIFVGLCFVVAMILYMVPSKMVAMASGFYVFRHPIFRDRMPSPALNFFRRLPSLSDRIM

>Potri.013G066400

MAETCTRKLIVEVCNARSLMPKDGQGTASAFATVDFDGQRRRTKTKLRDLNPEWDEKLEFLVHDTDSMATETLEISLYNDKKTGKRSTFLGKVRIAGSAFVKSGGETLVYYPLEKRSVFSQIKGELGLKVYYIDEDPPAPPAEQKPEEKAPETEENKPAEEAKPEEEKKEEEKKEEPKTESNKEAKKEEEKPSPPPQEENPKKPEEAAPPVKVENPPLAESEKKPSKEEKEKAEIVKRSEVTISDLELRSLASDRGRSAYDLVDRMPFLYVRVVKAKTANNESKSPVYAKLMIGTHSIKTKSQSDKDWDKVFAFDKEGLNSTSLEVSVWTEEKKENEETTQECSLGTVSFDLQEVPKRVPPDSPLAPQWYALESESSAGNEVMLAVWIGTQADEAFQEAWQSDSGGLLPETRAKVYLSPKLWYLRLTVIQTQDLHLGSGSEAKVRNPELYVKAQLGAQLFKTGRTSVGSTSASSANPTWNEDLVFVAAEPFEPFLTVTVEDVTNGQSVGHAKIHVASIERRTDDRTELKSRWFNLVGDDTKPYTGRIHVRVCLEGGYHVLDEAAHVTSDVRAAAKQLAKAPIGLLEVGIRGATNLLPVKTKDGTRGTTDAYVVAKYGPKWVRTRTILDQFNPRWNEQYTWDVNDPCTVLTIGVFDNGRYKHDEAAEKQGKDVRVGKVRIRLSTLDTNRVYFNQYSLTVVLPSGAKKMGEIEIAIRFSCSSWLSLIQAYTSPMLPRMHYVKPMGPTQQDILRQTAMRLVTTRLTRSEPPLGQEVVQFMLDSDTHMWSMRRSKANWFRVVGCLTRVATLARWTEGIRTWVHPPTSVLMHVLLVAVVLCPHLVLPTIFMYAFLILAFRFRYRQRVPLNMDSRLSYVDMVGPDELDEEFDGFPTTRSQDVVRIRYDRLRALAGRAQTLLGDFAAHGERLEALWNWRDPRATGIFVVFCLVASLVFYVVPFKVFVLGFGFYYLRHPRFRDDMPSIPVSFFRRLPSFSDQIL

>Potri.013G066500

MAETCTRKLIVEVCNARNLMPKDGQGTASAFATVDFDGQRRRTKTKLRDLNPEWDEKLEFLVHDTDSMATETLEISLYNDKKTGKRSTFLGKVRIAGSAFVKSGGETLVYYPLEKRSVFSQIKGELGLKVYYIDEDPPAPPAEQKPEEKAPETEENKPAEEAKPEEEKKEEEKKEEPKTESNKEAKKEEEKPSPPPQEENPKKPEEAAPPVKVENPPLAESEKKPSKEEKEKAEIVKRSEVTISDLELRSLASDRGRSAYDLVDRMPFLYVRVVKAKTANNESKSPVYAKLMIGTHSIKTKSQSDKDWDKVFAFDKEGLNSTSLEVSVWTEEKKENEEATQECSLGTVSFDLQEVPKRVPPDSPLAPQWYALESENSAGNEVMLAVWIGTQADEAFQEAWQSDSGGLLPETRAKVYLSPKLWYLRLTVIQTQDLHLGSAKARNPELYVKAQLGAQLFKTGRTSVGSTSASSANPTWNEDLVFVAAEPFEPFLTVTVEDVTNGQSVGHAKIHVASIERRTDDRTELKSRWFNLVGDDTKPYTGRIHVRVCLEGGYHVLDEAAHVTSDVRAAAKQLAKAPIGLLEVGIRGATNLLPVKTRDGTRGTTDAYVVAKYGPKWVRTRTILDRFNPRWNEQYTWDVYDPCTVLTIGVFDNGRYKHDEAAGKQGKDVRVGKVRIRLSTLDTNRVYLNQYSLTVLLPSGAKKMGEIEIAVRFSCSSWLSLIQAYTSPMLPRMHYVKPLGPAQQDILRHTAMRLVTARLTRSEPPLGQEVVQFMLDSDTHMWSMRRSKANWFRVVGCLTHVATLARWIEGIRTWVHPPTTILMHVLLVAVVLCPHLVLPTIFMYAFLILVFRFRYRQRVPLNIDSRLSYVDMVGLDELDEEFDGFPSTRSQDVVRIRYDRLRALAGRAQTLLGDFAAHGERLEALWNWRDPRATGIFVVFCLVASLVFYVIPFKVFVLGFGFYYLRHPRFRDDMPSVPVSFFRRLPSFSDQIL

>Potri.013G107700

MTILVVEVHDACDLMPKDGHGSASPYVEVDFDEQKQRTQTKPQELNPIWNEKLVFSVRNPRDLPNKTIEVVVYNDRKGGHNKNFLGCVRISGISVPLLSDSEAIDPQRYPLDKRGPFSHVKGDVALKIYAAHDGSHPPPPPPPTNTGNIETEATPVFQEIKTTMLQEDVIDDHEKKKKKKKNKDKEVRTFHTIGTATAAPAAAPAPPVSTGFVFQPQVMKEKAPTVETRTDFARAGPPTAMNMQMPRQNPEFLLVETSPPVAARMRYRGWDKMASTYDLVEQMHYLYVSVVKARDLPVMDVSGSLDPYVEVKLGNYKGKTKYLEKNQSPVWTQIFAFAKDRLQSNLLEVTVKDKDFGKDDFVGRVFFDLSEVPLRVPPDSPLAPQWYILEDKKGVKTRGEIMLAVWMGTQADESFPEAWHSDAHDISHTNLSNTRSKVYFSPKLYYLRVHVIEAQDLVPSDRGRMPDVYVKVQLGNQLRVTKPSEMRTINPIWNDELILVASEPFEDFIIVSVEDRIGQGKVEILGRVILSVRDVPTRLETHKLPDPRWLNLLRPSFIEEGDKKKDKFSSKILLCLCLDAGYHVLDESTHFSSDLQPSSKHLRKQNIGILELGILSARNLLPLKGKDGRTTDAYCVSKYGNKWVRTRTILDTLNPRWNEQYTWDVYDPCTVITIGVFDNCHINGSKEDARDQRIGKVRIRLSTLETNRIYTHYYPLLVLTHSGLKKHGELHLALRFTCTAWVNMLAHYGKPLLPKMHYYHPISVRHIDWLRHQAMQIVAARLARSEPPLRREAVEYMLDVDYHMWSLRRSKANVHRMMSMLSGVTAVCKWFNDICYWRNPITTCLVHVLFFILVCYPELILPTIFLYLFVIGLWNYRFRPRHPPHMDTRLSQADNAHPDELDEEFDTFPASRPSDIVRMRYDRMRSVAGRVQTVVGDLASQGERAQALLSWRDPRATAIFILFSLIGAVLIYVTLFQVVAVLVGLYVLRHPRFRSRMPSVPVNFFKRLPSRADMLL

>Potri.014G081700

MNNLKLGVEVVGAHDLMPKDGQGSANTFVELRFDHQKFRTAIKDKDLSPVWNESFYFNISDPNKLSNLSLEAIVYHHNRENSSQSILGKVRLTGTSFVPYSDAVVLHYPLEKQGILSRVKGELGLKVFVTDGPSIRSSNPLPAMESSPFSDSRATQTQASEQQIPNVAQKMFSDDKSESRQTFHHLPNPSQSQKQQHVPPAATQPPMDYGIHEMKSEPQAPRVVRMFSGSSAQPVDYALKETSPFLGGGQIVGGRVIRGDRPSSSYDLVEQMKYLYVRVVKAHDLPTMDVTGSLDPYVEVKVGNYKGITKHFEKNKNPEWNEVFAFAGDRLQSSVLEVMVKDKDLVKDDFVGIVRFDRNEVPTRVPPDSPLAPEWYRLEDKKGEKVKGELMLAVWYGTQADEAFPDAWHSDAISPDSSSFISTLIRSKVYHSPRLWYVRVKVIEAQDLVVSDKNRFPEAYVKVQIGNQVLKTKMAQSRTMNPVWNDELMFVAAEPFDDHLILVVEDRTGPNKDESIGKVVIPLNTVEKRADDHIIRSRWFGLERSVSAAMDEHQVKKDKFSSRLHLQVVLDGGYHVLDESTHYSSDLRPTAKQLWKPSIGVLELGVLNAEGLHPMKTREGKGTSDTYCVAKYGQKWIRTRTIINSLSPKYNEQYTWEVFDTATVLIVGVFDNNQHGGSNGNKDTKIGKVRIRLSTLETGRVYTHSYPLLVLHPSGVKKMGELHLAIRFSNTSFTNMVFQYSRPLLPKMHYVRPLTVMQQDMLRHQAVNVVAARLGRSEPPLRKEVIEYISDADSHLWSMRRSKANFFRLMSVFSGLLSVGKWFGEVCMWKNPITTVLVQILFVMLLYFPELILPTAFLYMFLIGVWNYRFRPRYPPHMNTRISHADAVNPDELDEEFDTFPSRQSPEIVRFRYDRLRSVAGRIQTVVGDVATQGERVQALLSWRDPRATTIFLIFCLVVAIVLYATPFQVLALLGGFYFMRHPRFRHKTPSAPINFFRRLPARTDSML

>Potri.016G049100

MQRPLHEDFLLKETNPHLGGGKITGDKLTSTYDLVEQMQYLYVRVVKAKELPAKDVTGSCDPYVEVKLGNYKGTTRHFEKKSNPEWNQVFAFSKDRMQASMLEVTVKDKDFVKDDFMGRVLFDLNEVPKRVPPDSPLAPQWYRLEDRKGDKFKGELMLAVWMGTQADEAFPEAWHSDAATVTGTDGLANIRSKVYLSPKLWYLRVNVIEAQDLQPSDKGRYPEVYVKATLGNQVLRTRVSPSRSINPMWNEDLMFVAAEPFEEPLILSVEDRIAPNKDEVLGKCAIPMHYVDRRLDHKPVNTRWFNLERHVIVEGEKKKETKFSSRIHTRICLEGGYHVLDESTHYSSDLRPTAKQLWKNSIGVLEVGILNAQGLMPMKTKDSRGTTDAYCVAKYGQKWVRTRTIIDSFTPKWNEQYTWEVFDPCTVITIGVFDNCHLHGGDKPGGARDSRIGKVRIRLSTLETDRVYTHSYPLLVLHPNGVKKMGEIHLAVRFTCSSLLNMMHMYSQPLLPKMHYIHPLTVSQLDSLRHQATQIVSMRLSRAEPPLRKEIVEYMLDVGSHMWSMRRSKANFFRIMNVFGGLIAVGKWFDQICNWKNPITTVLIHILFIILVLFPELILPTIFLYLFLIGVWYYRWRPRHPPHMDTRLSHAESAHPDELDEEFDTFPTSRPPDIVRMRYDRLRSIAGRIQTVVGDLATQGERLQSLLSWRDPRATALFVLFCLIAAIVLYVTPFQVVALLTGFYVLRHPRFRHKLPSVPLNFFRRLPARTDCML

>Potri.016G049300

MNPIAAPDHKDDFKLKNTKPQLGERWPHGGPRGGGGWISSERATSTYDLVEQMFYLYVRVVKAKDLPTNPVTGSFDPYMEVKVGNYKGKTQHFEKKTNPEWNQVFAFSKEKIQSSVVEVFLRDREMVLRDDYVGKVVFDMHEVPTRVPPDSPLAPQWYRLEGRSGDRKVRGEVMLAVWMGTQADEAFPESWHSDATSVHGEGVFNIRSKVYVSPKLWYLRVNVIEAQDVESLDRSQLPQVFVKAQVGNQILKTKLCPTRTTNPMWNEDLIFVAAEPFEEQLILTVENKASPAKDEVMGRANLPLHIFERRLDHRPVHSKWFNLEKFGFGALEGDKRHELKFSTRIHLRVCLEGAYHVLDESTMYISDQRPTARQLWKQPIGILEVGILSAQGLLPMKKKDGRGTTDAYCVAKYGLKWVRTRTIIENFNPKWNEQYTWEVYDPCTVITLGVFDNCHLGGTENPATVGGARNDMRIGKVRIRLSTLETDRIYTHSYPLLVLQPSGLKKMGELQLAVRFTCLSLANMIYLYGQPLLPKMHYLHSFTVNQLDSLRYQAMNIVAVRLGRAEPPLRKETVEYMLDVDSHMWSMRRSKANFFRIVSLFSGVISMSKWLGEVCKWKNPVTTVLVHVLFFILICYPELILPTIFLYMFLIGLWNYRFRARHPPHMDTKLSWAEAVHPDELDEEFDTFPTSKQQDVARMRYDRLRSVAGRIQTVVGDMATQGERFQALLSWRDPRATSLYIIFCLIAAVVLYITPFKIITLGTGLFWLRHPRFRSKQPSVPSNFFRRLPSRADSML

>Potri.016G113800

MTQPPNIVRKLLVEVVDARDLLPKDGQGSSSACVIADFDGQRKRTTTKYRDLNPVWKETLEFIVSDPNNMEFEELEVEVLNDKKFGNGSGRKNHFLGRVKVYGSQFSKRGEEGIVYFPLEKKSVFSCIRGEIGLRICFYDELVEEDQQQAPAPSEEDADTLQDQKPLKSPAVIEEEGRVFEVLARPEINCHDYHHPHHHHFHHNGTHSPPFVVIEESPPPVVQVNSEPSLGSQQVPLPEEPHYVETHTQYHPEVRRMQTTRVASSGDNRVKTLRPPIGDFSPKVISGRFKSESTERIHPYDLVEPMQYLFISIVKARGLSQNESPIVKLRTSTHCVRSKPASYRPGASPDSPEWHQVFALGHNNKTDGQLPNAAGNIEISVWDARSEQFLGGVCFDISEVPVRDPPDSPLAPQWYRLESDAAAGQICNRVSGDIQLSVWIGTQADDAFAEAWSSDAPYVSHTRSKVYQSPKLWYLRVTVIEAQDLHLSSNLPPLTVPDIRIKAQLGFQSARTRRGSMSNHSTSFRWIDDLIFVAGEPLEESLILLVEDRTTKEAVLLGHIIIPVSSIEQRYDERHVASKWFALEGGGGDTGGAGCATGGSYRGRIHLRLCLEGGYHVLDEAAHVCSDFRPTAKQLWKPAIGVLELGILGARGLLPMKTKGGGKGSTDAYCVAKYGKKWVRTRTITDSFEPRWNEKYTWQVYDPSTVLTIGVFDNWHMFGEMSDDKPDCRIGKIRIRVSTLESNKVYMNSYPLLVLLRTGLKKMGEIELAVRFACPSLLPDTCAVYGQPLLPKMHYLRPLGVAQQEALRGAATKMVSLWLARSEPPLGPEVVRYMLDADSHAWSMRKSKANWFRIVAVLAWAVGLAKWLDDIRRWRNSVTTVQA

>Potri.019G080000

MAKLVVEVHDACDLMPKDGHGSASPFVEVHFDEQRQRTQTKPRELNPIWNEKFSFNVNNPRDLPSKTIEVVVYNDRKGGHHKNFLGHVRISGNSVPLLSDSEAIDLQRYPLEKRGLFSHIKGDIALKIYAVHDGNHYPPPPTNAGNFETEATPAFQEINTNKLQAEDAIGDHEKKNKKKRKDKEVRTFHSIGTATGVETRTDFARAGPPTAMHMHMPKQNPEFLLVETSPPVAARMRYRGGDKMACAYDLVEQMRYLYVSVVKAKDLPAMDVSGSLDPYVEVKLGNYKGKTKYLEKNQSPVWKQNFAFSKDRLQSNLLEVTVKDKDFVTKDDFVGRVFFDLSEVPLRVPPDSPLAPQWYRLEDKRRIKTRGEIMLAVWMGTQADESFPEAWHSDAHDISHTNLANTRSKVYFSPKLYYLRVQIIEAQDLIPSDKGRMLEVSVKVQLGNQGRVTRSLQTRTINPIWNDELMFVASEPFEDFIIVSVEDRIGPGKDEILGRVILSVRDIPERLETHKFPDPRWFNLFKPSLAQEEGEKKKEKFSSKILLRLCLDAGYHVLDEATHFSSDLQPSSKHLRKPSIGILELGILSARNLLPMKGKDGRTTDAYCAAKYGNKWVRTRTILNTLNPRWNEQYTWEVYDPCTVITLGVFDNCHINGSKDDSRDQRIGKVRIRLSTLETHRIYTHYYPLLVLTPSGLRKHGELHLALRFTCTAWVNMVTQYGKPLLPKMHYVQPISVKHIDWLRHQAMQIVAARLSRAEPPLRREVVEYMVDVDYHMWSLRRSKANFLRIMSLLSGITAACKWYNDICNWRNPITTCLVHVLLFILVCYPELILPTIFLYLFVIGLWNYRFRPRHPPHMDTRLSQADNAHPDELDEEFDSFPASRPSDIVRMRYDRLRSVAGRVQTVVGDLASQGERAQALLSWRDPRATAIFILFSLIWAVFIYVTPFQVVAVLVGLYLLRHPRFRSKMPAVPVNFFKRLPSKTDILL

>Sm403140

MGKKVVVEILSAHNLMPKDGHGSANAYCIVEFDGQRQATKVKTKDLNPVWNEKLEFQVRDAQSMAQEAVRIEVLTAHPKEKNNRKKDGFLGRVRIEGISIKKQGDEAIVSYLLQKRSPFSHIKGELRVKVYWVDEAKKEEAKKGEEKKDGDKKDGEKKEGEKKNGEKKDEKKVERPPPPTVVPAADYVLKERAPVVTEKVRTYDLVEKMLYLYVRVVKGRNISKEEPYVVIKFGEAVVAKKKATKKDKVAVWEEVFAFSKDKIQGPTVEIVVAEDEKGSKDLGSVVLEISDIPFRVPDSPLAPQWHSLEDRKTRVKKDEGEVMLAVWSGTQEDESFPIAWQSDTGGHAHTKAKVYLSPKLWYLMVNVIEAQDLAVSDKSRFPNVCARVTLGPYQKWTTTFPKTPSASPMWNESKMFVAAEPFEEHLVVFVEDKVSADKAEVLGSVKISLAGNKQIARRSDPKEPVASFWYNLDKNGDKGFKGRVHLRLSFEGGYHVMDESTSYISDMRPTAKHLWKKSLGILQVGILQAKALLPMKNKDGRGTTDAYCVAKYGPKWIRTRTVVDSLNPKWNEQYTWEVYDPCTVVTICVFDNCHLSDNSSNAQPDGLIGKIRIRLSTLESNKVYANSYPLIALQPSGVKKMGELEITVRLATTTLIHVLQAYFQPPLPKLHYTRPLPVAEQEMLRIEAIRIVAGRLGRAEPPLRQEVIRYMLDTESNMFSMRRSRANYARLTNVLSGLVVVSNWFHEICKWSSPVTTLLVHVLFLILAWFPELILPTLFLYLFLIGVAHYRHRPRAPPSMDAQLSHATDGLSPDELDEEFDTIFTKKHPDLVKARYERLRLAASRLQTVVGDIAAQGERVHALLSWRDPRATGIFITFCFMLAIVLYVVPFKVIAILVGLYAMRHPRFRDKSPSVPMNFFRRLPSLADRIL

>Sm93870

MGRKLFVEVCNAADLMPKDGQGSASAYCTLEFDGQRRKTDTKAKDLNPVWNTVVEFPILEGKNLESEVLELSVLCEKRGAQRKPGFLGKVKIPGRSIVKKGEEALVYYPLEKRSMFSQVKGEIGLKVWWSDDPDPSPPAPASPSAAAAASGGGTPPAEEKKEDHHKEEKEERKEEEKKEEKKKDEKHEEKKKDEKHEEKKKEEKHEEKNEQKHEPKQYEEKKKEEKKENKKEDKEEKKPEETREYRSVGRREEQKDTTPALARGIGERVVTYDLVEKMNYLFVKVVKARALMESGSGSSYARIVFGSLTAKTKEVGKSLFPEWHEIFAFSKDNSAGPVVEVSIWDHETDQFMGAVGFDLQEIPFRVPPDSPLAPQWYRLENISKNAEKKVRGDVMLAIWWGTQADEAFTEAWQSDSGGYAHTRAKVYLSPKLWYLRVNVIEAQEVQPMDRTRFPEVSVRAQLGFQIYKTKVASNRNTSPQWNEDLLFVASEPFEDELLLVVQNKTAKPNEEEVLGMVKIALAGIEKRIDHRQVNSKWFDLVRYNGGDKHFHGRLHLRLCFDGGYHVMDEATHYSSCVRPTAKQLWRPVVGVLELGIIRGKDVHPMKTVDGRGATDAYCVAKYGQKWVRTRTIVDNLNPRWNEQYSWEVYDPCTVLTVGVFDNCHVHPHPEGGKDLKDLQIGKVRIRLSTLESERIYTNSHPLLMLQRSGVRKLGEIELAVRYSSVSIVSVMGLYFRPLLPKMHYLHPLGVTQSEILRISAMRLVAIRLNRSEPPLRQEVVQFMLDADFHVWSLRRSKVNYFRIMNLLAGPMAVGTWFHNICHWKNPVTTLLVHILFLILVMFPELILPTLFLYLSLIGAWRYRYRPRSPPSMDGKLSQAEQVEPDELDEEFDPIPTNKDPSVVKARYDRLRIVSSRIQHVLGDIATQGERLTALLSWRDPRASGIMVAVCMTIAIFLYVVPLRVIVVIVGLYVLRHPKFRERLPGWPINFFRRLPSLADRIL

>Sm171388

MIATKAIGDKVVTFDLVERMQYLFVRVVKARALASKDAAIDPFAKISLGSHTARTRSVPSTLYPEWNEVFAFGKERMGGPALEIAVSDDRDPDSSFLGSVVFEFAEIPVRVPPDSPLAPQWYRLERKSHHSQSSPRTVRGDIMLAVWLGTQADEAFTEAWQSDSGGYAHTRSKVYLSPKLWYLRVNVIEAQEVHLERFQPEVTVRAHLGFQVQRTRVASNRTTSPFWNEDLLFVAAEPFEDDLVLRVEERKSGGEKEEHALLGLVRIALSGVERRIDHRQVSSRWYNLEKHSGGGDGSEDEQKKHSFHGRLHLRVCLDGGYHVLDEPVNHLSCANPTARQLWKAGVGMLELGIIRGKDVLPMKNKEGRGSTDAYVVAKYGSKWVRTRTVMDSLNPRWNEQYRWDVHDPCTVLTIGVFDNAQLANRDARIGKVRIRLSTLESDRVYTNRYPLLSLQQSGVKKLGEVELAVRFTSASVLSMLQLYFQPLLPRMHYLHPLGVTQAEILRISAMRIVAIRLARSEPPLRQEVVQYMLDTDVNVWSLRRSKVNYFRLMSVLNGPMAVVRWMENICHWRNPVTTVLVHILFLILVWYPELILPTLFLYMFLIGLWQYRSRPRSPPSMEARLSQAEVVEPDELDEEFDPIPSAKDPNVIRARYDRVRIVAARIQNVLGDLATQGERVGALLSWRDPRATAIFVTFSLVVAVVLYVVPIRVIVVVAGLYAMRHPRFRDPLPAAPINFFRRLPSLADRIL

>Sm123924

MLPPGDFALKDTSPVLGHVGEKHISHDLVEKMQYLYVRVVKARDLVAKDLGGSSDPYVKVKVGEGYPAKTEIRKRSVNPVWNQVFAFGKDKIQGPTVEITVWDADKVSKDDFLGFVQFDLTEISKRVPPESPLAPQWYKLEPGRKGDVHVRGEIMLAVWWGTQADEAFSEAWQSDSGGHYHNKAKVYMSPKLWYLRVNVIEAQDLIPSEKNRLPEVSVRVQLGGTQVYKTKVSANRTNSPFWNQDMVFVAAEPFEEHLVLTVEDRVGGNKEEVLGVVKIPLKEVDRRIDHRLVNTRWFNLEKNGEKPFRGRLHLRVCFDGGYHVMDESTHHISDTRPTAKQLWKASMGVLEIGILSAKNLVPMKSRDGRSTTDAYCVAKYGQKWVRTRTCMDSFSPRWHEQYTWEVHDPCTVLTIGVFDNCHTKDEPGEKVSSGRDNPIGKVRIRVSTLESDRVYTNSYPLLVLQRSGVKKTGELELAVRFSCTSVLNMMHIYFTPPLPKMHYLHPLGVIELEQLRNIAIRIVSLRLARSEPPLRQEVVHYMLDTDSNMWSMRRSKVNYYRMLGVLSGAIAVTKWFSDICQWKNPLTTVLVHILFLILVWYPELILPTLFLYMFLIGAWHYRFRPRAPPYMDARLSQAEHVEHDELDEEFDTFPTSKSPDIVKHRYERLRMVASRIQSVLGDLASQGERLNALLSWRDPRATAIFITFCLVAAILLYVIPLRVVAVLLGIYALRHPRFRNRVPPVPMNFFRRLPSYADRIL

>Pp3c16_9250

MARKLIVEVVAAKALMPKDGQGSTNAYCVLDYDGQRKRTRVKPKDLDPVWNEKFEFTITDVAMPGDLEINIQNERNSGTGRRSSFLGKVTVPVSMVPNRPEAVRWFPLQKRGLFSHIKGDLGLRIWWQPTDSVTKSYEGDGVYSEGRVRQDPFEGGGVRPNKNVAMPDRQPDPRPNSVTVPENDFFVKETNPDLGKAVDHKQHFDLVEGMMYLFVRVVRARGLLGKDTTGLSDPYCKITVGPVKTVTRVFKRSLNPEWNEVFAVGRDKIQGGSLEVSVWDEDKLTGDDFLGGFMVDLHGVPLRKPPEAPLSPQWYRLEAKTGTENVRGEIMVAIWWGTQADEAFPDAWQSDTGGQAQFRQKVYLSPKLWYLRCNVIEAQDLVSHDNRPLEPYVKVFVAPYQTLRTRPSPTGTGSPFWNEDLMFVAAEPFEDIMYLDVLDRDVVLGHARVPLNSIERRIDGRPVASRWYVLDRGGGTGGSFLGRIHLRLCFDGGYHVMDESPNYISDTRPTARHLWRRPLGVLELGIHGANNLLPMKTTKDHRGSVDAYCVAKYGPKWIRTRTIFDSFNPRWQEQYTWEVHDPCTVLTVSVFDNRHTVPAGDAVSVKDLPIGKVRIRLSTLESDHVYTNAYPLLVVTPQGVKKIGEVELAIRFSCASTMNLIHSYLQPQLPKMHYFYPLDPRQMESLRMAAMNIVALRLMRSDPPLRQEVVQFMLDTEAERWSMRRSKANYYRIMGVLNGVLAVMNWFTDICSWKSPVTTVLVHILYLILVGYPELFLPTVFLYMFLIGSWSYRFRPRTPPFMDAKLSQGEYIGDPDELEEEFNVVPANRAQEVLKYRYERLRGVAGRIQNALGDLASMGEKLQSLLSWRDPRASAVFIAFCLTSSILLYVTPFQVVAVLLGVYALRHPRFRDPLPSIPLNLFKRLPSQADRIL

>Pp3c10_11080

MTGARKLVVEVISAKDLMPKDGHGSSNAYCVLDYDGQRKRTKVKSKDLDPTWNEKFEFAIHDPSAPGVLEINVQNEMNSGTGRRSSFLGRIVVPVSTVPPKPEAVRWYPLQKRGLFSHIKGDLGLKIWWALDEPPKAKNEKKEEKKLKEEKTEGAGGKKEGGGGGKKNQSKDDGGGDQENQGTAKSEGDGDAKENVKVGDGGGDEKPKGGGGEQNSKGAGGNGGNAGGKSKEVKKEVKADNQPQMPRPSLITVPETDFTVKETNPDLGKAVDYRQHFDLVEQMSYLFVRVVRARGLMGKDTNGLSDPYVRITVGPVRTETKIIKHDLNPVWNQVFAVGKDKLQGGTLELSVWDADKQSKDDFLGGFMIDLSEVPVRKPPESPLAPQWYRLESKVGPGRVRGEIMVAIWWGTQADEVFPEAWHSDTGGHAMFRSKTYLSPKLWYLRVNIIEAQDLVAMDKGRLPEPFVRAQVGPYQMLRTRPSAAVRGSSPFWNEDLMFVASEPFEDWLNLLVEDAAGPMGEILGLARIPLSTIERRIDGRPVPSRWYILEREGGKGGPFLGRIHLRLCFDGGYHVMDESPNYISDTRPTARQLWRPPLGVLELGIHGANNLLPMKTTKDNRGTTDAYCVAKYGPKWVRTRTIFDTFNPRFNEQYTWEVYDPCTVITVSVFDNRHTQPTGPAQVKDLPIGKVRIRLSTLESDRVYTNAYPLLVVTPQGVKKMGDIELAVRMTCASTANLMHAYVQPQLPRMHFFYPIEPRQQEHLRVAAMNIVALRLMRAEPPLRQEVVRFMLDTEAERWSMRRSKANYFRIMGVLHGVLAIMNWFSDICSWKSPVTTVLVHILFLILVWYPELLLPTMFLYMFLIGAWNYRFRSRIPPFMDSKLSQGEYIGDLDELEEEFNVVPANRAAEVLKLRYERLRSVAGRIQNALGDLASMGERLHSLLSWRDPRATAMFITFCLLTAIILYVTPFQVAAVLLGVYVLRHPRFRDPLPGLPINFFKRLPSQSDRIL

>Pp3c14_25200

MSGGRKLVVEITSARDLMPKDGQGSSNAYCVLDYDGQRKRTKVKTKDLDPTWNEKFEFLIHDPSMPGDLEINVQNDRNNGTGRRSSFLGRVVVPVSTVPPKPEGVKWHPLQKRGLFSHIKGDLGLKVWWAVDEPPKSDGEKKKKEGKAEGAKEEKEGGGGGGGKKQNKDGGGGNAAEDGNDGNEDTKPKGGGGGGEDKPKNGGGGGEQNSKGGGGNASGGGAGKSKEVKREAKADNQPQMPRPSLITVPEADFTVKETNPDLGKAVDYRQHFDLVEQMSYLFIRVVRARGLMGKDANGLSDPYVRITVGAVRTETKIIKHNLNPEWNQVFAVGRDKVQGGTLELSVWDADKQSKDDFLGGFMIALSEVPVRKPPESPLAPQWYRLESKAGPGRVRGEIMVAIWWGTQADEVFPEAWQSDTGGHAMFRSKTYLSPKLWYLRVNVIEAQDLGGMDKGRVPDPFVKAQVGPYQMLRTRPASVRSSSPFWNEDLMFVASEPFEDWLLLLVEDASGPRGEILGLARIPLNTIERRIDGRPVPSRWYILEREGGKGGPFLGRIHLRLCFDGGYHVMDESPNHISDTRPTARQLWRPSLGVLELGIHGANNLLPMKTTKDNRGTTDAYCVAKYGPKWVRTRTIFDSFNPRFNEQYTWEVYDPCTVITVSVFDNRHTHPMGPAQVKDLPIGKVRIRLSTLESDRVYTNSYPLLVVTPQGVKKMGDIELAVRLSCASTANLMHAYLQPQLPRMHFFYPIDPRQQEQLRVAAMNIVALRLMRSEPPLRQEVVQFMLDTEAERWSMRRSKANYFRIMGVLSGVLAVMNWFSDICSWKSPVTTVLVHILFLILVRYPELLMPTVFLYMFLIGAWNYRFRSRTPPFMDAKLSQGEYIGDLDELEEEFNVVPASKAPEVLRYRYERLRGVAGRIQNALGDLASMGERLHSLLSWRDPRATAMFITFCLIAAIVLYVTPFQVVAVLLGVYALRHPRFRDPLPALPINFFKRLPSQSDRIL

>Pp3c27_540

MVRKLIVEVVAAKALMPKDGQGSTNAYCVLDYDGQRKRTRVKSKDLDPTWNEKFEFLIRDVAIPGDLEINIQNERNSGTGRRSSFLGKVTVPIQFVPNKPEPVRWYPLQKRGLFSHIKGDLGLRIWWQPAESITKSMADEMSNRGRVRQDLMEGGGGRANNNVAKADRQPYPIRPNPGIVPETDFIVKETNPDLGKAVDYNQHFNLVEQMGYLFVRVVRARDLLGNGRCDPYCRVFVGPVKAETRIVMGDSNPEWNQVFAIGKDKIQGGAIELSVCNALSKDDFLGGFMVDLHEVPLRRPPEAPLSPQWYKLEAKTGKGSARGEIMVSIWWGTQADEAFPEAWHSDTGGQAQFRQKVYLSPKLWYLRCNVIEAQELASFDHRLSKPFVRVQVGPYQTLQTRPSFVRTGNPFWNEDLMFVASEPFEDILHLVVLDQVGSQNDILGQARIPLNSIERRIDGHPVVSRWYVLEREGGKGVAFLDRIHLRLCFDGGYHVMDESPNYISDTRPTARQLWKHPLGVLELGIHGANSLLPMKTTKDHRGSTDAYCVAKYGPKWIRTRTIFDSFNPRWQEQYTWEVHDPCTVLTVGVFDNRHAVAPGGMSKDLPIGKVRIRLSTLESDRVYTNAYPLLVVTPQGVKKMGELELAVRFSCASTVNLMHAYLQPQLPKMHYFYPLDPRQEEALRVAAMNIVALRLMRSDPPLRQEVVQFMLDTEAERWCMRRSKANYYRILGVLNGPLAVMNWFTDICSWKSPVTTILVHILYLILVWYPELFLPTVCLYMFLIGSWNYRFRSRTPPFMDAKLSQGEYVGDYDELEEEFNVVPAQRAQEVLKYRYERLRGVAGRIQNALGDLASMGEKFHSLLSWRDPRASAVFIAVCLISAIVLYVTPFQVVAILWGVYALRHPRFRDPLPSVPLNLLKRLPSQADRIL

>Pp3c27_520

MAGGRKLVVEVLAAKGLMPKDGQGSANAYCVLDFHGQRKRTRVKPKDLDPTWNEKFEFAMPEIGMSGDVEICVQNERKSGTGQRNSFLGRVIVPLNTVPNKPEAVRWYPLQKRGLFSHIKGDLGLKIWWQGSEQSLKGGKCGNNVQGEPVVVVGDGAGKNIPGIVVGGGKKREAKADQVPEGLRPNRITVPEADFTVKETNPDLGKAVDYKQHYDLVEEMTYLFIRVVRARNLMGKDNNGLSDPYVRISVGPVKTETRIIPRTLNPEWNQSFAIGRDKIQGGACELSVWDADKLSKDDFLGGFMIDLREVPPRKPPESPLAPQWYRLESKSGKGRVSGDLMVAIWWGTQADEVFPDAWHSDTGGSAMFRSKIYLSPKLWYLRVNVIEAQDLLASDRILTEPYVRVLVGPYQQLRTSRAVTRGGSPFWNEDLMFVASEPFDEMMQIYVEDRMVPGKEELLGHVQIPLMSIERRIDGRPVASRWYVLVRPGGGGGSFLGRIHLRLCFDGGYHVMDESSNYISDTRPTARQLWRPPLGVLEVGIHGANNLLPMKTTKDNRGSTDAYCVAKYGPKWIRTRTIFESFNPRWNEQYTWEVYDPCTVLTVGVFDNRHSFPVGGAPKDLPIGKVRIRLSTLESDRVYTNAYPLLVVTPQGVKKMGELEMAVRFTTAATANVLAAYLQPQLPKMHFFYPLDPRQLEMLRVAAMNIVALRLMRSEPPLRQEVVQFMLDTEAERWSMRRSKANYYRIMGVLSGVLAVMNWFSDICNWKSPVTTVLIHILFLILVWYPELLLPTVFFYMFLIGAWKYRFRSRTPPFMDAKLSQGEYIGHLDELEEEFNVIPASRAQEVLRMRYERLRGVAGRIQNAFGDLASMGEKLNSLLSWRDPRATTIFIGFCFVTAIVLYVTPFQVVAVLLGVYALRHPRFRDPLPSVPLNFFKRLPSLSDRIL

>Pp3c16_9260

MAGGRKLMVEVIAAKGLMPKDGEGSANAYCVLDYDGQRKRTRVKFKDLDPTWNQKFEFTMPAMRMQGYLEINVQNENKSGTGRRSCFMGRVVVPMNTVPSKPEAVRWYQLQKRGLFSHVKGDLGIKVWLQNLETAQKGGKNARDIQGEPAIVAGGGVPNGDVLVVGAGKLNKEAKADRVSEGPRPSTITVPEADFTVKETHPNLGNAVDYRQHHDLVEEMSYLFIRVVRARNLSGKDNNTLSDPYVKISVGPVKTETKFIPCTHNPEWNRCFAIGKDKIQGGTCELSVWDAGKISKDTFLGGFMIDLHGVPSRKPPESPLAPQWYRLESKTGNKAISGDLMVSIWWGTQADEVFPEAWHSDTGESSQFRSKLYMSPKLWYLRVNVIEAQDLLPTDRHMAEPYVRLHVGPYQTLRTSRSVTRGGSPFWNEDLLFVAAEPFDEVMHIIVEDRIAPGKEEIIGHIRIPLMSIARRIDGRPVASRWYVLERDGGRGAFLGRIHLRLCFEGGYHVVDESSNYISDTRPTARQLWKPSLGVLEVGIHCANNLLPMKTTKDNRGSTDAYCVVKYGPKWVRTRTIFESFNPRWNEQYTWEVFDPCTVVTVGVFDNRNTLTGGETLKDLPIGKVRIRLSTLESDRVYTNAYPLLVVTPQGVKKMGELEMAVRFSTASTANVIASYLQPQLPRMHFFYPLDPRQTHMLRVAAMNMVALRLMRSEFPLRQEVVLFMLDTEAERWSMRRSKANYYRIMGVLGGFLAVMNWFTDICNWKSPITTVLVHILFLILVWYPELLLPTVFLYMFLVGAWNYRFRSRTPPFMDAKLSQGEFIGHLDELEEEFNIVPANRAQEVLKHRYERLRGVAGRIQNGLGSLASMGERFQSLLIWRDPRATALFIAFCLVAAIVLYVTPFQVVAVLLAAYMLRHPRFRDPLPSVPLSFFKRLPSQSDRIL

>Aco010265.1

MSNYKLGVEVTSAHDLMPKDGQGSANPCVELHFEGQKFRTTIKEKDLNPVWNECFYFNVADPENLPNLALEAYIYNVSKTMHSKSFLGKVRIAGTSFVPFSDAVVMHYPLEKRGIFSRVKGELGLKVFLTDDPSIRPSDPLPSFDPVVNNPPQVPNPIFDPPKERMSEKRHMFHTVPKEVHHHSTAPISEQSVKYATDQMKPEPPKIVRMYSAASQQPVDYALKETSPFLGGGRVVAGRVIRADKPASTYDLVEQMQYLFVRVVKARDLPAMDASGSLDPFVEVRVGNYKGITKHFEKKQNPEWNEVFAFSRDRMQSSVLEVVVKDKDLVKDDFVGLVRFDLNDIPTRVPPDSPLAPEWYKLEGKRGDKTKGELMLAVWMGTQADEAFSDAWHSDAAAPIDSAVINAHIRSKVYHAPRLWYLRLNIIEAQDIIIPDKTRFPDVYVKVQLGNQFLRTKAVQARTFNPLWNEDHMLVAAEPFEDPLILSVEDRVGPNKDETIGRVIIPLQSIEKRVDDRVLYGRWFNLEKPFIVDVDQLKKDKFSSRIHLRVCLEGGYHVLDESTHYSSDLRPTAKQLWKPSIGLLELGVLSADGLHPMKTRDGKGTSDTYCVAKYGQKWVRTRTIINSLSPKYNEQYTWEVYDPSTVLTVGVFDNCQLGEKGGNSNGNKDVKIGKVRIRLSTLETGRVYTHSYPLLVLHPSGVKKMGELHLAIRFSSTSLINMLYIYSRPPLPKMHYTRPLTVMQLEMLRHQAVQIVAARLSRMEPPLRKEVVEYMSDVDSHLWSMRKSKANFFRLMSVFSGLFAASKWFGDVCAWKNPITTVLVHVLFIMLVCFPELILPTIFLYMFLIGLWNYRYRPRYPPHMNTKISHAEAVHPDELDEEFDTFPTSRSAELVRMRYDRLRSVAGRIQTVVGDMATQGERIQALLSWRDPRATAIFVLFCLIAAIVLYVSPFQVLAALVGFYIMRHPRFRHRLPSAPVNFFRRLPARTDSML

>Aco017114.1

MKKLRDPSPPHSQLGFHLLRSIEVSRQFEEKMQRVVVPPRPEEYSLKETTPHLGGGGAAGDKLTTTYDLVEQMQYLYVRVVKAKDLPTKDVTGSCDPFVEVKLGNYKGTTRHFEKKTNPEWNQVFAFSKERIQASHLEVVVKDKDFVKDDIIGRVVFDLTEIPKRVPPDSPLAPQWYRLEDRKGDKVKGELMLAVWWGTQADEAFPDAWHSDAATVPSDGLANIRSKVYLTPKLWYVRVNIIEAQDLQPSDKSRFPEVYVKAILGNQALRTRVSPSRTINPMWNEDLMFVAAEPFEEHLVLSVEDRVAPNKDEVLGKAIIPLQNVDRRLDYKAVNTRWYNLEKHVTVDGEQKKDTKFSSRIHVRICLEGGYHVLDESTHYSSDLRPTAKQLWKQSIGVLELGILSAQGLLPMKTKDGRGTTDAYCVAKYGQKWVRTRTIIDSFTPKWNEQYTWEVYDPCTVITIGVFDNCHLQGGEKAAGARDNRIGKVRIRLSTLETDRVYTHSYPLIVLLPSGVKKMGEVQLAVRFTCSSLLNMLHLYSQPLLPKMHYLHPLSVMQLDNLRHQAIQIVSMRLGRAEPPLRKEVVEYMLDVDSHMWSMRKSKANFFRIMGVLGPLIAVGKWFDQICHWKNPLTTILIHILFVILVLYPELILPTIFLYLFLIGVWYYRWRPRQPPHMDTRLSHAETAHPDELDEEFDTFPTSRPPDIIRMRYDRLRSVAGRVQTVVGDLATQGERLQSLLSWRDPRATALFVVFCLIAAIVLYVTPFRVVAFLTGLYVLRHPRFRHKLPSVPLNFFRRLPARTDSML

>Aco006924.1

MGAKEEEEEKLVVEVVAAHNLMPKDGQGSSSPYVEVEFEHQKRRTRSVPKELNPVWNERLLFPVSEPDDLPYRAIDVAVYNDRAHAHGGGARNFLGRVRVPAAGVPAPGEDAVPQLFPLEKRSLFSHIRGEISLKIYRTYGHAHAPAQQVGLGPGPGPGPGPGPGTAKAKEKAAPKHQKQQQQQQQQPPQLPPAVAAQPPPQQQPSAAQKQPRPRPGPELSGPGPAGAIKPMILATGPALGPEPYPVLCAGSGAQEFALKETRPRLGGAAAAAGGHRDKSSATYDLVEQVQYLYVRVVRARDLPQSGGEVHAEVKLGNYRGVTPPAPASAAHWGQVFAFSGDSVHSSVAEVLVKEREREESIGRVVFDLSEVPRRAPPDSTLAPQWYRLEDPSGDRRGELMASAWFGTQADEAFAEAWHSKAAGVHGDGLGSIKSKVYVAPKLWYLRVSVLEAQDLPPGAGDKARFPELFARAMVGAQILRTRAAAPAHPNRGPSNPVWAEDLMFVVAEPFDDLLVVSVEDRVGPGRDDVLGRAGSGAQSCRFGAGRVHLRLSLDGGYHVLDESTAHSSDLRPTAKQLWAPPVGVLELGVLGATALAPMKPPREGKPGSSADAYCVAKYAHKWVRTRTVVDSLCPRWNEQYTWEVFDPCTVITVGVFDNCHVDRTSSSSSSSPPSPRDARIGKIRIRLSTLETDRVYTHAYPLLMLHPSSGLKKMGELHLAVRFSCANAANMLHAYAGPLLPKMHYADPLLVRQVENLRYQATNVVAARLGRAEPPLGREVVEYMLDRGSHLWSMRRSKANFFRLVAVLSAPIAVGRWIESVRSWTRPVHSFLVASAYVLFVLFPELILPTLFLALAAAGLWRYRSRERHPPHMDVRLSYADAVYADELDEEFDTFPTRRPHDLVRMRYDRLRSVATQGERVQALLSWRDPRATLIFLAFCVIAAAALYAVPMKAILGMWGLYALRPPRFRSRMPSPFMSFFRRLPTKADSLL

>Aco015662.1

MSHDDLHHEDYQLKDTNPLLGERWPTAGGGTRQGLGGGGFSGWLSSDKLTSTYDLVEQMHYLYVRVVKARDLPPNPLTGSCDPYVEVKLGNYKGTTRHFDKRTNPEWNQVFAFSKDRIQSSTLEVYVKDKEMVGRDDYMGKVVFDLNEVPTRVPPDSPLAPQWYRLEDRRGEGKVRGEVMLAVWIGTQADEAFPEAWHADAASVHPEGVANIRSKVYVSPKLWYLRVNVIEAQDVQPNVRGRTPEVFVKAQVGNQVLKTKSCVAATLNPLWNEDLIFVVAEPFEEQLIISVEDRVSPRKDDLLGRVALPLTLFEKRLDHRPFVHSRWFDLEKFGIGVLEGETRRELRFATRVHLRVCLEGAYHVMDESTMYISDQRPTARQLWKPPVGVLEVGILGAKGLLPMKMRDGRGTTDAYCVAKYGQKWIRTRTMVGSFSPSWNEQYTWEVFDPCTVITIGVFDNCHLGNGGGGAVKDSRIGKVRIRLSTLETDRIYTQSYPLIVLQPSGVKKMGELQLAVRFTCLSFTNMIYLYGQPLLPKMHYLHPFTVNQLDNLRYQAMSIVAARLGRAEPPLRKEVVEYMLDVESHMWSMRRSKANFFRIMSLLSGTIGTFKWFSDVQRWKNPITTVLVHVLLLILVCYPELILPTICLYMFLIGLWNYRFRPRQPPHMDTKLSWAEAVHPDELDEEFDTFPTSRPQDVVFMRYDRLRSVAGRIQTVVGDIATQGERVQSLLSWRDPRATCLFVFFCLCAAVVLYVTPFKVVALVAGLYMLRHPRFRSKLPSVPSNFFRRLPSRADSML

>Aco003159.1

MKLVVEVVEAHDLMPKDGEGSSSAFAEVDFGNQITRTRTVPKDLNPAWNEKISFFLDDDPEGTAADLSKRQIEVSVYHERKGFPGHGFLGRVRILGSNIVKQGEEIVHLFPLERKWFFSPVKGEIALKVYVSPEPKSPPPGSASAEKSKVSSNCGVAGSEEEKEPSTIVAQAPTPPKKEEKEEGEEEAEEVIKVETTHHINKQQVSSQPGKSVEQQTPATVPVVPVMAYQQVHFGQAQPSRAEEYKLKDTKPQLGERWPIGGGHVRGSGGGWMGLGTGDKFTSTYDLVEQMYYLYVRVVKAKDLPANPITGSCDPYVEAKLGNYKGTTRHYEKRMNPEWNQVFAFSKERIQSTVLEVFVKDKEMVTRDEYVGKVVFDLNEVPTRVPPDSPLAPQWYRLEDRRGEGFKVRGEIMLAVWIGTQADEAFPDAWHADAASVQGEGVYSIRSKVYVSPKLWYLRVNVIEAQDVEPNEKGRLPEVFVKAQVGNQVLKTKPCPMRPGLSPMWNEDLVFVAAEPFDEQLVLTVEDRVSATKDEVLGRAALPLTLFEKRLDHRPVHSRWFNLERFGFAVLEGNHRKELRFASRVHLRVCLEGAYHVMDESTMYISDTRPTARQLWKPPIGVLEVGILSAQGLMPMKAKDSRGTTDAYCVAKYGQKWVRTRTVVSSFSPKWNEQYTWEVYDPCTVITLGVFDNCHLGNSGGGNSNGAAAQAKDSRIGKVRIRLSTLEMDRIYTHSYPLIALQPSGVKKMGELQLAVRFTCLSLASVIYLYGHPLLPKMHYIHPFTVSQVDSLQYMLDVDSHMWSMRRSKANFFRIVSLFSGAVGACRWFDSVCRWQNPITTVLVHVLFVILLSYPELILPTVFLYMFMIGLRNYRWRPRYPPHMDTKLSWAEAIHPDELDEEFDTFPTSRPHDLVRMRYDRLRSVAGRIQTVAGDLATQGERTQSLLSWRDPRATALFIMFCLVAAVVLYVTPFRVVALVAGLYVLRHPRFRSKLPSVPSNFFKRLPSRIDSML

>Aco011245.1

MKVAVEVADASDLMPKDGHGSANPFVEVDFQGQRQRTRTKFRDLSPSWNETLVFNLSDPSLLPSLPIDVTVYHDRRAAAAPEGGGGGHHRNFLGRVRISGVSVAPSPAEAVVQRFPLDKRGLFSXRRRRRRRRRRRPSSRAQEGEAEGKEPPAPAPRVFFSVGAAPPPAAAGAQKPPTNVFYDEKPARAVPPPPGATVVQARPPGGAGAPGAPRPEFGLVETRPPLAARLARSGRLGARDKISSTYDLVEPMHYLYVSVVKARNLPAKDITGSLDPYVEVKLGNYKGITKHLEKNQNPVWHQVFAFSKDRIQANMVEVTVKDKDLVKDDFVGRIVFDLTDVPLRVPPDSPLAPQWYRLEDKKGDKLTTGGGPELMLAVWIGTQADEAFPEAWHSDAHSVSLDALANTRSKVYFSPKLVYLRVLAIEAQDLVPADNSATSSVNPVWNEEFLFVAAEPFDEPLVITVEDRVAPGRDEPLGRLTLPVSVAVPRNEHNKPVEPKWFSLSRPTALLDEEKKESTSKFSSKVHLRLSLDLGYHVLDESTHYSSDLQPSSKNLRKPRIGILELGILSARNLVPMKAKDGRTTDAYCVAKYGNKWVRTRTLLDTLAPQWNEQYTWEVFDPCTVITIAVFDNCHVAGHNGGGDVRDQRIGKVRIRLSTLEADRIYTHLYPLLVLQTSGLKKTGELHLAVRFTCTAWVNMVTLYGKPLLPKMHYVQPISVLQLDYLRHQAMQIVAARLVRAEPPLRREVVEYMLDVDSHMFSLRRSKANFYRITSLFSGMAAIGRWYNSIRNWRNPITTVLVHVLFLILVGYPELILPTIFLYLFMIGIWSYRFRPRHPPHMDTRLSYAEMAHPDELDEEFDTFPTSKAADVVRMRYDRLRSVAGRVQTVAGDLATQGERAQALLSWRDPRATAIFIIISLIIAIVLYVTPFQVIAVIVGLYLLRHPRFRSKMPSVPYNFYRRLPSKSDMLL

>Aco009308.1

MGSYKLGVEVINAYNLMPKEKGSSNPHVELEFNGQKFRTTVKEKELNPVWNERFFFNISDPSSIPNLSLEASIYSINTERNSRSFLGKVRLAGTSFVPSSDAAVMHYPLENNIFFSRVRGELGLKVFLTVDPSIKASNPLPAIDPATKIHPQKQVLNPKPNPARDRSGGPRRTFHSIGKEAHNQHEHCPPVTTSEEPVKRDAGQFKCESPKSKCESPKFANMCSRAPSKKEAEYALKKTNPSLGFGQIVGGQVIHAEKPSTMYDLVEPMQYLFVRVVKAKDLPESTDPFTELKFGISKAATKQLEKKPNPEWNEVFAFSHECMHSTILEVQIKDKKQEKDEYIGRVRFNINDVPMRAPLDSQLAPEWYRLDDKKEEKIKGELMLAVWIGTQADEAFPDAWQSDVANAAADASVAASPHVRSKVYHAPRLWYVRVNIIEALDIEIADKDRTPEVFVKAQIGDQHFRTRKMKSRRSKYFWNEEIMFVAAEPFEDQLVLSVEDRVKLDKDEVIGRVTIPLFKVEKRVDDRTFHSRWFNLEKPNGADADKPKKDAADGDKAKKDKPASRIHLRVFLEGGYHVLDESTQYSSDFRPSAKQLWKPAVGVLQVGILGATGLLPMKTKDGKGMTDAYCVAKYGKKWIRTRTMINSLCPRFNEQHTWEIYDPATVLTVGVFDNCQIGEKGNGKKDAKDSKDGKEGQDAVIGKIRIRLSTLETGRVYTHSYPLLVLQPSGLKKMGELHLAVRFSSTSFVGMMSMYARPLLPKMHYVRPLMLFQIDVLCHHAIQILAARLGRMEPPLRKEVVEFMSDVDCHLWSMRRSKANFFRLTSVFSRLFEAGKWFKAICTWTKPVASVLVHVLFTMLVCFPELILPTAFLYMSFIGLWNYRHRAQYPPHMNTKLSQAENADPDELDEEFDALPTSKEAEVVKKRYDRLRCVAGKVQMLVGDVATQGERIQALISWRDPRATSVFVLLCSVAAAVAYVTPLRVVVALLGFYTMRHPRFRQKTPSAPANFFRRLPARIDSML

>Aco011746.1

MSSNLTLGVEVVSARELAPPRDGDGTANAFAELHFDSQQSRTTVKDRDLSPVWNEAFRFRVSSDPSSLPELRLDALLHFDSQQSRTTVKDYDLSPVWNEAFRFRVSDPSSLPELRLHALVFHLNRSTGSRSLLGKACISSTSFVSFADAATLYYPLEKRSLFSRARGELGLKVYLTNSSIPAVDPLIQPGAAFNQDPPSLADKTETVARNNSFLHLSREQVAAEPALTFAVKEMKPEPLRAVSVPAFPSASFRQPFDFQLKETNPPLGAGRAFGGRVFPGGRTAGTYDLVERMHYLFVRVVKGRELPPRDVAGSIDPFVEVRLGNYRGTTKHFEKKHNPEWDEVFAFSRESVQSSLVEVVLKDKSLAKDGVVGRVHFDLNEVPMRVPPDSPLAPEWYRLENVRGERIKGELMLAVWIGTQADECFPNALHADSAPVDPILASVHIRGKVYHAPRLWYVRVHVVEAQDVFLSDRSRVGEVFVRAKLGSQVLKTRVAPCRTPPYYRWDEDHLFVAAEPFEEELILSVEDRVGPNKEEVIGHCRVPLAQLERRFDEREVASRWFGLQRHGGGAEAIKEEKFSSKLHLRLCLEGGYHVLSESTHYSSDLRPTAKHLWRDPIGLLELGVLNADGLTPMRTRDGRGTCDPYCVAKYGQKWVRTRTVVDRLSPRFHEQYTWDVHDHATVLTVGVFDNCQLERRPAGAEAVRDAIVGKVRIRLSTLETGRVYTHSYPLLVLHNSGVKKMGELHLAIRFSATSFSDTLYAYSRPLLPRMHYVRPLSMVQQEVLRHQAVQIVALRLSRMEPPLRREVVEHMSDAHAHLWSMRRSKANFFRLMSVFSGVFAVAKWFADVCCWTNPVTTVLVHLLFVMLVCFPELILPTFFLYLFLIGLWNYRRRPRHPPHMNTRISHADVAHPDELDEEFDTFPTSRSPEIVRMRYDRLRSVAGRIQTIVGDVATQGERCQALVSWRDPRATAVFLLFCLCAALVLYVTPFPVVALLPGFYCMRHPRFRNRLPAVPMNFFRRLPARTDCLL

>Aco005172.1

MAVAEGGSRRLVVEVCNARNLMPKDGQGTASAYAIVDFDGQRRRTKTKLRDLNPQWDEKFEFLVHDPESMTGETLELNVYNDKRTGKRNTFLGKVKISGSSFAKAGSEVLIYYPLEKRSVFSQIKGEVALKVWYVDDPPATTASSEGGSGSEAKPEKAAAEGSEKATTAAEGEKKEKGEKKTEEAKTEEKKSSPAKEEKKNKSPEKPKAEESPASDAAAAEKSKPKEEEEKQKATNAAASPAKETQPAGFSGDLEIRPSAAVADRTTTGGGGGSYDLVDRVPYLFVRLLKAKRSGADAEKRPAHAKIAIGSHSVRTRPAKGGDWHQVFAFHKASLNSTALEVSVYEETPQPPPSSEGDKPAAAAAAAADTSLGSVSFDLQEIPKRVAPDSPLAPQWYTLDGPDEAAACDVMLAVWIGTQADEAFQEAWQSDSGGLVVHTRSKAYLSPKLWYLRLTVIQTQDLRLPPLPDSKARPAGSVGPELHIKAQLGGQVFKTNRVAPGASTNPSWNEDLLFVAAEPFDPYLTISVEDASSSSSAAGAPAVVGQARVPLSTVHRRLDDRAEPPSRWLNLAGGDEARPYAGRVHVRVCLEGGYHVLDEAAHVASDVRAASKQLSKPPVGLLEVGVRGAANLVPMKLAKDGSGGSTDAYVVLKYGPKWARTRTILDQFNPRWNEQYAWDVFDPCTVLTIGVFDNVRFKQAEPGTKDARIGKVRIRLSTLDTNRVYANTYPLTAVHPSGVKKMGEIELAIRFSCPSWLNLLQAYTTPLLPRMHYVRPLGPAQQDVLRHTAMRAVSARLARSEPPLGPEVVQYLLDTDAHVWSMRRSKANWFRVVGSLSGLANVARWAHGVRTWAHPPTTVLVHALLVAVILCPHMILPTLSLYLFLVVVWRYRARPREPAGMDPKLSHTDGVAPDELDEEFDGFPSSRAADVVRMRYDRLRALAGRAQTLLGDVAAQGERVAALMSWRDPRATGIFAVLCLLASLVFYVVPFKLLVLVMGFYYLRHPRFRDDMPSASFNFFRRLPSLSDRIL

>Aco019606.1

NQINTMSNNLKLGVEVVSAHDLLPRDGDGTVTTFVELSFDGQRHRTAVRDHDLNPYFNERFFFAVSDPSSLPSLVLDATVFHLHRPSLSRSFLGRVRIPASSVPASPSDSPVLYYPLEKRGGGRGGGVFSRIRGELGLRVFLTDDPSVQPSGQNLRQDQFADIPPEPDMSTFFRLFRSQQRQQQRSDQAPPSRVMRMFSSLSSRQPAEFQIKETSPALGGGRIVRGRVVVPGDKPGAFDLVEKMEFLFVRVVKARDLPAKDVTGSLDPYVEVIIGNYRGTTWHFEKNQNPEWNEVFAFPRDRLQASAVAVEVRDRDLVRDDYVGAVQFELNDVPLRAPPDGPLAPEWHRLEDKRGGRDVGELMLAVWFGTQADECFPSALHADTAAIDPATANTHARGKVYQAPTMWYLRVNVMDAHDVYMPQGDRSPEVFVRGRVGNQMLRTKMGRPGAAGIFKWNEVHFFVVAEPFDDELTVSVEDQAGPDQDRVIGYVNIPIATVDKRPDDRRTWPKWLDLRKPTLIDVDRLREDKFATKLQLQISLDGGYHVVDELIHYSSDFRPSSKHLWNAKKPIGMLELGILSAVGLQPMKTARDGRPTCDAFCVAKYGRKWVRTRTVVDSLSPKFNEQYAWDVYDHATVITVAVFDNSQVLTGSSTRDTAIGKVRIRLSTIETGRVYAHAYPLLVLHPSSGVKKMGELHLVVRFTATSFASMLHAYVRPVLPKMHYTHPITQLQQENLRLQAVQILATRLGLAEPPLRREVVEYMSEAQAHMWSMRRSKAHFYRLTEVFGGAVAAAKWLRDICRWANPVTTVLVHLLLVILVCFPHLILPTVLLHLLAVGLWNFWYRARYPPHMNTKISHVHGVSPDDLDEEFDLFPTTQRPEVVRSRYDRLRFIAGMMQKTLGEFATQGERVQSLLSWRDPRATAIFLVFCLVTAFVFLVVPFKVLVIWLGFYWMRHPRLRHKMPSVPTNFFRRLPARTDSLL

>Aco027649.1

MLERSTQXHPAPAPAPAPGPAHAPRVISGRFVSSAEPVERVQTTYDLVEPMQYLFVRVVRARGLRPCESPYVKVRTGSQSFRSKPARDSGSGEPEWNQVFALSHAKPEPTLEISVWDGAPAAADAFLGGVCFDLSDVPVRDQPDGPLAPQWYRLEGSEPGPRSVSGDIMVSVWIGTQADDAFAEAWISDAPYVSHTRSKVYQSPKLWYLRVSVIEAQDLRLPAAAAPAPAPCTPFDVRVKVQLGFQSARTRRSTVSGSGSTFSWAEDLMFVAAEPLDDQLVVLVEDRSAAKDPALLGLAAVPVPSVEQRLDERHVPASRWVNLEAEAEGGYRGRVHLRLCLEGGYHVLDEAAHVCSDYRPTAKQLWKPPVGVLELGILGARGLLPMKTKGGGGGKGSTDAYCVAKYGKKWVRTRTVTDSLDPRWNEQYTWQVYDPCTVLTVAVFDNWRMFADAAGEERPDYRIGKVRIRVSTLESNRAYTASFPLLVLLRSGLKKMGEVQLAVRFACPALLPDTWAMYAQPMLPRMHYLRPIGVAQQEALRGAAIRTVAGWLARAEPPLGPEVVRYVLDADAHGWSVRRSKANWFRIMGVVAWAVGLARWVDDVRRWRSPVTTVLVHVLYLVLVWYPELVVPTGALYVFLIGVWYYRFRPKGPAGMDARLSQADTVEQDELEEEFEPVPTSVEVLRVRYERLRTLAGRVQRVIGDLAAQGERLQALVSWRDPRATRIFIAVCLAVALVLYTVPPKMVAVALGFYFLRHPMFRDPMPPAAVNFFRRLPSLSDRLL

>Aco005910.1

MTGPPTAAAAGGNVRKLVVEVVDARDLVPKDGQGSCSAYAVVDFDGQRKRTPTAPRDLNPQWHHRLEFVVSEPAAMDAEELDVELYHDRRFSAPSSAPRKNQFLGRVRICGSQFARRGDEGIIYFPLEKRNLLSWVRGEIGLKIYYYDEPAPPPPEEKQPDSAGAAEPPAAPDHSAAAEEPKELPEVPAPTEAAVEIQQLPPPSPPPAGAEEASPPPEAHPAPEAEVADPYPPEVRKAHTTSSTERVRRSRRSNGGDYHPAPAPAPAPGPAHAPRVISGRFVSSAEPVERVQTTYDLVEPMQYLFVRVVRARGLRPCESPYVKVRTGSQSFRSKPARDSGTGEPEWNQVFALSHAKPEPTLEISVWDGAPAAADAFLGGVCFDLSDVPVRDQPDGPLAPQWYRLEGSEPGPRSVSGDIMVSVWIGTQADDAFAEAWISDAPYVSHTRSKVYQSPKLWYLRVSVIEAQDLRLPAAAAPAPAPAPCTPLDVRVKVQLGFQSARTRRSTVSGSGSTFSWAEDLMFVAAEPLDDQLVVLVEDRSAAKDPALLGLAAVPVPSVEQRLDERHVPASRWVNLEAEAEGGYRGRVHLRLCLEGGYHVLDEAAHVCSDYRPTAKQLWKPPVGVLELGILGARGLLPMKTKGGGGGKGSTDAYCVAKYGKKWVRTRTVTDSLDPRWNEQYTWQVYDPCTVLTVAVFDNWRMFADAAGEERPDYRIGKVRIRVSTLESNRAYTASFPLLVLLRSGLKKMGEVQLAVRFACPALLPDTWAMYAQPMLPRMHYLRPIGVAQQEALRGAAIRTVAGWLARAEPPLGPEVVRYVLDADAHGWSVRRSKANWFRIMGVVAWAVGLARWVDDVRRWRSPVTTVLVHVLYLVLVWYPELVVPTGALYVFLIGVWYYRFRPKGPAGMDARLSQADTVEQDELEEEFEPVPTSVEVLRVRYERLRTLAGRVQRVIGDLAAQGERLQALVSWRDPRATRIFIAVCLAVALVLYTVPPKMVAVALGFYFLRHPMFRDPMPPAAVNFFRRLPSLSDRLL

>Aco008766.1

MAATMRKLVVEVVEARNLLPKDGTGTSSPYVRVDFDGQRRKTRTAQRDLNPTWNEALEFDVAAAADLDEPLEVDVFHDVRVGPSRRNNFLGRVRLDSRQFCVRKGEEALMHFPLEKKSFFSWVRGEIGLKVYYVDDPVPAPAAAAAAAAAAEPDNSAANDSSDEPNSVHENCNEPAAPPLAEAPNAEKPAEAPPAEAEADGATAAASAATSELEETAPAAVPTENSEKPAEESDRQNMEAKEEAPPEPIAAAAVETRSEPEKEAAGPEWAPQRPRRMKGMGAEIAREGPAKYDLVDKMQYLFVRVVRARGLPAGAAPRVRVAAHGRRASTREARRAGPHHEWDRTFAFAREPYSGDPAAAPAASLEVSVWDLPPGEEDDHDADADDAEGEERHLLGALCFDASEVPLRDPPDSPLAPQWYRLEGPRGGGGGGQLMLATWIGTQADESFADAWKADAPPSAGSSRSKVYVSPKLWYLRVTVIEAQDALPSPTRDAAIAVAVRAALGSQVLRTRAAACRGGAPSWNEDLIFVAAEPFGEDERLLLSLEIRGAGKDAAAAAVGSAALPLSAVERRVDDRTVASRWLDVLPTVKKKGGPGQGHGHVPGGRLHVRACLDGGYHVADEAPHACSDYRPAARQLWRAPLGAVELGVVGCRGLLPMRTLRGKGSTDAYAVAKYGPKWARTRTVADSLDPAWNEQYTWPVYDPCTVLTLAVFDESPPSDPDAGPKDPAPPPCSRPMGKVRIRVSTLETDRVYRGSYPLILMLPGGAKRMGEIELAVRFARAGSVLDLVHAYGLPMLPPMHHVRPIPPALQEPLRLLDAAEPRGFSMRKVRANWHRIVAALAWTADAARWVEDTRNWRNPTATALAHGALVLLAWHPDLLLPVLAVHFAAVGAWRYRRRLQGPVPHPCVRVSMAETADREELDEEFDPVPSTRAAEVVWARYDRLRAVGARVQAMLGDVAAQAERVQALVTWRDPRATGMFVAMCLAVAVVLYVVPSRMMAVAAGFYYLRHPMFRDRMPPAAHNFFRRLPALSERII

>Aco003264.1

MKVAVEVVDARDLLSKDGHGTASPYVEVELDGQRRRTQTKRDDLNPTWNETLFFDVSDPLDLPSRTIHVSVLHHRLSGSSVAPSKADAAIQRCPLDRRPGLSFSRVRGDIALRLYAVSNDRHPPPLRRSRAVDFSTAYSSAPSTAPPRSRATDFSTAYTSAPSTAPTENEEKEEKKKRRRSTTRTFHSIGSQFGDPAGPMPFDNRDTTPPAVGAEGPAIGIRPPAETRPTPTAAPTGTTKKGGGDDKITATYDLVEPTTFLYVNVVKARDLPAMDITGGVDPYVEVRVGNYRNATKPLQGNQSPEWHQVFAFANDHIQSDDVELTVKDENVVRDGFVGRVCVLLAEVPRRTPPGSPLAPQWYRLKDKQGYYTRGEIMMAVWKGTQADEAYPEASHSDTHGLPFQALVHTHCKMYYLPRLCYLRLDIISAQDLVTSEPTRLHPDLFVRAQLGNQHDQTRISPSRSVSPTWNHRTLFVATLPSKPTTANPPLLDGSASPTPPMAAAPPPLGSIQLRVFYDAGYHVLDEMAHYCSDFQPSARPLRKDPIGALELGIRSGKDLVPVRSLNGAAPTTGYYCVAKYGPKWARTRTLVNTLNPMWQEQYTWEVFDPCTVLTVAVYHNNQLDAHDSGGGLRDQPLGKLRIRLSTLDAGRAYFYHHPLQLVHPSGIKRTGELRLAVRFRCTAWVNMMRLYARPMLPKQHYAEPIPPPEAPRDRHLRGEVVEHVLEGLPRNAPHAYSFRKSLANWRRAEALLSGAAAWAAWAGYVRDWRNPVTTLLAHANLVLLVRHPDLIIPVGFIYLFGRGVWNYRHRAKLQAEQVQSVEAGVGADEVDEEFDEIPTRRAVEVVRMRYDRLRLRAGRVQTMLGDVAGQAERAHALLSWRDPRATGVFLLFLGLATAVVYNVPFWLLLLGFGLYYMRHPVLRSRVPPAPVNFYKRLPSKVDMLL

>Solyc09g064230.1.1

MNNGKEKLVVEVVAAHNLMPKDGEGSSSPFVEVEFENQRQRTQVKMRDLNPVWNEKLVFHVNDAADLPYRTIEVNVFNEKRSNTSRNFLGRARVSGSSIAKEGEEIAQLYTLDKRSLFSHVRGELSLKIYLSTTEQVKQVITDNGNAGGGGGGVVSSGAPNAKKNKKLQQKQQQQTNGTNMVVQMGQENKVNFQNQNHSKPVESVPGDIKPVVITSVPGPIIPAVTGGGGVGLYTSGQGEFSLKETSPHLGGKDKTNSTYDLVEQMQYLYVRVVKAKDFSVFGVGGGGELVAEVKLGNYRGITKRVFSNHAEWDQVFAFSKDSVQSSVVEIFVKENNKDDFLGRVWFDLNEVPKRVPPDSQLAPQWYRMEDKKGDKSKGGELMVAIWFGTQADEAFAEAWHSKAANVHFDGLCSIKSKVYLSPKLWYLRVGVIEAQDIVMGEKGSSIMRYPELFAKVQVGNQVLRTRVSPPAATRSLTNPFWNEDLMFVVAEPFEDFLLVSIEDRLAPNREEVVARVLLPVSSLERRLNEKPVISRWFNLDTHLSNANDPKAVVRFASRIHLRASLDGGYHVLDEATMYISDVRPTAKQLWKPHIGVLEVGVLGATNLVPMKMKEGKGVSVDAYCVAKYGQKWVRTRTVVDSLSPKWNEQYTWEVFDPCTVITIGVFDNSRVDKNMANPVAGNRDSRIGKVRIRLSTLESDRVYTHAYPLLMLHPSGVKKMGELHLAVRFSCANMVNMLHMYSMPLLPKMHYVHPLSVSQLDSLRHQAMNVVATRLSRSEPPLGREVVEYMLDHDSHMWSMRKSKANFFRLTNVVSWFVIMSRFLESARNWHKPMHSALALIAFTILVLVPELIIPCVLLNLAAVGLWRYRSRPRHPPHMDTRLSYAESVYPDELDEEFDSFPTSRNAEIVRMRYDRLRSVAGRIQTVVGDMATQGERFQALLSWRDPRATFLFVIFCFFAAFFFYLVPIKWVVALWGLYYLRPPRFRNRLPSSAVCFLKRLPTRADSML

>Solyc10g080430.1.1

MQRPPQEDFLLKETKPHLGGGKVMGDKLTSTYDLVEQMQYLYVRVVKAKDLPGKDVTGSLDPYVEVRLGNYRGTTRHFEKKSNPEWSQVFAFSKDRIQASVLEVNVKDKDFIKDDFVGRVMFDLNEIPKRVPPDSPLAPQWYRLEDRHGNKVKGELMLAVWMGTQADEAFPESWHSDSAAVTGADALATIRSKVYLSPKLWYLRVNVIEAQDLIPGDRSRFPEVYVKAILGNQALRTRVSMSKTINPMWNEDLMFVAAEPFEEPLILSVEDRIAPNNDVVLGRCAIPLQYIERRLDHRPVTSKWYNLEKHIIIEGEKKKEIKFASRIHMRLYLEGGYHVLDESTHYSSDLRPTAKQLWKSSIGVLELGILNAHGISPMKTKDGRATTDAYCVAKYGQKWVRTRTIIDSFAPKWNEQYTWEVFDPCTVITIGVFDNCHLQVGDKSGGAKDSRIGKVRVRLSTLETGRVYTHSYPLLVLHPTGVKKMGEIHLAVRFTCSSLLNMMHMYSQPLLPKMHYIYPLTVSQLDNLRHQATQIVSLRLSRAEPPLRKEIVEYMLDVGSHMWSMRRSKANVFRIMGVLGGLIAIGKWFDQICNWKNPITTVLIHILFLILVLYPELILPTIFLYLFLIGVWYYRWRPRHPPHMDTRLSCADNAHPDELDEEFDTFPTSRPPDIVRMRYDRLRSIAGRIQTVVGDLATQGERLQSLLSWRDPRATALFVIFCLIAAIALYVTPFQVVALVNGFYVLRHPRFRHKLPSTPVNFFRRLPARTDCML

>Solyc08g008020.1.1

MILNNLKLGVEVVGAHNLLPKDGQGSSSSFVELYFDGQRFRTTIKEKDLSPVWNETFYFNISDPSNIHMLTLDAYVYNNIRASQSRSFLGKITINGTSFVPYSDAVVLHYPLEKRSIFSRVRGELGLKVYVIDDPSIKSSTPISTVNDTQVHIHSAQTPAPKIPRSEVRHTFHHLPNPNHPQQQQQAPAVPVPHQGARYIPEEMKVPEPQPPPQLVRMHSATMAQPVDYALKETSPFLGGGRVVGGRVIRTDRMSGCTYDLVEKMHFLFVRVVKARELPAMDITGSVDPYVEVRIGNYKGITKHIEKNQNPMWNVVFAFSRERMQASVLEVVVKDKDLVKDDFVGLCRFDLNEVPMRVPPDSPLAPEWYRLADKKGEKIKGELMLAVWIGTQADEAYPDAWHSDAALSVDTVASTLIRSKVYHAPRLWYVRVNVVEAQDLVPTDKTRFPDTYVKAQIGNQVLKTKPVQARTFNPLWNEDLLFVAAEPFEDNLVLTVEDRVAPGKDEIIGRVIIPLSMVEKRADDRMIHSRWFNLEKPVVVDIDQLKKEKFSSRLHLRVCLDGGYHVLDESTHYSSDLRPTAKQLWRPPIGVLELGVLNAVGLHPMKTRDGKGTSDTYCVAKYGHKWIRTRTIVDNLCPKYNEQYTWEVFDPATVLTVGVFDNTQLGEKGSNGTKDLKVGKVRIRISTLETGRVYTHSYPLLVLHPTGVKKMGELHLAIRFTCTSFANMLYKYSCPLLPKMHYVRPFTVMQLDMLRHQAVNIVAMRLGRAEPPLRKEVVEYMSDVDSHLWSMRRSKANFFRLMSIFTGLFAAGKWFGDICMWKNPITTVLVHVLFLMLVSFPELILPTVFLYMFLIGVWNYRYRPRYPPHMNTKLSQAESVHPDELDEEFDTFPTSRSPELVRMRYDRLRSVAGRIQTVVGDVATQGERLQSLLSWRDPRATALFVTFCLVAALAMYVTPFQVIAALIGIYMMRHPRFRHRLPSVPVNFFRRLPARTDSML

>Solyc01g094410.2.1

MSNLKLGVEVVGAHNLLSKDGQGSSSPFVELHFDGQKFRTTIKEKDLDPAWNETFYFNVSDPNDLSSLTLEALVFNNNKSSQSKSSLGKVKINGSSFVPYSDAVVLHYPLEKAGVFSRARGELGLKVFITDDPSVRVSNSFPATDSSSHIGSLSSLNDEPTQRVPGFISEPVANGKKGTRRTFHHLPNVKHQQQEPYSSFAESSQPIRFGPDQMKSTSQGPKVVRMYSGSSSQPAEYSLKETSPVLGGGRVVGGRVVRGGRKSSTYDLVEPMQFLFVRVVKAQDLPSKDITGSLDPYVEVRVGNYKGVTQHFEKNQSPEWNTVFAFSKERMQSSVLDVVVKDKDMLKDDFVGIVRVDLHDVPTRVAPDSPLAPEWYRLENKKGEKKKGELMLAVWIGTQADEAFPDAFHTDVASPIDMSVPSTQIRGKVYHSPRLWYVRVNVIEAQDLVVSEKNRIPDVFVKVRIGSQLLRTKPIRSQTMNAMWNEDLMFVAAEPFEEHLILSVEDHVASNKDEALGVVIIPLSTVEKRADDRFVRSRWYNLQEPGSAEIEEPKKKEKFSSRIHLRVTLDGGYHVLDESTHYSSDLRPTAKQLWKPSIGILELGILNVDGLHPSKTRDGRGTTDTYCVAKYGHKWVRTRTVIDSLNPKFNEQYTWEVYDPATVLTVGVFDNGQLEEKGSNGKRDMRIGKVRIRVSTLETGRVYTHSYPLLILHPSGVKKMGELHLAIRFSCASMVNMMFLYSRPLLPKMHYVKPLSVTQQDMLRYQAVNIVAARLSRAEPPLRKEVVEYMSDADAHLWSMRRSKANFFRLMSVFSGLFSVGKWFGDVCMWKNPITTSLVHVLFLMLVCFPELILPTVFLYMCLIGLWNYQYRPRYPPHMNTRISHADLTHPDELDEEFDTFPTSRSSDLVRMRYDRLRSLAGRIQTVVGDVATQGERILALLSWRDPRATVLFIIFCLLAAIVLYSTPFQLFAGLFGFYAMRHPRFRHKLPSAPLNFFRRLPAQTDSML

>Solyc01g086720.2.1

MKLIVEVIDAYDLMPKDGEGSVSAFVEVDFENQLSKTRTVPKNLNPTWNHKLIFHLDDIKNHRYKYIDVSVYHERRPIPGRNFLGRVRIPCSNIVKKGEEVYQRFQLEKKWFSSFVKGEIGLKIYISSPSDPNLYPKKSPSPSNIPSIENPEQLDNPPPSLPASEVSTLDTPKDSNSSEVQNTENTAISGADQSSSFAVVEKTGHLTPSEQDTESVEHIEETSQFVFKHQAMQQPVISIRKRPGFQPTMQHGVDHPRAIPSHQGVQLPIHHQVDHPRAIHGQPGVQPPMQYQVAQSRAMHNHPKDDYELKDTNPQLGEQWPRVGGYGGRGWMNSDRHASTYDLVEQMFYLYVRVVKSKDLQPSVLTGSCDPYVEVKMGNYKGRTKHFDKKMNAEWNQVFAFSKDRIQSSVLEVYVKDKDMMGRDDNLGKVVFDLNEVPTRVPPDSPLAPQWYRLEDQRGEGKIRGEIMLAVWMGTQADEAFSDAWHADAAFVHGEGVMSVRSKVYVSPKLWYVRVNVIEAQDIIPNDQSRLPEIFVKAQVGNQVLKTDICPARTANPMWNEDLVFVAAEPFEEQLVLSIEDRVHPMKDEILGKISFPLNTFEKRLDHRPVHSRWFNLEKFGFGSLEVDRRKELKFSSRVHLRVCLEGGYHVLDESTMYISDQRPTARQLWKPPVGILEVGILGAEGLLPMKMKDSRGSTDAYCVAKYGQKWVRTRTILDTFSPKWNEQYTWEVYDPSTVITLGVFDNCHLGVEKQGTGAARDSRIGKVRIRLSTLESHRIYTHSYPLLVLHPSGVKKMGELQLAVRFTSLSLANMIHTYGHPLLPKMHYLHPFTVNQVDNLRYQAMSIVAVRLARAEPPLRKEVVEYMLDVDSHMWSMRRSKANFFRIMSLLSGLISVNRWFGDICHWKNPVTSVLVHILFLILIWYPELILPTLFLYMFLIGLWNYRFRPRHPPHMDTKLSWAETAHPDELDEEFDTFPTSRPHDIVRMRYDRLRSVAGRIQTVVGDIATQGERLQGVLSWRDPRATSLFIMFSLFAAVMLYVTPFRVVALVAGLYMLRHPRFRSKMPSVPSNFFKRLPARTDSML

>Solyc10g078680.1.1

MQRPHQEDFSLKETKPHLGGGKITGDKLTSTYDLVEQMQYLYVRVVKAKDLPAKDVTGSLDPYVEVKLGNYKGTTRHFEKKSNPEWSQVFAFSKDRIQASVLEVIVKDKDFVKDDFVGRVLFDLNDIPKRVPPDSPLAPQWYRLEERNGTKVKGELMLAVWMGNQADEAFPEAWHSDAAAVSGADGLANIRSKVYLSPKLWYLRVNVIEAQDLIPNDKSRFPEVYVKAMLGNQALRTRVSMSKTINPLWNEDLMFVAAEPFEEPLILSVEDRVANKDEVLGRCAIPLQYVDRRLDHRPVNTRWFNLEKHVIVEGEKKEIKFASRIHMRVCLEGGYHVLDESTHYSSDLRPTAKQLWKSSIGVLEVGVLSAQGLSPMKTKDGRATTDAYCVAKYGQKWVRTRTIIDSFAPRWNEQYTWEVFDPCTVITIGVFDNCHLQGGDKSGGARDSRIGKVRIRLSTLETDRVYTHSYPLLVLHPTGVKKMGEIHLPLLPKMHYLHPLTVTQLDNLRHQATQIVSLRLSRAEPPLRKEIVEYMLDVTSHMWSMRRSKANFFRIMGVLGVVISVGKWFDQICNWKNPITTVLIHILFLILVLYPELILPTIFLYLFLIGIWYYRWRPRHPPHMDTRLSCADTAHPDELDEEFDTFPTSRSPDIIRMRYDRIRSIAGRIQTVVGDLATQGERLQSLLSWRDPRATALFVIFCLVAAVVLYVTPFQAVGLLTGFYVLRHPRFRYKLPSVPLNFFRRLPARTDCML

>Solyc03g077920.1.1

MGNEQPSNPQDDYKAKETKPQLGERWPHGGFRGGGGWISSDRVTSTYDLVEQMHFLYVRVVKARDLPPNPVTGSCDPYVEVKLGNYKGKTKHFDKKVNPEWKQVFAFSKEKIQSSVIDVFVRDKEMVQRDDYLGKVVFDMNEVPTRVPPDSPLAPQWYRLEDRRGESKVRGEVMLAVWMGTQADEAFSEAWHADAALVHGEGVHSVRSKVYVSPKLWYLRVNIIESQDVESLDKTQPPQVFVKAQVGKQVLKTKVCQTRTTNPFWNEDLLFVAAEPFEEQLVLTVECKAGPSKDEIAGRLVLPLNTFEKRLDHRPVHSRWFNLERFGFGVLEGDRRHERKFSTRIHLRACLEGGYHVLDESTMYISDQRPTARQLWKQPVGILEVGILSAQGLVPIKAKDGRKTTDAYCVAKYGLKWVRTRTILDNLSPKWNEQYTWEVYDPCTVITLGVFDNGHLGAENSGKDSRIGKVRIRLSTLETDRIYTMSYPLLVLQPSGVKKMGELQLAFRFTCLSLANIIYLYGHPLLPKMHYLHPFTVNQVDSLRYQAMNIVAVRLGRAEPPLHKEVVEYMLDVDSHMWSMRRSKANFFRIVSLFSGLISMSKWLGEVCKWKNPITTVLVHLLFCILICYPELILPTMFLYMFLIGIWNHRSRPRQPQHMDTKLSWAEAVISDELDEEFDTFPTSKPENTVKMRYDRLRSVAGRIQTVIGDMATQGERFQALLSWRDPRATSLFIVFCLIAAVILYVTPFKIIALLAALLYLRHPKFRSKMPSPPCNFFRRLPARADSML

>Solyc01g006620.2.1

MQKGSIHEEFALKQTAPKIVGSGVMIGGDKVTVAYDLVEQMEYLYVRVVKAKELTKDVTGSCDPYVEVKVGNYKGVTKHFEKKINPEWNYVFAFSQDRIQASYIEVCVKDKDVLLDDMIGRVVFDLVDVPRRVPPDSSLAPQWYRLEDKRGEKLKKGEIMLAVWRGTQADEAFCDAWHSDAAAVGSEGISRIRGKVYLSPRLWYIRVNVIECQDLVPSEKNRQPECCVKVMCGNQVLKTKISSIRSCSPMWNEDLVFVVAEPFEEPLVVTVEDKVGSNFEFLGKCVLPLSIVPKRLDNKPVPSTWHNLEKHTVVEGEKKETKFASKIHMRLSLDGGYHVLDESIHYSSDFKPTSKLLWKSSIGLLELGIISATGLSAMKSKDGRGTTDAYCVAKYGPKWVRTRTIIDSLSPQWNEQYTWEVHDPCTVITVGVFDNGYLQGGKCTSIGKVRIRLSTLETEKVYTHSYPLIVLHPSGVKKMGEVQLAVRFSCTSYVNMLSKYTQPLFPKMHYAHPMSITQQDFLRFQTIQILSTRLGRAEPPLKKEVVDYMLDVGSHIWSVRRAKANFFRLMYVVSPILAIGKWFDQICHWKNPLTTILIHILFVILVLYPGLIVPTFFLYLFLIGIWHYRLKPRHPPHMDIHISHAHGVFPDDLDEEFDTFPTSRGSDKVKMRYDRLRSIGGRIQTVVGDLATQGERFHSLLSWRDPRASALFVTFCLFAAIVMYVTPFQVIVILIGIYVLRHPRFRHKVPSLSTSFFKRLPARADCML

>Solyc01g086700.2.1

MSNQNQNPNKNQNQNPNQNHNSYPLVNEDFNLKETKPTLGGGRVTANDRLGTAFDLVEQMHYLYVRVVKAKDMPLKKDGNSKSHPFVEVMLGNLKGLTLHFEDKSSPEWNQVFVALKDRIQSRLLEVCLKDKSRIGDTDDGFIGKVHFEINEVPKRVPPDSPLAPQWYWLENRKGEKVKGELMLAVWIGTQADEAFQEALHLDATAVNGDGVANIKSKVYVSPRLWYLRVNVIEAQELQIGNKNRLQPEIYVRIMLGNVVLRTKNTLSKNVCPSWNEDLMFVVAEPFEDQLVLSVEDKVAPNKDELLGKCVISLQDVEKRVDFSTPISKWYGLEKEVVSEGGNKKVCKLNSKVHLRLSFDGGYHVLDELTHYSSDLKATSKELWKPSIGVLELGILNAQGLSPMKNRDGRGITDPYCVAKYGQKWIRTRTIINSFNPNWNEQYTWEVFDPCTVITIGVFDNCHLQGEDKNDKAKDSKIGKVRIRLSTLETNRVYTHSYPLIVLTPAGVKKMGEIQLAVRFSCSSVFNMLAMYSQPLLPTLHYLHPLTYYQIDNLRHQATQIVATRLSRAEPPLRRELVEYMLDVGSNTWSIRRCKANYVRIAGILTGLIAICKWFNGICTWKNPITTVLVHIIFFLFVCFPRLILSSMFVVVFLIGTWNYRMRPRKPPHMDIKLSQAERVPWDELDEEFDTFPTSRNNDAVRMRYDRLRSIGSRMQAVAGDLANQGERFYNLLTWRDPRATALFLIFCLVASILLYVTPFTILVSLMGFYTMRHPKFREKLPSVPLSFFRRLPAKTDSLL

>Solyc11g022400.1.1

MPKDGQGSASPFVAVDFDEQLQRTQTKNKDLNPLVFNIKSPRDLENQTISVYAYDDQKQGHHKKFLGRVKISGAFIPFSDSEALVQRYPLDKRGIFSHIKGDIALRIYAVLAGGGGGVADVIPTPVLVETEKQNVNNGEDRATPFTPFQETSTNNFEEQYMKETEIKKKKEPEVRTFHSIPVPVPASVETRPPLAARMGYWGKDKTASDLVDQMHFLNINVVKARDLPVMDISGSLDPYVEVKLGNYERVTRHFEKNQYPVWNSAFAFSKERLQSNLIEVTVKDKDLGKDDIVGKVMFDIDEVPLLVPPDSTLAPQWYRLINKKGEKIPRGEIMLAVWMGTRADEAFPEASHSDAHMASQQNLVNARSKDLLPSDRSRMPEAYAKLQLGHQARTTKPSPMRHINPVWNEELMFVVSEPFEEYLIIDVVDRVGPGKDELIGRAMISLKNIPTRVDNSKLIDAIWFNLLKPSHAADDDEKKKEVKFSSKIHLRVWIDAGYHVLDESTHFSSDLQPSSKFLRKPSIGLFELGILSAKNLMPMKSKEDRITDSYCVAKYGNKWVRTRTLIDTLAPRWNEQFSWEVFDPCTVVTIGVFDNCHINGKDEARDQRNGKVRIQRIGKVRIRLSTLETDQIYTDFYPLLVLTPSGLRKHGELHLTIRFKCTAWVNMVAQYGRPLLPKMHHVHPIPVRRIDWLRHQAVQIVAARLARAEPPLRKEVVEYMLDVDYQMFSLRRSKANFFRITGLLSGISAVHGWFYGICNWRNPLTTILVHVLFVILICYPELILPTIFLYLFVIGLWNYRIRPRAPLHLDARLSQAENAHPDELDEEFDTFPTSRQTDVIRMRYDRLRSLVGRVQTVVGDLAIQGERALSILSWRDPRATAIFIILALIWAVFLYVTPFKVVAVLIGLHWLRHPRFRSKLPSVPVNFFKRLPSKSDMLL

>Solyc03g113190.1.1

MNMSSGPPPEQQPQQQEPPSRPPQLVRKLVVEILDARNLLPKDGQGSSSPYVVVDFDGQKKRTSTVCRNLNPEWNEGLEFIISDPRTMEFEELDIEVFNDKKLSNGNARKNHFLGRVKLYGSQFARRGEEGLIYFPLEKKSVFSWIRGELGLKIYYYDEMVQEEEPPPPQPEQQQQQPPPPQEEMKKTPVFVMEDPRQRMLEIPMPMEVAMEAQEQSPPIVTIEESPPPMNMPPEQQQQCSHRHEEGPPMMSGPPMMSAPVPPSEYPPQEVKRMQAGRAGERVRVMRRPNGDYSPRVISGKVGGESERISAFDLVEPMHYLFVKIVKARGLAPSESPFVKIRTSNHFLRSKPAIIRPGEPLSNPEWQQVFSLGHNKQESTNSTLEISVWDSASDHFLGGVCFDLSDVPVRDPPDSPLAPQWYHLEGGADDQHKVSGDIQLSVWIGTQADDAFPESCSSDAPYVSHTRSKVYQSPKLWYLRITVIEAQDLHIAPNLPPLTAPEIRVKAQLGFQSVRTRRGSMNHHSSAFHWSEDLIFVAGEPLEDSLILLVEDRTTKDPALLGHIIIPVSSIEQRLDERLVPAKWFGLEGGPGGAYCGRLHLRMCLEGGYHVLDEAAHVCSDFRPTAKQLWKPAVGILELGILGARGLLPLKSKGPGKGSTDAYCVAKYGKKWVRTRTITDTFDPRWNEQYTWQVYDPCTVLTIGVFDNWRMFADSGDDKPDYRIGKVRIRVSTLENNKVYTNSYPLLVLLRSGLKKMGEIEVAIRFVCPSLLPETCAVYGQPVLPKMHYLRPLGVAQQEALRGAAIKMVAAWLARSEPPLGPEVVRYMLDADSHTWSMRKSKANWFRIVAVLAWAVGLAKWLDDIRRWRNPVTTILVHVLYLVLVWYPDLIVPTGFLYVFLIGVWYYRFRPKIPAGMDTRISQSETVDPDELDEEFDTIPSSKPPEIIRMRYDRLRILAARVQTVLGDFATQGERAQALVSWRDPRATKLFIIVCLIITIVLYAVPPKMVAVALGFYFLRHPMFRDPMPPATLNFFRRLPSLSDRLM

>Solyc01g065500.2.1

MGTVRKLVVEVTEARNLLPKDGHGTSSPYVVVDFYGQRRKTRPVTRDLSPQWNEVLEFNVGKPSDVFGDMLELDVYHDKSIGPTTRNNFLGRIRLSATQFVKKGEEALIYYPLEKKYWFSWISGEIGLKIYFVEQLLVPEVKPEPKQAPPADQPTEAAPEGEKPNSIEEEPPAQVVENPTTGEAEPPANEAENPTSVLMDPPQADEITQVKRSVSLGSIPEVKVSNNINIVTGPRPISRASSAIFSDAGSGPIEPSSFDLVEKMHYLFVRVVKARSLPTVGCPVVKMVVSGSHVLSKPARKTVLFEWDQTFAFGRDAPDSSSLLEVSVWDPSSAKSFDPTSDEAGHVFLGGICFDVSEIPLRDPPDSSLAPQWYRLEGGGAHRGDLMLATWVGTQADDSFPEAWKTDTADNPASKSKVYQSPKLWYLRSSVIEAQDISHSKDSSYHIKAQLGFQVQKTKSITTTTTGSQSWNEDLVFVAAEPFTENHLLFFLIETDRTAKEQTVLAVASIPLPTIERRVDDRKVASRWFTFEDPNEEKRIYKGRVHLRLCFDGGYHVMDEAAHVCSDYRPTARQLWKAPIGTVELGIIGCKNLLPMKGKGSTDAYAVAKYGNKWVRTRTISDSLEPRWNEQYTWRVYDPSTVLTIGVFDGCSEVAFESDECMRPDFRIGKVRVRISTLTTGKVYRNTFPLLLLSQTGLKKMGEIELAVRFIRATPTLDFLHVYSQPLLPMMHHVKPLGMVQQDSLRIAAVKIVASHLTRSEPPLRREVVTYMLDADSHSFSMRKVRANWFRIINVIAGVIDIVKWVDDTRGWKNPTATLLVHALLVMLVWFPDLIIPTFAFYVFVIGAWNYRFRSRDTLPHFDPKISLAESLDRDELDEEFDAMPCTRPNELVRARYDKLRMLGERVQTILGDFATQGERVQALVTWRDPRATGIFIGLCFVVAFILYLVPSKMVSMAFGFYYLRHPIFRDRMPSPALNFFRRLPSLSDRML

>Solyc10g080420.1.1

mqrppqedfslketkphlgggkvtgdkltstydlveqmqylyvrvvkakdlpgkdvtgsldpyvevrlgnyrgttrhfekksnpewnqvfafskeriqasvlevnvkdkdfikddfvgrvmfdlneipkrvppdsplapqwyrledrsgnkvkgelmlavwmgtqadeafpeswhsdaatvsgadalanirskvylspklwylrvnvieaqdlipsdrsrfpevyvkailgnqalrtrvsmsktinpmwnedlmfvaaepfeeplilsvedrvtaankdevlgrcviplqyidrrfdhrpinsrwynlekhiivegekkkeikfasrihmrlyleggyhvldesthyssdlrptakqlwkssigvlelgilnaqglspmktkdnrattdaycvakygqkwvrtrtiidsfapkwneqytwevfdpctvitigvfdnchlhggdkpggsrdsrigkvrirlstletdrvythsypllvlhptgvkkmgeihlavrftcsslmnmmhmysqpllpkmhyihpltvtqldslrhqatqivsmrlsraepplrkeiveymldvgshmwsmrrskanffrimgvlggliaigrwfdqicnwknpittvlihilflilvlypelilptiflylfligvwyyrwrprhpphmdtrlscadnahpdeldeefdtfptsrppdivrmrydrlrsiagriqtvvgdlatqgerlqsllswrdpratalfvifcliaaivlyvtpfqvvallsgfyvlrhprfrhklpsaplnffrrlpartdcml

>Solyc01g007170.2.1

MGDQICTRKVIVEVCNAKNLMPKDGQGTASAFAIVDFDGQRRRTKTKFRDLNPQWDERLEFLVHDVDSMASETLELNVYNDKKIGKRSNFLGKVKISGSTFVGVGFESLVYYPLEKRSVFSQIKGEIGLKIWFVDEETPPPPTATEEKKEEVAAEAAPEKKEEKTPTPAPEVTEEKPSTTTDENKSESAEKKEEEKKPDESGEKKEETPPENPKDGETPAAAAPPPPEVMEHPPIAQNKPPNKNPSANENTSELKILHKNLSTGVDRRTGAFDLVDQMPFLYVRLVKAKRAHHEPGSSAYAKLVIGTHSIKTKSLADYREWDQVFAFDKDGLNSTSLEVSIWVEKKEADDKIVENCMGNVSFDLQEVPKRVPPDSPLAPQWYSLEGVTGDQNPPGNDVMLAVWLGTQADEAFNEAWQSDSGGLIPETRAKVYLSPKLWYLRLTVIQTQDLQLGSGGTEPKVRNPDLYVKAQLGAQLFKTSRTTVGSSSSASNPTWNEDLVFVAAEPFEPFLVITVEDVTNGQVVGYAKVQVTSIDKRTDDKSEPRSRWFNLIGDEKKPYAGRIHVRTCLEGGYHVLDEAAHLTSDVRATAKQLSKPPIGLLEVGIRGANNLLPVKTKDGTRGTTDAYVVAKYGPKWVRTRTILDRFNPRWNEQYTWDVYDPCTVLTIGVFDNGRYKHDDALKKDVRLGKLRVRLSTLDTNKDILRHTAMRIVTARLARSEPALGQEVVQCMLDSDTHIWSMRRSKANWFRVVGCLSRAATLARWLDGIRTWVHPPTTILVHILLIAIVLCPHLVLPTICMYAFLIISLRYRYRQRVAITMDPRLSHVDAIGPDELDEEFDGFPSSRPMEHVRVRYDRLRALAGRAQTLLGDVAAQGERLEALFNWRDPRATGIFVIVCLFASLVFYVVPFKAFVVGSGLYYLRHPRFRDDMPSVPVNFFRRLPPLSDQIL

>Solyc11g022460.1.1

maklivevldasdlmpkdgqgsaspfvevdfdeqrqrtqtknkdlnpqwneklvfniknprdlenqtisvyvyndqkqghhknflgrvkisgafipfsdsealvqrypldkrgifshikgdialriyavlagggggvadvipppvsveteqqnvnngedratpftpfqetstnnfeeqymketeikkkdkkkkkesevrtfhsipapapvpvpasgpspppvvierradfakaggpmasnvmqmqmgggprpefglvetrpplaarmgywgrdktastydlveqmqflyinvvkardlpvmdisgsldpyvevklgnykgvtrhfeknqypvwnsvfafskerlqsnlievtvkdkdfgkddivgkvmfdiaevplrvppdsplapqwyrlinkkgekipqgeimlavwmgtqadeafpeawhsdahmasqqnlvntrskvyfspklyylrvhvieaqdllpsdrsrmpeayaklqlghqsrttkpspmrhinpvwneelmfvasepfeeyliidvvdrvgpgkdeligramisfkniptrvdnsklpdaiwfnllkpshaadddekkkevkfsskihlriwidagyhvldesthfssdlqpsskflrkpsigllelgilsaknlmpmkskegritdsycvakygnkwvrtrtlidtlaprwneqfswevfdpctvvtigvfdnchingkdeardqrigkvrirlstletdriythfypllvltpsglrkhgelhlairftctawvnmvaqygrpllpkmhyvqpisvrhidwlrhqamqivaarlvraepplrkevveymldvdyhmfslrrskanffrimgllsgisavhgwfngicnwrnplttilvhvlflilicypelilptiflylfviglwnyrfrprapphmdarlsqaenahpdeldeefdtfptsrqtdavrmrydrlrsvagrvqtvvgdlatqgeralsilswrdprataifiilaliwavflyvtpfqvvavliglywlrhprfrsklpsvpvnffkrlpsksdmll

>AtMCTP10

MTEAKTGTGNERLVVEIVGAHNLMPKDGEDSSSPFVEVQFENQRLRTKVKPKDLNPIWNEKLVFHVIDVNDLRHKALEINVYNEKRSSNSRNFLGKVRVLGSSVGREGESVVQLYTLEKRSLFSSVRGEISVKHYMTTTAENGENVRRVNRSGGSKKSKKVQNVSSSMAIQQQQQQQQQQISLHNHNRGNQQQSQQNGQGQRMLPFYPHQSEIKPLVITALPSPMPGPGPRPIVYSNGSSEFSLKETKPCLGGTSNGLGGLSSHKDKTSSTYDLVEQMQYLYVNIVKAKDLSVLGEVVSEVKLGNYRGVTKKVSSNSSNPEWNQVFVFSKERIQSSVVELFVKEGNKDEYTGRVLFDLSEIPTRVPPDSPLAPQWYKIENRNGGRGNGELMVSVWFGTQADEAFAEAWHSKAGNVHIEELSSIKSKVYLSPKLWYLRISVIEAQDVAIMDKGSSLMRFPELSAKLQVGSQILRTAIASAIPTKSFSNPYWNEDLMFVVAEPFEDCVTVVVEDRLNGGAIGGQNDVAVGRVQIPISAVERRTGDTLVGSRWFSLDNGNNNNRFGSRIHLRLSLDGGYHVLDEATMYNSDVRPTAKELWKPQVGLLEIGILSATGLMPMKVRDGKCGGIADSYCVAKYGPKWVRTRTVVDSLCPKWNEQYTWEVYDPCTVVTVGVFDNARVNENNNSRDVRIGKVRIRLSTLETGRVYTHSYPLIVLHPSGVKKTGELHLAVRLSCGNAVNMLHMYALPLLPKMHYTQPLGVHMLERLRYQTLNAVAARLSRAEPPLGREVVEYMLDHDFHVWSMRRSKANFFRLVNVISGLVAVAKLVEVMRSWSKPVYSTVFVLAFLFMVLFPELLLPCLLLYTAAVGVWRFRRRSRYPPHMDARISHAETVFPDELDEEFDTFPTSRGFDVVRMRYDRVRSIAGRVQTVVGDMASQGERVQALLSWRDPRATFLFLMFCLLAAVGFYTVPVKLTVAISGLYYLRPPRFRRKLPSRGLSFFRRLPSRADSLL

>AtMCTP6

MNKLVVEIVDASDLMPKDGQGSASPFVEVEFDEQRQRTQTRFKDLNPQWNEKLVFNVGDLKRLNNKTVDVTVYDDRRDNQPGKFLGRVKIAGAVVPLSESESGVQRYPLDKRGLFSNIKGDIALRIYAAPIDGGDFVSPPPDFAEKVMKEDKRFESQEFQFQNQNQNQNHYEQFEDEINNMETLKPTKKKEKESRTFHSIGAHAGGGGGAPPMSQAKQAYPPPPNQPEFRSDFMRAPGPPTGAVMQMQPPRQQNPEFQLIETSPPLAARMRQSYYYRSSGDKTSSTYDLVEQMHYLYVSVVKARDLPVMDVSGSLDPYVEVKLGNYKGLTKHLEKNSNPIWKQIFAFSKERLQSNLLEVTVKDKDLLTKDDFVGRVHIDLTEVPLRVPPDSPLAPQWYRLEDKKGMKTNRGEIMLAVWMGTQADESFPDAWHSDAHRVSHSNLSNTRSKVYFSPKLYYLRIHVMEAQDLVPSDKGRVPDAIVKIQAGNQMRATRTPQMRTMNPQWHEELMFVVSEPFEDMVIVSVDDRIGPGKDEILGRVFIPVRDVPVRQEVGKMPDPRWFNLQRHSMSMEEENEKRKEKFSSKILLRVCIEAGYHVLDESTHFSSDLQPSSKHLRKPSIGILELGILSARNLMPMKGKDGRMTDPYCVAKYGNKWVRTRTLLDALAPKWNEQYTWEVHDPCTVITIGVFDNSHVNDGGDFKDQRIGKVRVRLSTLETDRVYTHFYPLLVLTPGGLKKNGELQLALRYTCTGFVNMMAQYGRPLLPKMHYIQPIPVRHIDLLRHQAMQIVATRLSRSEPPLRREVVEYMLDVDYHMFSLRRSKANFSRIMSLLSSVTLVCKWFNDICTWRNPITTCLVHVLFLILVCYPELILPTVFLYLFVIGMWNYRYRPRHPPHMDARVSQADNAHPDELDEEFDTFPTSRPADIVRMRYDRLRSVGGRVQTVVGDLATQGERIQALLSWRDPRATALFIVFALIWAVFIYVTPFQVIAIIIGLFMLRHPRFRSRMPSVPANFFKRLPAKSDMLL

>AtMCTP4

MQRPPPEDFSLKETKPHLGGGKVTGDKLTTTYDLVEQMQYLYVRVVKAKELPGKDLTGSCDPYVEVKLGNYRGTTRHFEKKSNPEWNQVFAFSKDRVQASYLEATVKDKDLVKDDLIGRVVFDLNEIPKRVPPDSPLAPQWYRLEDGKGQKVKGELMLAVWFGTQADEAFPEAWHSDAATVSGTDALANIRSKVYLSPKLWYLRVNVIEAQDLIPSDKGRYPEVFVKVIMGNQALRTRVSQSRSINPMWNEDLMFVVAEPFEEPLILSVEDRVAPNKDEVLGRCAVPLQYLDKRFDYRPVNSRWFNLEKHVIMEGGEKKEIKFASKIHMRICLEGGYHVLDESTHYSSDLRPTAKQLWKPNIGVLELGVLNATGLMPMKAKEGGRGTTDAYCVAKYGQKWIRTRTIIDSFTPRWNEQYTWEVFDPCTVVTVGVFDNCHLHGGDKNNGGGKDSRIGKVRIRLSTLEADRVYTHSYPLLVLHPSGVKKMGEIHLAVRFTCSSLLNMMYMYSMPLLPKMHYLHPLTVSQLDNLRHQATQIVSTRLTRAEPPLRKEVVEYMLDVGSHMWSMRRSKANFFRIMGVLSGIIAVGKWFEQICVWKNPITTVLIHILFIILVIYPELILPTIFLYLFLIGVWYYRWRPRHPPHMDTRLSHADSAHPDELDEEFDTFPTSRPSDIVRMRYDRLRSIAGRIQTVVGDLATQGERFQSLLSWRDPRATALFVLFCLIAAVILYITPFQVVAFAIGLYVLRHPRLRYKLPSVPLNFFRRLPARTDCML

>AtMCTP15(QKY)

MNTTPFHSDPPPSRIQRKLVVEVVEARNILPKDGQGSSSAYVVVDFDAQKKRTSTKFRDLNPIWNEMLDFAVSDPKNMDYDELDIEVYNDKRFGNGGGRKNHFLGRVKIYGSQFSRRGEEGLVYFPLEKKSVFSWIRGEIGLKIYYYDEAADEDTAGGGGGQQQQQQQQQFHPPQQEADEQQHQQQFHPPPQQMMNIPPEKPNVVVVEEGRVFESAQSQRYTETHQQPPVVIVEESPPQHVMQGPNDNHPHRNDNHPQRPPSPPPPPSAGEVHYYPPEVRKMQVGRPPGGDRIRVTKRPPNGDYSPRVINSKTGGGETTMEKKTHHPYNLVEPMQYLFVRIVKARGLPPNESAYVKVRTSNHFVRSKPAVNRPGESVDSPEWNQVFALGHNRSDSAVTGATLEISAWDASSESFLGGVCFDLSEVPVRDPPDSPLAPQWYRLEGSGADQNSGRISGDIQLSVWIGTQVDEAFPEAWSSDAPHVAHTRSKVYQSPKLWYLRVTVLEAQDLHIAPNLPPLTAPEIRVKAQLGFQSARTRRGSMNNHSGSFHWHEDMIFVAGEPLEDCLVLMVEDRTTKEATLLGHAMIPVSSIEQRIDERFVPSKWHTLEGEGGGGGGGGGPGGGGGGGPYCGRISLRLCLEGGYHVLEEAAHVCSDFRPTAKQLWKPPIGILELGILGARGLLPMKAKNGGKGSTDAYCVAKYGKKWVRTRTITDSFDPRWHEQYTWQVYDPCTVLTVGVFDNWRMFSDASDDRPDTRIGKIRIRVSTLESNKVYTNSYPLLVLLPSGMKKMGEIEVAVRFACPSLLPDVCAAYGQPLLPRMHYIRPLGVAQQDALRGAATKMVAAWLARAEPPLGPEVVRYMLDADSHAWSMRKSKANWYRIVGVLAWAVGLAKWLDNIRRWRNPVTTVLVHILYLVLVWYPDLVVPTAFLYVVMIGVWYYRFRPKIPAGMDIRLSQAETVDPDELDEEFDTIPSSRRPEVIRARYDRLRILAVRVQTILGDFAAQGERIQALVSWRDPRATKLFIAICLVITIVLYAVPAKMVAVALGFYYLRHPMFRDTMPTASLNFFRRLPSLSDRLI

>AtMCTP14

MADNVLRKLIVEICSARNLMPKDGQGTASAYAIVDFDGQRRRTKTKFRDLNPQWDEKLEFFVHDVATMGEEILEINLCNDKKTGKRSTFLGKVKIAGSAFASAGSETLVYYPLEKRSVFSQIKGEIGLKAYYVDENPPAAPAATEPKPEAAAATEEKPPEIAKAEDGKKETEAAKTEEKKEGDKKEEEKPKEEAKPDEKKPDAPPDTKAKKPDTAVAPPPPPAEVKNPPIPQKAETVKQNELGIKPENVNRQDLIGSDLELPSLTRDQNRGGGYDLVDRMPFLYIRVAKAKRAKNDGSNPVYAKLVIGTNGVKTRSQTGKDWDQVFAFEKESLNSTSLEVSVWSEEKIEKEDKTTTTTESCLGTVSFDLQEVPKRVPPDSPLAPQWYTLESEKSPGNDVMLAVWLGTQADEAFQEAWQSDSGGLIPETRSKVYLSPKLWYLRLTVIQTQDLQLGLGSEAKSKIPTTELYVKAQLGPQVFKTARTSIGPSASSSGSGNPTWNEDLVFVASEPFEPFLIVTVEDITNGQSIGQTKIHMGSVERRNDDRTEPKSRWFNLAGDEKKPYSGRIHVKVCLEGGYHVLDEAAHVTSDVRPSAKQLAKPPIGLLEVGIRGATNLLPVKTRDGTRGTTDAYVVAKYGPKWIRTRTILDRFNPRWNEQYTWDVYDPCTVLTIGVFDNGRYKRDESGKQGRDVRVGKIRVRLSTLDMNRIYLNSYTLTVILPSGAKKMGEVEIAVRFSCPSWLSIIQAYVTPMLPRMHYVRPLGPAQQDILRHTAMRIVTARLARSEPPLGQEVVQYMLDTDNHVWSMRRSKANWFRVITFLSRAATIARWIHGIRTWVHPPTTVLVHLLLVAIVLCPHLVLPTVFMYAFLILALRFRYRGRVKVNSVDPRLSCVDSVAPDELDEEFDGFPTTRQPEVVRIRYDRLRALAGRAQTLLGDVAAQGERVEALFNWRDPRATCIFVVFCLFASFLFYIVPFKVFLLGSGFYYIRHPRFRDDMPSVPVNFFRRLPSMSDQIL

>AtMCTP3

MQRPPPEDFSLKETRPHLGGGKLSGDKLTSTYDLVEQMQYLYVRVVKAKELPGKDMTGSCDPYVEVKLGNYKGTTRHFEKKSNPEWNQVFAFSKDRIQASFLEATVKDKDFVKDDLIGRVVFDLNEVPKRVPPDSPLAPQWYRLEDRKGDKVKGELMLAVWFGTQADEAFPEAWHSDAATVSGTDALANIRSKVYLSPKLWYLRVNVIEAQDLIPTDKQRYPEVYVKAIVGNQALRTRVSQSRTINPMWNEDLMFVAAEPFEEPLILSVEDRVAPNKDEVLGRCAIPLQYLDRRFDHKPVNSRWYNLEKHIMVDGEKKETKFASRIHMRICLEGGYHVLDESTHYSSDLRPTAKQLWKPNIGVLELGILNATGLMPMKTKDGRGTTDAYCVAKYGQKWIRTRTIIDSFTPRWNEQYTWEVFDPCTVVTVGVFDNCHLHGGEKIGGAKDSRIGKVRIRLSTLETDRVYTHSYPLLVLHPNGVKKMGEIHLAVRFTCSSLLNMMYMYSQPLLPKMHYIHPLTVSQLDNLRHQATQIVSMRLTRAEPPLRKEVVEYMLDVGSHMWSMRRSKANFFRIMGVLSGLIAVGKWFEQICNWKNPITTVLIHLLFIILVLYPELILPTIFLYLFLIGIWYYRWRPRHPPHMDTRLSHADSAHPDELDEEFDTFPTSRPSDIVRMRYDRLRSIAGRIQTVVGDLATQGERLQSLLSWRDPRATALFVLFCLIAAVILYVTPFQVVALCIGIYALRHPRFRYKLPSVPLNFFRRLPARTDCML

>AtMCTP8

MMSNLKLGVEVISARLKPREDYGGVNAYVELRFDDQKVITMTKIDDSSPVWNEKFFFNISDTEDLSNQFLDAYVYNKTSSITKSCLGKIRILGTAFLPYSEAVGLPYPLEKEKWSMFSSAAANGGELALKVFLTDNPSPKVPNLISTKKIPSKSRHKFHNIPTNESNHSPRGNQQSFQPQPPPPQSQTALPPPMMESSLYQAPRFGTPIPTTMGFNPNPPDYSIKETKPILGGGKRARSSDHDLVEPMEFLFIKIVKARNLPSMDLTGSLDPYIEVKLGNYTGKTKHFEKNQNPVWNEVFAFSKSNQQSNVLEVIVMDKDMVKDDFVGLIRFDLNQIPTRVAPDSPLAPEWYRVNNEKGGEIMLAVWFGTQADEAFSDATYSDALNAVNKSSLRSKVYHSPRLWYLRVNVIEAQDLVIVPDRTRLPNPYVKIRLNNQVVRTKPSHSLNPRWNEEFTLVAAEPFEDLIISIEDRVAPNREETLGEVHIPIGTIDKRIDDNRTVPNRWFSLKTENQRRVRFATTRLHLNVCLEGGYHVLDESTYYSSDFRPSMKELLSHKQPSFGVLELGILRIEGLNLSQEGKKETVDAYCVAKYGTKWVRTRTVTNCLNPRFNEQYTWEVYEPATVITIGVFDNNQINSGNGNKGDGKIGKIRVRISTLEAGRIYSHSYPLLVLRPSGLKKMGELHLAIRFSCSSMFQMLMQYWKPLLPKMHYARPLKVVQQEILRQHAVNLVAARLSRAEPPLRKEVVEYISDSNSHLWSMRKSRANLFRLSSVFSGLLGTGEWFQDICRWKKPVETTAIHIIFLVLVCSPEMILPVMSLCLFMLGVWNYRLRPRQPPHMDTRLSFADNIHPEELNEEFDTFPFSSQDPGIVKMRYERLRSIASRAQTVVGDIAGQGERVQALLSWRDPRATSIFMVLCLVSTVVLYVVPFKVFVLLAGLYIMRPPRFRGKTPPGPINFFRRLPAKTDCML

>AtMCTP12

MAANKDEFSVKQIFPKLGGERGARNPRYGPTSSHDLVEQMEFLYVQVIQAINNSVVNPSARICCPVVEITLGNYKSSTKNLPMGPNMDWNQVFAFDKSKGDVLSVTLKDGPTNTVINKRNFKLASEIPTRVPPDARIAPQWYSMHNTETDFYMELLMSVWFGTQVDEVYPEAWFSDACEVCASRVINTRPKVYLAPRLCYVRVTIVSGHDLISKDKNKTPSVYVTATLGKVALKTKVSSGTNPSWNQDLIFVASEPLEGTVYIRLIDREDEQHEGCIGTLKKKLTEMTPLKVPSSAPALFYDIEMPTEVKPAGDSRRFASRLKMKLATDQAYHVAEECTQYSSDNRAFVKGLWPGLLGKLEIGILGATGLKGSDEKKQTIDSYVVAKYGNKWARTRTVVNSVSPKWNEQYSWDVYEKCTVLTLGIYDNRQILEDKNKANDVPIGKVRIPLNRVQSDWIYTCSYPILKLGSSGLKKMGELQLAVRFVYVAQGYARYSAPFRWMLPKAHYKSPLSMYQIDKLRAQAVEINCANLARTEPALRSEVVSDMLKPKSRNFSIRISKDNFDRLYTVVKMVLWCVSVIASVRSTTACTPKFIALGVSFVFLFWEYYIYWLVTSWLVAYCIVLCIVVILLREILKSPRQTYNWLFYRNVTPPPLILVDLKLRKLDSINLDELAEEFDSFPSSENDLNILRMRYDRLRKIMENVMLLMGDAATQGERLLAAFTLLERPFVLIILLALCYCSMLVVCLGWDLHVRKCLIFVFICYWVQLPWFRNNLPDGSLNFFRRLPSNEDLMF

>AtMCTP9

MSNIKLGVEVISAQGLLQRDKHNSCSPFVELKFDNQIFRATTKHNDPNPVWHECFYFVVSDPSVLSTRTLEAHVYSYQNEFDAKPFLGKVRVNGTSFVPRSEAAPFNYPLEKRSVFSRARGELCLRVFITDDPSVTPSVPTPVPESPQAYSPSPRKEHVKSLITADASMATDERRELKPKTRTFHNSAPLVKQQPMMNYGIHEMRAAPMPPRVVQVNGPGPSLHQLPPDFSVKETSPLLGGGRIVGGRVVRGTERPTSGTYDLVEEMKFLYVRVVKARDLPNKDLTGSLDPYVVVKIGNFKGVTTHFNKNTDPEWNQVFAFAKDNLQSNFLEVMVKDKDILLDDFVGIVKFDLREVQSRVPPDSPLAPQWYRLENKRGEKKNYEIMLAVWSGTQADEAFGDATFSDSLVDSDSSNIISANLRSKVYHSPRLWYLRVQILEAQDVIIVSDKSRVPEVFVRVKVGNQMLRTKFPQRSNNPKWGDEFTFVVAEPFEDNLVLSVEDHTAPNRDEPVGKAVILMNDIEKRIDDKPFHDRWVHLEDSISDAMDVDKAKKVKFATRLRYKAVLDGGYHVFDESMYNSSDLRPSSRKLWKPAIGVLELGILNANVFHSMKTREGKGTSDTYVVAKYGHKWVRSRTVINSMNPKYNEQYTWEVFDPATVLTICVFDNAHFAAGDGGNKRDQPIGKVRIRLSTLQTGRVYTHAYPLLVLQPTGLKKRGELHLAVRFTCTSVSSMLMKYTKPLLPKMHYILPLSTNQQEALKMQAINIIIVRLGRSEPPLRREVVDYLTDWKSQLFSMRRSKANFNRFTTVFSGALSVWKWMEQVCTWKTPVTTALVHVLYTMLVTFPEMILPTVFLYMAVIGMWNYRFKPRFPPHMDAKLSYADNVNSDELDEEFDTFPTVRAPDIVKMRYDRLRSVAGKVQSVAGDIAAQGERVQALLSWRDPRATAIFVTFCFIIAMALYITPFKLVALLSGYYFMRHPKLRHRIPSAPVNFFRRLPAMTDSML

>AtMCTP7

MMMSNLKLGVDVIGAHNLFPKDGQGTSNAYVELYFDGQKHRTTIKDRDLNPVWNESFFFNISDPSRLHYLNLEAQAYSHNRSTNGRSFLGKVSLSGTSFVPHSDAVVLHFPMERRGIFSRVRGELGLKVYITDEASLKSSAASNDHPDNLDPALPRAMNVEHRSDKRHVFYNLPNSAQEHQHQHPQGPNQSSSLAAEQDNHNEHHHHYVPKHQVDEMRSEPARPSKLVHAHSIASAQPADFALKETSPHLGGGRVVGGRVIHKDKTATSTYDLVERMYFLYVRVVKARELPIMDITGSVDPFVEVRVGNYKGITRHFEKRQHPEWNQVFAFAKERMQASVLEVVVKDKDLLKDDYVGFVRFDINDVPLRVPPDSPLAPQWYRLEDKKGEKIKGELMLAVWIGTQADEAFSDAWHSDAAMPVDCSPAISAVLRSKVYHAPRLWYVRVNVIEAQDLIPTDKTRFPDVYVKAQLGNQVMKTRPCQARTLGAVWNEDFLFVVAEPFEDHLVLTVEDRVAPGKDEIVGRTYIPLNTVEKRADDHMIHARWYNLERPVIVDVDQLKREKFSMRIHLRVCLEGGYHVLDESTHYSSDLRPSARPLWRQPIGVLELGILNAVGLHPMKTREGRGTSDTFCVGKYGQKWVRTRTMVDNLCPKYNEQYTWEVFDPATVLTVGVFDNGQLGEKGNRDVKIGKIRIRLSTLETGRIYTHSYPLLVLHPTGVKKMGELHMAVRFTCISFANMLYQYSKPLLPKMHYVRPFSVMQQDMLRHQAVNIVAARLGRAEPPLRKEIIEFMSDTDSHLWSMRKSKANFFRMMTVFSGVIAVGKWFSDICSWRNPITTVLVHVLFLMLVCLPELILPTMFLYMFLIGLWNYRFRPRYPPHMNTKISQAEAVHPDELDEEFDTFPTTRNPDMVRLRYDRLRSVAGRIQTVIGDLATQGERFQALLSWRDPRATAIFVILCFIAAIVFFITPIQIVVALAGFFTMRHPRFRHRLPSVPVNFFRRLPARTDSML

>AtMCTP11

MAVNGTGNGTGDGDFSLKETSPNIGNGGVNGGEKLTSSFDLVEAMHFLYARIVRARALPVNDSFVAVKIGSYKGRTKQILNSNPNPEFHETFAFTKTRLQGDILEVVVRNRDNPNEDDIVGKCKFDVAEIPTRVPPDSPLAPQWYRLEDRNGVKIGGEIMVSVWIGTQADEVFSEAWHSDSASVTGENVVNTRSKVYLSPRLWYLRVNVIEAQDLVLLHPNRINPEILIKGFLGNVVVRSRISQTKSVSPVWNEDMMFVAVEPFDDSLILSVEDKVGPREECLGRCEIKLSQVERRVLPGPVPSLWYNVEHIGETGEGRRFAGRIHLRVSLDGGYHVLDESIQYSSDYRASAKLLWTPPIGVLELGVLNATGLMPMKSRGGRGTTDAYCVAKYGTKWVRTRTIVDTFDPKWNEQYTWEVYDPYTVITIGVFDNLKLFGAGNENRLINDSRIGKIRIRLSTLVTSKIYTHSYPLMVLKPDGVKKMGEIQLAVRFTATSMMDMLQKYTEPLLPEMHYISPLSIYQLDSLRHQATHILCINLGRNEPALGRDVVEYMLDVGSNIWSLRRGRANFERLVSFFDGWIDAWKWFDEICKWKSPVTSVLVHIVCLFVVFLPKYCVFSMLLYCFVFGLYRFGLRPRHPPHMDIKLSKADSALPDELDEEFDVFPSSKSGDVLKRRYDRLRGIAGRMMIVLGDLATQGERVKSLLSWRDPRATSLFLTFCFVSCGVICFVSMKLLLTFLAFYVMRHPRVRVFDIPSIPQNFFRRLPSRADSIL

>AtMCTP13

MAANKDEFSVKQISPKLGGERGARNPYGPTSLHDLVEQMEFLYVDVIRAIKNSDVDPGPCDPVVEITLGNYKSSTKDLPVGPNMDWNQVFAFDKTKGDVLSVTLKDRLTNTVINKSNFKLASEIPTRAPPDARIAPQRYPLRNTKTGFYLMMSVWFGTQVDEVYPVAWFSDASEVSTCVINTRPKVYLAPRLCYVRVTIVSGHDLISTDRNRTPSVYVTATLGQVTLKTEVSSGTNPSWNKDLIFVASEPLEGTVYIRLIDRVDDQHEERIIGKLEKKLSEMTPLKVPSSAPALFYDIEVEPAGDSRRFASRLKMKLATDQAYHVAEESIQYSSDYRPFVKGLWPCLLGKLEIGILGATGLKGSDERKQGIDSYVVAKYGNKWARTRTVVNSVTPKWNEQYSWDDYEKCTVLTLGIYDNRQIFKEDQANDVPIGKVRISLNRVESDWIYACSYPILKLGSSGLKKMGELQLAVRFVYVAQGYARYSAPFRWLLPKAHYKSPLSVYQIEEMRAEAVKINCANLARTEPALRNEVVWDMLKPKTNTRYSTCDMRKVAALAFFDLFLYWPSLIVWLAIYLVVVPCIVLVGLSGLHKFLTRKFWNKRENPRSPLIVNDLKLWKLESPNLDELEEEFDSFPSSVSDVNILRMRYDRIRMVCQRPMILLGDAASQGERLYALLTFNGDDQLASFYCWLICVLVALCWYNIPMWLWSLYPIAYWLNFTPLRNDMPCGVSNFFRRLPTNEVLF

>AtMCTP1(FTIP1)

MAAKDGAKSQEDYKLKDMKPELGERWPHGGQRGGTGWIGSERAASTYDLVEQMFYLYVRVVKAKDLPPNPVTSNCDPYVEVKIGNYKGKTKHFEKRTNPEWNQVFAFSKDKVQSSTVEVFVRDKEMVTRDEYIGKVVFDMREVPTRVPPDSPLAPQWYRLEDRRGESKKRGEVMVAVWLGTQADEAFPDAWHSDASSVQGEGVQSVRSKVYVSPKLWYLRVNVIEAQDVEPSDRSQPPQAFVKVQVGNQILKTKLCPNKTTNPMWNEDLVFVAAEPFEEQFFLTVENKVTPAKDEVMGRLISPLSVFEKRLDHRAVHSKWYNLEKFGFGALEGDKRHELKFSSRIHLRVCLEGGYHVMDESTLYISDVKPTARQLWKSPIGILEVGILSAQGLSPMKTKDGKATTDPYCVAKYGQKWVRTRTIIDSSSPKWNEQYTWEVYDPCTVITLGVFDNCHLGGSEKSNSGAKVDSRIGKVRIRLSTLEADRIYTHSYPLLVLQTKGLKKMGEVQLAVRFTCLSLAHMIYLYGHPLLPKMHYLHPFTVNQLDSLRYQAMSIVAARLSRAEPPLRKENVEYMLDVDSHMWSMRRSKANFFRIVSVFAGLIAMSKWLGDVCYWKNPLTTILFHVLFFILICYPELILPTTFLYMFLIGLWNFRFRPRHPAHMDTKVSWAEAASPDELDEEFDTFPTSKGQDVVKMRYDRLRSVAGRIQMVVGDIATQGERFQALLSWRDPRATCLFVIFCLVAAMILYVTPFKIIALAGGMFWMRHPKFRSKMPSAPSNFFRKLPSKADCML

>AtMCTP5

MQKPGQNIDFALKETSPKIGAGSVTGDKLCSTYDLVEQMHYLYVRVVKAKELPGKDVTGSCDPYVEVKLGNYRGMTKHFEKRSNPEWKQVFAFSKERIQASILEVVVKDKDVVLDDLIGRIMFDLNEIPKRVPPDSPLAPQWYRLEDRHGRKVKGELMLAVWMGTQADEAFSDAWHSDAATVGPEGVTHIRSKVYLSPKLWYVRVNVIEAQDLIPHDKTKFPEVYVKAMLGNQTLRTRISQTKTLNPMWNEDLMFVVAEPFEEALILAVEDRVAPNKDETLGRCAIPLQNVQRRLDHRPLNSRWFNLEKHIMVEGEQKEIKFASRIHLRIFLEGGYHVLDESTHYSSDLRPTAKQLWKPSIGLLEVGIISAHGLMPMKSKDGKGTTDAYCVAKYGQKWIRTRTIVDSFTPKWNEQYTWEVFDTCTVITFGAFDNGHIPGGSGKDLRIGKVRIRLSTLEADRIYTHSYPLLVFHPSGIKKTGEIQLAVRFTCLSLINMLHMYSQPLLPKMHYIHPLSVLQLDSLRHQAMNIVSARLNRAEPPLRKEIVEYMLDVDSHMWSMRRSKANFFRIMNVLSGLIAVGKWFDQICNWRNPITTILIHVLFIILVLYPELILPTVFLYLFLIGIWNFRWRPRHPPHMDTRLSHADAVHPDELDEEFDTFPTSRSSEIVRMRYDRLRSIGGRVQTVIGDLATQGERFLSLLSWRDPRATTLFVLFCLIAAIVLYVTPFQVVALLAGIYVLRHPRFRHKLPSVPLNLFRRLPARSDSLL

>AtMCTP16

MATTRKLVVEVVDAKDLTPKDGHGTSSPYVVLDYYGQRRRTRTIVRDLNPVWNETLEFSLAKRPSHQLFTDVLELDMYHDKNFGQTRRNNFLGRIRLGSDQFVGQGEEALIYYPLEKKSLFNLVQGEIGLRVYYADEKPPPLKPTVAPLETVVEEKTEETKAEGPDESKPPPETNDIPAEVKETVKPPQPPPEESSPAEGPKPDEEASPPLQENATVGGEEPPASESDKNEAEAKPVEEPPQNQPDGEDIVLESEDTMSWASAPRSPLPEVIISRSVSGSIPETKNGPQPLRRSVSETASYTSEISDVSTIERSTFDLVEKMHYVFIRVVKARSLPTSGSPVTKISLSGTMIQSKPARKTSCFEWDQTFAFLRDSPDLSSSPILEISVWDSSTGIETSQFLGGICFDVSEIPLRDPPDSPLAPQWYRLEGGGAHNSDLMLATWTGTQADESFPDAWKTDTAGNVTARAKVYMSSKLWYLRATVIEAQDLLPPQLTAFKEASFQLKAQLGSQVQKTKSAVTRNGAPSWNEDLLFVAAEPFSDQLVFTLEYRTSKGPVTVGMARVPLSAIERRVDDRLVASRWLGLEDPNDEKRGNRSRVHIRLCFDGGYHVMDEAAHVCSDYRPTARQLWKPAVGIVELGIIGCKNLLPMKTVNGKGSTDAYTVAKYGSKWVRTRTVSDSLDPKWNEQYTWKVYDPCTVLTIGVFDSWGVYEVDGGKEATRQDLRIGKVRIRISTLETGKAYRNTYPLLMLVNGGVKKLGEIELAVRFVRTAPPLDFLHVYTQPLLPLMHHIKPLSLFQEDMLRNTAVKILAAHLSRSEPPLRPEIVRYMLDADTHTFSMRKVRANWLRIVNVVAGMVDVVRWVDDTRFWKNPTSTLLVHALVVMLIWFPDLIVPTLAFYLFVIGAWNYRFRSRAALPHFDPRLSLADAADRDELDEEFDVVPSNRPPEMVRLRYDKLRNVGARVQTILGEVAAQGEKMQALVTWRDPRATGIFVGLCFFVALVLYLVPTKMVAMASGFYYFRHPIFRDRKPSPVLNFFRRLPSLSDRLM

>AtMCTP2

MRNTTKLVVHVVDAQYLMPRDGQGSASPFVEVDFLNQLSKTRTVPKSLNPVWNQKLYFDYDQSVINQHNQHIEVSVYHERRPIPGRSFLGRVKISLCNIVYKDDQVYQRFTLEKKWLLSSVKGEIGLKFYISSSEEDQTFPLPSKPYTSPTQASASGTEEDTADSETEDSLKSFASAEEEDLADSVSECVEGKKSEEVKEPVQKLHRQEVFARPAPMQSIRLRSRENPHEAQKPMSRGANQLHPQNPNHLQSYGDTDLDDFKVKDMNLDLGERWPNPNAGERFTGTYDLVEQMFYLYVRVVKAKELPPGSITGGCDPYVEVKLGNYKGRTKIFDRKTTIPEWNQVFAFTKERIQSSVLEVFVKDKETLGRDDILGKVVFDLNEIPTRVPPNSPLAPQWYRLEDWRGEGKVVRGEIMLAVWMGTQADEAFPEAWHADSASVHGEGVFNIRSKVYVSPKLWYLRVNVIEAQDMIPSDRNRLPDVFVKASVGMQTLKTSICSIKTTNPLWKEDLVFVVAEPFEEQLVISVEDRVHTSKDEVIGKITLPMNVFEKRLDHRPVHSRWFNLDKYGTGVLEPDARRKEHKFSSRIHLRICLEGGYHVMDESTMYISDTRPTARQLWKQPVGMLEIGILGANGLVPMKLKDGRGSTNAYCVAKYGQKWVRTRTILDTLSPRWNEQYTWEVYDPCTVITLGVFDNSHLGSAQSGTADSRDARIGKVRIRLSTLEAHKIYTHSFPLLVLQPHGLKKTGDLQISVRFTTLSLANIIYNYGHPLLPKMHYLFPFTVNQVDGLRYQAMNIVSTRLGRAEPPLRKEVVEYMLDVDSHLWSMRRSKANFFRIMSLLSGYFLVGKWLEDVCNWRYPVTSVLVNVLFFILVMYPELILPTMFLYMFFIGLWNFRSRPRHPPHMDMKLSWAEAVGPDELDEEFDTFPTSRSQELVRLRYDRLRSVAGRIQTVVGDIAAQGERIQSLLSWRDPRATSLFILFCLAASVVLYAMPFKAIALASGLYYLRHPKFRSKLPSLPSNFFKRLPSSTDSLL

>Os06g41090(OsFTIP1)

MTMTGGHHHDAHHEDFQLKDTNPLLGEQWPKGAAGPARPAVGGGIAGWLGLEKPSSTYDLVEQMFFLYVRVVKAKDLPPNPITGSPMDPYVEVKLGNYKGTTKHYDRRANPEWDQVFAFSKSRVQSNVLEVYLKDKEMLGRDDYVGRVVFDLAEVPTRVPPDSPLAPQWYRLEERRVGGGGDGGGLKVRGELMLAVWIGTQADEAFPEAWHSDAATVRGEGVASVRSKAYVSPKLWYLRVNVIEAQDVQPQARGRAPEVFVKAQVGNQILKTSVVAAPTLNPRWNEDLVFVVAEPFEEQLLLTVEDRVTPRKDDLLGRAALPLALFEKRLDHRPFVQSRWFDLEKFGIGGAIEGETRRELRFASRVHVRACLEGAYHVMDESTMYISDTRPTARQLWKPPVGVLEVGILGAAGLQPMKNRDGRGTTDAYCVAKYGQKWVRTRTMLGTFSPTWNEQYTWEVFDPCTVITIGVFDNNHLGNGNGNGNNAGGGGGGSPPARDARVGKIRIRLSTLETDRVYTHAYPLIVLQPSGVKKMGELRLAVRFTCLSLMNMVHLYTQPLLPRMHYLHPFTVTQLDALRYQAMGIVAARLGRAEPPLRREVVEYMLDVESHMWSMRRSKANFFRAVSLFSGAAAAARWFADVCHWKNVATTALVHVLLLILVWYPELILPTVFLYMFMIGLWNYRRRPRHPPHMDTKMSWAEAVHPDELDEEFDTFPTSRQQDVVYMRYDRLRSVAGRIQTVVGDMATQGERLQSLLGWRDPRATCLFVVFCLVAAVVLYVTPFRVVALVAGLYLLRHPRFRSRLPAVPSNFFRRLPSRADSML

>Os05g30750(OsFTIP7)

MMQRPFRPEEYSLKETSPHLGGGAAGDKLTTTYDLVEQMQYLYVRVVKAKDLPSKDITGSCDPYVEVKLGNYKGTTRHFEKKTNPEWNQVFAFSKERIQSSVVEIIVKDKDFVKDDFIGRVLFDLNEVPKRVPPDSPLAPQWYRLEERNGHKVKGELMLAVWMGTQADEAFPEAWHSDAASIPGDGLASIRSKVYLTPKLWYLRVNVIEAQDLIPNDRTRFPDVYVKAMLGNQALRTRVSPSRTLNPMWNEDLMFVAAEPFEEHLILSVEDRIAPGKDDVLGRTIISLQHVPRRLDHKLLNSQWYNLEKHVIVDGEQKKETKFSSRIHLRICLEGGYHVLDESTHYSSDLRPTAKQLWKHSIGILELGILTAQGLLPMKTKDGRGTTDAYCVAKYGQKWVRTRTIIDSFTPKWNEQYTWEVYDPCTVITIGVFDNCHLNGGEKANGARDTRIGKVRIRLSTLETDRVYTHAYPLIVLTPAGVKKMGEVQLAVRFTCSSLLNMMHLYSQPLLPKMHYVHPLSVMQVDNLRRQATNIVSTRLSRAEPPLRKEIVEYMLDVDSHMWSMRKSKANFFRIMGVLSPLIAVAKWFDQICHWRNPLTTILIHILFVILVLYPELILPTIFLYLFLIGVWYYRWRPRQPPHMDTRLSHAESAHPDELDEEFDTFPTSRPPDIVRMRYDRLRSVAGRIQTVVGDLATQGERLQSLLSWRDPRATALFVTFCFVAAIVLYVTPFRVVVFLAGLYTLRHPRFRHKMPSVPLNFFRRLPARTDSML

>Os04g58720

MAAYKLGVEVASAHDLMPKDGQGSASACVELTFDGQRFRTAIKDKDLNPVWNERFYFNVSDPSNLPELALEAYVYNINRSIDGSRSFLGKVRIAGTSFVPFPDAVVMHYPLEKRGMFSRVKGELGLKVYITNDPSIKASNPLPAMDPVSNNPPPTPAEQIATDITGTNLSTTHEHRAEVKTLHTIAKEVQHQHHGHGHLPASFPDQPSKYAVDQMKPEPQQPKIVRMYSAASQQPMDYALKETSPFLGGGQVVGGRVIRAEKHASTYDLVERMQYLFVRVVKARDLPDMDVTGSLDPYVEVRVGNYRGITRHFEKQKNPEWNAVFAFSRDRMQATILEVVVKDKDLLKDDFVGLVRFDLNDVPMRVPPDSPLAPEWYRLVHKTGDKSRGELMLAVWIGTQADEAFPDAWHSDAATLEDPSAVTHMKSKVYHAPRLWYLRVNIIEAQDIAITDKTRYPDVFVRAQVGHQHGRTKPVQARNFNPFWNEDLMFVAAEPFEDHLILSLEDRVAPNKDEVLGRVIIPLTMIDRRADDRIVHGKWFNLEKPVLIDVDQLKKEKFSTRLHLRLCLDGGYHVLDESTNYSSDLRPTAKQLWKPSIGLLELGILGAQGIVPMKTRDGKGSSDTYCVAKYGSKWVRTRTIVNNPGPKFNEQYTWEVYDPATVLTVGVFDNGQLGEKGGEKTSSSKDAKIGKVRIRLSTLETGRVYTHSYPLLVLHPSGVKKMGELHLAIRFSSTSLVNMMYLYSRPLLPKMHYVRPIPVLQVDMLRHQAVQIVSARLSRMEPPLRKEVVEYMSDVDSHLWSMRRSKANFFRLMSVFSGLFAVSKWFNGVCSWRNPITTVLVHILFIMLVCFPELILPTVFLYMFLIGVWNYRYRPCYPPHMNTKISHAEAVHPDELDEEFDTFPTSRSPDVIRMRYDRLRSVAGRIQTVVGDIATQGERVQALLSWRDPRATAIFVLFCLIAAIVLYVTPLQVLAALAGFYVMRHPRFRYRLPSTPVNFFRRLPARTDSML

>Os07g30020

MMSNLKLGVEVTSAHDLLPKEQGTCNPYVEIEFDDQKFRTAIKERDINPVWNEQFYFNISDPSRLTEKDLEAYVYHANRASNSKTCLGKVRISGTSFVSHSDATPLHYPLEKRTILSRARGELGLRVFLTDDPSVRVSAPGQEFDFISTPTTAQEQVAANAIPNPFQETRADQVRQFQHLPKEQHQHRPQPMTAQPYYPESSYGQQQQKTYSAVGNKAEGPPPPVMRMYAQGPQQQPVEFQLKETSPTLGGGRVIGGRVIPGEKAGAYDLVEKMQYLFVRVVKARDLPHMDITGSLDPYVEVHLGNYKMKTRHFEKNQRPEWDEVFAFPREVMQSTSLEVIVKDKDFIRDDYVGRVSIDLNEVPLRVPPDSPLAPEWYRLVGKEGHRDKGELMLAVWYGTQADECFPSAIHAGSEPIDSHLHNYIRGKVYPVPRMWYVRVNVIGAQDIFPMENHIPDVFVKVRLGHQMLKTRPARSPTRNFMWNEEMMFVAAEPFEEDLIIQIEDRVAQNKDEVIGETMIPLARLPRRADHKPVLPAWFDLRRPGLIDLNQLKEDKFYAKVQLRICLEGGYHVLDESTQYCSDLRPTMKQLWKPPIGLLEVGILSANGLNPTKTKHERGSCDAYCVAKYGQKWVRTRTIVDNLNPRFNEQYTWDVFDHGTVLTIGLFDNCHISADSNHSSSPGHMDKPIGKVRIRLSTLETGRVYTHTYPLLVLHPSGVKKMGELHLAIRFTATSLLNVLFTYSRPLLPKMHYAQPLSIVQQEMLRHQAVQLVAQRLGRMEPPVRREVVEFMSDARSHLWSMRRSKANFFRLMQVFSGFIAAGKWFGDVCQWKNPVTTVLVHVLFIMLVFYPDLILPTIFLYMFLIGLWNYRFRPRFPPHMNTRISHADMTNPDELDEEFDTFPTSKSPDLVRMRYDRLRHVAGRIQTVVGDIATQGERLQSLLSWRDPRATSMFLLFCLLTAVILYVTPFQVIALCLGFFWMRHPRFRHKVPSAPVNFFRRLPAKTDSLL

>Os04g59520

MKVGVEILDASELAPKDGAGACNAFVEVEFDGQKQRTPTKPADRSPQWNHTLVFDVRDPSRLPSLPVDVSVHHDRSLTDHHATRLHTFLGRVRISAASLAPSPQDALLQRYPLEKRGLFSRVSGDIALRLYLIANDSPDPPPAPAVHHHQHQPPQSVSAEQPDSRPPPAFPHGEAQAQAQPPPPESESKGKTTHDHEPPRVFRSVPVQAPAPAASQPRRATLHAVAAPPPPPGQTVIMPRPPGPAPGPPPSAFGLVETKPPLPAKMGPRAAVAAAAKIASTYDMVEPMSYLYVSVVKARDLPNMDITGALDPYVEVRLGNFKGVTRHLEKNPNPVWRQVFAFSRDHLQSSQLEVVVKDKDVLKDDFVGRVVFDMTDIPNRVPPDSPLAPQWYRLADRSGEKIRHGEIMLAVWNGTQADEAFPEAWHSDAHSVSLDSLASTRSKVYYSPKLIYLKVVAIAAQDLIPAEKGRPLAPSIVKIQLGGQTRRTRSQGSANPMWNEEFLFVAAEPFDEPLVVTVEERVAAGRDEPVGRVIIPVAAPYVPRNDLAKSIEAKWFSLSRALTADEAAAAEATKLKSSFASKIHLRLSLETAYHVLDESTHYSSDLQPAAKKLRKSPIGILELGILGARNLAGGKSPYCVAKYGAKWVRTRTLVGTAAPRWNEQYTWEVFDLCTVVTVAVFDNCHLTGGGDAKDQRIGKVRVRLSTLETERVYTHFYPLMTLTPGGLKKTGELHLAVRFTCTAWANMLAMYGKPLLPKMHYTHPISVLQMDYLRFQAMQMVAARLGRAEPPLHREVVEYMLDVDSHMFSLRRSKANFKRMTSLFSGAVAVARWMDGICKWKNPVTTILVHVLFLILVCYPELILPTVFLYLFVIGVWNYRRRPRKPAHMDTALSHAEAEQVHPDELDEEFDTFPTSKPGDVVRMRYDRLRSVAGRVQTVVGDLATQGERAQALLSWRDPRATSIFVLLSLIIAVVLYVTPFQVVAVVVGLYLLRHPRFRSKQPSVPFNFYKRLPAKSDVLL

>Os05g35480

MKGAMPPRPFMMPGPGGPMPPPQQFGLVETRPPLAAVLRPRFNIPGLHPSAAAASAAGKIASTYDLVESMRFLYVHVVKAKDLPAVSAAGTIDPFVEVKLGNFKGTTPVLGGNHNPSWKQVFAFSATHLQAHVLEVAVKAKDLAGGDDLIGRVGFDLSEVPIRVPPDSPLAPQWYRLENKRGEKTRGEIMLSVWLGTQADEAFPDAWHSDAHAAAGPGAVASTRAKVYFSPKLVYLRVAAIGAQDLVPLDASRPANFCVKLQLAGQVRRTRPGAPPGTLNPIWNEEFMFVVSEPFDEPLFVTVEDRVGPGRDEPLGRIMLPLNAAMPRHDHFGKPVEPRWYSLARPSDDPDKKEGKFASKIQLRMSLDFGYHVLDESTYYSSDLQPSSKHTRKPSIGILELGILGARNLIPMKGKDGRTTDAYCVAKYGPKWVRTRTILNTLNPQWNEQYTWEVFDPCTVITVVVFDNNQIGKNGDARDESIGKVRIRLSTLETDRVYTHFYPLLALKPSGLKKTGELHLAVRFTCTAWVNMIALYGRPLLPKMHYTQPISVMQLDYLRHQAMQIVAARLSRAEPPLRREVVEYMLDVGSHMFSLRRSKANFYRITSLFCGFAATAKWYDGIRSWRNPITTVLVHMLFLILICYPELILPTIFLYMFMIGLWNYRYKPRHPPYMDTKLCHAEFTNPDELDEEFDSFPSSRPADIVRMRYDRLRSVGGRVQTVVGDLATQGERAHALLSWRDPRATAIFIFLSLVVAIVLYVTPFQVLLVIAMLYLLRHPRFRSRMPSVPFNFYRRLPAKSDMLL

>Os04g39680

MASLAGDGKSRPVNRPDGEPETTVSRVMACPYVFRAQAPPAMAKEAMATPTRPQVRETWPAGGGGGGGGWMGVGSGERVASAYDLVEQMHYLYVRVVRARGLTAAASTVAGGGGCNPYVEVRLGNYRGTTRHHERKAAPEWNQVFAFSRERVQASVLEVFVRDKDAVAAVARDGYVGRVAFDVGEAPVRVPPDSPLAPQWYRLEDVGGGGGRAVQGEVMLAVWVGTQADEAFADAWHAGAASVRGGGDGVAAVQSTRSKVYVTPKLWYLRISVLEAQDVVPGAVAGAGGDKGRHGEAFVVVKVQVGGVTLRTKPCCRPTSPSWNEELVFVVAEPFDEPAVLVIEARAAHPGKDEIVSRAVLPLTLFERRLDRRGAAAATHTQSQWFSLEPFVHRPRHSPEEPAFAGRVHLRACLDGAYHVMDEPAMYASDTRPTARQLWRPPIGVLEVGVLGAQGLPPMKTAADGGRGTTDAYCVAKYGHKWVRTRTVVDSSTPRWNEQYTWEVYDPCTVLTLAVFDNCNLGNGGGGGKDQRIGKVRIRLSTLEMDRVYTNAHRLVVLHPSGLRKNGDVCLAVRLTCLSLASVLRLYGEPLLPGAHYVHPFAVAQLDGLRRQAVGVVAARLGRAEPPLRREVVEYMLDAGSHLWSIRRSRANFLRATALLSGAAGAARWLADVCHWRSPATTILAHLLLVTFACFPELILPTAFLYASVAGAWSYRRRPRRPPQADAGLSCAEAAGADEFDEEADTFPTSRPDGVVRARYDRLRTVAGRIQAVVSDVATQGERVRSLLAWRDPRATAVFTAACLAAAVVAYATPPRVVALVAGLYLLRHPRFRSRMPSAAGNFFKRLPSRADTML

>Os02g57090

MAKAAEKLVVEVVAAHNLMPKDGQGSSSAYVEVEFEHQRRRTRARPKELNPVWNERLVFAVADPDDLPYRAIDVGVYNDRAASGGVAGGGGAAPHGRNFLGKVRVPAAGVPAPGEEVVPQLFTLEKRSLFSHIRGEITLKIYRTNSGEVVVKSKPEKPVKAVVSGPEVVAAPPVTGPKKQQQQQPVVAVQPPPPQPEAPMDILPPPAPVLMKPVMLADPYPASAVFSGPGDFSLKETRPRLGGGTTADKASATYDLVEQMQYLYVRVVRARGVAAVGETVAEVKLGNYRGVTPATAAHHWDQVFAFSKETIQSSFVEVFVRARGSDDHVGRVWFDLSEVPRRAPPDSTLAPQWHIMEDRKGERGAAEVMIAVWFGTQADEAFAEAWHSKAAGVHGYGPLGSIKSKVYVAPKLWYLRVSVIEAQDLIPMDKGPMAIGRYPELFVRAQVGSQMLRTRPAPVAANRGPSSPFWNEDLMFVVAEPFEEFLVLSLEDHVSPGRDDVLGRLVVPVSSIERRWDEKLVVSRWFGLDRGTGGGNVASGNTNRFGSRVHLRLSLDGGYHVLDEATAYSSDLRPTGKQLWQPHVGVLELGVLGATGLIPMKARDGRGATSDAYCVAKYGQKWIRTRTVVDSVCPRWNEQYTWEVFDPCTVITVGVFDNCHVDKPASGNTTLAVRDNCIGKVRIRLSTLETDRVYTHAYPLLMLHPSGVKKMGELHLAVRFCCGNAGNMFHAYVRPLLPKMHYIEPLLVRQVESLRFQATNVVAARLGRAEPPLGREVVEYMLDHRSHLWSMRRSKANFFRLVTVLSGPITIGRWFELVRSWNRPVHSCLAVFTFLVFVTMPELILPTAFLAMAFTGLWRYRVRSRHPPHMEMRLSHADAATVDELDEEFDTFPSSRGDVVRFRYDRLRSVAGRVQTVVGDIATQGERMQALLSWRDPRATVLFSIACVLAAVIAYTIPMKVLVGLWGLYAMRPPRFRSRMPSPLMNFFRRLPSKADSLL

>Os03g44890

MVEEGARRRVVVEVCNARNLMPKDGQGTASAYAVVDFDGQRRRTATRPRDLNPQWGERLEFLVHDPDAMCAETLELNLYNDKKAIAATGGGGRRGGTFLGKVKVAGASFSKAGDEVLVYYPLEKRSVFSQIKGEIGLKIWFVDEPPPPPPPAAPADGKADAAAEKKEAAEGGKEEKEKAPAAAAASAAEEKKPEAPAEEKKAEEAKKEEKKSAEADKKEEKDDKKKSPEKGKKDGEKPKEEGKAKDETKKEVAPVPPSPSKAPPPSPSKMELAAAGVAGDLEIRPQSAAERSMAASAGNASYDLVDRVPYLFVRLLKAKHHGGGDKQPLYAQLSIGTHAVKTRAATAAGEWDQVFAFHKDSLTATSLEVTVHEEAKKPAAEGEATPPDTNLGYVSFDLHEVPKRSPPDSALAPQWYTLEGHANDGTAACDVMLAVWVGTQVDEAFQEAWQSDSGGYLVHTRSKAYLSPKLWYLRLSVIQAQDLRLPAPPDAKAKPMGPAFPELYVKAQLGAQVFKTCRVALGSAATGTSNPSWNEDLLFVAAEPFDPFLTVVVEDIFSGQPVGQARVPLSTVHRRSDDRVEPPSRWLNLCGDEARPYAGRVHVRVCLEGGYHVLDEAANVASDVRAASKQLSKPPVGMLEVGIRGAANLVPMKIAKDGASGSTDAYVVLKYGPKWARTRTILDQFNPRWNEQYAWDVFDPCTVLTIAVFDNVRYRSAEASGDAGKLPKDARIGKLRIRLSTLDANRVYANTFALTAVHPVGVRKMGELELAIRFTCPSWLTLMQAYGSPLLPRMHYVKPLGPAQQDVLRHTAMRIVSGRLARSEPPLGPEVVQYLLDTDTHSWSMRRSKANWFRVVGCLSHVATAVRWANRVRTWTHPTTTVLVHALLVAVVLCPEMILPTVCLYLFLVLLWRYRARPREPTGMDPRLSHVDSVSPDELDEEFDGLPSARPADVVRMRYDRLRAVAGRAQTLLGDVAAQGERIEALLSWRDPRATAVFAVVCLLAALVMYAVPFKLLLLAMGFYYLRHPRFRGDMPSAGFNFFRRLPSNSDRVL

>Os01g40480

MAAGAPPPESGPPPPGPPGAMRVARRLAVEVVDARDLVPKDGLGTSSAFAVVDFDGQRKRTRTVPRDLSPQWHERLEFAVHDPAAMHAEALDVSLYHDRRFNPSGGGGGGGGKNHFLGRVRIYGSQFSRRGEEGIVYFPLEKRSLLSWIRGEVGLKIYYYDEAPPPPEDRPPEGGGGDNAPPPEVPPEAPRDLPDIPAPTEAAVEVQQPPVHPPVIIVEEAPPPMHHPMMPPMHGPHGPMMPPAVHGPHGPMAPPMHQHQHPQPQPEPEPEPEPDGGEAGYPPEVRKTRMATERVRIARHLSGGLGPEYYATSPRVISGRFVSTGDAVEPVQSSYDLVEPMRYLFVRVVKVRGIRACEGPYVKIQAGPHTLRSRPGRDVSGTGNPEWNQVFAINHAKPEPTLEISVWDGGAPSPIEAFLGGVCFDLSDVPVRDQPDGPLAPQWYRLEGGEPGMVTGDIMVAVWIGTQADEAFPEAWNTDAPYAAYTRSKVYQSPKLWYLRASVIEAQDLRVPAPPPGLPFDVRVKIQVGFQSARTRRSVASRSSGSAFAWEEDLMFVVSEPLDESLVVLVEDRSMIKEPALLGHATIPVNSVEQRLHERQLVASRWFSLEGGTSDIGIGPGGGPPGFYSGRLHLRLCLEGGYHVLDEAAHVCSDYRPTAKQLWRPPVGVLELGIIGACGLLPMKTKGGAKGSTDAYCVAKYGKKWVRTRTVTDSLNPRWNEQYTWQVYDPCTVLTVAVFDNWRMFAFAGAGDEQRQDYRIGKVRVRVSTLESNRAYTASYPLLVLLRSGLKKMGEVQLAVRFTSPAHLPDTWATYTSPLLPRMHYLRPIGVAQQEALRAAAVRVVAAWLERSEPPLGREVVRHMLDVDAHTWSVRRAKANWFRIMGVLAWAVGLARWLDSVQRWRSPPTTVLVHVLYLVLVWYPELAVPTASLYVFLIGVWYYRFRPRGPAGMDARLSQADTVEADDLEEEFDAVPPPEVLRARYERLRTLAGRVQRVMGDVAAQGERVQALVSWRDPRASRIFVGVCLAVAVALYAMPSKMVAVAGGFYYLRHPMFRDPMPPAAVNFFRRLPSLSDRML

>Os02g44490

MGDVLPRSYARPAGMLPPNNEFGIREITPGLACSGPGGAYELVERMEYLYVRVVKARGLKWSGEFDPFAELRLGGYSCITRHVEKTASPEWDDVFAFSRERIHAPFLDVLVRGRGFAKDDYVGSTRLDLGILPDAPASVQPDSSPAPQWYPVFDKKGEFRGEVMMAVWFGTQKDSYFDSAVHADAAFPVDDKLAAHIKHIRYDVPRLCYVRVKFTEVRDIVFADKARVGEVFVRSRILGQVHRTRTSMDHRWKDEENGHLFVAAAPFKDYLNMSVVGVKNGKEEVIGHVNVLLDSFERRCDARPISPRWFSLMQPEGAAKIDKYSAKISVVLCLECGYKVLSEPVHYLSDVRPAAREQERERKCIGLVELGIREAILTATRTRDGRGSCDAYCVAKYGVKWYRTRTVTDSISPRFHQQYHWEVHDHCTVLTVAVFHNSQIGDKGGLVAGDPVKDVLLGKVRIRLSTLETGRTYAYAYPLMSLHGGGVKKMGELRLAVRFSSTSTLGLFQTYAQPHLPPMHYHRPLTVVQQEMLRREAVTIIAHRMGRMDPPLRRECVEHLCESHALRWSMRRSKAHFFRLAEALEPLSAASAWFYHVCRWTNPVTTVAVHVIFTMLVCYPRLVLPTFFLYKFMLGMRNYLRRPKHPWHVDMRVSHADTAHPDELDEEFDEFPTARPPEVVRMRYDKLRSLNARIQEIVGDIATHAERARCVMTWRDPRATGLYLLGCLCLAVITFSVPFQAVALLTGFYLMRHPILRQRLPDVVANFFRRLPCKVDCLL

>Os07g07070

MAAAETVRKLVVEVVEARNLLPKDGTGTSSPYARVDFDGQRRKTHTVPRELNPAWNEALEFNFAGVAGDVVVGGEPLEVAVLHDVRVGPSRRSNFLGRVRLDARQFVRKGEEALIYFPLEKKGFFNWVRGEIGLRVYYLDEPVAPPPPPPEPPAADPAPAEAAPDAPPADADAAPEAPEKAEEALPAASGGDDGATDKPPETDAAAAAATSAPEEEAPVMASEAVAASAEAAPEEEQILTPPPPPTPTPTPMLRQVPVPARPPPPPPEAPVERSKHDLVDKMPYLFVRVVRARGLPAGAHPHVRVAAGGRHASTREARRGAFFEWDQTFAFVRDPGATDSPGPTLEVSVWDLPPDADVSDADDRHFLGGLCFDTADVHARDPPDGPLATQWYRLEGGRRLAGADLMVATWAGTQADEAFADAWKADSPASSVAAAAASRAKVYVSPKLWLLRLTVIEAQDTLTAPPPRDAGIAVRGTLGFQSLKTRTAPVARNGGPSWNEDLLFVAAEPHADGDDCLVISLEVRHGKDAFPVGSASISLATIERRVDDRKVASKWIDLLPSDEAMKKVGKKAAMHMHGGRLHVRVCLDGGYHVADEQPYASSDFRPSARQLWRPPIGVVELGIVGCKGLLPMRTADGKGCTDAYAVAKYGPKWARTRTISDSFDPAWNEQYTWPVYDPCTVLTVGVFDDPPPPSPSQLPDGAKDAAAFSRPMGKVRIRLSTLESGRVYRGVYPLIMMLPTGAKRMGDVELAIRFAASASALDVLHMYGRPALPPMHHLRPIPAASRDALRLSAARISAAHLARSEPPLRREAATWMLDAAEPRGFSMRKLRANWTRAVAALSWVSDAARWAEDTRSWRNPTATALAHAVLVLLAWHPDLVVPTLTLHVAAVGVWKYRRRPRAPAPHPCVRASMAEAADREELDEEFDAIPSSRPPEVVRARYDRARMVGARLQAMVGDVATQAERLQALVSWRDPRATGVFVALCVFVAMALYVVPIKVVAVVAGFYYLRHPMFRDRMPAPAINFFRRLPSMSERIM

>GhMCTP1-A

MAVEKVDFSLKETSPNIGGDRVSGGEKLTSSFDLVEKMEFLFVRIVRARDLPLKAVNGIIDPYVEIKIGNYNATTKYFEKKPDFEWNQVFAFGQDQLQATTMEITVRDKELIIGDNMIGKITVALHEVPPCLPPDSPLASQWYKLEDKNGFTLRKGELMLAMWYSTQADRVFPDAWHSDSAIVSGESLLNTRSKAYLSPRLWYLRVNVIQARDLVPGRRDRNPQVYVKAVVGDVILRTRVSPDKNVNPKWNEDLMFVVAEPFFDPLIVTVEDRLENNTVRCLGKCVIRLSNVEQRLLPLPADPLWYNLEDIVFEDGMEKEVNFFSKLNMCVSLDGGYHVFDESVQIGSDYRPTAKMLWTAMIGVLELGIINASDLQPMKLRDGRETTDAYCVAKYGPKWVKTRTVVDSFDPKWNEQYSWDVYDPYTMLTIGVFDDCHLHGGDAVAVGDGKDPSFGKVRIRLSTLATNKIYTYSYPLLVLQPNGAKKMGELQLAIRFTCSSYLSLFLVYTMNPLLPHMHHIYPLSIYQLDILRKQAVRILCWSLSRTEPPLRQEVVEYMLDGGSPMWSLRKAKANFQRVLATFKCFSDARQWFDEIRKWNNSAATVLVLAIYCIIVLKPDLILPTVTLYSIQVMILQWRKRPRRPTHIDVNLSVAGSVTADELDEELDTFPSSRQFDVLRMRYDRLRSIAGRVVTVISDIATQVERFHSLLNWRDPRITVTFLVCCLVGSFMLYYLISLKVFLIFGGFYVTRPPFLGKDMLNAPQNFFSRLPTKADYML

>GhMCTP2-A

MPENFSLKETKPNIGGGRVSGGREKLTSSFDLVEVTHFLFVRVVKARDLACSNATVGGGVCHPFVEIKIGNYKGSTKYLEMNKPNPEWNQVFAFTKDRIQSLSVEITVREKEFVNDEFIGKIAIDMSDIPTRVPPDSPLAPQWYKLEAEANSSVGELMLILWFGTQADEVFIDAWHSDVASVSGDSITNTRSKVYLAPRLWYLRVNIIEAQDLVVPGDKNRIPEVYVKAALGNVKLRTRVSADKSLNPRWNEDLMFVAAEPFYDHLVLTVVDKNNEEEISLGRCMVHLSEAYIRWLPEPVSAKWYNVEGGGDVVEELKFASKLNMRISLDGGYHVFDESVDCSSDYRATFKGLWPPAIGVLELGIIGASVLVPMKSRDGRETTDAYCVAKYGPKWARTRTVVNSFSPKWNEQYTWEVYDPYTVLFIGIFDNCHLNGEKGPNPKDPSIGKLRIRLSTLSTNRVYTYSYPLIALQPSGVKKMGEIQLALRFTSPSYTSLLAAYARPLFPKMHYIHPLSVYQLDSLRQQAIGILCSRLSSAKPPLRTEVTGCMLDAGYQLWSPRKAKANTERLIDAVHVITEAWKWFDTIKKWNNPAANVLVIVLYLIVVFYPSLVLPTLLIYCFLIGIWQYRKRPRDPTHIDIKLSLADSTNAEEWDEEFDTFPSSKQGDVLRMRYDRLRSMAGKVMVMVGDLATQGERLTALWSWQDPVASAIFLAFCVMAAVVFCSGLVPPRCILVMVGLSVMRPPSFGIDIPCAPQNVFGRLPTKTDCML

>GhMCTP2-D

MPENFSLKETKPNIGGGRVSGGREKLTSSFDLVEVTHFLFVRVVKARDLACSNATVGGGVCHTFVEIKIGNYKGSTKYLEMNKRNPEWNQVFAFTKDRIQSLSVEITVREKEFVNDEFIGKIAIDMSDIPTRVPPDSPLAPQWYKLEAEANSSVGELMMILWFGTQADQVFIDAWHSDVASVSGDSITNTRSKVYLSPRLWYLRVNIIEAQDLVVPGDKNRIPEVYVKAALGNVKLRTRVSADKSLNPRWNEDLMFVAVEPFYDHLVLTVVDKNNEEISLGRCMVHLSEAYIRWLPEPVGAKWYNLEGGGDVVEELKFASKLNMRISLDGGYHVFDESIDCSSDYRATFKGLWPPAIGVLELGIIGASVLVPMKSRDGRETTDAYCVAKYGPKWVRTRTVVNSFSPKWNEQYTWEVYDPYTVLIIGIFDNCHLNGEKGPNPKDPSIGKLRIRLSTLSTNRVYTYSYPLIALQPSGVKKMGEIQLALRFTSPSYTSLLAAYARPLFPKMHYIHPLSVYQLDSLRQQAIGILCSRLSSAEPPLRTEVTGFMLDAGYQLWSPRKAKANTERLMDAVHVITEAWKWFDTIKKWNNPAANVLVIVLYLVVVFYPSLVLPTLLIYCFLIGIWQYRKRPRDPTHVNIKLSLADSTNADEWDEEFDTFPSSKQGEVLRMRYDRLRSMAGKVMVMVGDLATQGERLTALWSWQDPVASAIFLALCVMAAVVFCSGLVPPRCILVMVGMFVMRPPSFGIGIPCAPQNVFGRLPTKTDCML

>GhMCTP3-A

MNKLVVEVVDAYDLMPKDDQGSSSPFVEVEFDGQRQRTQTKHKDLNPSWHESLVFYINQPGDLEYKTIDVTVYNDREGNHGHHRNFLGRVKISGASVPSSESGSSVQHYPLDKRGLFSNIKGEIALKLYQVRDELPREQVQRAAPASAVAENEETGRFQESQFQETPFQEINNVNNFAEEIKVEEKKKKKNEPEVRTFHSIGKVPEVRTFHSVGTGTGGPPPAPPPMKEKPPAVEIRADFAKAAAPAASVMHMQMPRQNPDYLLVETRPPVAARLRYRGGDKTLTTYDLVEQMHYLYVNVVKAKDLPVMDMSGSLDPYVEVKLGNYKGQTKHLEKNQNPVWHQIFAFSKERVQSNLLEVVVKDKDFGKDDFVGKIVFDVMEIPLRVPPDSPLAPQWYRLADKKGDKVKGEIMLAVWMGTQADESFPEAWHSDAHNISHSNLANTRSKVYFSPKLYYLRVHVMEAQDLVPHDKGRLPDPYVKVVLGNQIRPTKVIQRTIHPVWDEQLLFVASEPFEDYIIISVDDRIGPGKDEILGRAMIPVREVPQRLETGKPPDPRWFNLLKHSKAEEEGEKKKEKFSSKILLLIFLEAGYHVLDESTHFSSDLQPSSKFLRKQSIGILELGILSAKNLQPMKMKDGKLTDAYCVAKYGNKWVRTRTLLDTLYPRWNEQYTWEVHDPCTVITIGVFDNSHTNGSKDDARDQRIGKVRVRLSTLEIDRVYTHYYPLLVLTPGGLKKNGELQLALRFTCTAWVNMVAQYGRPLLPKMHYVQPIPVMNIDWLRHQAMQIVAARLQRAEPPLRREVVEYMLDVDYHMWSLRRSKANFNRIMSLLSGVTAICKWFNDICYWRNPITTCLAHILFLILTKTSPTYGCSAFTSGSYTPR

>GhMCTP3-D

MSKLVVEVVDAYDLMPKDDQGSSSPFVEIEFDGQRQRTQTKHKDLNPSWHESLVFDISQPGDLEYKTIDVTVYNDRKGNHGHHRNFLGRVKISGASVPSSESGSSVQHYPLDKRGLFSNIKGEIALKLYQVCDELPREQVQRAVPASVVAENEETGRFQESQFNETPFQEINGGRFQESQFQETPFQEINNVNNFDEEIKVDEKKKKKKKKEPEVRTFHSIGKEPEVRTFHSVGTGTGGPPPAPPPMKEKPPAVEIRADFAKAAAPAASVMHMQMPRQNPDYLLVETRPPVAARLRYRGGDKTLTTYDLVEQMHYLYVNVVKAKDLPVMDMSGSLDPYVEVKLGNYKGQTKHLEKNQNPVWHQIFAFSKERVQSNLLEVVVKDKDFGKDDFVGKIVFDVMEIPLRVPPDSPLAPQWYRLADKKGDKVKGEIMLAVWMGTQADESFPEAWHSDAHNISHSNLANTRSKVYFSPKLYYLRVHVMEAQDLVPHDKGRLPDPYVKVVLGNQIRPTKVIQRTIHPVWDDQLMFVASEPFEDYIIISVDDRIGPGKDEILGRAMIPVREVPQRLETGKPPDPRWFNLLKPSKAEEEGEKKKEKFSSKILLRIFLEAGYHVLDESTHFSSDLQPSSKFLRKQSIGILELGILSAKNLQPMKMKDGKLTDAYCVAKYGNKWVRTRTLLDTLSPRWNEQYTWEVHDPCTVITIGVFDNSHTNGSKDDARDQRIGKVRVRLSTLEIDRVYTHYYPLLVLTPGGLKKNGELQLALRFTCTAWVNMVAQYGRPLLPKMHYVQPIPVMNIDWLRHQAMQIVAARLQRAEPPLRQEVVEYMLDVDYHMWSLRRSKANFNRIMSLLSGVTAICKWFNDICYWRNPITTCLTKTSPTYGCSAFTSGSYTPR

>GhMCTP4-A

MQKPPPQSVDFALKQTSPNIGAGAVTVDKLSCTYDLVEQMQYLYVLVVKAKDLPGKDVTGSCDPYVEVKLGNYKGVTKHFEKKSNPEWNQVFGFSKERIQASILEVFVKDKDVVVDDLIGRVVFDVNEIPKRVPPDSPLAPQWYRLEDRKGNKPKGELMLAVWMGTQADEAFPDAWHSDAASVGPDGVTNIRSKVYLSPKLWYVRVNVIEAQDLVPSDKGRFPEVFVKAVLRNQALRTRISPSKTINPMWNEDLMFVVAEPFEEPLFLSVEDRVGSNKDETLGKCVIPLQVVQRRLDHKPVNSRWHNLEKHVIVDGEKKEMKFASRIHLRICLEGGYHVLDESTHCSSDLRPTAKQLWRPNIGILELGILSAHGLMPMKTKDGRGTTDAYCVAKYGQKWIRTRTIVDNFMPRWNEQYTWEVFDPCTVITIGVFDNGHIHGGAGGAKDARIGKVRIRLSTLEANRVYTHSYPLLVLHSSGVKKTGEVQLAVRFTCSTLINMLHMYSHPLLPKMHYIHPLSVIQLDSLRHQAVQMVSVRLSRAEPPLRKEVVEYILDVDSHMWSMRRSKANFFRIMGVLSGLIAVGKWFDQICNWRNPITTILIHILFIILVLYPELILPTVFLYLFLIGIWNYRWRPLHPPHMDTRVSHAVAAHPDELDEEFDTFPTSRPSDIIRMRYDRLRSIGGRVQTVIGDLATQGERLQSLLSWRDPRATTLFVTFCLIAAIVLYVTPFQVVALLIRLYALRHPRFRHKLPSVPLNFFRRLPARSDSML

>GhMCTP4-D

MQKPPPQSVDFALKQTSPNIGAGAVTGDKLSCTYDLVEQMQYLYVRVVKAKDLPGKDVTGSCDPYVEVKLGNYKGVTKHFEKKSNPEWNQVFGFSKERIQASILEVFVKDKDVVVDDLIGRVVFDVNEIPKRVPPDSPLAPQWYRLEDRKGNKAKGELMLAVWMGTQADEAFPDAWHSDAASVGPDGVTNIRSKVYLSPKLWYVRVNVIEAQDLVPSDKGRFPEVFVKAVLGNQALRTRISPSKTINPMWNEDLIFVVAEPFEEPLFLSVEDRVGSNKDETLGKCVIPLQVVQRRLDHKPVNSRWHNLEKHVIVDGEKKEMKFASRIHLRICLEGGYHVLDESTHCSSDLRPTAKQLWRPNIGILELGILSAHGLMPMKTKDGRGTTDAYCVAKYGQKWIRTRTIVDNFMPRWNEQYTWEVFDPCTVITIGVFDNGHIHGGAGGAKDARIGKVRIRLSTLEADRVYTHSYPLLVLHSSGVKKTGEVQLAVRFTCSTLINMLHMYSHPLLPKMHYIHPLSVIQLDSLRHQAMQIVSVRLSRAEPPLRKEVVEYMLDVDSHMWSMRRSKANFFRIMGVLSGLIAVGKWFDQLCNWRNPITTILIHILFIILVLYPELILPTVFLYLFLIGIWNYRWRPLHPPHMDTRLSHADAAHPDELDEEFDTFPTSRPSDIVRMRYDRLRSIGGRVQTVIGDLATQGERLQSLLSWRDPRATTLFVTFCLIAAIVLYVTPFQVVALLIGLYALRHPRFRHKLPSVPLNFFRRLPARSDSML

>GhMCTP5-A

MTTSAQQLPPQPPNTVRKVIVEVVDARDLLPKDGQGSSSPYVIADFDGQKKRTSTKYRDLNPVWNEALEFTVSDPDNMEVEELEIEVFNDKKFGNGSGRKNHFLGRVKLYGSQFAKRGEEGLVYFPLEKKSVFSWIRGEIGLRIYYYDEIVEDQPPPEDSPPQQQQQPPQTEDTKPTPGLVVVEEGRIFEAPPAHMEYPHGVNGYAHGAVPCYNSPPVVVVEESPPDVVHVHEEQPPPAEPTAMPMQPHMAPGIPVPEEHFPVPEVRKMQSSRGERVRVLKRPNGDYSPREIGCNKTQGDNATAAGVGGPERIHPYGLVEPMHYLFVKIVKARRLAPNEAPYVKIRTSNDYVKTKPTIYRPGEPTDSPEWGKVFYFGYNKQESANATLEISVWDSPTENFLGGVCFDLSDVPVRESPDSPLAPQWYRLESGAVDQNSGSVSGDIQLAVWIGTQNDDAFPEAMSSDAPYVAHTRSKVYQSPKLWYLRLTIIEAQDLQIAPNLPPLTVPEIRVKAQLGFQSVRSRRGNMNNHSMSVHWNEDLIFVAGEPLEDSLILLVEDRTNKEVSLLGHVMIPLISIEQRIDERRVASKWLGLEGGAGGGGGGPYCGRIHLRLCLEGGYHVLDEAAHVCSDFRPTAKQLWKPPIGILELGILGARGLLPMKTKGGGKGSTDAYCVAKYGKKWVRTRTVTDSFDPRWNEQYTWQVYDPCTVLTVGVFDNWRMFADVSEAKPDSRIGKIRIRISMLESNRVYTNSYPLLILTRMGLKKMGEIELAVRFACPSLLPDTCSAYGQPLLPRMHYLRPLGVAQQEALRGAATKMVAQWLARSEPPLGQEVVKYMLDADSHTWSMRKSKANWFRIVAVLAWAVGLAKWLDNIRRWKNPVTTVLVHVLYLVLVWYPDLVMPTGSLYVVLIGVWYYRFRPKIPAGMDIRLSQAETVDPDEIDEEFDTIPSSKPPEIIRARYDRLRILAGRVQTVLGDFATQGERVQALVSWRDPRATKLFIGVCLAITFILYVVPPKMVAVALGFYYLRHPMFRDPMPPASLNFFRRLPSLSDRLM

>GhMCTP5-D

MTTSSQQLPPQLPNTVRKVIVEVVDARDLLPKDGQGSSSPYVIVDFDGQKKKTSTKYRDLNPVWNEALEFTVSDPDNMDVEELEIEVCNDKRFGNGSGRKNHFLGRVKLYGSQFAKRGEEGLVYFPLEKKSVFSWIRGEIGLRIYYYDEIVEDQPPPEDPPPQQQQQPSQTEDTKPTPGLVVVEEGRIFEVPPAHMEYPHGVNGYTHGAAPCYPSSPVVVVEESSPDVVHVHEELPTPAKPTAMPMQPHMAPGISVSEEHFPVPEVRKMQSSRGERVRVLKRPNGDYSPREIVCNKTQGDNATVAGVGGAEKIHPYGLVEPMHYLFVKIVKARRLAPNEAPYVKIRTSNDYVKTKPTIYRPGEPTESPEWGKVFYFGYNKQESANATLEISVWDSPTENFLGGVCFDLSDVPVRESPDSPLAPQWYRLESGAVDQNSHSVSGDIQLAVWIGTQNDDAFSEAMSSDAPYVAHTRSKVYQSPKLWYLRLTIIEAQDLQIAPNLPPLTVPEIRVKAQLGFQSVRSRRGNMNNHSMSVHWNEDVIFVAGEPLEDSLILLVEDRTNKDVSLLGHVMIPLISIEQRIDERRVASKWIGLEGGAGGGGGGGGPYCGRIHLRLCLEGGYHVLDEAAHVCSDFRPTAKQLWKPPIGILELGILGACGLLPMKNKGGGKGSTDAYCVAKYGKKWVRTRTVMDSFDPRWNEQYTWQVYDPCTVLTVGVFDNWRMFADVSEDKPDSRIGKIRIRISMLESNKVYTNSYPLLILTRMGLKKMGEIELAVRFACPSLLPDTCSAYGQPLLPRMHYLRPLGVAQQEALRGAATKMVAEWLARSEPPLGQEVVKYMLDADSHTWSMRKSKANWFRIVAVLAWAVGLAKWLDNIRRWKNPVTTVLVHVLYLVLVWYPDLVMPTGSLYVVLIGVWYYRFRPKIPAGMDIRLSQAETVDPDEIDEEFDTIPSSKPPEIIRARYDRLRILAGRVQTVLGDFATQGERVQALVSWRDPRATKLFIGVCLAITFILYVVPPKMVAVALGFYYLRHPMFRDPMPPASLNFFRRLPSLSDRLM

>GhMCTP6-A

MNGTVATGKEKLVVEVIAAHNLMPKMGKGRLRRSWRWSSRTKGIGLRAVEVNVFNERRSSNSRNFLGKVRVSGSSIAKEGEEGPQMYTLDKRSLFSHIRGEITLKLYVSTREEVKQVGIDNNGVMGSGSASVSTSGFSKKKMQQQNPPLVVQQQQLVQENKPRLQGQNHAKPIEPKPGELKPVVITTGPGPAVPVSSAAGGAGAGLSLFSTGSNEFSLKETSPHLGGGPLNKDKTSSTYDLVEQMQYLYVRVVKARDISFFGGGGEIMAEVKLGNYRGVTKRVSSNHADWDQVFAFSKDCIQSSMVEVVVKEGNKDDYLGLVWFDLNEVPRRVPPDSPLAPQWHRMEDKKGDKSKGEVMVSIWFGTQADEAFAEAWHSKAANVHFDGLCSIKSKVYLSPKLWYLRVSVIEAQDVLGEKGSTLLRYPELSAKVQVGNQVSRTRISGVSPNRSLSNPFWNEDFMFVVAEPFEDYLLVSVEDRVGPGRDEVVGRVLLPVMAVERRTDDKQVVSRWFNLDNHFGNPAETKLVTGFGSRIHLRVSLDGGYHVLDEATMYSSDVRPTAKQLWKPHIGVLEMGILGATGLMPVKVKEGKGGTTDAYCVAKYGQKWVRTRTVVDSLSPKWNEQYTWEVFDPCTVITIGVFDNCRTDKNIITNGVPRDSRIGKVRIRLSTLESDRVYTHSYPLLMLHPSGVKKMGELHLAVRFSCANMGNMLHMYTLPLLPKMHYVQPMSVNQLESLRYQAMNVVASRLSRAEPPLGREVVEYMLDHDSHMWSMRRSKANFFRLMSVISGLVAMNSWFESMRNWSKPVYSSLFVATFLILVALPELLIPATLLYMATMGLWRYRFRPRHPPHMDTRLSHAESVYPDELDEEFDSFPTSRSVDVVKMRYDRLRSVAGRIQTVVGDMATQGERFQALLSWRDPRATFLFVILCLFAAVGFYAVPLKVVVALWGNYALRPPRFRSKLP

>GhMCTP6-D

MNGTVATGKEKLVVEVIAAHNLMPKDGEGSSSPFVEVEFENQRHRTQVKRKDLNPIWNEKLVFHIKDVADLPYRAVEFNVFNERRSSNSRNFLGKVRVSGSSIAKEGEEGPQMYTLDKRSLFSHIRGEITLKLYVSTREEVKQVGIDNNGVMGTGSASVSTSGFSKKKMQQQNPALVVQQQQLVQENKPRLQGQIHAKPVEPKPGELKPVVITTGLGPAVPVSSAGGGAGAGLSLFSTGSNEFSLKETSPHLGGGPLNKDKTSSTYDLVEQMQYLYVRVVKARDISFFGGGGEIMAEVKLGNYRGVTKRVSSNHADWDQVFAFSKDCIQSSMVEVVVKEGNKDDYLGRVWFDLNEVPRRVPPDSPLAPQWHRMEDKKGDKSKGEVMVSIWFGTQADEAFAEAWHSKAANVHFDGLCSIKSKVYLSPKLWYLRVSVIEAQDVLGEKGSTLLRYPELSAKVQVGNQVSRTRISGVSPNRSLSNPFWNEDFMFVVAEPFEDYLLVSVEDRVGPGRDEVVGRVLLPVMAVERRTDDKQVVSRWFNLDNHFGNPAETKLVTGFGSRIHLRVSLDGGYHVLDEATMYSSDVRPTAKQLWKAHIGVLEMGILGATGLMPVKVKEGKGGTTDAYCVAKYGQKWVRTRTVVDSLSPKWNEQYTWEVFDPCTVITIGVFDNCRTDKNIITNGVPRDSRIGKVRIRLSTLESDRVYTHSYPLLMLHPSGVKKMGELHLAVRFSCANMGNMLHMYTLPLLPKMHYVQPMSVNQLESLRYQAMNVVASRLSRAEPPLGREVVEYMLDHDSHMWSMRRSKANFFRLMSVISGLVAMNRWFESMRNWSKPVYSSLFVATFLILVALPELIIPATLLYMATMGLWRYRFRPCHPPHMDTRLSHAESVYPDELDEEFDSFPTSRSVDVVKMRYDRLRSVAGRVQTVVGDMATQGERFQALLSWRDPRATFLFVILCLFAAVGFYAVPLKVVVALWGTYALRPPRFRSKLPCRALSFFRRLPTKADSLL

>GhMCTP7-A

MSSLKLGVEVVGAHDLLAKDGQGSFCAFVELEFDGQRCRTTTKEKDLNPVWNESFYFNVSDPNKLSSLPLEAFVYNHNKANNAKTCLGKVRLTAPSFVNYSDAVVLHYPLEKRSIFSRAKGELGLKVFVTDDPSIKPSNPLPAMEPDVVPKSISKEKNGKRHTFFHLPNAKQPQQQQQKVTPAPPQQQMNYGVHEMKSGPQAFPGSSSQPFDYALKETSPFLGGGRIVGGRVIRGDRPTSTYDLVEQMRFLFVRVVKARDLPSKDATGSLNPYVEVKVGNYKGITKHYEKKQNPEWNQVFAFSRETVQSSVLEVVVKDKDLVKDDSVGFVRIDLHEVPTRVPPDSPLAPEWYRLEDKKGEKKKGELMLAVWYGTQADEAFPDAWHSDAIAPGDSTSVSPTYIRSKVYHSPRLWYVRVNVIEAQDLVPADKSRFPDAYVKVQIGNQILKTKPVQTRNMNPIWNEEFMFVAAEPFEDHLTFTVEDHAGPNKDETIGRTVIALNSIDRRADDRFVRTRWYNLEKSLSDAMDSDRAKKDKFHSRLHVCVCLDGGYHVLDESTQYSSDLRPTAKQLWKSSIGVLELGILNADGLQPMKTRDGKGTSDTYCVAKYGHKWVRTRTIVNSLNPKYNEQYTWEVYDTATVLTVGVFDNSQIGGSDNSRDNKIGKVRIRISTLETGRVYTHCYPLLVLHPSGVKKLGELHLAIRFSSTSMVNMMSQYSLPLLPKMHYKRPLSVIQQDILRHQAVNIVAARLGRAEPPLRREVVEYMSDANAHLWSMRRSKANFLRLTSVFSGLFSAGKWFGEVCTWKNPITTVLVHTLFAMLVCFPELILPTVFLYMFLIGVWNYRYRPRYPPHMNTSLSCADAVSPDELDEEFETFPASKSSDIVRMRYDRLRSVAGRIQTVVGDIATQGERLQALLSWRDPRATTIFVLLCLVAAIVLYVTPFQMIALLAGFYVMRHPRFRHKTPSAPINFFRRLPARTDSML

>GhMCTP7-D

MSSLKLGVEVVGAHDLLAKDGRGSFCPFVELEFDGQRCRTTTKEKDLSPVWNESFYFNVSDPNKLSSLPLEAFVYNHNKANNAKTCLGKVRLTAPSFVNYSDAVVLHYPLEKRSILSRAKGELGLKVFVTDDPTIKPSNPLPAMDPDVVPKSIPKEKNGKRHTFFHLPNAKQPQQQQQKVTPAPPQQQMNYGVHEMKSGPQALKAVNMFPGSSSQPFDYALKETSPFLGGGRIVGGRVIRGDRPTSTYDLVEQMRFLFIRVVKARDLPSKDATGSLDPYVEVKVGNYKGITKHYEKKQNPEWNQVFAFSRETVQSSVLEVVVKDKDLVKDDSVGFVRIDLHEVPTRVPPDSPLAPEWYRLEDKKGEKKKGELMLAVWYGTQADEAFPDAWHSDAIAPGDSTSVSPTYIRSKVYHSPRLWYVRVNVIEAQDLVPADKSRFPDAYVKVQIGNQILKTKPVQTRNMNPIWNEEFMFVAAEPFEDHLTFTVEDHAGPNKDETIGRTVIALNSIDRRADDRFVRTRWYNLEKSLSDAMDSDRAKKDKFHSRLHVCVCLDGGYHVLDESTQYSSDLRPTAKQLWKSSIGVLELGILNADGLQPMKTRDGKGASDTYCVAKYGHKWVRTRTIVNSLNPKYNEQYTWEVYDTATVLTVGVFDNSQIGGSDNTRDNKIGKVRIRISTLETGRVYTHCYPLLVLHPSGVKKLGELHLAIRFSSTSMVNMMSQYSRPLLPKMHYKRPLSVIQQDILRHQAVNIVAARLGRAEPPLRREVVEYMSDANAHLWSMRRSKANFLRLTSVFSGLFSAGNWFGEVCTWKNPITTVPVHILFAMLVCFPELILPTVFLYMFLIGVWNYRYRPRYPPHMNTSLSCADAVSPDELDEEFESFPASKSSDIVRMRYDRLRSVAGRIQTVVGDIATQGERLQALLSWRDPRATTIFVLLCLVAAIVLYVTPFQMIALLAGFYVMRHPRFRHKTPSAPINFFRRLPARTDSML

>GhMCTP8-A

MEKRMQSLNAQAPLLPGDHQEDYNLKDTSPQLGERWPNGGAFGGRGWINGGDRFTSTYDLVEQMFYLYVKVVKAKELPPSSVTGSCDPYVEVKLGNYKGRTKHFDRKSNPEWNQVFAFSKDRVQSSLLEVFVKDKEMAGRDDYVGRVVFDLNEVPTRVPPDSPLAPQWYRLEDRRGEGKVRGEVMLAVWMGTQADEAFPEAWHSDAASVHGEGVFNIRSKVYVSPKLWYLRVNVIEAQDVVANDRSRLPDVFVKAQIGNQVLRTKICPTRTPNPLWNEDLVFVTAEPFEEQLLITVEDRVHPSKEDVLGKISLPLNEFEKRLDHRPVNSRWFNLEKYGFGVMEGDRRKELKFSSRIHLRVCLEGGYHVLDESTMYISDQKPTAKQLWKQPVGILEVGILGAQGLLPMKMKDGRGSTDAYCVAKYGQKWVRTRTIMETFNPRWNEQYTWEVYDPCTVITLGVFDNSHLGGNSGGGSNAGRDARIGKVRIRLSTLEAHRTYTHSYPLLVLHPHGLKKMGELQLAIRFTTLSLANMIYVYGQPLLPKMHYLHPFTVNQVDNLRYQAMNIVARRLGRAEPPLRKEVVEYMLDVDSHMWSMRRSKANFFRIMSLVSGMVAIGQWFGSVCYWKNPITSVLVHILFLILVWYPELILPTLCFYMFLVGLWNYRYRPRYPPHMDTKLSWAESVNPDELDEEFDTFPTCKPHDVVRMRYDRLRSVAGRIQTVVGDIATQGERFESLLGWRDPRATSLFIVFCVCAAVVLYATPFRVVALVGGLYYLRHPRFRSKLPSVPSSFFKRLPARTDSLL

>GhMCTP8-D

MEKRMQSLYAQAPLLPGNHQEDYNLKDTSPQLGERWPNGGTFGGRGWMNGGDRFTSTYDLVEQMFYLYVRVVKAKELPPSSLTGSCDPYVEVKLGNYKGRTKHFDRKSNPEWNQVFAFSKDRVQSSLLEVFVKDKEMAGRDDYVGRVVFDLNEVPTRVPPDSPLAPQWYRLEDRRGEGKVRGEVMLAVWMGTQADEAFTEAWHADAASVHGEGVFNIRSKVYVSPKLWYLRVNVIEAQDVVANDRSRLPDVFVKAQIGNQVLRTKICPTRTPNPLWNEDLVFVTAEPFEEQLLITVEDRVHPSKEDVLGKISLPLNEFEKRLDHRPVNSRWFNLEKYGFGVMEGDRRKELKFSSRIHLRVCLEGGYHVLDESTMYISDQRPTAKQLWKQPVGILEVGILGAQGLLPMKMKDGRGSTDAYCVAKYGQKWVRTRTIMETFNPRWNEQYTWEVYDPCTVITLGVFDNSHLGGNSGGGSNAGRDARIGKVRIRLSTLEAHRTYTHSYPLLVLHPHGLKKMGELQLAIRFTTLSLANMIYIYGQPLLPKMHYLHPFTVNQVDNLRYQAMNIVARRLGRAEPPLRKEVVEYMLDVDSHMWSMRRSKANFFRIMSLVSGMVAIGQWFGSVCYWKNPITSVLVHILFLILVWYPELILPTLCFYMFLVGLWNYRYRPRYPPHMDTKLSWAESVNPDELDEEFDTFPTCRPHDVIRMRYDRLRSVAGRIQTVVGDIATQGERFESLLGWRDPRATSLFIVFCVCAAVVLYATPFRVVALVGGLYYLRHPRFRSKLPSVPSSFFKRLPARTDSLL

>GhMCTP9-A

MSNLKLGVDVVSAHNLLPKDGQGSSSAFVELYFDGQKYRTTIKEKDLNPVWNESFYFNISDPSMLHFHTLEAYVYNNIRGTNTRAFLGKVCLTGTSFVPYSDAVVLHYPLEKRGIFSRVRGELGLKVYITDDPSIKSSIPAPAVESLATNESHVTQIHAQTVQSSVMKDRVESSRHTFHHLPNPNLHQQDHHHHHHHSSDPAGHHHHVPKHIADEMKPEPPPPKLVRMYSAASAQPVDFALKETSPFLGGGRVVGGRVIHADKTASTYDLVERMHFLYVRVVKAGELPSVDVTGGIDPFVEVKVGNYKGITKHFEKKQNPEWNQVFAFSRDRMQASILEVVIKDKSMVKDETVGIVRFDINEVPLRVPPDSPLAPQWYRLEDKKGDKVKGELMLAVWIGTQADEAFSDAWHSDAATPVDSSPATFAVLRSKVYHSPRLWYVRVNIVEAQDLVPTEKNRFPDVYVKAQIGHQVLKTKPCQARTLNAIWNEDLLFVAAEPFEDHLALSVEDRVAPGKDEIIGRVIIPLNSVDKRADDRIIHSRWFNLEKPVAVDVDQLKKEKFSSRIHLRVCLDGGYHVLDESTHYSSDLRPTAKQLWRPPIGVLELGILNAVGLHPMKTRDGRGTSDTYCVAKYGHKWVRTRTLVDNLSPKYNEQYTWEVFDPATVLTVGVFDNSQLGEKGSNGNKDLKIGKVRIRISTLEAGRVYTHSYPLLVLHPTGVKKMGELHLAIRFTCISFVNMLYQYSRPLLPKMHYVRPFSVMQLDMLRHQAVNIVAARLGRAEPPLRKEVIEYMSDVDSHLWSMRKSKANFFRLMTVFSGLFAIGKWFGDICMWKNPITTVLVHVLFLMLACLPELILPTVFLYMFLIGVWNFRYRPRYPPHMNTKVSQAEAVHPDELDEEFDTFPTSKSPELVRMRYDRLRSVAGRIQTVIGDIATQGERFQALLSWRDPRATAIFITFCLIAAIVLFVTPFQVIAALAGFYAMRHPRFRYRLPAVPINFFRRLPARTDSML

>GhMCTP9-D

MSNLKLGVDVVSAHNLLPKDGQGSSSAFVELYFDGQKYRTTIKEKDLNPVWNESFYFNISDPSMLHFHTLEAYVYNNIRGTNTRAFLGKVCLTGTSFVPYSDAVVLHYPLEKRGIFSRVRGELGLKVYITDDPSIKSSIPLPAVESLATNESHVTRIHAQTVQSSVMNDRVESSRHTFHHLPNPNLHQQDHHHHHHSSDPAGHHHHVPKHIADEMKPEPPPPKLVRMYSAASAQPVDFALKETSPFLGGGRVVGGRVIHADKTASTYDLVERMHFLYVRVVKARELPSVDVTGGIDPFVEVKVGNYKGITKHFEKKQNPEWNQVFAFSRDRMQASVLEVVIKDKSMVKDETVGVVRFDINEVPLRVPPDSPLAPQWYRLEDKKGDKVKGELMLAVWIGTQADEAFSDAWHSDAATPVDSSPATFAVLRSKVYHSPRLWYVRVNIVEAQDLVPTEKNRFPDVYVKAQIGHQVLKTKPCQARTLNAIWNEDLLFVAAEPFEDHLVLSVEDRVAPGKDEIIGRVIIPLNSVDKRADDRMIHSRWFNLEKPVAVDVDQLKKEKFSSRIHLRVCLDGGYHVLDESTHYSSDLRPTAKQLWRPPIGVLELGILNAVGLHPMKTRDGRGTSDTYCVAKYGQKWVRTRTLVDNLSPKYNEQYTWEVFDPATVLTVGVFDNSQLGEKGSNGNKDLKIGKVRIRISTLEAGRVYTHSYPLLVLHPTGVKKMGELHLAIRFTCVSFVNMLYQYSRPLLPKMHYVRPFSVMQLDMLRHQAVNIVAARLGRAEPPLRKEVIEYMSDVDSHLWSMRKSKANFFRLMTVFSGLFAIGKWFGDICMWKNPITTVLVHVLFLMLACLPELILPTVFLYMFLIGVWNFRYRPRYPPHMNTKVSQAEAVHPDELDEEFDTFPTSKSPELVRMRYDRLRSVAGRIQTVIGDIATQGERFQALLSWRDPRATAIFITFCLIAAIVLFVTPFQVIAALAGFYAMRHPRFRYRLPAVPINFFRRLPARTDSML

>GhMCTP10-A

MAENCTRKLIVEICNAKNLMPKDGQGTASAYAIVDFDGQRRRTKTKFRDLNPVWDEKLEFLVHDIESMAAEMLEINLYNDKKMGKRSTFLGKVKLAGSVFVKAGEETLVYYPLEKRSVFSQIKGEIGVKVFYVDEEAPPAPAEPAAEQKAETAEEKPKEEEDKKEENVEEKKEEEEKPKEEPPKEEEKPNPPPAEASKSEDTTAAATPPPPPTEVENPPIAHKEEASSTKVVATKSKVETGKSSQLVINELELRSLSGDHNRIAYDLVNRMPFLYVRVVKAKRANKEPACPLHAKLVIGTHSIKTKSQIDKDWDQVFAFDKEGLNSSSLEVSVWAEEEKKEEQKEGDAATTATTVVVDNCLGSVSFDLQEVPKRVPPDSPLAPQWYSLESEKSPGNDVMVSVWVGTQADEAFQEAWQSDSGGLIPETRAKVYLSPKLWYLRLTVIQTQDLQLGSVSEPKVRSPELYVKAQLGAQLFKTSRTPVGSAWNEDLVFVAAEPFEPFLVVMVEDWSNGQLVGQAKIHVPSLERRTDDKTEPKSRWFNLVGAENKPYAGRIHVKACLEGGYHVLDEAAHVTSDVQAAAKQLAKPPIGLLDVGIRGASNLLPVKTKDGTRGTTDAYVVAKYGQKWIRTRTILDRFNPRWNEQYTWDVYDPCTVLTIGVFDNGRYKRDETGKPGRDLRIGKIRVRLSTLDTNKVYLNSYMLTVLLPNGAKKMGEIEIAVRFSCSSWLSLIQAYGTPLLPRMHYLRPLGPAQQDILRQTAMHIVTARLARSEPPLGQEVVQFMLDTDTHVWSMRKSKANWFRVVGCLSHAAILARWLDGISTWAHPPTTILVHVLLIAVVMCPQLVLSTIFMYAFLILALRFRYRMRVPHNVDLRLSYVDAVGPDELDEEFDGLPTTRSPNTVRFRYDRLRALASRAQTLLGDVAAQGERLEALFNWKDPRATGIFVVVCLFASLLFYVVPFKVFVLGSGFYYIRHPRFRGDMPSAPLNFFRRLPSLSDQIM

>GhMCTP10-D

MAENCTRKLIVEICNAKNLMPKDGQGTASAYAIVDFDGQRRRTKTKFRDLNPVWDEKLEFLVHDIESMAAEMLEINLYNDKKMGKRSTFLGKVKLAGSVFVKAGEETLVYYPLEKRSVFSQIKGEIGVKVFYVDEEAPPAPAEPAAEQKAETAEEKPKEEEDKKEENVEEKKEEEEKPKEEPPKEEEKPNPPPAEASKSEDTTAAATPPPPPPEVENPPIAHKEEASSTKVVATKSKAETGKSSQLVINELELRSLSGDHNRIAYDLIDRMPFLYVRVVKAKRANKEPACPLHAKLVIGTHSIKTKSQIDKDWDQVFAFDKEGLNSSSLEVSVWAEEEKKEEQKEGDAAATAATVVVDNCLGAVSFDLQEVPKRVPPDSPLAPQWYSLESEKSPGNDVMVSVWVGTQADEAFQEAWQSDSGGLIPETRAKVYLSPKLWYLRLTVIQTQDLQLGSGSEPKVRSPELYVKAQLGAQLFKTSRTPVGLAWNEDLVFVAAEPFEPFLVVMVEDWSNGQLVGQAKIHVPSLERRTDDKTEPKSRWFNLVGAENKPYAGRIHVRACLEGGYHVLDEAAHVTSDVQAAAKQLAKPLIGLLDVGIRGASNLLPVKTKDGTRGTTDAYVVAKYGQKWIRTRTILDRFNPRWNEQYTWDVYDPCTVLTIGVFDNGRYKRDEAGKPGRDLRIGKIRVRLSTLDTNKVYLNSYMLTVLLPNGAKKMGEIEIAVRFSCSSWLSLIQAYGTPLLPRMHYLRPLGPAQQDILRQTAMRIVTARLARSEPPLGQEVVQFMLDTDTHVWSMRKSKANWFRVVGCLSHAAILARWLHGVRTWAHPPTTILVHVLLIAVVMCPQLVLSTIFMYAFLILALRFRYRMRVTHNVDLRLSYVDAVGPDELDEEFDGLPTTRSPDTVRFRYDRLRALASRAQSLLGDVAAQGERLEALFNWKDPRATGIFVVVCLFASLLFYVVPFKVFVLGSGFYYIRHPRFRGDMPSAPLNFFRRLPSLSDQIM

>GhMCTP11-A

MNPATAANQKEEFKLKDTKPQLGERWPHGGTRGGGGWISSERATSTYDLVEQMFYLYVRVVKAKDLPTNPVTGTIDPYVEVKLGNYKGKTQHFEKKPNPEWHQVFAFSKEKIQSSILEVFVRDREMVGRDDYIGRVIFDMNEVPTRVPPDSPLAPQWYRLEDRRGESKVRGEVMLAVWMGTQADEAFPDAWHTDASSVQGEGVFNIRSKVYVSPKLWYLRVNVIEAQDVEPHDKSQLPQAFVKAQVGNQILKTKVCPQKTQNPMWNEDLIFVAAEPFEEQLYLTVENKVTSAKDEVMGRIILPLHIFERRLDHRAVHSKWFNLEKFGFGALEGDKRHELKFSSRIHLRVCLEGAYHVLDESTMYISDQRPTARQLWKNPIGILEVGILSAQGLQPMKNKEGKGSTDAYCVAKYGQKWVRTRTIIESLNPKWNEQYTWEVYDPCTVITLGVFDNNHLGGSGGKNDSRIGKVRIRLSTLETDKIYTHSYPLLVLQPSGLKKMGELQLAVRFTCLSLANMIYLYSHPLLPKMHYLHPFTVNQLDSLRYQAMNIVAVRLGRAEPPLRKEVVEYMLDVDSHMWSMRRSKANFFRIVSLFSGVIAMSKWLGEVCQWKNPITTILVHVLFFILICYPELILPTMFLYMFLIGIWNYRYRPRHPPHMDTKLSWAEVAHPDELDEEFDTFPTSKAQDVIRMRYDRLRSVAGRIQTVVGDMATQGERFLALLSWRDPRATSLFVLFCLVAAVALYVTPFKIMALVAGLYWLRHPRFRSKLPSVPSNFFRRLPSRADSML

>GhMCTP11-D

MNPAAAANQKEEFKLKDTKPQLGERWPHGGTRGGGGWISSERXQHWKQIRFIPTLTLFSSYNHLDRMGELQLAVRFTCLSLANMIYLYSHPLLPKMHYLHPFTVNQLDSLRYQAMNIVAVRLGRAEPPLRKEVVEYMLDVDSHMWSMRRSKANFFRIVSLFSGVIAMSKWLGEVCQWKNPITTILVHVLFFILICYPELILPTMFLYMFLIGIWNYRYRPRHPPHMDTKLSWAEVAHPDELDEEFDTFPTSKAQDVIRMRYDRLRSVAGRIQTVVGDMATQGERFLALLSWRDPRATSLFVLFCLVAAVALYVTPFKIMALVAGLYWLRHPRFRSKLPSVPSNFFRRLPSRADSML

>GhMCTP12-A

MMPRPPPEDFALKETNPHLGGGKVSGDKLTSTYDLVEQMQYLYVRVVKAKDLPGKDVTGSCDPYVEVKLGNYLGKTRFFEKKSNPEWNQVFAFSKDRLQASLLEVTVKDKDFVKDDFIGKVFFDLNEIPKRVPPDSPLAPQWYRLEDRQGNKVKGELMLAVWMGTQADEAFPEAWHSDAAFVSGADGLANIRSKVYLSPKLWYLRVNVIEAQDLQPGDKGRYPEVFVKAILGNQALRTRISQARGINPMWNEDLMFVAAEPFEEPLILSVEDRVAPNKDEVLGKCAIPLQYVDRRLDHKPVNGRWFNLEKHVIIEGEKKKETKFASRIHMRICLEGGYHVLDESTHYSSDLRPTAKQLWKSSIGVLELGILNAHGLMPMKNKDGRGTTDAYCVAKYGQKWVRTRTIIDSFAPKWNEQYTWEVFDPCTVITIGVFDNCHLHGGDKAAGAKDTKIGKVRIRLSTLETDRVYTHSYPLLVLHPNGVKKMGEIHLAVRFSCSSLLNMMHMYSHPLLPKMHYLHPLTVSQLDSLRHQATQIVSMRLGRAEPPLRKEVVEYMLDVGSHMWSMRRSKANFFRIMNVLGGLIAVGKWFDQICNWKNPITTVLIHILFIILVLYPELILPTIFLYLFLIGVWYYRWRPRHPPHMDTRLSHADSAHPDELDEEFDTFPTSRPSDIVRMRYDRLRSIAGRIQTVVGDLATQGERLQSLLSWRDPRATALFVIFCLVAAIVLYVTPFQVVALLAGFYILRHPRFRHKLPSVPLNFFRRLPARTDSML

>GhMCTP12-D

MMPRPPPEDFALKETNPHLGGGKVSGDKLTSTYDLVEQMQYLYVRVVKAKDLPGKDVTGSCDPYVEVKLGNYLGKTRFFEKKSNPEWNQVFAFSKDRLQASLLEVTVKDKDFVKDDFIGKVFFDLNEIPKRVPPDSPLAPQWYRLEDRQGNKVKGELMLAVWMGTQADEAFPEAWHSDAAFVSGADGLANIRSKVYLSPKLWYLRVNVIEAQDLQPGDKGRYPEVFVKAILGNQALRTRISQARGINPMWNEDLMFVAAEPFEEPLILSVEDRVAPNKDEVLGKCAIPLQYVDRRLDHKAVNSRWFNLEKHVIVEGEKKKETKFASRIHMRICLEGGYHVLDESTHYSSDLRPTAKQLWKSSIGVLELGILNAHGLMPMKNKDGRGTTDAYCVAKYGQKWVRTRTIIDSFAPKWNEQYTWEVFDPCTVITIGVFDNCHLHGGDKAAGAKDTKIGKVRIRLSTLETDRVYTHSYPLLVLHPNGVKKMGEIHLAVRFSCSSLLNMMHMYSHPLLPKMHYLHPLTVSQLDSLRHQATQIVSMRLGRAEPPLRKEVVEYMLDVGSHMWSMRRSKANFFRIMNVLGGLIAVGKWFDQICNWKNPITTVLIHILFIILVLYPELILPTIFLYLFLIGVWYYRWRPRHPPHMDTRLSHADSAHPDELDEEFDTFPTSRPSDIVRMRYDRLRSIAGRIQTVVGDLATQGERLQSLLSWRDPRATALFVIFCLVAAIVLYVTPFQVVALLAGFYILRHPRFRHKLPSVPLNFFRRLPARTDSML

>GhMCTP13-A

MSEPDTDFSLKETSPNIGGGRVSGSEKLTSSFDLVEQLHFLYVKMVRAKGLLPSPCNPFVEVKVGNYEGTTKSLENNPDPEWNQVFAFSKDRIQAVTVEITVRNKESSTNDKVVGKLRFDIPDIPSRVPPDSPLAPQWYSLEDENGSKGERGELMLAIWMGTQADEVFPDAWHSDAASVNGESIANTRSKVYISPRLWYLRINIIQAQDLVPMNNKRIPQVYVKAMLGNMALRSRFSPDKSLNPTWNEDLMFVAAEPFDDPLMLSVVDKLSDNKEEVLGSCSIHLSKVEKRLMPIPIEANWYNLEQSVQDNRNNKEVRFASRLHLRVCLDGGYHVLDESIYYSSDFRATSKFLWPPAIGVLELGILNASGLLPMKSKNGRGTTDAYCVAKYGPKWVRTRTIVNSFAPKWNEQYTWDVYDPYTVITIGVFDNCHLQGGSNAVDSQDRRIGKVRIRLSTLNADRIYTLSYPLIVLEPNGVKKTGEIQLSVRFTCSSTWNLLQSYTQPLFPQMHYLLPLSVYQIESLRHQATHTLSSRLRRAEPPLQSEVVEYMLDVGSNVWSLRRGRANLERLLAAFNLLVEAWKWFDQIRKWKNPISTMAVHFLYSMLILFPDMILPLVFLICVVHGASQYRKRPRHPPHMDTKLSLVESVQSDDFDEEFDTFPTSKNVKVLKKRYDRLRSIAGRMMTIIGDLATQAERLNSMLSWRDPRATSLFMAFCLIACIAFYLVPWRLFALGFGFFGMRHPRFRISIPSMPQNFFKRLPARTDSMI

>GhMCTP13-D

MSEPDIDFSLKETSPNLGGGRVSGSEKLTSSFDLVEQLHFLYVKMVRAKGLPPSPCNPFVEVKVGNYEGTTKSLENNPAPEWNQVFAFAKDRIQAVTVEITVRNKESSTNVDKVVGKLRFDIPDIPSRVPPDSPLAPQWYSLEDENGSKGERGELMLAIWMGTQADEAFPDAWHSDAASVNGESIANTRSKVYISPRLWYLRINIIQAQDLVPMNNKRIPQVYVKAMLGNMALRSRFSPDKSLNPTWNEDLMFVAAESFDDPLMLSVVDKLSDNKEEVLGSCSIHLSKVEKRLMPIPIEANWYNLEQSVQDNRNNKEVRFASRLHLRVCLDGGYHVLDESIYYSSDFRATSKFLWPPAIGVLELGILNASGLLPMKSKNGRGTTDAYCVAKYGPKWVRTRTIVNSFAPKWNEQYTWDVYDPYTVITIGVFDNCHLQGGSNAVDSQDRRIGKVRIRLSTLNTDRIYTLSYPLIVLEPNGVKKTGEIQLSVRFTCSSTWNLLQSYTQPLFPQMHYLLPLSVYQIESLRHQATHTLSSRLRRAEPPLQSEVVEYMLDVGSNVWSLRRGRANLERLLAAFNLLVEAWKWFDQIRKWKNPISTMAVHFLYSMLILFPDMILPLVFLMCVVHGASQYRKRPRHPPHMDTKLSLVESVQSDDFDEEFDTFPTSKNVKVLKKRYDRLRSIAGRMMTIIGDLATQAERLNSMLSWRDPRATSLFMAFCLIACIAFYLVPWRLFALGFGFFGMRHPRFRISIPSMPQNFFKRLPARTDSMI

>GhMCTP14-A

MSSFKLGVEVVGAHDLVAKDGQGSSNPFVELHFDDQRFRTTTKENDLNPVWNESFYFNISDPNNLIKLPLEAYVYSHNKANNTKTCLGKLRLTGTSFVPYSDAVVMHYPLEKRAIFSHAQGELGLKVFLTDDPSIKLSNPLPAMESFLDTDVGSGYAQTPNFPSSLPKEKTDKRHTFHHLPNANHSQQKQNFTPMPPQQQMNYGVHEMKSEPQAAKVVHMFSGSSSHSQPSDYALKETSPFLGGGRIIGGRVIRGDRPTSTYDLVEQMRYLFVRVVKARDLPSKDLTGSLDPYVEVKVGNYKGITKHYERNQNPEWNQVFAFARETMQSTVLEVVLKDKDLVKDDFVGIVRFDLHEVPMRVPPDSPLAPEWYRLQDKKGEKKKGELMLAVWYGTQADETFPDAWHSDAFAPGDSTSIASPYIRSKVYHSPRLWYVRVNVIEAQDLVPSDKNRFPDVYVKVQIGNQILKTKTVQPRNLNPIWNEEFMFVTSEPFEDHLIFSVEDRVGSNKDETMGKAVVPLNSVEKRADDRVVRTRWYNLEKSLSDAMVGDRAKKDKFHSRLHVCVCLDGGYHVLDESTQYSSDLRPTAKQLWKSSIGVLELGILNADRLQPMKTKDGKGTSDTYCVAKYGHKWVRTRTIVNSLNPKYNEQYTWEVYDPATVLTVGVFDNCQIGDSNGNKDIKIGKVRIRISTLETGRVYTHSYPLLVLHPSGVKKMGELHLAIRFSSTSMLSMMFQYSRPLLPKMHYKRPLSVIQQDMLRYQAVNIVAARLGRAEPPLRREVIEYMSDANAHLWSMRRSKANFLRLTSVFSGLFEVGKWFGEVCKWKNPMTTVLVHFLFVMLVCFPELILSTVFLYMFLIGVWKYRYRPRYPPHMDKSLSCADAVSPDELDEEFESFPASRSSDLVRMRYDRLRSVAGRVQSVVSDIANQGERLQALLSWRDPRATTIFVIFCLAAAIVLYVTPFQVFALIAGFYCMRHPRFRHKIPAAPINFFRRLPARTDSML

>GhMCTP14-D

MSSFKLGVEVVGAHDLVAKDGKGSSNPFVELHFDDQRFRTTSKENDLNPVWNESFYFNISDPNNLIKLPLEAYVYSHNKANNTKTCLGKLRLTGTSFVPYSDAVVMHYPLEKRTIFSDAKGELGLKVFLTDDPSIKLSNPLPAMESFLDTDVGSGYAQTPNFPSSLPKEKTDKRHTFYHLPNANHSQQKQNFTPMPPQQQMNYGVHEMKSEPQAAKVVHMFSGSSSHSQPSDFALKETSPFLGGGRIIGGRVIRGDRPTSTYDLVEQMRYLFVRVVKARDLPSKDLTGSLDPYVEVKVGNYKGITKHYERNQNPEWNQVFAFARETMQSTVLEVVLKDKDLVKDDFVGIVRFDLHEVPMRVPPDSPLAPEWYRLQDKKGEKKKGELMLAVWYGTQADETFPDAWHSDAFAPGDSTSIASPYIRSKVYHSPRLWYVRVNVIEAQDLVPSDKNRFPDVYVKVQIGNQILKTKTVQPRNLNPIWNEEFMFVTSEPFEDHLIFSVEDRVGSNKDETMGKAVVPLNSVEKRADDRVVRTRWYNLEKSLSDAMDGDRAKKDKFHSRLHVCVCLDGGYHVLDESTQYSSDLRSTAKQLWKSSVGVLELGILNADRLQPMKTKDGKGTSDTYCVAKYGHKWVRTRTIVNSLNPKYNEQYTWEVYDPATVLTVGVFDNCQIGDSNGNKDIKIGKVRIRISTLETGRVYTHSYPLLVLHPSGVKKMGELHLAIRFSSTSMLSMMFQYSRPLLPKMHYKRPLSVIQQDMLRYQAVNIVAARLGRAEPPLRREVIEYMSDANAHLWSMRRSKANFLRLTSVFSGLFEVGKWFGEVCKWKNPMTTVLVHFLFVMLVCFPELILSTVFLYMFLIGVWKYRYRPRYPPHMDTSLSCADAVSPDELDEEFESFPASRSSDLVRMRYDRLRSVAGRVQSVVSDIANQGERFQALLSWRDPRATTIFVIFCLAAAIVLYVTPFQVFALIAGFYCMRHPRFRHKIPAAPINFFRRLPARTDSML

>GhMCTP15-A

MATTQKLVVEVVDARNLVPKDGHGTSSPYVVVDYYGQRKRTRTVTRDINPTWNEVLQFNVEKPSDVFSDMLELDVYHDKNHGPTSRQNSLGRLKLSSGQFVKKGEEALIYYPLEKKYLLSWIRGEIGLKIYYVNDVPPPPPLPVEEAKAKEGKVKPPSDNAAAPVQSDQQPPPAEVKPDDNAASPPADGESVKAVEAASAGEEKGEAEGEKPAEGYDQVEPEADSKSVQADDHDDDIVLDPTSANWVPPPQSGIMASVTSGSIPEIKVTRTTSAPPPFTQPATPASYASSVDPPDHTRIERSSFDLVEKMHYVFVKVVKAGNLPTNGSPIVKIAAAGCHVTSKPARKAKYFEWDQTFAFARDTPETSSFLEVSVWADAGGANFLGGVCFDATEIPLRDPPDSPLAPQWYRLEGGGAHRGDLMLATWIGTQADEAFPDSWKSDAVGNLVSSRAKIYLSPKLWYLRASVLEAQDILPLTALKEGSFQMKAQLGFQVQKTKASISRNGNPSWNEDLMFVAAEPFAEHLIFYLEYRQQKGPVTLGVVGIPLTSIERRVDDRKVISRWFNLEDPKSDKKGYKGRVHLSLCFDGGYHVMDEAAHVCSDYRPTARQLWKPPVGTLELGIIGCKNLLPMKTINSKGSTDAYTVAKYGSKWVRTRTVSDSLDPKWNEQYTWNVYDPCTVLTIGVLDSWGALDIDSGKEATRPDFRMGKVRIRVSTLETGKVYKNTYPLVMLGNSGVQKMGELEVAVRFVRAAPTLDFLHVYSQPLLPLMHHIKPLGMPQQEMLRSTSVKILAAHLSRSEPPLRSEVVRYMLDADSHTFSMRKVRANWSRIVNVLSCLIDMVRWIEDTRMWKNPTATILVHALLVMLVWFPDLIIPTLAFYVFAIGVWNYRFRSKEKLPHFCPKMSLADSVDRDELDEEFDTMPSTRSPDIIRTRYDKLRAIGARVQNILGDVATQGERVQALVTWRDPRATAIFVGLCLMVAFVLYLVPPKMIAMAFGFYYFRHPVFRDQMPSPALNFFRRLPSLSDKIM

>GhMCTP15-D

MATTQKLVVEVVDARNLVPKDGHGTSSPYVVVDYYGQRKRTRTVTRDINPTWNEVLQFNVEKPSDVFSDMLELDVYHDKNHGPTSRQNSLGRLKLSSGQFVKKGEEALIYYPLEKKYLLSWIRGEIGLKIYYVDDVPPPPPPPVEEAKEEEGKVEPPSDNAAAPVPSDQQPPPAEVKPDDNAANPPADGESVKAVEAAPVGEEKGEAEGQKPAEGYDQVEPEADSKSEQADDHDDDIVLDPTSANWVPPPQSGIMASATSGSIPEIKVTRTTSAPPPLTRPATAASYVSSVDPPDHTRIERSSFDLVEKMHYVFVKVVKAGSLPTNGNPTVKIAVAGCHATSKPARKAKYFEWDQTFAFARDTPETSSFLEVSVWADAGGANFLGGVCFDATEIPLRDPPDSPLAPQWYRLEGGGAHRGDLMLATWIGTQADEAFPDSWKSDAAGNLVNSRAKIYLSPKLWYLRASVLEAQDILPLTALKEGSFQMKAQLGFQVQKTKASVSRNGNPSWNEDLMFVAAEPFAEHLIFYLEYRQQKGPVTLGVVGIPLTSIERRVDDRKVISRWFNLEDPKSDKKGYKGRVHLSLCFDGGYHVMDEAAHVCSDYRPTARQLWKPPVGTLELGIIGCKNLLPMKTINSKGSTDAYTVAKYGSKWVRTRTVSDSLDPKWNEQYTWKVYDPCTVLTIGVFHSWGALDIDSGKEATRPDFRMGKVRIRVSTLETGKVYKNIYPLVMLGNSGVQKMGELEVAVRFVRAAPTLDFLHVYSQPLLPLMHHIKPLGMPQQEMLRSTSVKILAAHLSRSEPPLRSEVVRYMLDADSHTFSMRTVRANWSRIVNVLSCLIDMVRWIEDTRMWKNPTATILVHALLVMLVWFPDLIIPTLAFYVFAIGVWNYRFRSKEKLPHFCPKMSLADSVDRDELDEEFDTMPSTRSPDIIRTRYDKLRAIGARVQNILGDVATQGERVQALVTWRDPRATAIFVGLCLMVAFVLYLVPPKMIAMAFGFYYFRHPLFRDRMPSPALNFFRRLPSLSDKIM

>GhMCTP16-A

MAENCTRKLIVEICNANNLMPKDGQGTASAYAIVDFAGQRRRTKTKFRDLNPVWDEKLEFLVHDIGSMASEILEINLYNDKKIGKRSNFLGKIKLAGTVFVSAGAESLVYYSLEKRSVFSQVKGEIGVKIFYVDEEAAPTSAEQKEETAEEKPPEEEKPAEENKEEAPKEEEKPNPPPPESSNPQDAVAASTSETNPAPEEENNQPLANKEEPPTETAKDKAETGKSTELIINNLELRSLSGDHNRMGYDLVDPMPFLYVRVVKAKVANKELACPLHAKIVIGTHSIKAKTQINRDWDQVFAFDKEGLNSSSLEVSVWTEEGKNEEAPSSLVESWLGTVSFDLQEVPKRVPPDSPLAPQWYSLESESETGNDVMVAVWVGTQADEAFQEAWQSDSGGLIPETRAKVYLSPKLWYLRLTVIQTQDLQLDLVSEAKVRSPEIYVKALLGAQLFKTSRTEVGSAWNEDLVFVAAEPFEPFLVVKVEDASNRQSVGDVKIHVSSIDRRTDDKTEVKSRWFNLIGGENKPYAGRIHVRVCLEGGYHVLDEAAHVTSDVRATAKQLTKPPIGLLDVGIRGANNMLPVKTKDAIRFCCSSWLSLIQAYGSPMLPRMHHVSPLGPAQQDILRYTAMRIVTARLARSEPALGQEVVQFMLDSDTHVWSMRRSKANWFRVVGCLSRVASLVRWLDEIRTWVHPPTTVLVHGLLVAAVVCSHLVLPTTFMYAFLILALRLRYCRRIPYNVESRLSYVDVESPDELDEEFDEFLTNKSSDTIRIRYDRLRVLASRIQTLLGDVAVQGERLEALFSWKDPRATGIFLVFCLFVSLLFYLVPLKVLVLGAGFYYIRHPRFRDDMPSLPINFFRRLPSLSDQIM

>GhMCTP16-D

MAENCTRKLIVEICNANNLMPKDGQGTASAYAIVDFAGQRRRTKTKFRDLNPVWDEKLEFLVHDIGSMASEILEINLYNDKKIGKRSNFLGKIKLAGTVFVSAGAESLVYYSLEKRSVFSQVKGEIGVKIFYVDEEAAPTPPEQKEETAEEKPPEEEKPAEENKEEAPKEEEKPNPPPPESSNPQDAVAASTSETNPAPEEENNQPLANKEEPPSETAKYKAETGKSTELIINNLELRSLSGDRNHIGYDLVDPMPFLYVRVVKAKVANKEPACPLHAKIVIGTHSIKTKTQIDRDWDQVFAFDKERLNSSSLEVSVWTEEGKNEEAPSSLAESCLGTVPFDLQEVPKRVPPDSPLAPQWYSLESESETGNDVMVAVWVGTQADEAFQEAWQSDSGGLIPETRAKVYLSPKLWYLRLTVIQTQDLQLDLVSEAKVRSPEIFVKALLGAQLFKTSRTQVGSAWNEDLVFVAAEPFEPFLVVKVEDASNGQSVGEVKIHVSSIDKRTDDKTEVKSRWFNLIGGENKPYAGRIHIRVCLEGGYHVLDEAAHVTSDVRATAKQLTKPPIGLLDVGIRGANNLLPVKTKDGPCGTTDAYVVAKYGPKWIRTRTILDRFNPHWNEQYTWDVYDSCTVLTIGVFDNGRYKGDKDVSIGKVRLRLSTLDMNKVYRNSHTLTVLSPDGVKKMGEIEIAIRFCCSSWLSLIQAYGSPMLPRMHHVSPLGPAQQDILRYTAMRIVTARLARSEPALGQEVVQFMLDSDTHVWSMRRSKANWFRVVGCLSRVASLVRWLDEIRTWVHPPTTVLVHGLLVAAVVCSHLVLPTIFMYAFLILALRLRYCRRIPYNVESRLSYVDVVSPDELDEEFDEFSTNKSSDTIRIRYDRLRALASRVQTLLGDVAVQGERLEALFSWKDPRATGILLVFCLFVSLLFYLVPLKVLVLGAGFYYIRHPRFRDGMPSIPINFFRRLPSLSDQIM

>GhMCTP17-A

MQKPPQSIDFALKETSPKIGAGAVTGDKLSSTYDLVEQMQYLYVRVVKAKDLPGKDVTGGCDPYVEVKLGNYKGVTKHFEKKNNPEWNQVFAFSKERIQASVLEVYVKDKDVVADDLIGRALFDLNEIPKRIPPDSPLAPQWYRLEDRKGDKAKGELMLAVWMGTQADEAFPEAWHSDAASVGPDAVASIRSKVYLSPKLWYVRVNVIEAQDLVPNDKSRFPEVFVKAMLGNQALRTRISQSKTINPMWNEDLMFVVAEPFEEPLVLSVEDRVGGNKDETLGKCVIPLHIVQRRLDHRPVNSQWLNLEKHVIVDGEKKEIKFASRIHLRICLEGGYHVLDESTHYSSDLRPTAKQLWRPSIGILELGILSAHGLMPMKTKDGRGTTDAYCVAKYGQKWIRTRTIVDNFMPRWNEQYTWEVFDTCTVITVGVFDNGHVHGGAGGARDARIGKVRIRLSTLEADRVYTHSYPLLVLHPSGVKKTGEIQLALRFTCSSLINMLHMYSHPLLPKMHYIHPLSVIQLDVLRHQAMQIVSTRLSRAEPPLRKEVVEYMLDVDSHMWSMRRSKANFFRIMGVLSGLIAVGKWFDQICNWRNPLTTILIHILFIILVLYPELILPTIFLYLFLIGIWNYRWRPRHPPHMDTRLSHADAAHPDELDEEFDTFPTSRPSDIVRMRYDRLRSIAGRVQTVIGDLATQGERFQSLLSWRDPRATTLFVTFCLIAALVLYVTPFQVVALLAGIYVLRHPRFRHKLPSTPLNFFRRLPARSDSML

>GhMCTP17-D

MQKPPQSIDFALKETSPKIGAGAVTGDKLSSTYDLVEQMQYLYVRVVKAKDLPGKDVTGGCDPYVEVKLGNYKGVTKHFEKKNNPEWNQVFAFSKERIQASVLEVYVKDKDVVADDLIGRALFDLNEIPKRIPPDSPLAPQWYRLEDRKGDKAKGELMLAVWMGTQADEAFPEAWHSDAASVGPDAVASIRSKVYLSPKLWYVRVNVIKAQDLVPNDKSRFPEVFVKAMLGNQALRTRISQSKTINPMWNEDLMFVVAEPFEEPLVLSVEDRVGGNKDETLGKCVIPLHIVQRRLDHRPVNSQWLNLEKHVIVDGEKKEIKFASRIHLRICLEGGYHVLDESTHYSSDLRPTAKQLWRPSIGILELGILSAHGLMPMKTKDGRGTTDAYCVAKYGQKWIRTRTIVDNFMPRWNEQYTWEVFDTCTVITVGVFDNGHVHGGAGGARDARIGKVRIRLSTLEADRVYTHSYPLLVLHPSGVKKTGEIQLALRFTCSSLINMLHMYSHPLLPKMHYIHPLSVIQLDVLRHQAMQIVSTRLSRAEPPLRKEVVEYMLDVDSHMWSMRRSKANFFRIMGVLSGLIAVGKWFDQICNWRNPLTTILIHILFIILVLYPELILPTIFLYLFLIGIWNYRWRPRHPPHMDTRLSHADAAHPDELDEEFDTFPTSRPSDIVRMRYDRLRSIAGRVQTVIGDLATQGERFQSLLSWRDPRATTLFVTFCLIAALVLYVTPFQVVALLAGIYILRHPRFRHKLPSTPLNFFRRLPARSDSML
